# Design of Fluorescent Coumarin-Hydroxamic Acid Derivatives as Inhibitors of HDACs: Synthesis, Anti-Proliferative Evaluation and Docking Studies

**DOI:** 10.3390/molecules25215134

**Published:** 2020-11-04

**Authors:** Santiago García, Itzel Mercado-Sánchez, Luis Bahena, Yolanda Alcaraz, Marco A. García-Revilla, Juvencio Robles, Nancy Santos-Martínez, David Ordaz-Rosado, Rocío García-Becerra, Miguel A. Vazquez

**Affiliations:** 1 Departamento de Química, Universidad de Guanajuato, Guanajuato, Gto. 36050, Mexico; mares.santiago22@gmail.com (S.G.); im.mercadosanchez@ugto.mx (I.M.-S.); luisbahena@unam.mx (L.B.); magarcia@ugto.mx (M.A.G.-R.); 2 Departamento de Farmacia, Universidad de Guanajuato, Guanajuato, Gto. 36050, Mexico; yolaalca@ugto.mx (Y.A.); robles__j@hotmail.com (J.R.); 3 Departamento de Biología de la Reproducción Dr. Carlos Gual Castro, Instituto Nacional de Ciencias Médicas y Nutrición Salvador Zubirán, Ciudad de México 14080, Mexico; santos_2105@hotmail.com (N.S.-M.); david.ordazr@incmnsz.mx (D.O.-R.); 4 Departamento de Biología Molecular y Biotecnología, Instituto de Investigaciones Biomédicas, Universidad Nacional Autónoma de México, Ciudad de México 04510, Mexico; rocio.garciab@iibiomedicas.unam.mx

**Keywords:** coumarins, hydroxamic acids, HDAC, gene expression, fluorescent probe, docking analysis

## Abstract

Coumarin-hydroxamic acid derivatives **7a**–**k** were herein designed with a dual purpose: as antiproliferative agents and fluorescent probes. The compounds were synthesized in moderate yields (30–87%) through a simple methodology, biological evaluation was carried out on prostate (PC3) and breast cancer (BT-474 and MDA-MB-231) cell lines to determine the effects on cell proliferation and gene expression. For compounds **7c**, **7e**, **7f**, **7i** and **7j** the inhibition of cancer cell proliferation was similar to that found with the reference compound at a comparable concentration (10 μM), in addition, their molecular docking studies performed on histone deacetylases 1, 6 and 8 showed strong binding to the respective active sites. In most cases, antiproliferative activity was accompanied by greater levels of cyclin-dependent kinase inhibitor p21, downregulation of the p53 tumor suppressor gene, and regulation of cyclin D1 gene expression. We conclude that compounds **7c**, **7e**, **7f**, **7i** and **7j** may be considered as potential anticancer agents, considering their antiproliferative properties, their effect on the regulation of the genes, as well as their capacity to dock to the active sites. The fluorescent properties of compound **7j** and **7k** suggest that they can provide further insight into the mechanism of action.

## 1. Introduction

Coumarins are part of a flavonoid group that acts as a secondary metabolite in plants. These heterocyclic compounds are known to form part of a wide variety of natural and synthetic compounds showing diverse pharmacological activity. Such compounds include anti-inflammatory [1], antioxidant [2], hepatoprotective [3], antithrombotic [4], antiviral [5], antimicrobial [6,7,8], antituberculosis [9,10], anti-carcinogenic [11], antidepressant [12,13], antihyperlipidemic [14] and anticholinesterase agents [15,16], and they are usually associated with low toxicity.

**Figure 1:**
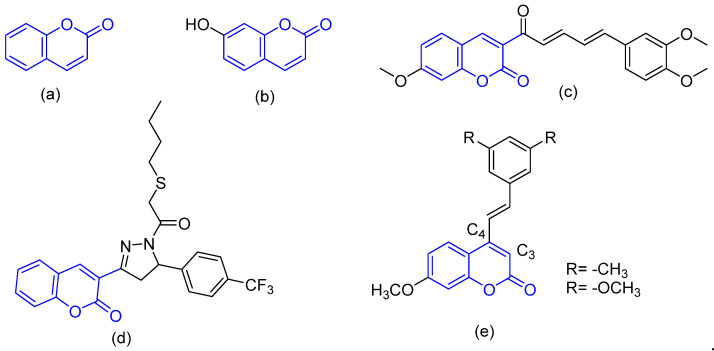
Library of structures with a coumarin core: (**a**) coumarin, (**b**) 7-hydroxycoumarin, (**c**) Siedel’s cytostatic coumarin, (**d**) Wu’s coumarin cytostatic agent, and (**e**) coumarin-stilbene.

**Figure 2:**
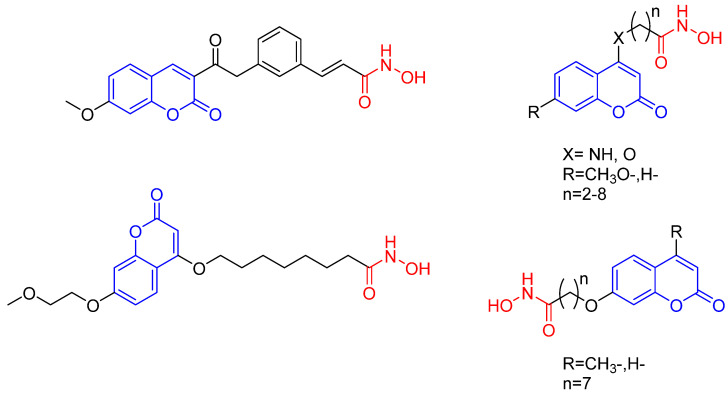
Examples for structures of coumarin and hydroxamic acids moieties that indicates the effectiveness in the application as anticancer agents.

Coumarins have been extensively examined as bioactive agents [17,18], today they are one of the most versatile types of compounds for anticancer drug design and discovery, as evidenced by several reports [19]. According to an in vitro study against renal cell carcinoma, for example, coumarin (Figure 1a) and 7-hydroxycoumarin (Figure 1b) are potent cytotoxic and cytostatic agents [20]. A common strategy for the optimization of antiproliferative activity is the functionalization of the coumarin core [21,22] (Figure 1c,d). A study of coumarins with stilbene revealed that the substitution pattern is the key to their potency. The shifting of *trans*-vinylbenzene from the C-4 to C-3 position in the coumarin ring leads to a two-fold decrease in activity [23] (Figure 1e). For the discussed above, the synthesis and characterization of coumarin based compounds is a relevant topic to be addressed. Even that, only a few recent studies include a computational characterization of coumarin based compounds as a HDAC Inhibitor. Regarding with this, Zhang and coworkers in recent research indicate the effectiveness of having a coumarin moiety as the main structure for its modification and application as anticancer agents, in combination with an hydroxamic acid functionalization to potentialize the activity (Figure 2) [24,25,26]. This kind of information is a strong background to embrace the hypothesis of the present work.

**Figure 3:**
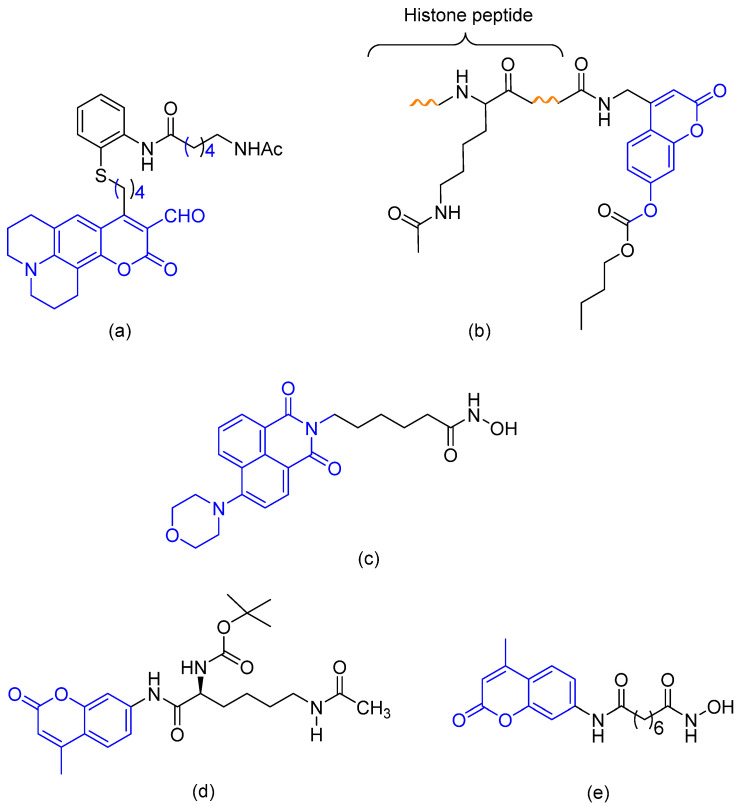
Fluorescent probes for the detection of HDAC activity. (**a**) Coumarin structure for real-time detection of HDAC activity, (**b**) K4(Ac)-CCB structure for monitoring of HDAC activity, (**c**) Scriptaid derivative (**d**) Coumarin probe that can access the catalytic site of HDAC, (**e**) Coumarin-SAHA derivatives.

**Figure 4:**
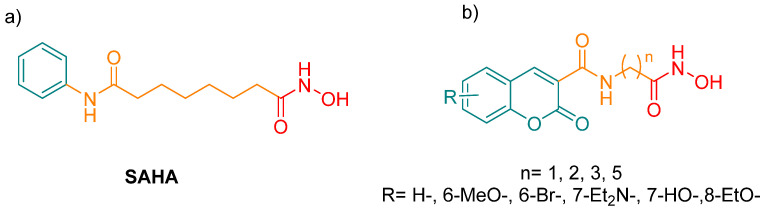
Structure of SAHA (C_14_H_20_N_2_O_3_) (**a**) and the synthesized test compounds (**b**).

Histone deacetylase (HDAC) is an amidohydrolase which deacetylates the histone lysine residues for chromatin remodeling and thus plays a vital role in the epigenetic regulation of gene expression, due to its over expression in several forms of cancer, HDAC is considered as a potential anticancer drug target. HDAC inhibitors are natural or synthetic small molecules that can alter the acetylation status to regulate various cellular events such as cell survival, differentiation and apoptosis in tumor cells and thus exhibit anticancer activity [27,28]. The application of coumarins, a well-known fluorophore [29,30,31], as fluorescent probes, has been described in various contexts, including the real-time (RT) detection of histone deacetylase (HDAC) activity by an acetyl-lysine functionalized mimic (Figure 3a) [32], as well as the monitoring of HDAC activity by K4(Ac)-CCB (Figure 3b) which consists of the histone H3 peptide containing acetyl-Lys and a coumarin fluorophore with a carbonate ester [33] and by a scriptaid derivative (Figure 3c). Unlike K4(Ac)-CCB, the scriptaid derivative bears the pharmacophore structure of HDAC inhibitors and has shown potential anticancer activity, as observed in the visualization of its distribution in MDA-MB-231 cells. Other reports have also described the use of coumarins as fluorescent probes and at the same time as HDAC inhibitors. Jung, M. et al. [34,35] developed probes with distinct structures that can access the catalytic site of HDAC in order to inhibit sirtuins (Figure 3d). Similarly, a coumarin-SAHA compound (Figure 3e) [36] based in the FDA approved structure suberoylanilide hydroxamic acid (SAHA) (Figure 4a) [37], was employed as a fluorescent probe for determining the *K*_d_ and *K*_off_ of HDAC8 enzyme-inhibitor complexes. Moreover, it was found that coumarin-thiophene derivatives induce the selective inhibition of HeLa cells (IC_50_ = 9.43 μM) [38]. In these studies, coumarin compounds used as fluorescent probes have provided insights into the mechanisms of action of HDACs. In addition, in a recent study Abdizadeh et al. [39] addresses the construction of a QSAR model to evaluate the biological activity of coumarin based benzamides as HDACs inhibitors. We think that the interdisciplinary work combining the synthesis, biological evaluation, and computational characterization of coumarin based compounds is a relevant issue.

There are few descriptions in the literature of coumarin derivatives that target HDACs in order to function simultaneously as a fluorescent probe and an antiproliferative agent. The aim of the current contribution was to design coumarin derivatives with this dual function. Two strategies were employed for the design of HDAC inhibitors: to improve the binding property of the capping group and to explore the influence of a flexible linker on binding (by the integration of an amide group containing an aliphatic linker of different chain sizes at the C-3 position). Hence, the principle characteristics of the present test compounds are similar to those of SAHA (Figure 4a). Evaluation was made of the effects of the derivatives on the proliferation of cancer cells as well as on the regulation of the p21, p53 and cyclin D1 (CDK1) genes. In addition, the subcellular location was established with two of these fluorescent analogues.

## 2. Results and Discussion

### 2.1. Chemistry

Based on the results of our previous studies [40,41], we envisioned the design of a compound with a dual purpose, serving as a fluorescent probe and an inhibitor of HDAC enzymes. For the former objective, the coumarin core was included in the compounds. For the latter, this core was structurally modified to improve ligand binding to the active site of the HDAC enzymes. To increase the interaction of the coumarin derivative with the external surface of the active site of the enzyme, the aromatic ring was substituted with different groups having distinct electronic characteristics.

**Scheme 1:**
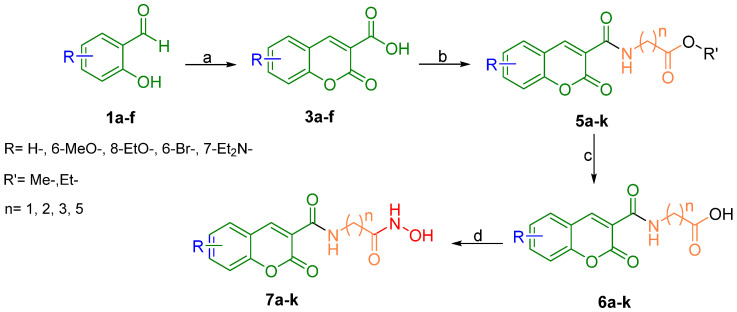
Reagent and reaction conditions. (**a**) Aldehyde (1 mmol), Meldrum’s acid (1.2 mmol), H_2_O, reflux, 5–6 h, 80–90%. (**b**) Compounds **3a**–**f** (1 mmol), amine hydrochloride (1.2 mmol), DMAP (5 mol%), CDI (1.1 mmol), DMF, rt, overnight, 46–90%. (**c**) Compounds **5a**–**k** (1 mmol), LiOH (7 mmol), THF/H_2_O (1/2), rt, 12 h, 50–93%; (**d**) Compounds **6a**–**k** (1 mmol), NH_2_OH/HCl (1.2 mmol), DMAP (5% mmol), CDI (1.1 mmol), DMF, rt, overnight, 30–87%.

The synthetic route for the new compounds is outlined in Scheme 1. Compounds **3a**–**f** were prepared in high yield by the condensation of Meldrum’s acid (**2**) and aldehydes **1a**–**f** [42,43]. After a recrystallization process, the carboxylic acids underwent a coupling reaction with various hydrochloride amine salts, and then with carbonyldiimidazole (CDI) to provide the esters **5a**–**k**. The hydrolysis of this series with lithium hydroxide gave the terminal carboxylic acids **6a**–**k**. The latter compounds were converted into hydroxamic acids by another coupling reaction with hydroxylamine hydrochloride and CDI to afford **7a**–**k** (Scheme 1, Table 1). The structure of the target compounds was confirmed by mass spectroscopy (HRSM) and NMR. Thus, a simple methodology was developed for the synthesis of the proposed molecules. It was tolerant to different functional substituents on the coumarin core. Water and filtration could be used as the only form of purification at all stages. These are attractive characteristics that facilitate the application of the methodology.

Absorbance and emission studies on **7a**–**k** were first carried out in MeOH before testing other solvents. Solubility turned out to be the limiting factor. In general, the peak of absorption at a concentration of 1 × 10^−6^ M was found between 283–419 nm. The emission peak at the same concentration and with the same solvent was observed at 405–470 nm (Table 1).

### 2.2. Biological Evaluation

Several HDAC inhibitors, such as SAHA have shown potent antiproliferative activities against various cancer cell lines, including human breast and prostate cancer cells [44,45,46]. Therefore, the latter two cancer cell lines were employed to evaluate the antiproliferative effect of compounds **7a**–**k**. The cells were incubated with different concentrations of SAHA or the newly synthesized compounds for 3–6 days. Depending on the cell line and the antiproliferative response, some of them were then assessed with the SRB assay. As expected, SAHA inhibited the proliferation of all cancer cell lines in a dose-dependent manner. Although the effect of compounds **7a**–**k** varied, most of them demonstrated powerful antiproliferative activity against the cell lines (Supplementary Material Figure S1). The growth inhibitory effect was tested at 10 μM, revealing SAHA-like activity for **7c**, **7e**, **7f**, **7i** and **7j**, and less potent activity for **7b** and **7h** (Table 2).

Greater sensitivity to antiproliferative activity was shown by the breast versus prostate cancer cells. Zhao et al. obtained similar results where coumarin-containing hydroxamate HDAC inhibitors were more potent to inhibit MDA-MB-231 cell proliferation compared with lung adenocarcinoma cell lines [26]. The compounds exhibiting the strongest antiproliferative activity were **7c**, **7e**, **7f** and **7i**, substituted with H, 6-MeO, 8-EtO and 6-Br, respectively. Interestingly, a common structural characteristic of the most active compounds **7c**, **7e**, **7f** and **7i**, is that they all possess the same side chain, comprising from five methylene groups, whereas the aromatic ring coumarin substituents vary. The compounds unable to modify cell growth were **7a**, **7d**, **7g** and **7k**, substituted with H, 6-MeO, 6-Br and 7-Et_2_N, respectively. Consequently, compounds **7c**, **7e**, **7f**, **7i** and **7j** (7-Et_2_N) could possibly be used as antineoplastic agents.

#### 2.2.1. Effects of SAHA Analogues on the Expression of Cell Cycle Regulatory Genes

SAHA suppresses growth and induces cell cycle arrest and apoptosis of human breast and prostate cancer cells. This occurs in part by the regulation of the proteins involved in the cell cycle and apoptosis, such as cyclin-dependent kinase (CDK) inhibitors p21, p53 and cyclin D1 (CD1) [45,46,47,48]. Therefore, the effect of compounds **7a**–**k** on the expression of cell cycle regulatory proteins was examined. 

Accordingly, breast and prostate cancer cells were treated in the presence and absence of the compounds having demonstrated the best antiproliferative activity (**7c**, **7e**, **7f**, **7i** and **7j**) and one that did not change cell growth (**7d**). In both the BT-474 and PC3 cell lines, most of the compounds produced an effect similar to SAHA, significantly increasing p21 gene expression and diminishing p53 and CD1 mRNA levels compared to the vehicle (Table 3). These results were not found in cells treated with **7i**. As expected, p21 and CD1 gene expression was not affected by **7d**, but mRNA levels of p53 were significantly decreased.

Overall, the antiproliferative activity of the compounds correlated well with gene regulation in the BT-474 and PC3 cancer cell lines. The exception was **7i**, which displayed growth inhibition comparable to that of SAHA at 10 μM (Table 2) but it did not change the expression of cell cycle regulatory genes. In this compound, due the enhanced lipophilicity character from Br substituent on the coumarin ring could modify its capacity to enter the nucleus of the cell, thus not allowing it to modulate genetic expression. Nevertheless, it showed activity against HDACs located in the cytoplasm or plasma membrane, such as HDAC3 [49,50]. 

According to the current findings, **7c**, **7e**, **7f**, **7i** and **7j** likely influence the enzymatic activity of HDACs [45,47,51], considering the antiproliferative properties of the compounds, their effect on the regulation of the p21, p53 and cyclin D1 genes, as well as their ability to dock at the active sites of HDAC1, HDAC6 and HDAC8. However, future research is needed to directly analyze the inhibition induced by the compounds on the enzymatic activity of HDACs.

#### 2.2.2. Novel Fluorescent SAHA Analogues

**Figure 5:**
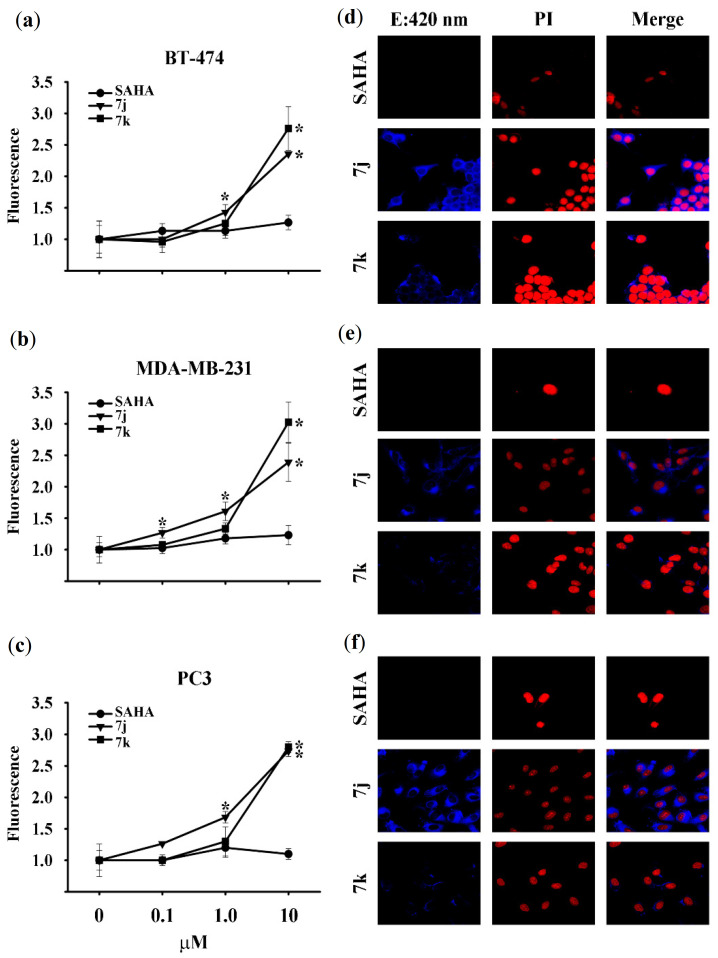
Relative fluorescence emission intensity and subcellular location of compounds **7j** and **7k**. (**a**–**c**) Breast and prostate cancer cells were treated in the presence (at different concentrations) and absence of the two compounds, followed by spectrofluorometric quantification. The value of vehicle-treated cells was set to one. * *p* < 0.05 vs vehicle. (**d**–**f**) Fluorescence microscopy of **7j** and **7k** in the three cell lines. Blue = **7j**, **7k**. Red = propidium iodide (PI). Blue/red = merged image of **7j**, **7k** and PI. Representative images from two independent experiments are shown.

Due to the inherent fluorescent properties of coumarins, an analysis of the excitability and subcellular location of compounds **7a**–**k** was carried out. The relative fluorescence emitted was evaluated after BT-474, PC3 and MDA-MB-231 cell lines were incubated with various concentrations of the compounds for 72–144 h. SAHA was used as the non-fluorescent negative control. Of the 11 compounds evaluated, the relative fluorescence was only significantly increased for **7j** and **7k**, compared to vehicle-treated cells (Figure 5a–c). Indeed, a significant fluorescence emission intensity was shown by **7j** as of 0.1 μM in MDA-MB-231 cells (Figure 5b). Data are expressed as the mean ± SD of determinations in triplicate from at least three distinct experiments. The value of vehicle-treated cells was arbitrarily set at one. 

The cells with a significant difference in relative fluorescence were exposed to 10 μM of **7j**, **7k** or SAHA and confocal microscopy images were taken (Figure 5d–f). Subsequently, to visualize the cellular penetration and subcellular location of **7j** and **7k**, confocal microscopy images were obtained for the three cell lines. There was a significant increase in fluorescent emission intensity (indicating cellular uptake), especially for compound **7j** (Figure 5d–f). With intracellular cytoplasmic localization and colocalization experiments using propidium iodide, a dramatic change was observed in the color of the nucleus in BT-474 cells (Figure 5d) and to a lesser extent in MDA-MB-231 and PC3 cells (Figure 5e,f), indicating that **7j** had penetrated the nucleus.

Since the fluorescence of **7k** was found in the cytoplasm but not the nucleus, this compound apparently did not penetrate the nuclear envelope of any of the three cell lines herein examined. Hence, the lack of antiproliferative activity of **7k** could be due to its inability to reach the nucleus as well as its limited cellular uptake. Compound **7j** is expected to be a valuable tool for future studies on the mechanism of action of HDAC inhibitors, given its fluorescent properties and antiproliferative activity.

### 2.3. Molecular Docking

The overexpression of HDAC1 in the BT-474 [52,53] and PC3 cell lines [44] is reported to promote the development of cancer. In the MDA-MB-231 cell line, contrarily, HDAC6 and HDAC8 play a critical role in proliferation [54]. Accordingly, such isoforms are ideal biological targets for docking experiments. Compounds **7c**, **7e**, **7f**, **7i** and **7j** were docked into the active site of HDAC1, HDAC6 and HDAC8 to gain insight into the ligand-receptor binding mode.

The total interaction energies are shown in Table 4. There is not a direct correlation between the energies and the biological activity. Nevertheless, the molecular coupling in the active site of the protein was successfully found, validating the docking methodology. We observe that the best pose of SAHA docked on HDAC8 displays a RMSD of about 0.51 Å, when it is compared with the co-crystallized structure. In addition, we used the study of the interactions between drugs and HDACs to get insight about the interaction mechanism.

**Figure 6:**
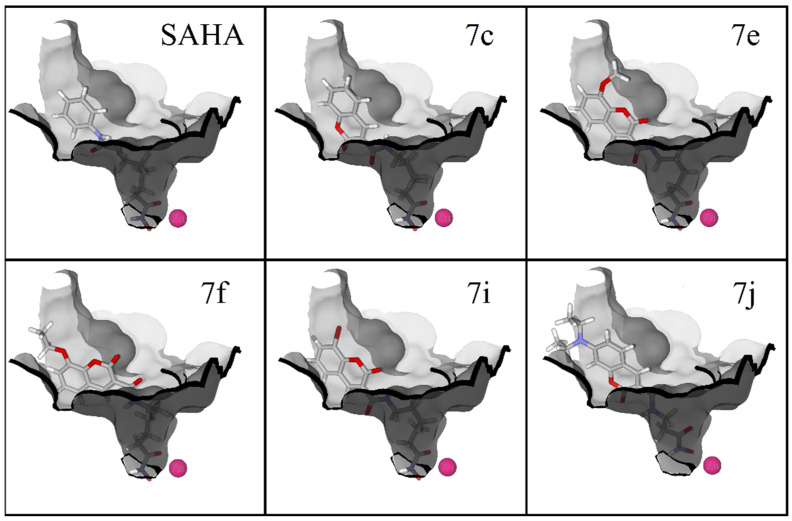
The binding modes for SAHA and compounds **7c**, **7e**, **7f**, **7i** and **7j** at the active site of HDAC1, according to molecular docking calculations. The ligands are illustrated with a tube model. Oxygen atoms are depicted in red, the bromine atom in dark red, carbon atoms in gray, nitrogen atoms in purple and the zinc atom in pink.

Regarding HDAC1, docking calculations demonstrated that four of the five compounds (**7c**, **7e**, **7f** and **7i**) have a binding mode like SAHA. For **7j**, the hydroxamic group inside the pocket is coordinated with the zinc atom in monodentate geometry. For all the other ligands, including SAHA, there is a bidentate chelation effect. The coumarin rings of all ligands cap the active site. For **7c** and **7j**, the coumarin rings expose the carbonyl group of the moiety to the enzyme, giving rise to acceptor hydrogen bond interactions. For **7e**, **7f** and **7i,** the carbonyl group of the moiety is positioned opposite to the enzyme surface (Figure 6).

**Figure 7:**
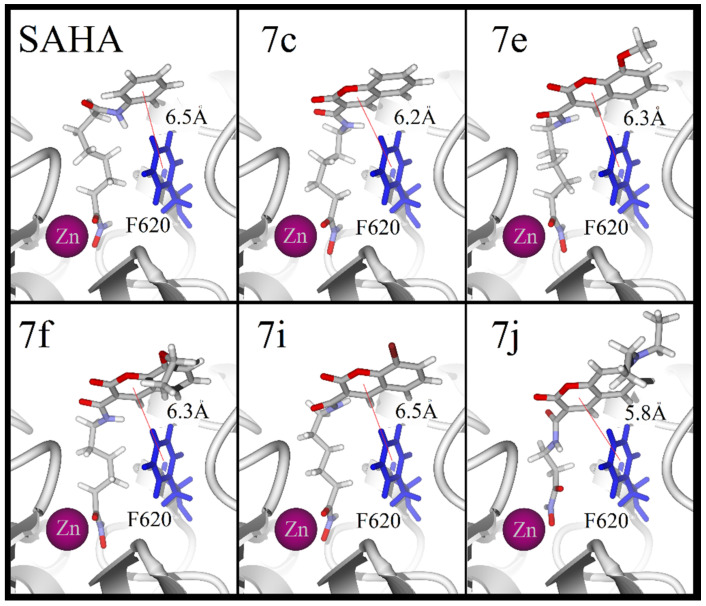
Potential T-stacking interactions between the coumarin ring of studied compounds (SAHA, **7c**, **7e**, **7f**, **7i** and **7j** center) and the phenylalanine residue (structure in blue labeled as F620) in the active site of HDAC6. T-stacking interactions are displayed as red lines, distances between interacting aromatic moieties are showed in Ångstroms (**Å**). Zn atom is presented as a pink sphere.

The docking simulations at the active site of HDAC6 evidence a potential T-stacking interaction between the phenylalanine ring and the coumarin ring of all ligands (**7c**, **7e**, **7f**, **7i**, **7j** and SAHA), with distances of about 5.5 Å. A bidentate coordination with the zinc atom was exhibited by four of these compounds and a monodentate coordination by **7j**. For all these compounds, the coumarin ring exposes the carbonyl group moiety in a very similar conformation (Figure 7).

**Figure 8:**
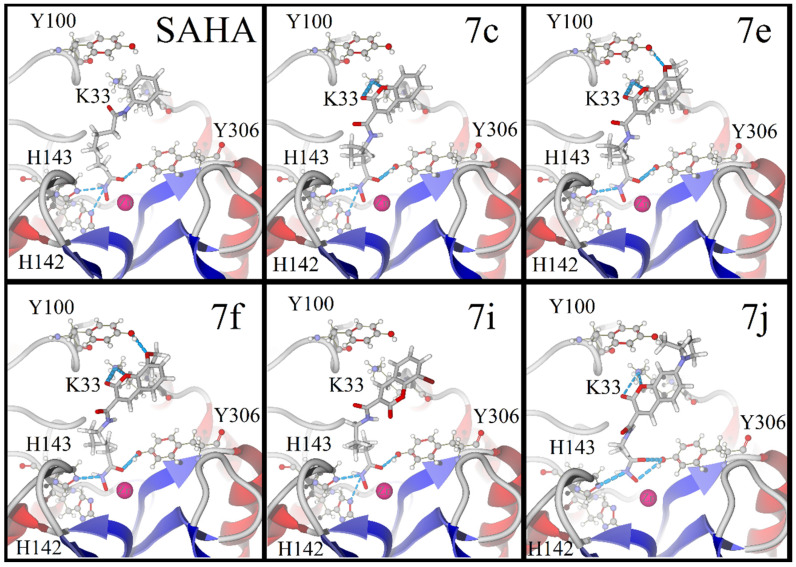
Hydrogen-bond interactions (blue lines) for SAHA, **7c**, **7e**, **7f**, **7i**, **7f** and **7j** in the pocket of HDAC8. Zinc atom displayed as a pink sphere.

Finally, when docking the five compounds at the active site of HDAC8, all the ligands except **7j** displayed a binding mode comparable to that of the reference drug. The fundamental similarity is the coordination between the hydroxamic group and the zinc atom. Compounds **7c** and **7i** had the strongest acceptor hydrogen bonds (and the largest quantity of the same) with histidine and tyrosine, and such bonds were formed at the bottom of the pocket. For **7c**, **7e** and **7f**, acceptor hydrogen bonds with lysine were detected higher up in the pocket, and for **7e** and **7f** an extra acceptor hydrogen-bond was observed with tyrosine outside the pocket. Contrarily, the halogen in **7i** promoted the opposite orientation of the coumarin rings, thus avoiding hydrogen bond interactions with lysine. For **7j**, on the other hand, there were two acceptor hydrogen bonds with tyrosine, two with lysine and one with histidine. However, it showed a monodentate coordination with the zinc atom (Figure 8). The binding properties of **7f** agree with its antiproliferative activity displayed by the biological test. It has several desirable properties that are common among HDAC inhibitors, including stacking interactions, a bidentate coordination with the atomic center, and a considerably number of hydrogen-bond interactions. 

**Figure 9:**
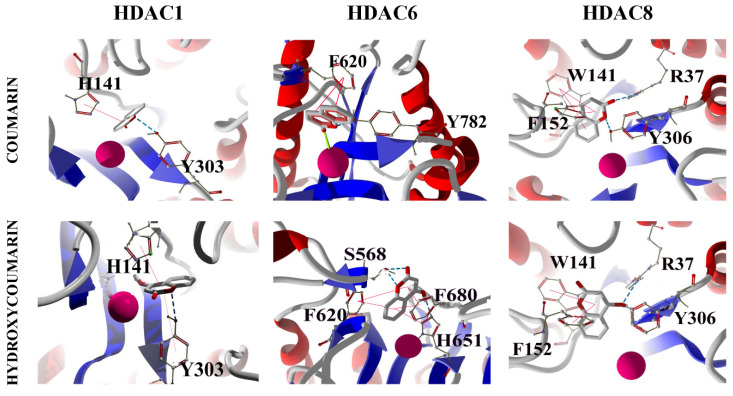
Potential interactions between the coumarin and 4-hydroxycoumarin and the residue in the active site of HDAC1, HDAC6 and HDAC8. The stacking interactions are show as a red line, hydrogen bond is blue dotted line and the coordination bond with Zinc is the green line.

It is important to compare the binding properties of tested molecules with the ones of coumarin and hydroxycoumarins (3, 4, 5, 6 and 7-hydroxycoumarins). The docking between coumarin and hydroxycoumarins and HDAC1, HDAC6 and HDAC8, revealed unions mainly of π-staking type. The aromatic groups of the amino acids of the enzymatic active site interact with the coumarin ring of both ligands via a π-staking interaction (Figure 9). An essential characteristic of HDAC inhibitors studied in this work is the coordination with the Zn (II) cofactor. Therefore, the docking experiments suggest that 4- and 7-hydroxycoumarins do not bind the cofactor at all, an example for this is displayed in Figure 9 lower panel. We detected that, for only some cases, there is a possible monodentate coordination with Zn (II) for coumarin and hydroxycoumarins. From these results, we conclude that SAHA and its analogues possess a pharmacological activity that coumarin and hydroxycoumarins cannot display by itself, since SAHA and analogs exhibit bidentate coordination between the hydroxamic functional group and Zn (II). Hence, the functionalization of the coumarin ring enlarges the possibility of chelation with the cofactor Zn (II). Such fact increases the possibilities of cancer cell growth inhibition. A detailed information of the binding energies of coumarin and hydroxycoumarins is located in the Supplementary Material. 

### 2.4. ADMET Properties for SAHA Analogues

The Absorption, Distribution, Metabolism, Excretion and Toxicity (ADMET) properties are relevant for the characterization of a drug candidate. In the present contribution, the determination of the physicochemical and pharmacokinetic properties of SAHA and analogues (**7c**, **7e**, **7f**, **7i** and **7j**) was carried out through a free web tool to evaluate pharmacokinetics: SwissADME [55]. In general, SAHA and its analogs display a large polar surface area (PSA) and good solubility in water. Regarding the partition coefficient between *n*-octanol and water (log Po/w), the classic descriptor of lipophilicity, SAHA and analogs are within the optimal range (−0.7, +5.0) (Table 5). Besides, the prediction for passive human gastrointestinal absorption (HIA) was found to be high, in contrast to the permeability of the blood-brain barrier (BBB) which was found negative for SAHA and analogues (Table 6). On the other hand, SAHA is a substrate for P-glycoprotein (P-gp), the same behavior is exhibited by **7e**, **7f**, **7i** and **7j**, except for the **7c**. The binding of a drug with P-gp is relevant, since this protein can export molecules to reduce its intracellular concentration below the effective cytotoxic threshold [56]. The above discussion can be related with the in vitro observations of a treatment with **7c**, in which the sensitivity of BT-474 breast cancer cells was higher than the rest of the analogues. As a matter of fact, in such, culture P-gp is overexpressed and for this reason it is capable to disperse all SAHA analogues except for the **7c**.

Therefore, it can be inferred that the new SAHA analogs exhibit good absorption, favorable permeability to the interior of the cell and low toxicity to the cytochrome P450 enzyme. It should be noted that in vitro and in vivo studies indicate that SAHA is not metabolized by the cytochrome p450 system, rather is metabolized by glucuronidation, hydrolysis and beta-oxidation [57]. In addition, SAHA is eliminated via renal [58].

## 3. Materials and Methods

### 3.1. Chemistry

All solvents and reagents were commercially available and used without further purification. Melting points were determined on a RY-1 MP apparatus. Absorption spectra were recorded on a Perkin-Elmer Lambda 50 apparatus (Perkin-Elmer Inc., Waltham, MA, USA), and Emission spectra on a FL-7000 FL spectrometer (Hitachi Inc., Chiyoda, Tokio, Japon). ESI-HRMS spectra were obtained on a Maxis Impact ESI-QTOF-MS spectrometer (Bruker Corporation 40 Manning Road Billerica, MA 01821, USA) and a Bruker Daltonics mass spectrometer (Bruker Corporation 40 Manning Road Billerica, MA 01821, USA). ^1^H-NMR and ^13^C-NMR spectra were acquired in CDCl_3_ or DMSO-*d*_6_ solutions with the following NMR spectrometers: a Bruker Avance III HD NMR spectrometer [Bruker Corporation 40 Manning Road Billerica, MA 01821, USA] with a Bruker Ascent 400 MHz magnet or a Bruker Avance III HD with a Bruker Ultra Shield 500 MHz HD magnet. Spectra were recorded at 25 °C with TMS and solvent signals allotted as internal standards. Chemical shifts are expressed in ppm (δ) and *J*-values in hertz.

#### 3.1.1. General Procedure for the Preparation of Coumarin-3-Carboxylic Acids (**3a**–**f**)

In a round-bottom flask, a solution of the corresponding salicylaldehyde (1 mmol) and Meldrum’s acid (1.2 mmol) was stirred in water at reflux for 5–6 h. The precipitate was obtained by filtration. The purification was carried out by recrystallization with MeOH and cold water [42,43].

*2-oxo-2H-chromene-3-carboxylic acid* (**3a**), white solid (82% yield); mp = 183–184 °C; IR (KBr): ν_max/cm_^−1^ = 1746, 1685, 1614, 1569; UV-Vis (MeOH): λ_max/nm_ = 296; ^1^H-NMR (500 MHz, CDCl_3_): δ 12.26 (s, 1H, H-3a), 8.96 (s, 1H, H-4), 7.80 (dd, *J* = 14, 7.7 Hz, 2H, H-5,8), 7.51 (t, *J* = 8.9 Hz, 2H, H-6,7); ^13^C-NMR (125 MHz, CDCl_3_): δ 164.10, 162.42, 154.59, 151.52, 135.79, 130.52, 126.28, 118.48, 117.23, 114.90; HRMS (ESI) *m*/*z* calcd. for C_10_H_6_O_4:_ [M + H]^+^ 191.0339, found 191.0342.

*6-methoxy-2-oxo-2H-chromene-3-carboxylic acid* (**3b**), yellowish solid (90% yield); mp = 202–205 °C; IR (KBr): ν_max/cm_^−1^ = 1761, 1607, 1622, 1574; UV-Vis (MeOH): λ_max/nm_ = 295; ^1^H-NMR (500 MHz, CDCl_3_): δ 12.37 (s, 1H, H-3a), 8.91 (s, 1H, H-4), 7.43 (d, *J*= 9.1 Hz, 1H, H-8), 7.36 (dd, *J* = 9.1, 2.8 Hz, 1H, H-7), 7.12 (d, *J* = 2.8 Hz, 1H, H-5), 3.91 (s, 2H, H-6a); ^13^C-NMR (125 MHz, CDCl_3_): δ 164.22, 162.55, 157.25, 151.22, 149.22, 124.44, 118.90, 118.34, 115.00, 111.01, 56.05; HRMS (ESI) *m*/*z* calcd. for C_11_H_8_O_5_: [M + H]^+^ 221.0444, found 221.0446.

*6-bromo-2-oxo-2H-chromene-3-carboxylic acid* (**3c**), white solid (85% yield); mp = 195–197 °C; IR (KBr): ν_max/cm_^−1^ = 1737, 1716, 1690, 1609; UV-Vis (MeOH): λ_max/nm_ = 291; ^1^H-NMR (500 MHz, CDCl_3_): δ 12.06 (s, 1H, H-3a), 8.87 (s, 1H, H-4), 7.90 (d, *J* = 2.1 Hz, 1H, H-8), 7.87 (dd, *J* = 8.8, 2.2 Hz, 1H, H-7), 7.40 (d, *J* = 8.8 Hz, 1H, H-5); ^13^C-NMR (125 MHz, CDCl_3_): δ 163.41, 161.82, 153.33, 150.09, 138.42, 132.45, 119.82, 118.97, 116.01; HRMS (ESI) *m*/*z* calcd. for C_10_H_5_BrO_4_: [M + H]^+^ 268.9444, found 270.9428.

*8-ethoxy-2-oxo-2H-chromene-3-carboxylic acid* (**3d**), greenish solid (80% yield); mp = 186–188 °C; IR (KBr): ν_max/cm_^−1^ = 1756, 1674; UV-Vis (MeOH): λ_max/nm_ = 309; ^1^H-NMR (500 MHz, CDCl_3_): δ 12.32 (s, 1H, H-3a), 8.91 (s, 1H, H-4), 7.38 (t, *J* = 7.9 Hz, 1H, H-6), 7.30 (d, *J* = 7.9 Hz, 2H, H-5,7), 4.24 (q, *J* = 7.0 Hz, 2H, H-8a), 1.54 (t, *J* = 7.0 Hz, 3H, H-8b); ^13^C-NMR (125 MHz, CDCl_3_): δ 163.89, 162.53, 151.78, 146.81, 144.31, 126.16, 121.23, 119.19, 118.17, 114.90, 65.32, 14.64; HRMS (ESI) *m*/*z* calcd. for C_12_H_10_O_5_: [M + H]^+^ 235.0601, found 235.0604.

*7-(diethylamino)-2-oxo-2H-chromene-3-carboxylic acid* (**3e**), white solid (85% yield); mp = 195–197 °C; IR (KBr): ν_max/cm_^−1^ = 1737, 1716, 1690, 1609; UV-Vis (MeOH): λ_max/nm_= 291; ^1^H-NMR (500 MHz, CDCl_3_): δ 12.06 (s, 1H, H-3a), 8.87 (s, 1H, H-4), 7.90 (d, *J* = 2.1 Hz, 1H, H-8), 7.87 (dd, *J* = 8.8, 2.2 Hz, 1H, H-7), 7.40 (d, *J*= 8.8 Hz, 1H, H-5); ^13^C-NMR (125 MHz, CDCl_3_): δ 163.41, 161.82, 153.33, 150.09, 138.42, 132.45, 119.82, 118.97, 116.01; HRMS (ESI) *m*/*z* calcd. for C_10_H_5_BrO_4_: [M + H]^+^ 268.9444, found 270.9428.

*7-hydroxy-2-oxo-2H-chromene-3-carboxylic acid* (**3f**), brownish solid (90% yield); mp= 263–264 °C; IR (KBr): ν_max/cm_^−1^ = 3126, 1712, 1683, 1619; UV-Vis (MeOH): λ_max/nm_ = 351; ^1^H-NMR (500 MHz, DMSO-d_6_): δ 11.87 (s, 1H, H-7a), 8.64 (s, 1H, H-4), 7.70 (d, *J* = 8.6 Hz, 1H, H-5), 6.82 (dd, *J* = 8.6, 2.1 Hz, 1H, H-6), 6.70 (d, *J* = 2.0 Hz, 1H, H-8); ^13^C-NMR (125 MHz, DMSO-d_6_): δ 164.66, 164.39, 158.12, 157.42, 149.87, 132.44, 114.46, 112.83, 111.06, 102.24; HRMS (ESI) *m*/*z* calcd. for C_10_H_6_O_5_: [M + H]^+^ 207.0288, found 207.0291.

#### 3.1.2. General Procedure for the Preparation of Coumarin-3-Carboxamides (**5a**–**k**)

In a dry round-bottom flask purged with N_2_, the corresponding coumarin (1 mmol), 4-(dimethylamino)pyridine (DMAP) (5% mol), and carbonyldiimidazole (CDI) (1.1 mmol) were dissolved in DMF. After 30 min of stirring at room temperature, hydrochloride amine salt was added and stirring continued overnight. Water was added when the reaction was completed. The resulting precipitate was obtained by filtration and purified by recrystallization with MeOH and cold water.

*Methyl (2-oxo-2H-chromene-3-carbonyl)glycinate* (**5a**), white solid (84% yield); mp = 181–183 °C; IR (KBr): ν_max/cm_^−1^ = 3328, 3054, 1749, 1710, 1655; UV-Vis (MeOH): λ_max/nm_= 300; ^1^H-NMR (500 MHz, CDCl_3_): δ 9.25 (s, 1H, H-3a), 8.91 (s, 1H, H-4), 7.77–7.56 (m, 2H, H-5,7), 7.50–7.31 (m, 2H, H-6,8), 4.26 (d, *J* = 5.6 Hz, 2H, H-3b), 3.80 (s, 3H, H-3c); ^13^C-NMR (125 MHz, CDCl_3_): δ 169.73, 161.91, 161.24, 154.55, 148.77, 134.30, 129.90, 125.35, 118.52, 117.96, 116.72, 52.42, 41.69; HRMS (ESI) *m/z* calcd. for C_13_H_11_NO_5_: [M + H]^+^ 262.0710, found 262.0718.

*Ethyl 3-(2-oxo-2H-chromene-3-carboxamido)propanoate* (**5b**), white solid (60% yield); mp = 128–130 °C; IR (KBr): ν_max/cm_^−1^ = 3348, 3055, 1726, 1706, 1647, 1612; UV-Vis (MeOH): λ_max/nm_ = 297; ^1^H-NMR (500 MHz, CDCl_3_): δ 9.14 (s, 1H, H-3a), 8.90 (s, 1H, H-4), 7.81-7.56 (m, 2H, H-6,8), 7.39 (m, 2H, H-5,7), 4.20 (q, *J* = 7.1 Hz, 2H, H-3d), 3.76 (q, *J* = 6.3 Hz, 2H, H-3b), 2.66 (t, *J* = 6.4 Hz, 2H, H-3c), 1.29 (t, *J* = 7.1 Hz, 3H, H-3e); ^13^C-NMR (125 MHz, CDCl_3_): δ 171.77, 161.59, 161.26, 154.47, 148.36, 134.06, 129.80, 125.27, 118.61, 118.39, 116.65, 60.84, 35.44, 34.21, 14.20; HRMS (ESI) *m*/*z* calcd. for C_15_H_15_NO_5_: [M + H]^+^ 290.1023, found 290.1029.

*Methyl 6-(2-oxo-2H-chromene-3-carboxamido)hexanoate* (**5c**), white solid (85% yield); mp = 88–90 °C; IR (KBr): ν_max/cm_^−1^ = 3321, 3054, 1742, 1721, 1706, 1607; UV-Vis (MeOH): λ_max/nm_ = 298; ^1^H-NMR (500 MHz, CDCl_3_): δ 8.91 (s, 1H, H-4), 8.83 (s, 1H, H-3a), 7.73–7.63 (m, 2H, H-6,8), 7.39 (m, 2H, H-5,7), 3.67 (s, 3H, H-3g), 3.47 (dd, *J* = 13.1, 6.9 Hz, 2H, H-3b), 2.34 (t, *J* = 7.5 Hz, 2H, H-3f), 1.68 (tt, *J* = 14.8, 7.4 Hz, 4H, H-3c,3e), 1.49–1.36 (m, 2H, H-3d); ^13^C-NMR (125 MHz, CDCl_3_): δ 174.02, 161.47, 154.39, 148.26, 133.98, 129.79, 125.28, 118.59, 116.61, 51.51, 39.66, 33.91, 29.09, 26.52, 24.58; HRMS (ESI) *m*/*z* calcd. for C_17_H_19_NO_5_: [M + H]^+^ 318.1336, found 318.1345.

*Ethyl 4-(6-methoxy-2-oxo-2H-chromene-3-carboxamido)butanoate* (**5d**), yellowish solid (81% yield); mp = 131–132 °C; IR (KBr): ν_max/cm_^−1^ = 3325, 2981, 2948, 1734, 1698; UV-Vis (MeOH): λ_max/nm_= 297; ^1^H-NMR (500 MHz, CDCl_3_): δ 8.92 (s, 1H, H-3a), 8.86 (s, 1H, H-4), 7.34 (d, *J* = 9.1 Hz, 1H, H-8), 7.24 (dd, *J* = 9.1, 2.9 Hz, 1H, H-7), 7.08 (d, *J* = 2.9 Hz, 1H, H-5), 4.15 (q, *J* = 7.1 Hz, 2H, H-3e), 3.88 (s, 3H, H-6a), 3.51 (dd, *J* = 13.2, 6.8 Hz, 2H, H-3b), 2.41 (t, *J* = 7.4 Hz, 2H, H-3d), 1.98 (p, *J* = 7.2 Hz, 2H, H-3c), 1.26 (t, *J* = 7.1 Hz, 3H, H-3f); ^13^C-NMR (125 MHz, CDCl_3_): δ 172.95, 161.64, 156.61, 149.01, 148.14, 122.57, 118.98, 118.54, 117.73, 110.67, 60.48, 55.92, 39.12, 31.72, 24.79, 14.22; HRMS (ESI) *m*/*z* calcd. for C_17_H_19_NO_6_: [M + H]^+^ 334.1285, found 334.1304.

*Methyl 6-(6-methoxy-2-oxo-2H-chromene-3-carboxamido)hexanoate* (**5e**), pale yellowish solid (90% yield); mp = 101–102 °C; IR (KBr): ν_max/cm_^−1^ = 3310, 2939, 2859, 1745, 1717, 1697, 1658; UV-Vis (MeOH): λ_max/nm_ = 297; ^1^H-NMR (500 MHz, CDCl_3_): δ 8.88 (s, 1H, H-3a), 8.86(s, 1H, H-4), 7.33 (d, *J* = 9.1 Hz, 1H, H-8), 7.24 (dd, *J* = 9.1, 2.9 Hz, 1H, H-7), 7.08 (d, *J* = 2.9 Hz, 1H, H-5), 3.88 (s, 3H, H-6a), 3.67 (s, 3H, H-3g), 3.46 (dd, *J* = 13.1, 6.9 Hz, 2H, H-3b), 2.34 (t, *J* = 7.5 Hz, 2H, H-3f), 1.67 (tt, *J* = 15.1, 7.5 Hz, 4H, H-3c,3e), 1.52–1.36 (m, 2H, H-3d); ^13^C-NMR (125 MHz, CDCl_3_): δ 173.99, 161.55, 156.59, 148.97, 148.02, 122.49, 119.00, 118.63, 117.69, 110.65, 55.91, 51.50, 39.64, 33.90, 29.09, 26.52, 24.58; HRMS (ESI) *m*/*z* calcd. for C_18_H_21_NO_6_: [M + H]^+^ 348.1442, found 348.1480.

*Methyl 6-(8-ethoxy-2-oxo-2H-chromene-3-carboxamido)hexanoate* (**5f**), white solid (68% yield); mp= 98–99 °C; IR (KBr): ν_max/cm_^−1^ = 3343, 1718, 1655, 1613; UV-Vis (MeOH): λ_max/nm_ = 311; ^1^H-NMR (500 MHz, CDCl_3_): δ 8.88 (s, 1H, H-4), 8.86 (s, 1H, H-3a), 7.27 (d, *J* = 7.6 Hz, 1H, H-6), 7.25 (t, *J* = 3.9 Hz, 1H, H-7), 7.18 (dd, *J* = 7.7, 1.4 Hz, 1H, H-5), 4.22 (q, *J* = 7.0 Hz, 2H, H-8a), 3.67 (s, 3H, H-3g), 3.46 (dd, *J* = 13.2, 6.9 Hz, 2H, H-3b), 2.34 (t, *J* = 7.5 Hz, 2H, H-3f), 1.67 (tt, *J* = 14.7, 7.5 Hz, 4H, H-3c, 3e), 1.52 (t, *J* = 7.0 Hz, 3H, H-8b), 1.47–1.39 (m, 2H, H-3d); ^13^C-NMR (125 MHz, CDCl_3_): δ 173.96, 161.52, 161.17, 148.46, 146.41, 144.31, 125.07, 120.84, 119.44, 118.62, 116.73, 65.16, 51.45, 39.65, 33.91, 29.09, 26.52, 24.59, 14.68; HRMS (ESI) *m*/*z* calcd. for C_19_H_23_NO_6_: [M + H]^+^ 362.1598, found 362.1620.

*Methyl (6-bromo-2-oxo-2H-chromene-3-carbonyl)glycinate* (**5g**), white solid (46% yield); mp = 160–162 °C; IR (KBr): ν_max/cm_^−1^ = 3337, 3048, 1740, 1724, 1655; UV-Vis (MeOH): λ_max/nm_= 290; ^1^H-NMR (500 MHz, CDCl_3_): δ 9.18 (s, 1H, H-3a), 8.82 (s, 1H, H-4), 7.83 (d, *J* = 2.3 Hz, 1H, H-5), 7.76 (dd, *J* = 8.8, 2.3 Hz, 1H, H-7), 7.31 (d, *J* = 8.8 Hz, 1H, H-8), 4.25 (d, *J* = 5.6 Hz, 2H, H-3b), 3.80 (s, 3H, H-3c); ^13^C-NMR (125 MHz, CDCl_3_): δ 169.63, 161.39, 160.59, 153.32, 147.40, 137.00, 131.92, 119.96, 119.00, 118.45, 118.01, 52.48, 41.73; HRMS (ESI) *m*/*z* calcd. for C_13_H_10_NO_5_: [M + H]^+^ 339.9742, found 339.9802.

*Ethyl 3-(6-bromo-2-oxo-2H-chromene-3-carboxamido)propanoate* (**5h**), white solid (58% yield); mp = 148–149 °C; IR (KBr): ν_max/cm_^−1^ = 3383, 3053, 1734, 1717, 1657; UV-Vis (MeOH): λ_max/nm_ = 292; ^1^H-NMR (500 MHz, CDCl_3_): δ 9.09 (s, 1H, H-3a), 8.82 (s, 1H, H-4), 7.83 (d, *J* = 2.0 Hz, 1H, H-5), 7.74 (dd, *J* = 8.8, 2.1 Hz, 1H, H-7), 7.29 (d, *J* = 8.8 Hz, 1H, H-8), 4.20 (q, *J* = 7.1 Hz, 2H, H-3d), 3.75 (q, *J* = 6.2 Hz, 2H, H-3b), 2.66 (t, *J* = 6.3 Hz, 2H, H-3c), 1.29 (t, *J* = 7.1 Hz, 3H, H-3e); ^13^C-NMR (125 MHz, CDCl_3_): δ 171.76, 161.06, 160.58, 153.22, 147.00, 136.74, 131.84, 120.07, 119.43, 118.36, 117.90, 60.87, 35.49, 34.11, 14.19; HRMS (ESI) *m*/*z* calcd. for C_15_H_14_BrNO_5_: [M + H]^+^ 368.0128, found 368.0132.

*Methyl 6-(6-bromo-2-oxo-2H-chromene-3-carboxamido)hexanoate* (**5i**), white solid (77% yield); mp = 147–149 °C; IR(KBr): ν_max/cm_^−1^ = 3336, 3051, 1734, 1715, 1658; UV-Vis (MeOH) λ_max/nm_ = 292; ^1^H-NMR (500 MHz, CDCl_3_): δ 8.84 (s, 1H, H-4), 8.75 (s, 1H, H-3a), 7.84 (d, *J* = 2.2 Hz, 1H, H-5), 7.74 (dd, *J* = 8.8, 2.3 Hz, 1H, H-7), 7.30 (d, *J* = 8.8 Hz, 1H, H-8), 3.67 (s, 3H, H-3g), 3.46 (dd, *J* = 13.1, 7.0 Hz, 2H, H-3b), 2.34 (t, *J* = 7.5 Hz, 2H, H-3f), 1.67 (tt, *J* = 15.0, 7.5 Hz, 4H, H-3c,3e), 1.50–1.37 (m, 2H, H-3d); ^13^C-NMR (125 MHz, CDCl_3_): δ 173.98, 160.91, 160.84, 153.16, 146.89, 136.67, 131.83, 120.14, 119.56, 118.33, 117.92, 51.52, 39.75, 33.89, 29.05, 26.50, 24.57; HRMS (ESI) *m*/*z* calcd. for C_17_H_18_BrNO_5_: [M + H]^+^ 396.0441, found 396.0448.

*Ethyl 3-(7-(diethylamino)-2-oxo-2H-chromene-3-carboxamido)propanoate* (**5j**), yellow solid (40% yield); mp = 134–136 °C; IR (KBr): ν_max/cm_^−1^ = 3444, 3320, 2940, 1739, 1702; UV-Vis (MeOH): λ_max/nm_ = 419; ^1^H-NMR (500 MHz, CDCl_3_): δ 9.08 (s, 1H, H-3a), 8.69 (s, 1H, H-4), 7.42 (d, *J* = 9.0 Hz, 1H, H-5), 6.64 (dd, *J* = 9.0, 2.1 Hz, 1H, H-6), 6.49 (d, *J* = 1.8 Hz, 1H, H-8), 4.19 (q, *J* = 7.1 Hz, 4H, H-7a), 3.73 (q, *J* = 6.3 Hz, 2H, H-3d), 3.45 (q, 2H, H-3b), 2.64 (t, *J* = 6.5 Hz, 2H, H-3c), 1.29 (t, *J* = 7.2 Hz, 3H, H-3e), 1.24 (t, *J* = 7.2 Hz, 6H, H-7b); ^13^C-NMR (125 MHz, CDCl_3_): δ 171.94, 163.25, 162.61, 157.67, 152.55, 148.09, 131.13, 110.21, 109.91, 108.35, 96.59, 60.72, 45.07, 35.25, 34.49, 14.20, 12.42. HRMS (ESI) *m*/*z* calcd. for C_19_H_24_N_2_O_5_: [M + H]^+^ 361.1758, found 361.1780. 

*Methyl 6-(7-(diethylamino)-2-oxo-2H-chromene-3-carboxamido)hexanoate* (**5k**), yellow solid (61% yield); mp = 90–91 °C; IR (KBr): ν_max/cm_^−1^ = 3367, 2978, 1735, 1688, 1650; UV-Vis (MeOH): λ_max/nm_ = 418; ^1^H-NMR (500 MHz, CDCl_3_): δ 8.80 (s, 1H, H-3a), 8.70 (s, 1H, H-4), 7.43 (d, *J* = 8.9 Hz, 1H, H-5), 6.64 (dd, *J* = 8.9, 2.3 Hz, 1H, H-6), 6.50 (d, *J* = 2.0 Hz, 1H, H-8), 3.67 (s, 3H, H-3g), 3.49–3.38 (m, 6H, H-7a,3b), 2.33 (t, *J* = 7.5 Hz, 2H, H-3f), 1.66 (qd, *J* = 15.0, 7.4 Hz, 4H, H-3c,3e), 1.51–1.36 (m, 2H, H-3d), 1.24 (t, *J* = 7.1 Hz, 6H, H-7b); ^13^C-NMR (125 MHz, CDCl_3_): δ 174.08, 163.08, 162.80, 157.60, 152.47, 148.01, 131.08, 110.43, 109.91, 108.40, 96.56, 51.49, 45.06, 39.42, 33.97, 29.28, 26.58, 24.66. HRMS (ESI) *m*/*z* calcd. for C_21_H_28_N_2_O_5_: [M + H]^+^ 389.2071, found 389.2102.

#### 3.1.3. General Procedure for the Preparation of *N*-(2-Oxo-2*H*-Chromene-3-Carboxamide) Acids (**6a**–**k**)

In a round-bottom flask, the corresponding ester (1 mmol) was added and dissolved in 10 mL of THF:H_2_O (1:2). The mixture was stirred at room temperature before adding LiOH (7 mmol) and stirring was continued overnight. Upon completion of the reaction, HCl was slowly added until a precipitate appeared, which was filtered and purified by recrystallization with MeOH and cold water to afford the desired product. Diethylamine derivatives were synthesized by using the Mravljak, J. et al. methodology [59].

*(2-oxo-2H-chromene-3-carbonyl)glycine* (**6a**), white solid (93% yield); mp = 246–247 °C; IR (KBr): ν_max/cm_^−1^ = 3315, 2967, 2686, 1759, 1712, 1638; UV-Vis (MeOH): λ_max/nm_ = 297; ^1^H-NMR (400 MHz, DMSO-*d*_6_): δ 12.78 (s, 1H, H-3c), 8.99 (s, 1H, H-3a), 8.86 (s, 1H, H-4), 7.95 (d, *J* = 7.4 Hz, 1H, H-8), 7.71 (t, *J* = 7.5 Hz, 1H, H-7), 7.46 (d, *J* = 8.1 Hz, 1H, H-5), 7.39 (t, *J* = 7.0 Hz, 1H, H-6), 4.00 (s, 2H, H-3b); ^13^C-NMR (100 MHz, DMSO-*d*_6_): δ 171.27, 161.63, 160.84, 154.46, 148.58, 134.77, 130.89, 125.64, 118.89, 118.66, 116.64, 41.97; HRMS (ESI) *m*/*z* calcd. for C_12_H_9_NO_5_: [M + H]^+^ 248.0553, found 248.0561.

*3-(2-oxo-2H-chromene-3-carboxamido)propanoic acid* (**6b**), white solid (70% yield); mp = 199–200 °C; IR (KBr): ν_max/cm_^−1^ = 3323, 3068, 1725, 1706, 1635; UV-Vis (MeOH): λ_max/nm_ = 297; ^1^H-NMR (400 MHz, DMSO-d_6_): δ 12.34 (s, 1H, H-3d), 8.85 (s, 1H, H-3a), 8.82 (s, 1H, H-4), 7.93 (d, *J* = 7.4 Hz, 1H, H-8), 7.69 (t, *J* = 7.4 Hz, 1H, H-7), 7.44 (d, *J* = 8.2 Hz, 1H, H-5), 7.38 (t, *J* = 7.1 Hz, 1H, H-4), 3.47 (d, *J* = 5.0 Hz, 2H, H-3b), 2.46 (d, *J* = 7.7 Hz, 2H, H-3c); ^13^C-NMR (100 MHz, DMSO-d_6_): δ 173.58, 161.46, 160.86, 154.35, 148.14, 134.58, 130.76, 125.59, 119.08, 118.92, 116.59, 35.55, 34.13; HRMS (ESI) *m*/*z* calcd. for C_13_H_11_NO_5_: [M + H]^+^ 262.0710, found 262.0719.

*6-(2-oxo-2H-chromene-3-carboxamido)hexanoic acid* (**6c**), white solid (75% yield); mp = 145–146 °C; IR (KBr): ν_max/cm_^−1^ = 3357, 1710, 1694, 1527; UV-Vis (MeOH): λ_max/nm_ = 299; ^1^H-NMR (500 MHz, DMSO-*d*_6_): δ 12.01 (s, 1H, H-3g), 8.85 (s, 1H, H-4), 8.69 (t, *J* = 5.6 Hz, 1H, H-3a), 7.99 (d, *J* = 7.4 Hz, 1H, H-8), 7.88–7.65 (m, 1H, H-7), 7.51 (d, *J* = 8.3 Hz, 1H, H-5), 7.44 (t, *J* = 7.4 Hz, 1H, H-6), 3.39–3.29 (m, 2H, H-3b), 2.22 (t, *J* = 7.3 Hz, 2H, H-3f), 1.62–1.47 (m, 4H, H-3c,3e), 1.36–1.27 (m, 2H, H-3d); ^13^C-NMR (125 MHz, DMSO-*d*_6_): δ 174.86, 161.48, 160.85, 154.31, 147.72, 134.45, 130.67, 125.57, 119.62, 118.96, 116.58, 39.42, 34.04, 29.15, 26.43, 24.64; HRMS (ESI) *m*/*z* calcd. for C_16_H_17_NO_5_: [M + H]^+^ 304.1179, found 304.1184.

*4-(6-methoxy-2-oxo-2H-chromene-3-carboxamido)butanoic acid* (**6d**), light yellow solid (81% yield); mp = 200–203 °C; IR (KBr): ν_max/cm_^−1^ = 3311, 1700, 1531; UV-Vis (MeOH) λ_max/nm_ = 298; ^1^H-NMR (500 MHz, DMSO-*d*_6_): δ 12.12 (s, 1H, H-3e), 8.78 (s, 1H, H-4), 8.74 (t, *J* = 5.8 Hz, 1H, H-3a), 7.52 (d, *J* = 2.9 Hz, 1H, H-5), 7.43 (d, *J* = 9.1 Hz, 1H, H-8), 7.32 (dd, *J* = 9.1, 3.0 Hz, 1H, H-7), 3.82 (s, 3H, H-6a), 3.36 (dd, *J* = 13.1, 6.7 Hz, 2H, H-3b), 2.30 (t, *J* = 7.4 Hz, 2H, H-3d), 1.78 (p, *J* = 7.2 Hz, 2H, H-3c); ^13^C-NMR (125 MHz, DMSO-d_6_): δ 174.54, 161.66, 160.88, 156.40, 148.78, 147.54, 122.29, 119.64, 119.35, 117.65, 112.24, 56.27, 39.00, 31.59, 24.99; HRMS (ESI) *m*/*z* calcd. for C_15_H_15_NO_6_: [M + H]^+^ 306.0972, found 306.0974.

*6-(6-methoxy-2-oxo-2H-chromene-3-carboxamido)hexanoic acid* (**6e**), light yellow solid (81% yield); mp = 142–144 °C; IR (KBr): ν_max/cm_^−1^ = 3322, 1721, 1702, 1575; UV-Vis (MeOH): λ_max/nm_ = 298; ^1^H-NMR (500 MHz, DMSO-d_6_): δ 8.79 (s, 1H, H-4), 8.72 (s, 1H, H-3a), 7.52 (s, 1H, H-5), 7.42 (d, *J* = 9.2 Hz, 1H, H-8), 7.38-7.27 (m, 1H, H-7), 3.82 (s, 3H, H-6a), 3.32 (dd, *J* = 13.0, 6.7 Hz, 2H, H-3b), 2.23 (t, *J* = 7.3 Hz, 2H, H-3f), 1.61–1.49 (m, 4H, H-3c,3e), 1.40–1.30 (m, 2H, H-3d); ^13^C-NMR (125 MHz, DMSO-*d*_6_): δ 174.85, 161.45, 160.98, 156.41, 148.77, 147.61, 122.32, 119.52, 119.36, 117.66, 112.23, 56.26, 39.42, 34.04, 29.14, 26.44, 24.64; HRMS (ESI) *m*/*z* calcd. for C_17_H_19_NO_6_: [M + H]^+^ 334.1285, found 334.1288.

*6-(8-ethoxy-2-oxo-2H-chromene-3-carboxamido)hexanoic acid* (**6f**), white solid (83% yield); mp = 169–170 °C; IR (KBr): ν_max/cm_^−1^ = 3337, 1716, 1605, 1542; UV-Vis (MeOH): λ_max/nm_ = 312; ^1^H-NMR (500 MHz, DMSO-d_6_): δ 12.02 (s, 1H, H-3g), 8.80 (s, 1H, H-4), 8.69 (t, *J* = 5.7 Hz, 1H, H-3a), 7.49 (d, *J* = 7.1 Hz, 1H, H-7), 7.40 (d, *J* = 7.6 Hz, 1H, H-5), 7.34 (t, *J* = 7.9 Hz, 1H, H-6), 4.20 (q, *J* = 6.9 Hz, 2H, H-8a), 3.32 (dd, *J* = 13.2, 6.7 Hz, 2H, H-3b), 2.22 (t, *J* = 7.3 Hz, 2H, H-3f), 1.59–1.49 (m, 4H, H-3c,3e), 1.42 (t, *J* = 7.0 Hz, 3H, H-8b), 1.38–1.29 (m, 2H, H-3d); ^13^C-NMR (125 MHz, DMSO-d_6_): δ 174.84, 161.45, 160.63, 147.96, 145.97, 143.74, 125.49, 121.55, 119.60, 117.31, 65.03, 39.43, 34.05, 29.15, 26.44, 24.64, 15.01; HRMS (ESI) *m*/*z* calcd. for C_18_H_21_NO_6_: [M + H]^+^ 348.1442, found 348.1452.

*(6-bromo-2-oxo-2H-chromene-3-carbonyl)glycine* (**6g**), white solid (83% yield); mp = 269–270 °C; IR (KBr): ν_max/cm_^−1^ = 3341, 1726, 1648, 1560; UV-Vis (MeOH) λ_max/nm_ = 291; ^1^H-NMR (500 MHz, DMSO-d_6_): δ 12.84 (s, 1H, H-3c), 9.02 (t, *J* = 5.5 Hz, 1H, H-3a), 8.87 (s, 1H, H-4), 8.27 (d, *J* = 2.3 Hz, 1H, H-5), 7.90 (dd, *J* = 8.8, 2.3 Hz, 1H, H-7), 7.49 (d, *J* = 8.9 Hz, 1H, H-8), 4.07 (d, *J* = 5.5 Hz, 2H, H-3b). ^13^C-NMR (125 MHz, DMSO-d_6_): δ 171.16, 161.32, 160.33, 153.48, 147.25, 136.87, 132.70, 120.75, 119.79, 118.89, 117.14, 42.00. HRMS (ESI) *m*/*z* calcd. for C_12_H_8_NO_5_: [M + H]^+^ 325.9659, found 325.9668.

*3-(6-bromo-2-oxo-2H-chromene-3-carboxamido)propanoic acid* (**6h**), white solid (50% yield); mp = 251–252 °C; IR (KBr): ν_max/cm_^−1^ = 3380, 1705, 1657, 1533; UV-Vis (MeOH): λ_max/nm_ = 292; ^1^H-NMR (500 MHz, DMSO-d_6_): δ 12.39 (s, 1H, H-3d), 8.88 (t, *J* = 5.8 Hz, 1H, H-3a), 8.84 (s, 1H, H-4), 8.25 (d, *J* = 2.2 Hz, 1H, H-5), 7.88 (dd, *J* = 8.8, 2.3 Hz, 1H, H-7), 7.47 (d, *J* = 8.9 Hz, 1H, H-8), 3.54 (q, *J* = 6.4 Hz, 2H, H-3b), 2.54 (t, *J* = 6.7 Hz, 2H, H-3c); ^13^C-NMR (125 MHz, DMSO-*d*_6_): δ 173.50, 161.16, 160.35, 153.38, 146.79, 136.70, 132.59, 120.80, 120.25, 118.85, 117.10, 35.62, 34.11; HRMS (ESI) *m*/*z* calcd. for C_13_H_10_NO_5_: [M + H]^+^ 339.9815, found 339.9817.

*6-(6-bromo-2-oxo-2H-chromene-3-carboxamido)hexanoic acid* (**6i**), white solid (93% yield); mp = 182–183 °C; IR (KBr): ν_max/cm_^−1^ = 3347, 1715, 1657, 1562; UV-Vis (MeOH): λ_max/nm_= 293; ^1^H-NMR (500 MHz, DMSO-d_6_): δ 12.01 (s, 1H, H-3g), 8.80 (s, 1H, H-4), 8.66 (t, *J* = 5.7 Hz, 1H, H-3a), 8.25 (d, *J* = 2.2 Hz, 1H, H-5), 7.88 (dd, *J* = 8.8, 2.3 Hz, 1H, H-7), 7.47 (d, *J* = 8.8 Hz, 1H, H-8), 3.31 (dd, *J* = 13.1, 6.7 Hz, 2H, H-3b), 2.22 (t, *J* = 7.4 Hz, 2H, H-3f), 1.59–1.47 (m, 4H, H-3c,3e), 1.37–1.28 (m, 2H, H-3d); ^13^C-NMR (125 MHz, DMSO-*d*_6_): δ 174.84, 161.18, 160.32, 153.33, 146.37, 136.59, 132.51, 120.83, 120.78, 118.85, 117.08, 39.47, 34.05, 29.11, 26.42, 24.64; HRMS (ESI) *m*/*z* calcd. for C_16_H_16_NO_5_: [M + H]^+^ 382.0212, found 382.0289.

*3-(7-(diethylamino)-2-oxo-2H-chromene-3-carboxamido)propanoic acid* (**6j**), yellow crystals (75% yield); mp = 202–205 °C; ^1^H-NMR (DMSO-*d_6_*, 500 MHz): δ 8.84 (t, *J* = 5.1 Hz, 1H), 8.65 (s, 1H), 7.66 (d, *J* = 8.9 Hz, 1H), 6.78 (d, *J* = 8.6 Hz, 1H), 6.59 (s, 1H), 3.54–3.50 (m, 2H), 3.47 (dd, *J* = 13.7, 6.8 Hz, 4H), 2.49 (t, *J* = 6.2 Hz, 2H), 1.14 (t, *J* = 6.9 Hz, 6H).

*6-(7-(diethylamino)-2-oxo-2H-chromene-3-carboxamido)hexanoic acid* (**6k**), yellow crystals (73% yield); mp = 150–152 °C; ^1^H-NMR (DMSO-*d_6_*, 500 MHz): δ 8.67–8.62 (m, 2H), 7.68 (d, *J* = 9.0 Hz, 1H), 6.80 (dd, *J* = 9.0, 2.2 Hz, 1H), 6.62 (d, *J* = 2.0 Hz, 1H), 3.48 (dd, *J* = 14.0, 7.0 Hz, 4H), 3.31–3.27 (m, 2H), 2.21 (t, *J* = 7.3 Hz, 2H), 1.51 (dq, *J* = 14.8, 7.4 Hz, 4H), 1.31 (dd, *J* = 15.2, 8.1 Hz, 2H), 1.14 (t, *J* = 7.0 Hz, 6H).

#### 3.1.4. General Procedure for the Preparation of the Title Compounds (**7a**–**k**) 

In a dry round-bottom flask purged with N_2_, the corresponding carboxylic acid (1 mmol), DMAP (5% mol) and CDI (1.1 mmol) were dissolved in DMF. After 30 min of stirring at room temperature, hydroxylamine hydrochloride was added and stirring continued overnight. Upon completion of the reaction, sodium bicarbonate was added. The resulting precipitate was obtained by filtration and purified by recrystallization with MeOH and cold water.

*N-(2-(hydroxyamino)-2-oxoethyl)-2-oxo-2H-chromene-3-carboxamide* (**7a**), white solid (50% yield); mp = 226–227 °C; IR (KBr): ν_max/cm_^−1^ = 3304, 3240, 3043, 1709, 1668; UV-Vis (MeOH): λ_max/nm_ = 297; Em (MeOH): λ_max/nm_ = 406; ^1^H-NMR (400 MHz, DMSO-*d*_6_): δ 10.68 (s, 1H), 9.04 (s, 1H), 8.90 (s, 1H), 8.00 (s, 1H), 7.76 (s, 1H), 7.61–7.35 (m, 2H), 3.92 (s, 2H); ^13^C-NMR (100 MHz, DMSO-d_6_): δ 165.66, 161.61, 160.84, 154.38, 148.16, 134.71, 130.84, 125.64, 118.92, 118.88, 116.62, 41.09; HRMS (ESI) *m*/*z* calcd. for C_12_H_10_N_2_O_5_: [M + Na]^+^ 285.0488, found 285.0505.

*N-(3-(hydroxyamino)-3-oxopropyl)-2-oxo-2H-chromene-3-carboxamide* (**7b**), white solid (45% yield); mp = 170–173 °C; IR (KBr): ν_max/cm_^−1^ = 3469, 3302, 3194, 1708, 1648; UV-Vis (MeOH) λ_max/nm_ = 303.12; Em (MeOH): λ_max/nm_ = 405; ^1^H-NMR (500 MHz, DMSO-*d*_6_): δ 8.88 (s, 1H), 8.87 (s,1H), 7.99 (d, *J* = 6.6 Hz, 1H), 7.76 (dd, *J* = 11.5, 4.2 Hz, 1H), 7.51 (d, *J* = 8.3 Hz, 1H), 7.45 (t, *J* = 7.2 Hz, 1H), 3.54 (dd, *J* = 12.7, 6.5 Hz, 2H), 2.29 (t, *J* = 6.7 Hz, 2H); ^13^C-NMR (125 MHz, DMSO-d_6_): δ 167.78, 161.45, 160.78, 154.36, 148.09, 134.59, 130.76, 125.60, 119.11, 118.91, 116.60, 116.56, 36.12, 32.39. HRMS (ESI) *m/z* calcd. for C_13_H_12_N_2_O_5_: [M + H]^+^ 277.0819, found 277.0860. 

*N-(6-(hydroxyamino)-6-oxohexyl)-2-oxo-2H-chromene-3-carboxamide* (**7c**), white solid (50% yield); mp = 110–112 °C; IR (KBr): ν_max/cm_^−1^ = 3336, 3207, 1706, 1537; UV-Vis (MeOH): λ_max/nm_ = 329.96; Em (MeOH): λ_max/nm_ = 405; ^1^H-NMR (500 MHz, DMSO-d_6_): δ 8.84 (s, 1H), 8.68 (s, 1H), 7.97 (d, *J* = 6.3 Hz, 1H), 7.74 (s, 1H), 7.50 (d, *J* = 7.4 Hz, 1H), 7.44 (s, 2H), 3.31 (d, *J* = 3.4 Hz, 3H), 1.96 (s, 2H), 1.53 (s, 5H), 1.29 (s, 2H); ^13^C-NMR (125 MHz, DMSO-*d*_6_): δ 169.47, 161.48, 160.85, 154.30, 147.71, 134.46, 130.66, 125.58, 119.59, 118.94, 116.57, 39.49, 32.66, 29.17, 26.50, 25.31; HRMS (ESI) *m*/*z* calcd. for C_16_H_18_N_2_O_5_: [M + Na]^+^ 341.1114, found 341.1019.

*N-(4-(hydroxyamino)-4-oxobutyl)-6-methoxy-2-oxo-2H-chromene-3-carboxamide* (**7d**), light green solid (30% yield); mp = 154–156 °C; IR (KBr): ν_max/cm_^−1^ = 3531, 3241, 1706, 1554; UV-Vis (MeOH): λ_max/nm_ = 363; Em (MeOH): λ_max/nm_ = 458; ^1^H-NMR (500 MHz, DMSO-*d*_6_): δ 8.80 (s, 1H), 8.75 (s, 1H), 7.54 (d, *J* = 2.2 Hz, 1H), 7.44 (d, *J* = 9.1 Hz, 1H), 7.32 (dd, *J* = 9.1, 2.5 Hz, 1H), 3.82 (s, 3H), 3.32 (dd, *J* = 13.1, 6.6 Hz, 2H), 2.03 (t, *J* = 7.5 Hz, 2H), 1.82–1.72 (m, 2H); ^13^C-NMR (125 MHz, DMSO-d_6_): δ 169.09, 161.64, 160.89, 156.40, 148.79, 147.62, 122.35, 119.63, 119.37, 117.70, 112.25, 56.28, 39.22, 30.32, 25.72; HRMS (ESI) *m*/*z* calcd. for C_15_H_16_N_2_O_6_: [M + H]^+^ 321.1081, found 321.1132.

*N-(6-(hydroxyamino)-6-oxohexyl)-6-methoxy-2-oxo-2H-chromene-3-carboxamide* (**7e**), light green solid (87% yield); mp = 75–77 °C; IR (KBr): ν_max/cm_^−1^ = 3318, 2935, 1702, 1575; UV-Vis (MeOH): λ_max/nm_ = 299; Em (MeOH): λ_max/nm_ = 458; ^1^H-NMR (500 MHz, DMSO-*d*_6_): δ 10.36 (s, 1H), 8.81 (s, 1H), 8.72 (s, 1H), 7.54 (s, 1H), 7.44 (d, *J* = 9.0 Hz, 1H), 7.33 (d, *J* = 8.9 Hz, 1H), 3.82 (s, 3H), 3.31 (s, 2H), 2.21 (t, *J* = 7.2 Hz, 1H), 1.97 (d, *J* = 7.2 Hz, 1H), 1.53 (d, *J* = 6.3 Hz, 4H), 1.37–1.27 (m, 2H); ^13^C-NMR (125 MHz, DMSO-d_6_): δ 169.52, 161.48, 161.01, 156.43, 148.80, 147.65, 122.36, 119.60, 119.40, 117.70, 112.28, 56.30, 39.48, 32.65, 29.18, 26.50, 25.30; HRMS (ESI) *m*/*z* calcd. for C_18_H_20_N_2_O_5_: [M + Na]^+^ 371.1219 found 371.1194.

*8-ethoxy-N-(6-(hydroxyamino)-6-oxohexyl)-2-oxo-2H-chromene-3-carboxamide* (**7f**), light green solid (48% yield); mp = 167–169 °C; IR (KBr): ν_max/cm_^−1^ = 3335, 1933, 1716, 1539; UV-Vis (MeOH): λ_max/nm_= 311; Em (MeOH) λ_max/nm_= 486; ^1^H-NMR (500 MHz, DMSO-d_6_): δ 8.81 (s, 3H), 8.69 (s, 3H), 7.50 (d, *J* = 7.6 Hz, 3H), 7.41 (d, *J* = 8.0 Hz, 3H), 7.34 (t, *J* = 7.9 Hz, 3H), 4.20 (q, *J* = 6.8 Hz, 6H), 1.53 (s, 12H), 1.42 (t, *J* = 6.9 Hz, 11H), 1.32 (dd, *J* = 17.8, 7.6 Hz, 7H); ^13^C-NMR (125 MHz, DMSO-d_6_): δ 169.51, 161.47, 160.65, 147.99, 145.97, 143.72, 125.53, 121.55, 119.61, 117.32, 65.02, 34.06, 32.64, 29.19, 26.50, 25.30, 15.02; HRMS (ESI) *m*/*z* calcd. for C_18_H_22_N_2_O_6_: [M + H]^+^ 362.3820, found 362.3843.

*6-bromo-N-(2-(hydroxyamino)-2-oxoethyl)-2-oxo-2H-chromene-3-carboxamide* (**7g**), white solid (46% yield); mp = 202–205 °C; IR (KBr) ν_max/cm_^−1^ = 3417, 3272, 1712, 1537; UV-Vis (MeOH): λ_max/nm_ = 294; Em (MeOH): λ_max/nm_ = 424; ^1^H-NMR (500 MHz, DMSO-d_6_): δ 9.01 (d, *J* = 4.6 Hz, 1H), 8.86 (s, 1H), 8.28 (s, 1H), 7.90 (d, *J* = 8.8 Hz, 1H), 7.50 (d, *J* = 8.9 Hz, 1H), 3.92 (d, *J* = 5.2 Hz, 2H); ^13^C-NMR (125 MHz, DMSO-d_6_): δ 165.55, 161.34, 160.34, 153.43, 146.83, 136.83, 132.67, 120.78, 120.13, 118.91, 117.14, 41.13; HRMS (ESI) *m*/*z* calcd. for C_12_H_9_BrN_2_O_5_: [M + H]^+^ 339.9695, found 339.9711.

*6-bromo-N-(3-(hydroxyamino)-3-oxopropyl)-2-oxo-2H-chromene-3-carboxamide* (**7h**), white solid (45% yield); mp = 185–187 °C; IR (KBr): ν_max/cm_^−1^ = 3354, 3040, 2854, 1708, 1538; UV-Vis (MeOH): λ_max/nm_ = 295; Em (MeOH): λ_max/nm_ = 415; ^1^H-NMR (500 MHz, DMSO-d_6_): δ 8.86 (d, *J* = 5.4 Hz, 1H), 8.83 (s, 1H), 8.26 (s, 2H), 7.89 (dd, *J* = 8.8, 1.8 Hz, 1H), 7.48 (d, *J* = 8.8 Hz, 1H), 3.53 (dd, *J* = 12.6, 6.4 Hz, 3H), 2.28 (t, *J* = 6.7 Hz, 2H; ^13^C-NMR (125 MHz, DMSO-d_6_): δ 161.16, 160.30, 153.40, 146.78, 146.58, 136.72, 136.54, 132.57, 120.82, 118.86, 117.06, 36.19, 32.31; MS (ESI) *m*/*z* calcd. for C_13_H_11_BrN_2_O_6_: [M + H]^+^ 356.1440, found 356.1252.

*6-bromo-N-(6-(hydroxyamino)-6-oxohexyl)-2-oxo-2H-chromene-3-carboxamide* (**7i**), white solid (65% yield); mp = 202–205 °C; IR (KBr): ν_max/cm_^−1^ = 3344, 2934, 1724, 1559; UV-Vis (MeOH): λ_max/nm_ = 283; Em (MeOH): λ_max/nm_ = 424; ^1^H-NMR (500 MHz, DMSO-d_6_): δ 8.80 (d, *J* = 4.2 Hz, 2H), 8.67 (t, *J* = 5.7 Hz, 2H), 8.25 (dd, *J* = 6.4, 2.2 Hz, 2H), 7.88 (dd, *J* = 8.8, 2.3 Hz, 2H), 7.47 (dd, *J* = 8.6, 5.5 Hz, 2H), 3.30 (dd, *J* = 12.6, 6.4 Hz, 5H), 1.96 (t, *J* = 7.3 Hz, 4H), 1.52 (dt, *J* = 14.9, 7.4 Hz, 8H), 1.29 (dd, *J* = 14.7, 7.7 Hz, 4H); ^13^C-NMR (125 MHz, DMSO-d_6_): δ 169.41, 162.78, 161.19, 160.36, 153.32, 146.40, 136.61, 132.51, 120.79, 118.87, 117.09, 39.52, 36.25, 29.16, 26.48, 25.32; HRMS (ESI) *m/z* calcd. for C_16_H_17_BrN_2_O_5_: [M + H]^+^ 397.0321, found 397.1750.

*7-(diethylamino)-N-(3-(hydroxyamino)-3-oxopropyl)-2-oxo-2H-chromene-3-carboxamide* (**7j**), yellow solid (82% yield); mp = 126–128 °C; IR (KBr): ν_max/cm_^−1^ = 3231, 1699, 1616; UV-Vis (MeOH): λ_max/nm_ = 419; Em (MeOH): λ_max/nm_ = 468; ^1^H-NMR (500 MHz, DMSO-d_6_): δ 8.78 (t, *J* = 5.7 Hz, 1H), 8.65 (s, 2H), 7.67 (d, *J* = 9.0 Hz, 1H), 6.78 (d, *J* = 9.0 Hz, 1H), 6.60 (d, *J* = 1.3 Hz, 1H), 3.47 (q, *J* = 6.8 Hz, 4H), 2.25 (t, *J* = 6.6 Hz, 2H), 2.01 (t, *J* = 7.5 Hz, 2H), 1.13 (t, *J* = 6.9 Hz, 6H); ^13^C-NMR (125 MHz, DMSO-d_6_): δ 169.13, 167.85, 162.60, 162.05, 157.70, 152.87, 148.16, 132.02, 110.57, 108.12, 108.10, 96.34, 44.79, 12.77; HRMS (ESI) *m*/*z* calcd. for C_17_H_21_N_3_O_5_: [M + H]^+^ 348.1554, found 348.1570. 

*7-(diethylamino)-N-(6-(hydroxyamino)-6-oxohexyl)-2-oxo-2H-chromene-3-carboxamide* (**7k**), yellow solid (82% yield); mp = 173–174 °C; IR (KBr): ν_max/cm_^−1^ = 3122, 1700, 1618; UV-Vis (MeOH): λ_max/nm_ = 418; Em (MeOH): λ_max/nm_ = 470; ^1^H-NMR (500 MHz, DMSO-d_6_): δ 8.78 (t, *J* = 5.7 Hz, 1H), 8.65 (s, 2H), 7.67 (d, *J* = 9.0 Hz, 1H), 6.78 (d, *J* = 9.0 Hz, 1H), 6.60 (d, *J* = 1.3 Hz, 1H), 3.47 (q, *J* = 6.8 Hz, 4H), 2.25 (t, *J* = 6.6 Hz, 2H), 2.01 (t, *J* = 7.5 Hz, 2H), 1.13 (t, *J* = 6.9 Hz, 6H); ^13^C-NMR (125 MHz, DMSO-d_6_): δ 169.13, 167.85, 162.60, 162.05, 157.70, 152.87, 148.16, 132.02, 110.57, 108.12, 108.10, 96.34, 44.79, 35.87, 32.68, 12.77. HRMS (ESI) *m*/*z* calcd. for C_20_H_27_N_3_O_5_: [M + H]^+^ 389.1951, found 389.1969.

### 3.2. Computational Methodology: Molecular Docking

Compounds **7a**–**k** were docked on the X-ray structure of the HDAC homologue (downloaded with the PDB code 1T69), which was found co-crystalized with a SAHA molecule. During the molecular docking procedure, the types of protein-ligand interactions were carefully analyzed. This procedure was carried out by using the Molegro Virtual Docker V5 software suite, the search algorithm MolDock Simplex Evolution (with a mesh size of 0.3 Å), and the evaluation function MolDock Score. All bound water molecules and ligands were eliminated, and the polar hydrogen was added. The compounds were carefully monitored to analyze the types of ligand-protein interactions involved. All ligands were previously built on *Spartan 08* [60,61] and optimized with the semi empirical AM1 method [62]. The output files were exported as pdb files to be used with the docking software. All flexible bonds were set free to proceed with the flexible computational experiments.

The crystal structures of human HDAC1, 6 and 8 were obtained from the protein data bank (PDB codes 5ICN, 5EDU and 1T69, respectively) [63,64,65]. The former was acquired in complex with the H4K16Hx peptide, with 14 structural errors that were detected and repaired. HDAC6 was obtained in complex with TSA, with 22 structural errors that were detected and repaired. HDAC8 was received in complex with SAHA, with 16 structural errors that were detected and repaired. Previous to docking, all water molecules were removed, and the polar hydrogen was added. After the calibration process, a root-mean-square deviation (RMSD) value of 1.89 Å was found for the H4K16Hx peptide on HDAC1, a value of 0.27 Å for TSA on HDAC6, and a value of 0.51 Å for SAHA on HDAC8. Molecular docking was carried out on the *Molegro Virtual Docker V5* software suite with the search algorithm *MolDock Simplex Evolution* (with a grid of 0.2 Å) and the evaluation function *MolDock* *Score* [66], using a docking spherical zone defined by a 15 Å radius centered on the active site of each HDAC isoform.

### 3.3. Biological Assay

#### 3.3.1. Cell Culture

Breast (BT-474, MDA-MB-231) and prostate (PC3) cancer cell lines (ATCC, Manassas, VA, USA) were cultured and maintained according to the indications of the supplier. The cells were maintained in a humidified atmosphere with 5% CO_2_ at 37 °C.

#### 3.3.2. Sulforhodamine B (SRB) Assay

The cells were seeded in 96-well culture plates at a density of 3000 cells/well and treated in the presence (at various concentrations) and absence of compounds **7a**–**k** or SAHA. All biological assays were performed in triplicate. The cells were incubated for 72–144 h (depending on the cell line) at 37 °C in a humid environment with 95% air and 5% CO_2_. Cell proliferation was determined by using the SRB assay [67]. Briefly, the cells were fixed with 10% trichloroacetic acid at 4 °C for 1 h. The plates were then washed with tap water and air-dried. The cells were stained for 1 h with 0.4% SRB dissolved in 1% acetic acid. To remove unbound stain, plates were washed four times with 1% acetic acid and air-dried. The bound protein stain was solubilized with 10 mM of unbuffered Tris base [tris(hydroxymethyl) aminomethane]. Absorbance was measured at 492 nm in a microplate reader (BioTek, Winooski, VT, USA).

#### 3.3.3. Fluorescence Microscopy and Spectrofluorometric Quantification

For relative fluorescence quantification, the cells were grown in suitable 96-well plates (Corning Costar Model 3904 assay plate; Corning, NY, USA) and treated as aforementioned. Subsequently, they were washed with phosphate-buffered saline and incubated in serum-free DME medium without phenol red. Fluorescence was read on a Biotek Synergy HT plate reader (420 nm excitation, 485 nm emission).

For confocal microscopy, the cells were grown in 2-well chamber slides (Lab-Tek II) at a density of 1 × 10^5^ cells/well. Then the cells were exposed to 10 μM of **7j**, **7k** or SAHA for 48 h. After two washings with Hanks balanced salt solution (HBSS), the cells were fixed with methanol/water (80/20 *v*/*v*) at 4 °C for 30 min, followed by two washings with HBSS. To stain the nuclei, the cells were incubated for 1 h at room temperature in a solution of propidium iodide (PI) (3 μg/mL) and rinsed with HBSS. The chambers were removed, and cover slips were mounted onto slides with VECTASHIELD mounting medium (Vector Laboratories). Images were taken on an Olympus FV1000 confocal upright microscope with a 40X objective lens at excitation and emission wavelengths, respectively, of 536 and 617 nm for red fluorescence (PI) and 420 and 485 nm for fluorescence analogues.

#### 3.3.4. Real Time RT-PCR

For gene expression analysis, the cells were incubated in the presence of SAHA, one of the compounds **7a**–**k** (10 μM) or the vehicle only (DMSO) for 24 h. RNA was extracted with the Trizol reagent and subjected to reverse transcription with the RT transcriptor system. RT-PCR was carried out on a LightCycler 2.0 instrument from Roche (Roche Diagnostics, Mannheim, Germany) according to the following protocol: activation of Taq DNA polymerase and DNA denaturing at 95 °C for 10 min, then 45 amplification cycles consisting of 10 s at 95 °C, 30 s at 60 °C, and 1 s at 72 °C. The oligonucleotides employed were cyclin D1 (CCND1)-F, GAAGATCGTCGCCACCTG; CCND1-R, GACCTCCTCCTCGCACTTCT; p21-F, TCACTGTCTTGTACCCTTGTGC; p21-R, GGCGTTTGGAGTGGTAGAAA; p53-F, GTCCCAAGCAATGGATGATT; and p53-R, TCTGGACCTGGGTCTTCAGT. The gene expression of the housekeeping gene, glyceraldehyde-3-phosphate dehydrogenase (GAPDH), was used as an internal control. It was AGCCACATCGCTGAGACAC for GAPDH-F and GCCCAATACGACCAAATCC for GAPDH-R. 

#### 3.3.5. Statistical Analyses

Data are expressed as the mean ± standard deviation (SD). Statistical differences were determined by one-way ANOVA followed by the Holm–Sidak procedure on a specialized software package (SigmaStat Version 3.5, Jandel Scientific Systat Software, Inc., Richmond, VA, USA).

## 4. Conclusions

A new family of coumarin derivatives functionalized with SAHA-like hydroxamates is herein reported. The compounds show an important antiproliferative activity on two cell lines of breast cancer and one of prostate cancer. The antiproliferative effect of the reference compound (SAHA) and the test compounds was generated at comparable concentrations (10 μM). For instance, both **7i** and SAHA generated a high percentage of antiproliferative activity at a concentration of 10 μM. Furthermore, this activity correlates with the gene regulation detected in two human cancer cell lines. The experiment with propidium iodide revealed the location of compound **7j** in cancer cells, evidenced by the dramatic change in the color of the nucleus in BT-474 cells and to a lesser extent in MDA-MB-231 and PC3 cells. The insights gained presently should certainly be useful for designing derivatives as better fluorescence probes and inhibitors of HDAC enzymes. The docking studies suggest that HDAC1, 6 and 8 are probably inhibited by **7c**, **7e**, **7f**, **7i** and **7j**, which efficiently blocked the active sites of these three isoforms.

## Figures and Tables

**Table 1:**
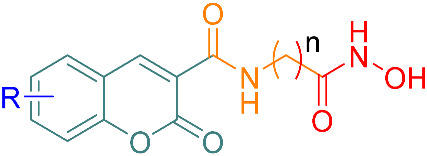
Results of the synthesis of coumarins **7a**–**k** as well as data on absorption and emission.

Product	R	*n*	Yield (%)	λ_ab_ (nm)	λ_em_ (nm)
**7a**	H-	1	50	297	406
**7b**	H-	2	45	303	405
**7c**	H-	5	50	329	405
**7d**	6-MeO-	3	30	363	458
**7e**	6-MeO-	5	87	365	458
**7f**	8-EtO-	5	48	311	486
**7g**	6-Br-	1	46	294	424
**7h**	6-Br-	2	45	295	415
**7i**	6-Br-	5	65	283	424
**7j**	7-Et_2_N-	2	82	419	470
**7k**	7-Et_2_N-	5	82	419	470

**Table 2:** Growth inhibitory effects (GI%) exerted by compounds **7a**–**k** on three cancer cell lines.

Compound	BT-474	MDA-MB-231	PC3
**SAHA**	98.22 ± 1.82 *	97.54 ± 1.59 *	87.86 ± 7.85 *
**7a**	9.90 ± 7.79	17.39 ± 15.15	11.31 ± 7.65
**7b**	40.06 ± 18.94 *	47.81 ± 11.06 *	22.89 ± 19.30
**7c**	95.98 ± 2.61 *	91.61 ± 2.49 *	68.82 ± 22.14 *
**7d**	33.24 ± 15.25	16.57 ± 8.78	8.97 ± 16.21
**7e**	82.08 ± 25.72 *	92.28 ± 5.43 *	71.02 ± 11.55 *
**7f**	91.88 ± 12.54 *	100.08 ± 0.29 *	88.62 ± 14.78 *
**7g**	6.73 ± 7.40	14.00 ± 7.13	5.10 ± 8.93
**7h**	61.83 ± 11.82 *	64.30 ± 17.82 *	32.64 ± 14.31
**7i**	93.47 ± 4.29 *	93.64 ± 3.27 *	75.85 ± 16.43 *
**7j**	70.84 ± 18.62 *	78.50 ± 5.79 *	53.02 ± 13.35 *
**7k**	7.05 ± 13.26	0.00 ± 3.9	7.69 ± 21.95

Data are expressed as the mean ± SD of the percentage of growth inhibition, based on determinations in triplicate in at least three different experiments. * *p* < 0.05 vs. vehicle.

**Table 3:** The effect of compounds **7a**–**k**, when tested against two cancer cell lines, on the expression of genes related to apoptosis and the cell cycle.

**BT-474**			
**Compound**	**p21**	**p53**	**CD1**
**SAHA**	17.39 ± 3.70 *	0.12 ± 0.08 *	0.02 ± 0.00 *
**7c**	3.81 ± 1.08 *	0.57 ± 0.27 *	0.51 ± 0.09 *
**7d**	1.47 ± 0.41	0.47 ± 0.08 *	0.92 ± 0.06
**7e**	5.47 ± 2.01 *	0.08 ± 0.00 *	0.57 ± 0.07 *
**7f**	23.65 ± 6.69 *	0.51 ± 0.22 *	0.40 ± 0.36
**7i**	0.44 ± 0.02 *	0.74 ± 0.21	0.91 ± 0.05
**7j**	26.56 ± 0.44 *	0.57 ± 0.24 *	0.32 ± 0.09 *
**PC3**			
**Compound**	**p21**	**p53**	**CD1**
**SAHA**	7.44 ± 2.24 *	0.12 ± 0.14 *	0.04 ± 0.01 *
**7c**	3.48 ± 0.55 *	0.25 ± 0.17 *	0.61 ± 0.13
**7d**	1.41 ± 0.27	0.49 ± 0.23 *	0.64 ± 0.19
**7e**	3.90 ± 0.43 *	0.18 ± 0.11 *	0.30 ± 0.02 *
**7f**	3.60 ± 0.78 *	0.16 ± 0.20 *	0.32 ± 0.22 *
**7i**	2.07 ± 0.66	0.42 ± 0.39	0.90 ± 0.30
**7j**	3.80 ± 2.33	0.16 ± 0.06 *	0.43 ± 0.27 *

BT-474 and PC3 cells were incubated in the presence (10 μM) or absence of SAHA or compounds **7c**–**f**, **i**–**j** for 24 h. Subsequently, mRNA was extracted, and real-time PCR (qPCR) was performed. Data are expressed as the mean ± SD of the genes/GAPDH, using the normalized ratio of triplicate determinations for mRNA from at least two different experiments. Vehicle-treated cells were arbitrarily given a value of one. * *p* < 0.05 vs. vehicle.

**Table 4:** Interaction energies (ΔG) of molecules docked into the active site of HDACs isoforms.

Ligand	HDAC1 (kcal/mol)	HDAC6 (kcal/mol)	HDAC8 (kcal/mol)
**SAHA**	−86.16	−55.33	−20.42
**7c**	−89.78	−73.66	−25.20
**7e**	−118.77	−68.80	−31.56
**7f**	−111.23	−79.28	−30.17
**7i**	−108.99	−68.82	−29.20
**7j**	−113.59	−80.73	−48.89

**Table 5:** Physicochemical parameter values of compounds SAHA and **7a**–**k**.

Compound	PSA (Å^2^)	Log P	Solubility (mg/mL)	Solubility Class
**SAHA**	108.64	1.92	0.509	Soluble
**7c**	91.57	3.41	0.0665	Soluble
**7e**	117.87	1.75	0.636	Soluble
**7f**	117.87	1.75	0.636	Soluble
**7i**	107.89	1.77	0.38	Soluble
**7j**	98.74	1.52	1.04	Soluble

**Table 6:** Pharmacokinetic parameter of compounds SAHA and **7a**–**k**.

Compound	HIA	BBB	Pgp Substrate	CYP1A2 Inhibitor
**SAHA**	High	No	Yes	No
**7c**	High	No	No	Yes
**7e**	High	No	Yes	No
**7f**	High	No	Yes	No
**7i**	High	No	Yes	No
**7j**	High	No	Yes	No

## Supplementary Materials

The supplementary materials are available online.

## References

[1] Fylaktakidou K.C., Hadjipavlou-Litina D.J., Litinas K.E., Nicolaides D.N. (2004). Natural and Synthetic Coumarin Derivatives with Anti-Inflammatory / Antioxidant Activities. Curr. Pharm. Des..

[2] Saeedi M., Goli F., Mahdavi M., Dehghan G., Faramarzi M.A., Foroumadi A., Shafiee A. (2014). Synthesis and Biological Investigation of Some Novel Sulfonamide and Amide Derivatives Containing Coumarin Moieties. Iran. J. Pharm. Res..

[3] Atmaca M., Bilgin H.M., Obay B.D., Diken H., Kelle M., Kale E. (2011). The hepatoprotective effect of coumarin and coumarin derivates on carbon tetrachloride-induced hepatic injury by antioxidative activities in rats. J. Physiol. Biochem..

[4] Peng X.-M., Damu G.L.V., Zhou C.-H. (2013). Current Developments of Coumarin Compounds in Medicinal Chemistry. Curr. Pharm. Des..

[5] Završnik D., Muratović S., Makuc D., Plavec J., Cetina M., Nagl A., De Clercq E., Balzarini J., Mintas M. (2011). Benzylidene-bis-(4-Hydroxycoumarin) and Benzopyrano-Coumarin Derivatives: Synthesis, ^1^H/^13^C-NMR Conformational and X-ray Crystal Structure Studies and In Vitro Antiviral Activity Evaluations. Molecules.

[6] Wang S.-F., Yin Y., Wu X., Qiao F., Sha S., Lv P.-C., Zhao J., Zhu H.-L. (2014). Synthesis, molecular docking and biological evaluation of coumarin derivatives containing piperazine skeleton as potential antibacterial agents. Bioorg. Med. Chem..

[7] Ostrov D.A., Hernández Prada J.A., Corsino P.E., Finton K.A., Le N., Rowe T.C. (2007). Discovery of novel DNA gyrase inhibitors by high-throughput virtual screening. Antimicrob. Agents Chemother..

[8] Shamsa F., Foroumadi A., Shamsa H., Samadi N., Faramarzi M.A., Shafiee A. (2011). Synthesis and In-vitro Antibacterial Activities of Acetylanthracene and Acetylphenanthrene Derivatives of Some Fluoroquinolones. Iran. J. Pharm. Res..

[9] Patel R.V., Patel P.K., Kumari P., Rajani D.P., Chikhalia K.H. (2012). Synthesis of benzimidazolyl-1,3,4-oxadiazol-2ylthio-*N*-phenyl (benzothiazolyl) acetamides as antibacterial, antifungal and antituberculosis agents. Eur. J. Med. Chem..

[10] Keri R.S., Sasidhar B.S., Nagaraja B.M., Santos M.A. (2015). Recent progress in the drug development of coumarin derivatives as potent antituberculosis agents. Eur. J. Med. Chem..

[11] Nair R.V., Fisher E.P., Safe S.H., Cortez C., Harvey R.G., DiGiovanni J. (1991). Novel coumarins as potential anticarcinogenic agents. Carcinogenesis.

[12] Gu X., Zhou Y., Wu X., Wang F., Zhang C.-Y., Du C., Shen L., Chen X., Shi J., Liu C. (2014). Antidepressant-like effects of auraptenol in mice. Sci. Rep..

[13] Capra J.C., Cunha M.P., Machado D.G., Zomkowski A.D.E., Mendes B.G., Santos A.R.S., Pizzolatti M.G., Rodrigues A.L.S. (2010). Antidepressant-like effect of scopoletin, a coumarin isolated from *Polygala sabulosa* (Polygalaceae) in mice: Evidence for the involvement of monoaminergic systems. Eur. J. Pharmacol..

[14] Yuce B., Danis O., Ogan A., Sener G., Bulut M., Yarat A. (2009). Antioxidative and lipid lowering effects of 7,8-dihydroxy-3-(4-methylphenyl) coumarin in hyperlipidemic rats. Arzneimittelforschung.

[15] Orhan I.E., Gulcan H.O. (2015). Coumarins: Auspicious Cholinesterase and Monoamine Oxidase Inhibitors. Curr. Top. Med. Chem..

[16] Brühlmann C., Ooms F., Carrupt P.-A., Testa B., Catto M., Leonetti F., Altomare C., Carotti A. (2001). Coumarins Derivatives as Dual Inhibitors of Acetylcholinesterase and Monoamine Oxidase. J. Med. Chem..

[17] Katsori A.-M., Hadjipavlou-Litina D. (2014). Coumarin derivatives: An updated patent review (2012–2014). Expert Opin. Ther. Pat..

[18] Borges F., Roleira F., Milhazes N., Santana L., Uriarte E. (2005). Simple Coumarins and Analogues in Medicinal Chemistry: Occurrence, Synthesis and Biological Activity. Curr. Med. Chem..

[19] Thakur A., Singla R., Jaitak V. (2015). Coumarins as anticancer agents: A review on synthetic strategies, mechanism of action and SAR studies. Eur. J. Med. Chem..

[20] Egan D., James P., Cooke D., O’Kennedy R. (1997). Studies on the cytostatic and cytotoxic effects and mode of action of 8-nitro-7-hydroxycoumarin. Cancer Lett..

[21] Seidel C., Schnekenburger M., Zwergel C., Gaascht F., Mai A., Dicato M., Kirsch G., Valente S., Diederich M. (2014). Novel inhibitors of human histone deacetylases: Design, synthesis and bioactivity of 3-alkenoylcoumarines. Bioorg. Med. Chem. Lett..

[22] Wu X.-Q., Huang C., Jia Y.-M., Song B.-A., Li J., Liu X.-H. (2014). Novel coumarin-dihydropyrazole thio-ethanone derivatives: Design, synthesis and anticancer activity. Eur. J. Med. Chem..

[23] Belluti F., Fontana G., Bo L.D., Carenini N., Giommarelli C., Zunino F. (2010). Design, synthesis and anticancer activities of stilbene-coumarin hybrid compounds: Identification of novel proapoptotic agents. Bioorg. Med. Chem..

[24] Ding J., Liu J., Zhang Z., Guo J., Cheng M., Wan Y., Wang R., Fang Y., Guan Z., Jin Y. (2020). Design, synthesis and biological evaluation of coumarin-based *N*-hydroxycinnamamide derivatives as novel histone deacetylase inhibitors with anticancer activities. Bioorg. Chem..

[25] Yang F., Zhao N., Song J., Zhu K., Jiang C.-S., Shan P., Zhang H. (2019). Design, Synthesis and Biological Evaluation of Novel Coumarin-Based Hydroxamate Derivatives as Histone Deacetylase (Hdac) Inhibitors with Antitumor Activities. Molecules.

[26] Zhao N., Yang F., Han L., Qu Y., Ge D., Zhang H. (2020). Development of Coumarin-Based Hydroxamates as Histone Deacetylase Inhibitors with Antitumor Activities. Molecules.

[27] Manal M., Chandrasekar M.J.N., Gomathi Priya J., Nanjan M.J. (2016). Inhibitors of histone deacetylase as antitumor agents: A critical review. Bioorg. Chem..

[28] Qiu X., Xiao X., Li N., Li Y. (2017). Histone deacetylases inhibitors (HDACis) as novel therapeutic application in various clinical diseases. Prog. Neuro-Psychopharmacol. Biol. Psychiatry.

[29] Tasior M., Kim D., Singha S., Krzeszewski M., Ahn K.H., Gryko D.T. (2015). π-Expanded coumarins: Synthesis, Optical Properties and Applications. J. Mater. Chem. C.

[30] Yan F., Sun X., Zu F., Bai Z., Jiang Y., Fan K., Wang J. (2018). Fluorescent probes for detecting cysteine. Methods Appl. Fluoresc..

[31] Jung Y., Jung J., Huh Y., Kim D. (2018). Benzo[*g*]coumarin-Based Fluorescent Probes for Bioimaging Applications. J. Anal. Methods Chem..

[32] Rooker D.R., Buccella D. (2015). Real-time detection of histone deacetylase activity with a small molecule fluorescent and spectrophotometric probe. Chem. Sci..

[33] Baba R., Hori Y., Mizukami S., Kikuchi K. (2012). Development of a Fluorogenic Probe with a Transesterification Switch for Detection of Histone Deacetylase Activity. J. Am. Chem. Soc..

[34] Hoffmann K., Brosch G., Loidl P., Jung M. (1999). A non-isotopic assay for histone deacetylase activity. Nucleic Acids Res..

[35] Heltweg B., Dequiedt F., Marshall B.L., Brauch C., Yoshida M., Nishino N., Verdin E., Jung M. (2004). Subtype Selective Substrates for Histone Deacetylases. J. Med. Chem..

[36] Singh R.K., Mandal T., Balasubramanian N., Cook G., Srivastava D.K. (2011). Coumarin-suberoylanilide hydroxamic acid as a fluorescent probe for determining binding affinities and off-rates of histone deacetylase inhibitors. Anal. Biochem..

[37] Al-Yacoub N., Fecker L.F., Möbs M., Plötz M., Braun F.K., Sterry W., Eberle J. (2012). Apoptosis Induction by SAHA in Cutaneous T-Cell Lymphoma Cells Is Related to Downregulation of c-FLIP and Enhanced TRAIL Signaling. J. Investig. Dermatol..

[38] Wittine K., Ratkaj I., Benci K., Suhina T., Mandić L., Ilić N., Pavelić S.K., Pavelić K., Mintas M. (2016). The novel coumarin[3,2-c]thiophene and its hydroxamic acid and ureido derivatives: Synthesis and cytostatic activity evaluations. Med. Chem. Res..

[39] Abdizadeh R., Hadizadeh F., Abdizadeh T. (2020). QSAR analysis of coumarin-based benzamides as histone deacetylase inhibitors using CoMFA, CoMSIA and HQSAR methods. J. Mol. Struct..

[40] Garcia S., Vazquez J.L., Renteria M., Aguilar-Garduño I.G., Delgado F., Trejo-Duran M., Garcia-Revilla M.A., Alvarado-Mendez E., Vazquez M.A. (2016). Synthesis and experimental-computational characterization of nonlinear optical properties of triazacyclopentafluorene-coumarin derivatives. Opt. Mater. (Amst.).

[41] Bahena L., Cervantes C., Soto-Arredondo K.J., Martínez-Alfaro M., Zarco N., García-Revilla M.A., Alcaraz-Contreras Y., Tirado L.P., Vázquez M.A., Robles J. (2017). In Silico, Synthesis and Biological Investigations of Pyrrolo[3,4-C]Pyrrole Hydroxamic Acid Derivatives as Potential Anticancer Agents. J. Mex. Chem. Soc..

[42] Song A., Wang X., Lam K.S. (2003). A convenient synthesis of coumarin-3-carboxylic acids via Knoevenagel condensation of Meldrum’s acid with ortho-hydroxyaryl aldehydes or ketones. Tetrahedron Lett..

[43] Maggi R., Bigi F., Carloni S., Mazzacani A., Sartori G. (2001). Uncatalysed reactions in water: Part 2. Preparation of 3-carboxycoumarins. Green Chem..

[44] Kim N.H., Kim S.-N., Kim Y.K. (2011). Involvement of HDAC1 in E-cadherin expression in prostate cancer cells; its implication for cell motility and invasion. Biochem. Biophys. Res. Commun..

[45] Huang L., Pardee A.B. (2000). Suberoylanilide hydroxamic acid as a potential therapeutic agent for human breast cancer treatment. Mol. Med..

[46] Butler L.M., Agus D.B., Scher H.I., Higgins B., Rose A., Cordon-Cardo C., Thaler H.T., Rifkind R.A., Marks P.A., Richon V.M. (2000). Suberoylanilide hydroxamic acid, an inhibitor of histone deacetylase, suppresses the growth of prostate cancer cells in vitro and in vivo. Cancer Res..

[47] Patra N., De U., Kim T.H., Lee Y.J., Ahn M.Y., Kim N.D., Yoon J.H., Choi W.S., Moon H.R., Lee B.M. (2013). A novel histone deacetylase (HDAC) inhibitor MHY219 induces apoptosis via up-regulation of androgen receptor expression in human prostate cancer cells. Biomed. Pharmacother..

[48] Knutson A.K., Welsh J., Taylor T., Roy S., Wang W.-L.W., Tenniswood M. (2012). Comparative effects of histone deacetylase inhibitors on p53 target gene expression, cell cycle and apoptosis in MCF-7 breast cancer cells. Oncol. Rep..

[49] Kong Y., Jung M., Wang K., Grindrod S., Velena A., Lee S.A., Dakshanamurthy S., Yang Y., Miessau M., Zheng C. (2011). Histone Deacetylase Cytoplasmic Trapping by a Novel Fluorescent HDAC Inhibitor. Mol. Cancer Ther..

[50] Longworth M.S., Laimins L.A. (2006). Histone deacetylase 3 localizes to the plasma membrane and is a substrate of Src. Oncogene.

[51] Gui C.-Y., Ngo L., Xu W.S., Richon V.M., Marks P.A. (2004). Histone deacetylase (HDAC) inhibitor activation of p21WAF1 involves changes in promoter-associated proteins, including HDAC1. Proc. Natl. Acad. Sci. USA.

[52] Greer C.B., Tanaka Y., Kim Y.J., Xie P., Zhang M.Q., Park I.-H., Kim T.H. (2015). Histone Deacetylases Positively Regulate Transcription through the Elongation Machinery. Cell Rep..

[53] De U., Kundu S., Patra N., Ahn M.Y., Ahn J.H., Son J.Y., Yoon J.H., Moon H.R., Lee B.M., Kim H.S. (2015). A New Histone Deacetylase Inhibitor, MHY219, Inhibits the Migration of Human Prostate Cancer Cells via HDAC1. Biomol. Ther. (Seoul).

[54] Park S.Y., Jun J.A., Jeong K.J., Heo H.J., Sohn J.S., Lee H.Y., Park C.G., Kang J. (2011). Histone deacetylases 1, 6 and 8 are critical for invasion in breast cancer. Oncol. Rep..

[55] Daina A., Michielin O., Zoete V. (2017). SwissADME: A free web tool to evaluate pharmacokinetics, drug-likeness and medicinal chemistry friendliness of small molecules. Sci. Rep..

[56] Holohan C., Van Schaeybroeck S., Longley D.B., Johnston P.G. (2013). Cancer drug resistance: An evolving paradigm. Nat. Rev. Cancer.

[57] Herrera-Martínez M., Orozco-Samperio E., Montaño S., Ariza-Ortega J.A., Flores-García Y., López-Contreras L. (2020). Vorinostat as potential antiparasitic drug. Eur. Rev. Med. Pharmacol. Sci..

[58] Iwamoto M., Friedman E.J., Sandhu P., Agrawal N.G.B., Rubin E.H., Wagner J.A. (2013). Clinical pharmacology profile of vorinostat, a histone deacetylase inhibitor. Cancer Chemother. Pharmacol..

[59] Mravljak J., Ojsteršek T., Pajk S., Sollner Dolenc M. (2013). Coumarin-based dual fluorescent spin-probes. Tetrahedron Lett..

[60] Shao Y., Molnar L.F., Jung Y., Kussmann J., Ochsenfeld C., Brown S.T., Gilbert A.T.B., Slipchenko L.V., Levchenko S.V., O’Neill D.P. (2006). Advances in methods and algorithms in a modern quantum chemistry program package. Phys. Chem. Chem. Phys..

[61] Wavefunction, Inc.. https://www.wavefun.com.

[62] Rocha G.B., Freire R.O., Simas A.M., Stewart J.J.P. (2006). RM1: A reparameterization of AM1 for H, C, N, O, P, S, F, Cl, Br, and I. J. Comput. Chem..

[63] Watson P.J., Millard C.J., Riley A.M., Robertson N.S., Wright L.C., Godage H.Y., Cowley S.M., Jamieson A.G., Potter B.V.L., Schwabe J.W.R. (2016). Insights into the activation mechanism of class I HDAC complexes by inositol phosphates. Nat. Commun..

[64] Hai Y., Christianson D.W. (2016). Histone deacetylase 6 structure and molecular basis of catalysis and inhibition. Nat. Chem. Biol..

[65] Somoza J.R., Skene R.J., Katz B.A., Mol C., Ho J.D., Jennings A.J., Luong C., Arvai A., Buggy J.J., Chi E. (2016). Structural Snapshots of Human HDAC8 Provide Insights into the Class I Histone Deacetylases. Structure.

[66] Thomsen R., Christensen M.H. (2006). MolDock: A New Technique for High-Accuracy Molecular Docking. J. Med. Chem..

[67] Keepers Y.P., Pizao P.E., Peters G.J., van Ark-Otte J., Winograd B., Pinedo H.M. (1991). Comparison of the sulforhodamine B protein and tetrazolium (MTT) assays for in vitro chemosensitivity testing. Eur. J. Cancer Clin. Oncol..

