# Supplementary material for: Design of Fluorescent Coumarin-Hydroxamic Acid Derivatives as Inhibitors of HDACs: Synthesis, Anti-Proliferative Evaluation and Docking Studies
Source: Molecules. 2020 Nov 4;25(21):5134. doi: 10.3390/molecules25215134 (PMC7662212; doi:10.3390/molecules25215134)
Supplement: Supplementary file 1 [file molecules-25-05134-s001.pdf]

Supplementary Material

Section	Contents	Page
1	Synthetic procedures and spectroscopic data of compounds	1-108
2	Antiproliferative activity	109
3	Docking of coumarin and hydroxycoumarins with HDAC1, HDAC6 and HDAC8.	110-111

1. Synthetic procedures and spectroscopic data of compounds

All solvents and reagents were commercially available and used without further purification. Melting points were determined on an RY-1 MP apparatus. Absorption spectra were recorded on a Perkin-Elmer Lambda 50 apparatus and emission spectra on an FL-7000 FL spectrometer. ESI-HMRS spectra were obtained on a Maxis Impact ESI-QTOF-MS spectrometer and a Bruker Daltonics mass spectrometer. ^{1}H NMR and ^{13}C NMR spectra were acquired in $CDCl_3$ or DMSO-d_6 solutions at 25 °C with TMS and solvent signals allotted as internal standards, using the following NMR spectrometers: a Bruker Avance III HD with a Bruker Ascent 400 MHz magnet or with a Bruker Ultra Shield 500 MHz HD magnet. Chemical shifts are reported in ppm (δ) and J-values are given in hertz (Hz).

General procedure for the preparation of coumarin-3-carboxylic acids (3a-f)

In a round-bottom flask, a solution of the corresponding salicylaldehyde (1 mmol) and Meldrum's acid (1.2 mmol) was stirred in water and maintained at reflux for 5-6 h. The purification was carried out by recrystallization with MeOH and cold water.

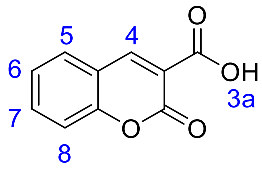

(3a) White solid (82% yield); mp 183-184 ºC; IR (KBr) $\nu_{max/cm-1}$= 1746, 1685, 1614, 1569; UV-vis (MeOH) $\lambda_{max/nm}$= 296; ^{1}H NMR (500 MHz, $CDCl_3$) δ 12.26 (s, 1H, H-3a), 8.96 (s, 1H, H-4), 7.80 (dd, *J*= 14, 7.7 Hz, 2H, H-5,8), 7.51 (t, *J*= 8.9 Hz, 2H, H-6,7); ^{13}C NMR (125 MHz, $CDCl_3$) δ 164.10, 162.42, 154.59, 151.52, 135.79, 130.52, 126.28, 118.48, 117.23, 114.90; HRMS (ESI) *m/z* calcd. for $C_{10}H_6O_4$ $[M+H]^+$ 191.0339, found 191.0342.

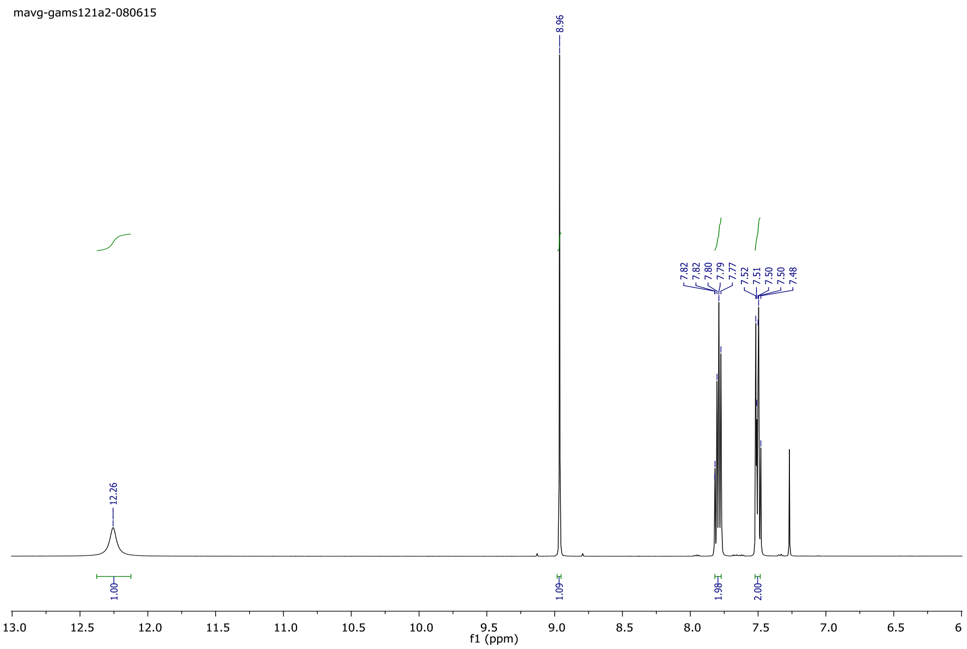

^{1}H spectrum of **3a** in $CDCl_3$, 500 MHz

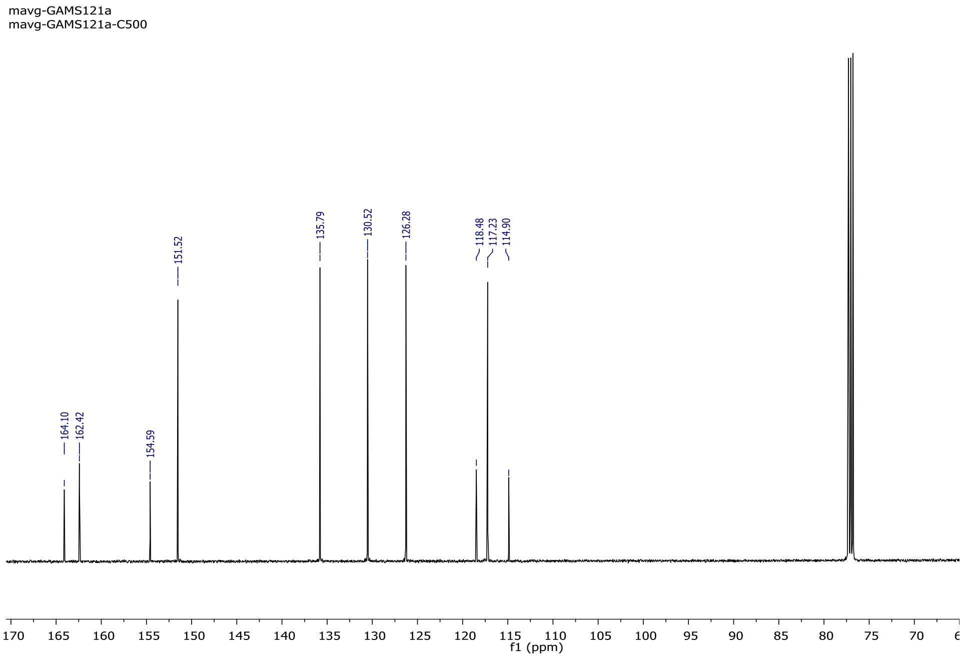

^{13}C spectrum of **3a** in $CDCl_3$, 125 MHz

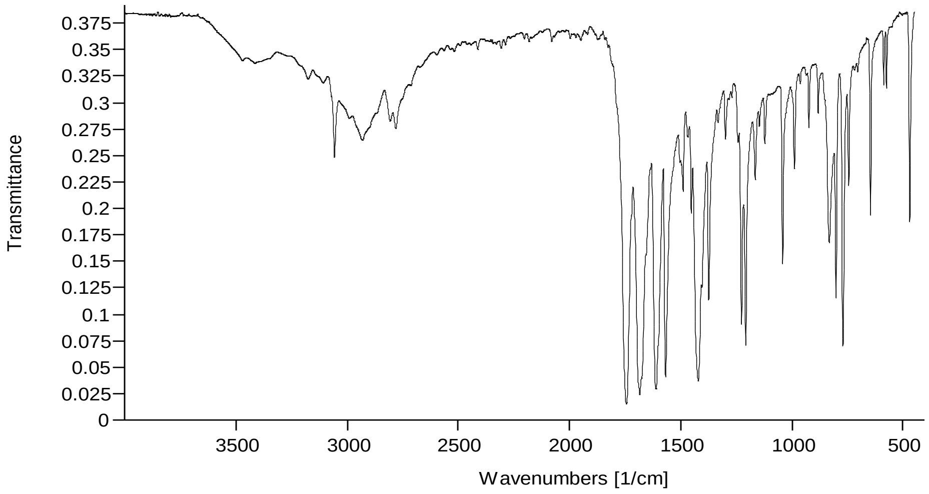

IR spectrum of **3a** in KBr

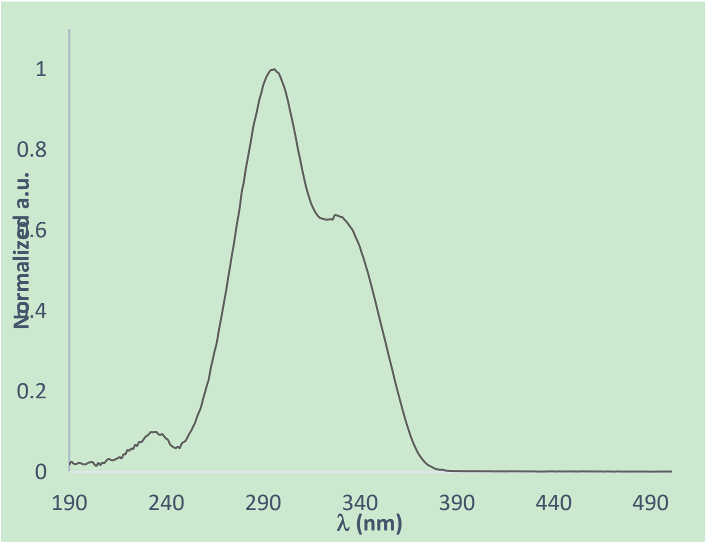

UV spectrum of **3a** in MeOH

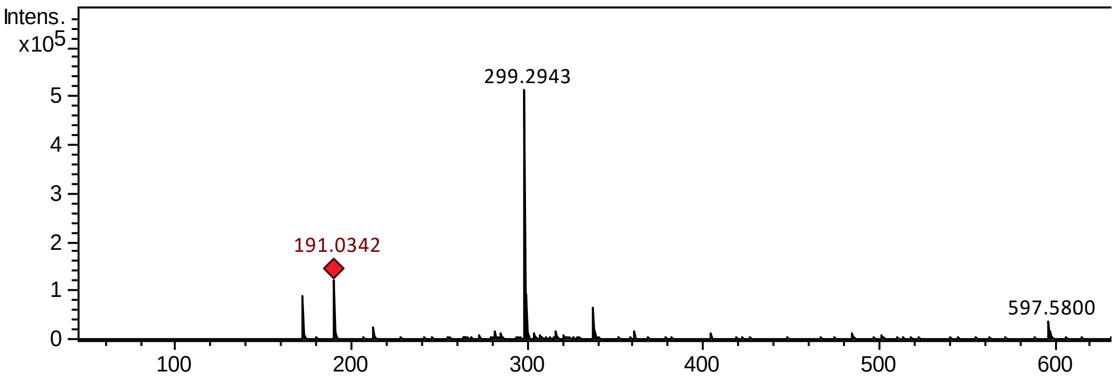

Mass spectrum, ESI(+), of **3a**

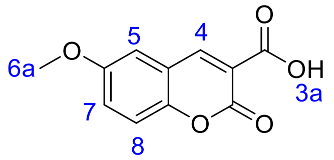

(**3b**) Yellowish solid (90% yield); mp 202-205 ºC; IR (KBr) $\nu_{max/cm-1}$= 1761, 1607, 1622, 1574; UV-vis (MeOH) $\lambda_{max/nm}$= 295; ^{1}H NMR (500 MHz, $CDCl_3$) δ 12.37 (s, 1H, H-3a), 8.91 (s, 1H, H-4), 7.43 (d, *J*= 9.1 Hz, 1H, H-8), 7.36 (dd, *J*= 9.1, 2.8 Hz, 1H, H-7), 7.12 (d, *J*= 2.8 Hz, 1H, H-5), 3.91 (s, 2H, H-6a); ^{13}C NMR (125 MHz, $CDCl_3$) δ 164.22, 162.55, 157.25, 151.22, 149.22, 124.44, 118.90, 118.34, 115.00, 111.01, 56.05; HRMS (ESI) *m/z* calcd. for $C_{11}H_8O_5$ $[M+H]^+$ 221.0444, found 221.0446.

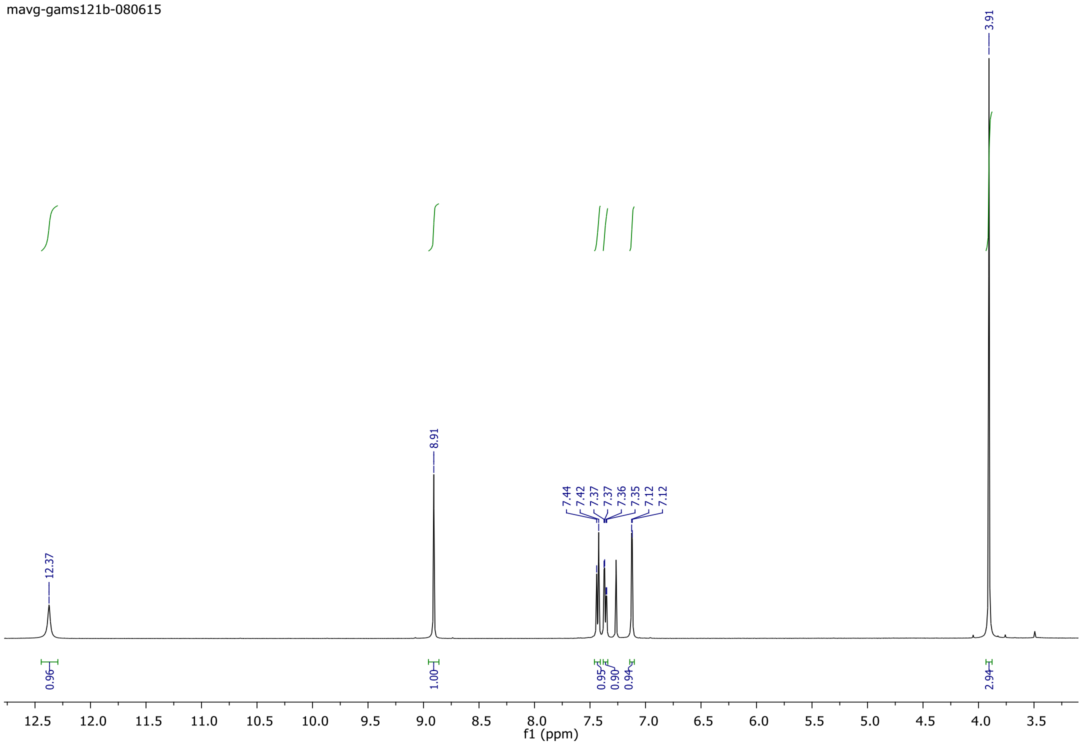

^{1}H spectrum of **3b** in $CDCl_3$, 500 MHz

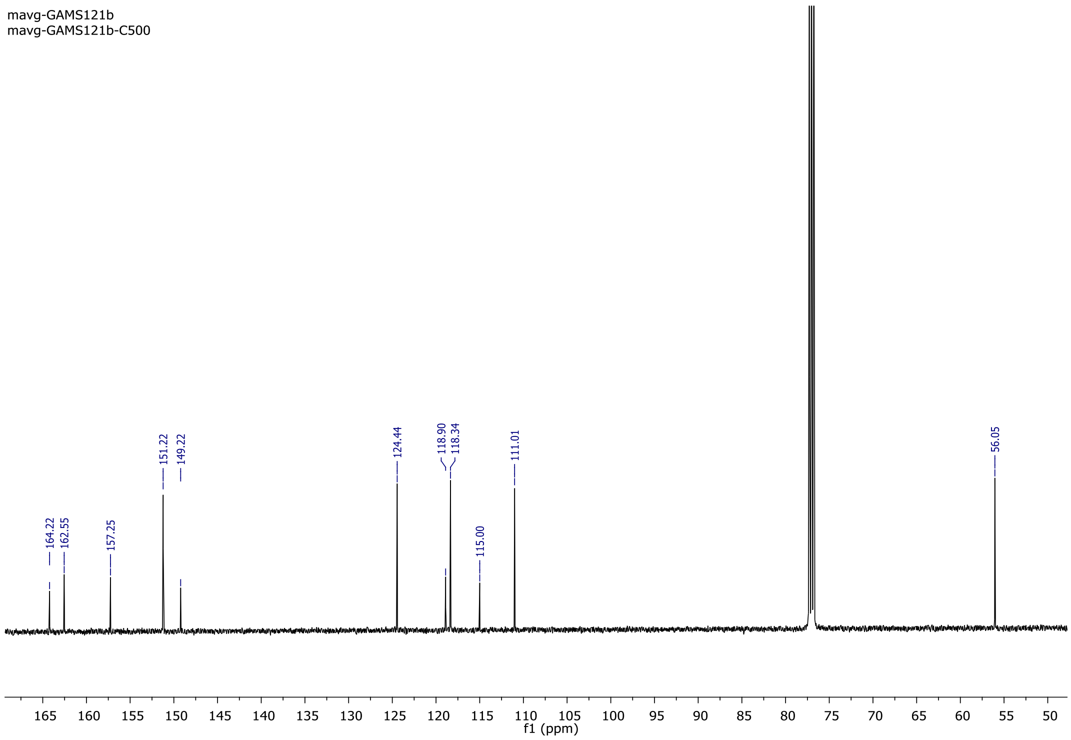

^{13}C spectrum of **3b** in $CDCl_3$, 125 MHz

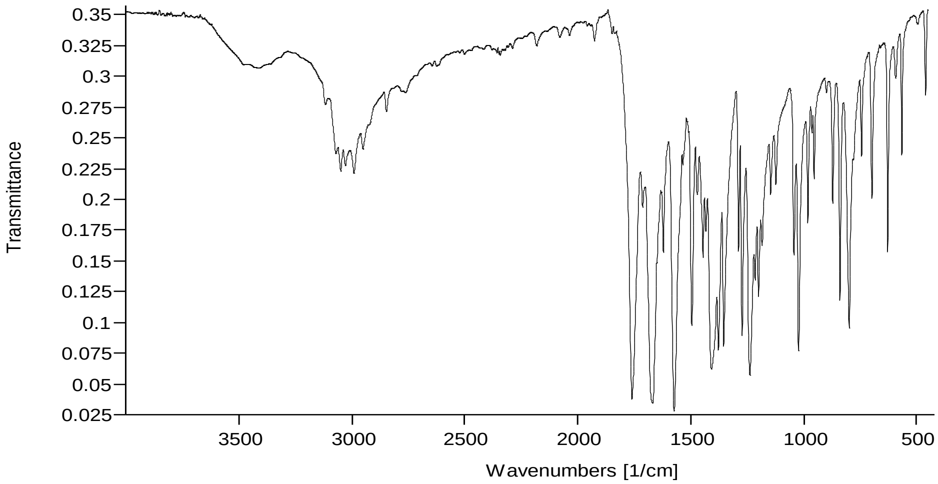

IR spectrum of **3b** in KBr

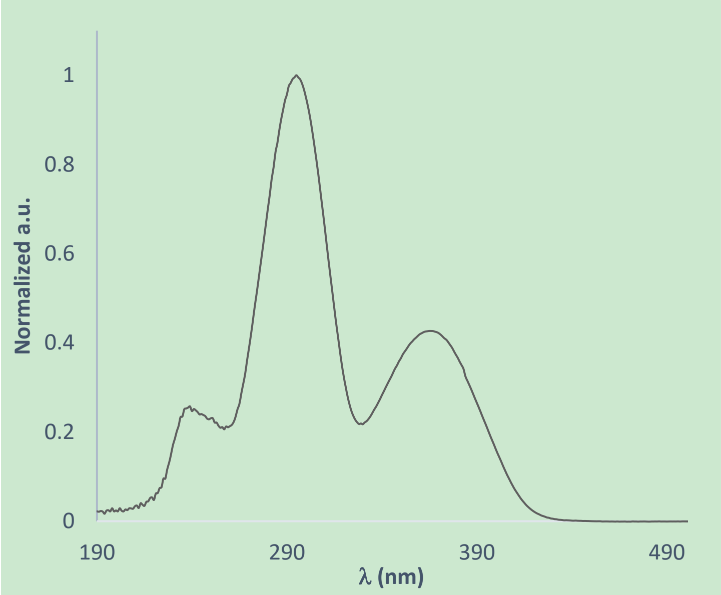

UV spectrum of **3b** in MeOH

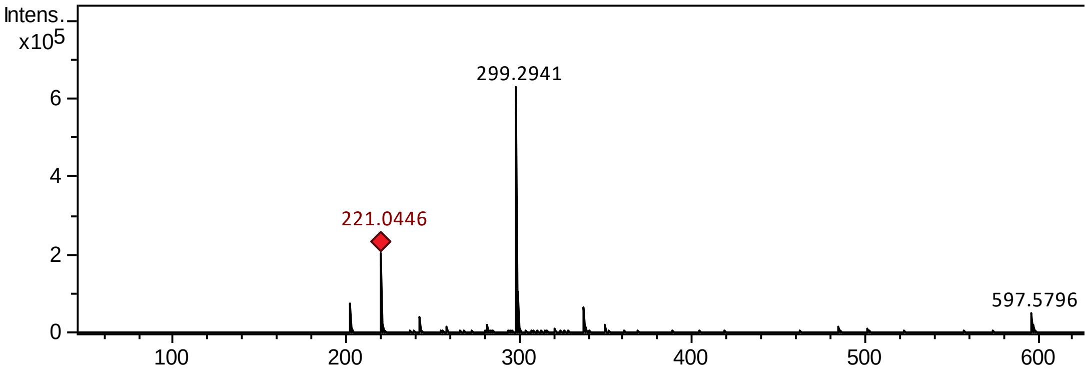

Mass spectrum, ESI(+), of **3b**

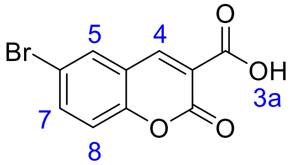

(3c) White solid (85% yield); mp 195-197 ºC; IR (KBr) $\nu_{max/cm-1}$= 1737, 1716, 1690, 1609; UV-vis (MeOH) $\lambda_{max/nm}$= 291; ^{1}H NMR (500 MHz, $CDCl_3$) δ 12.06 (s, 1H, H-3a), 8.87 (s, 1H, H-4), 7.90 (d, *J* = 2.1 Hz, 1H, H-8), 7.87 (dd, *J* = 8.8, 2.2 Hz, 1H, H-7), 7.40 (d, *J*= 8.8 Hz, 1H, H-5); ^{13}C NMR (125 MHz, $CDCl_3$) δ 163.41, 161.82, 153.33, 150.09, 138.42, 132.45, 119.82, 118.97, 116.01; HRMS (ESI) *m/z* calcd. for $C_{10}H_5BrO_4$ $[M+H]^+$ 268.9444, found 270.9428.

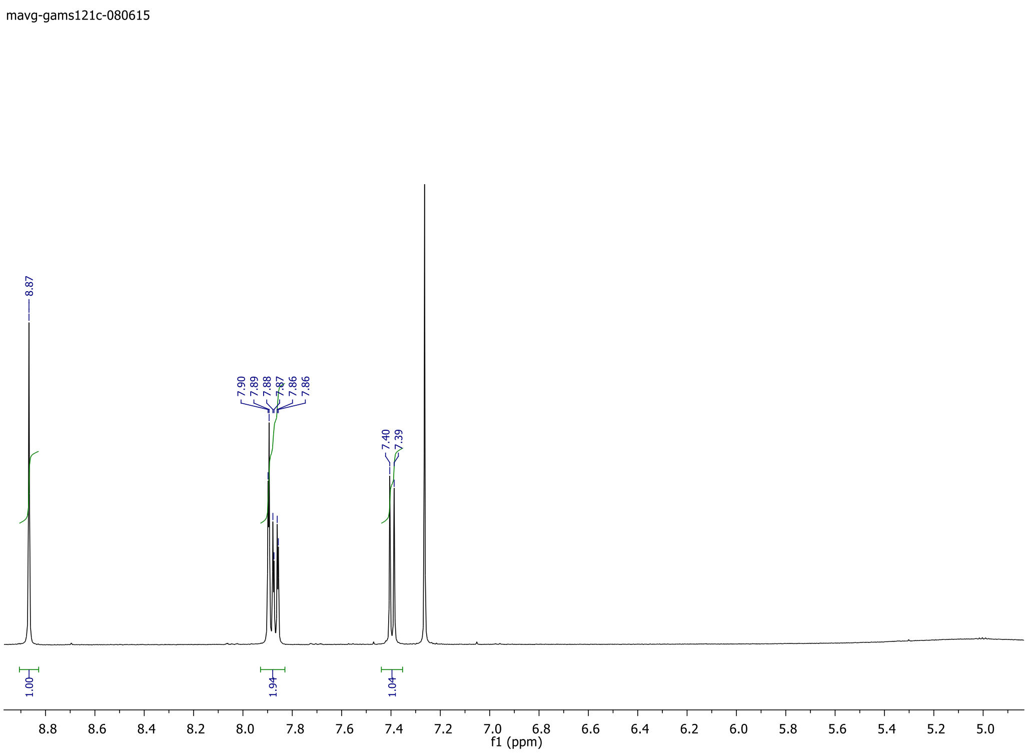

^{1}H spectrum of **3c** in $CDCl_3$, 500 MHz

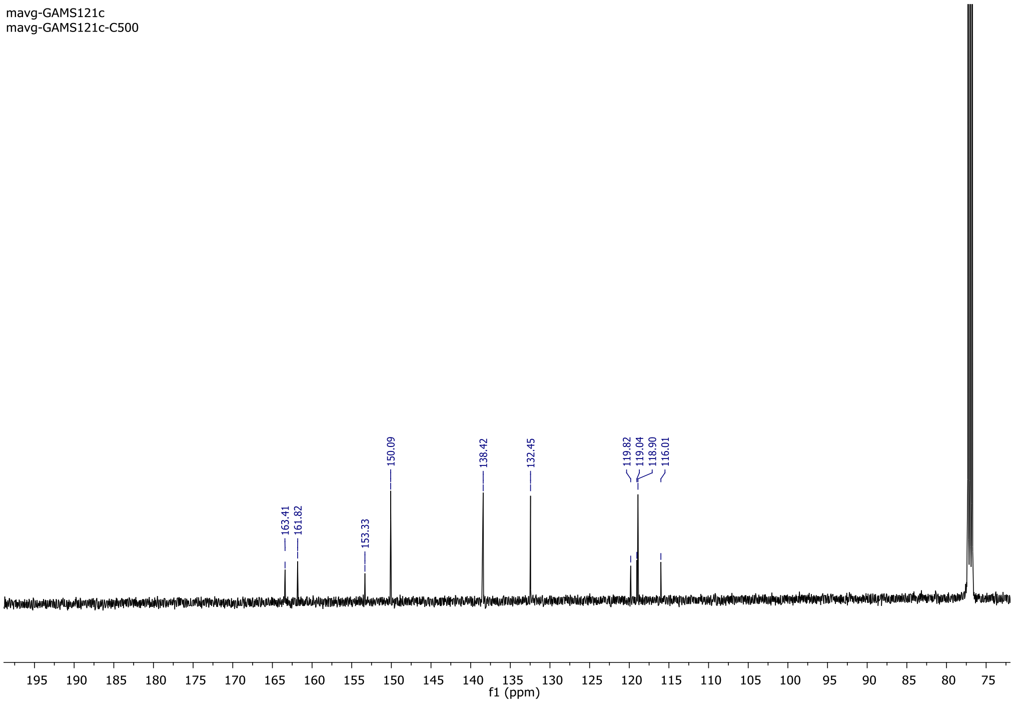

^{13}C spectrum of **3c** in $CDCl_3$, 125 MHz

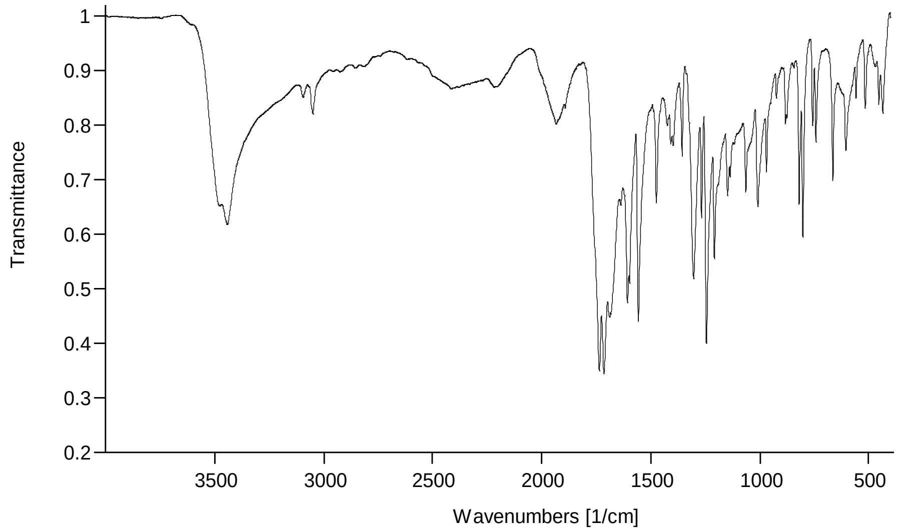

IR spectrum of **3c** in KBr

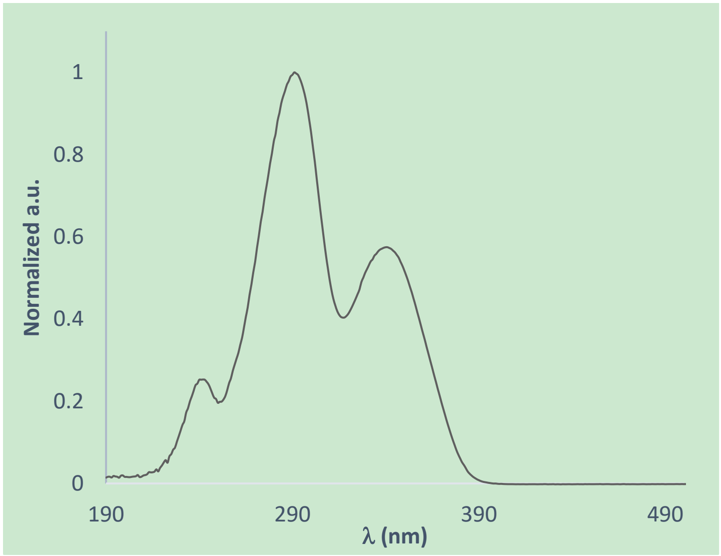

Uv spectrum of **3c** in MeOH

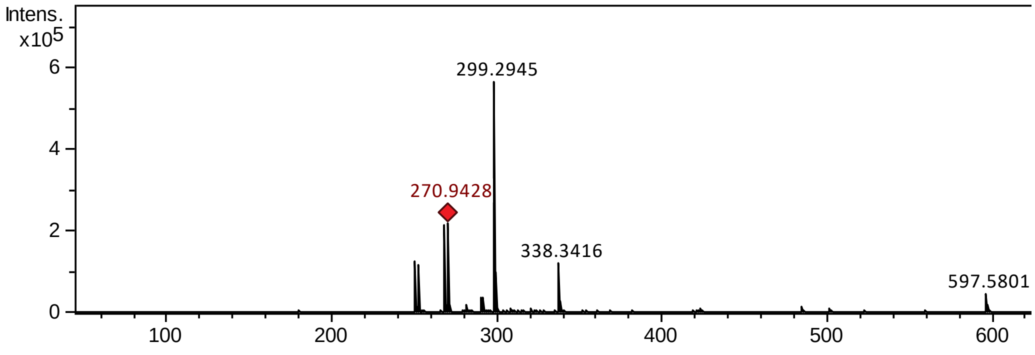

Mass spectrum, ESI(+), of **3c**

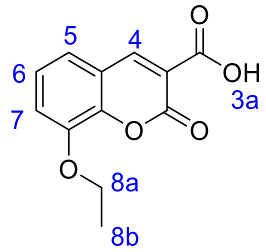

(3d) White solid (85% yield); mp 195-197 ºC; IR (KBr) $\nu_{max/cm-1}$= 1737, 1716, 1690, 1609; UV-vis (MeOH) $\lambda_{max/nm}$= 291; ^{1}H NMR (500 MHz, $CDCl_3$) δ 12.06 (s, 1H, H-3a), 8.87 (s, 1H, H-4), 7.90 (d, *J* = 2.1 Hz, 1H, H-8), 7.87 (dd, *J* = 8.8, 2.2 Hz, 1H, H-7), 7.40 (d, *J*= 8.8 Hz, 1H, H-5); ^{13}C NMR (125 MHz, $CDCl_3$) δ 163.41, 161.82, 153.33, 150.09, 138.42, 132.45, 119.82, 118.97, 116.01; HRMS (ESI) *m/z* calcd. for $C_{10}H_5BrO_4$ $[M+H]^+$ 268.9444, found 270.9428.

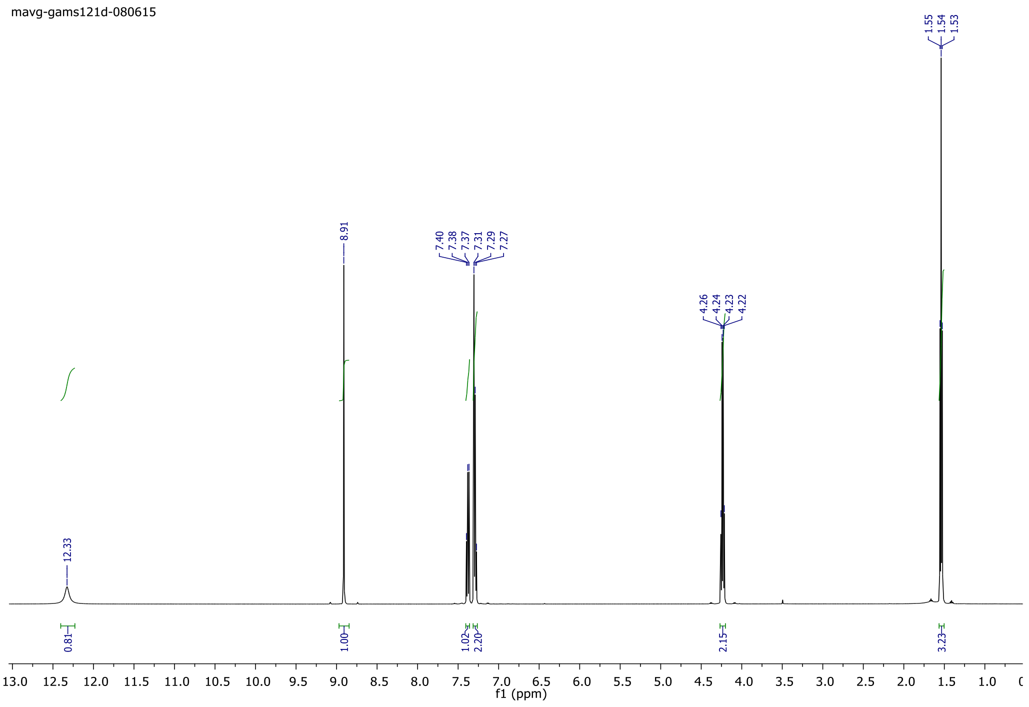

^{1}H spectrum of **3d** in $CDCl_3$, 500 MHz

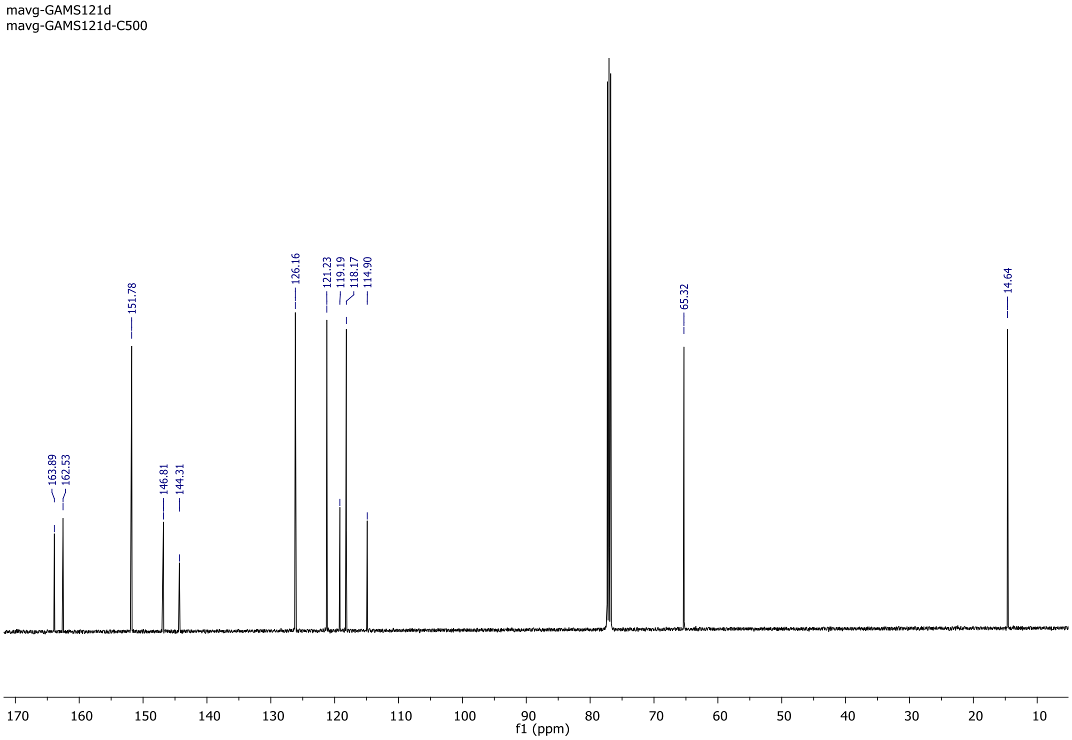

^{13}C spectrum of **3d** in $CDCl_3$, 125 MHz

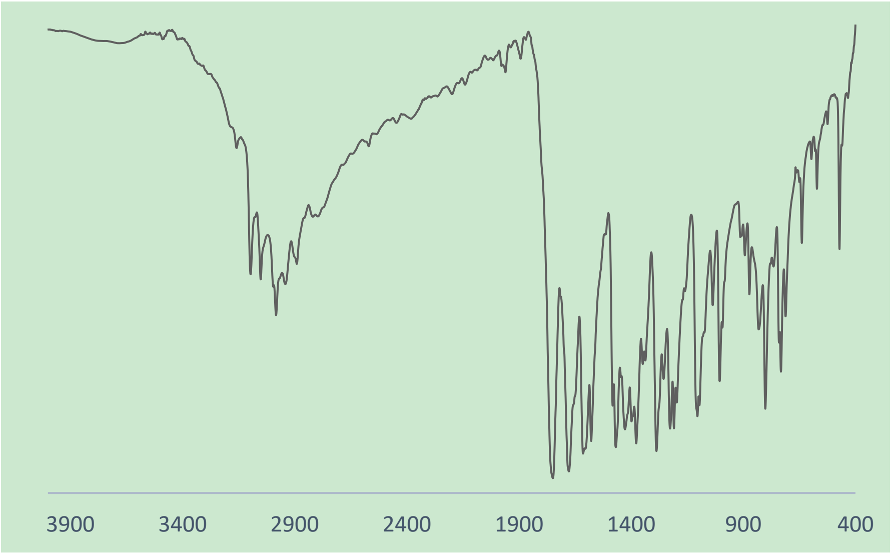

IR spectrum of **3d** in KBr

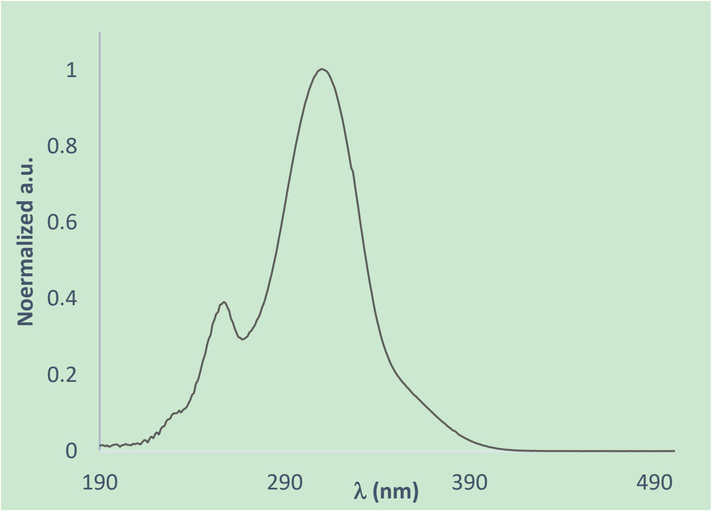

UV spectrum of **3d** in MeOH

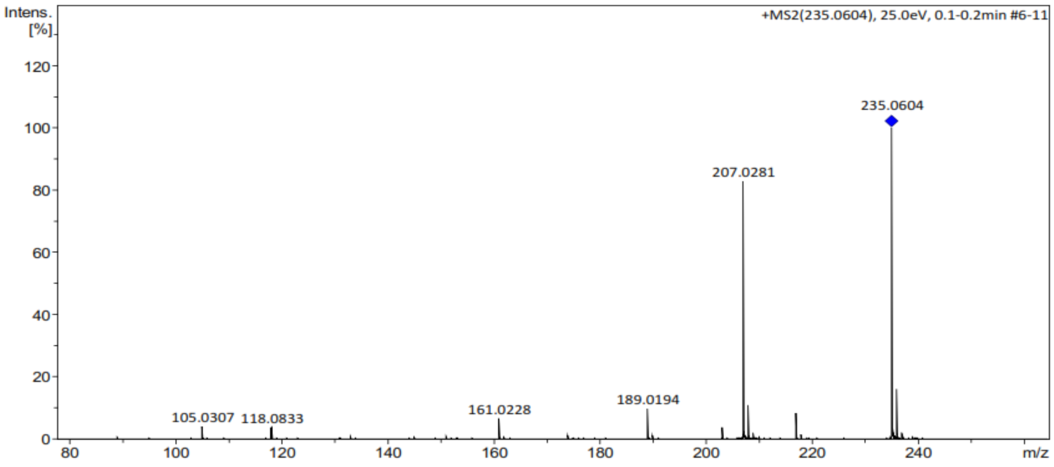

Mass spectrum of **3d**

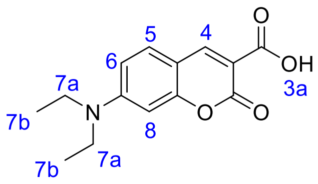

(3e) White solid (85% yield); mp 195-197 ºC; IR (KBr) $\nu_{max/cm-1}$= 1737, 1716, 1690, 1609; UV-vis (MeOH) $\lambda_{max/nm}$= 291; ^{1}H NMR (500 MHz, $CDCl_3$) δ 12.06 (s, 1H, H-3a), 8.87 (s, 1H, H-4), 7.90 (d, *J* = 2.1 Hz, 1H, H-8), 7.87 (dd, *J* = 8.8, 2.2 Hz, 1H, H-7), 7.40 (d, *J*= 8.8 Hz, 1H, H-5); ^{13}C NMR (125 MHz, $CDCl_3$) δ 163.41, 161.82, 153.33, 150.09, 138.42, 132.45, 119.82, 118.97, 116.01; HRMS (ESI) *m/z* calcd. for $C_{10}H_5BrO_4$ $[M+H]^+$ 268.9444, found 270.9428.

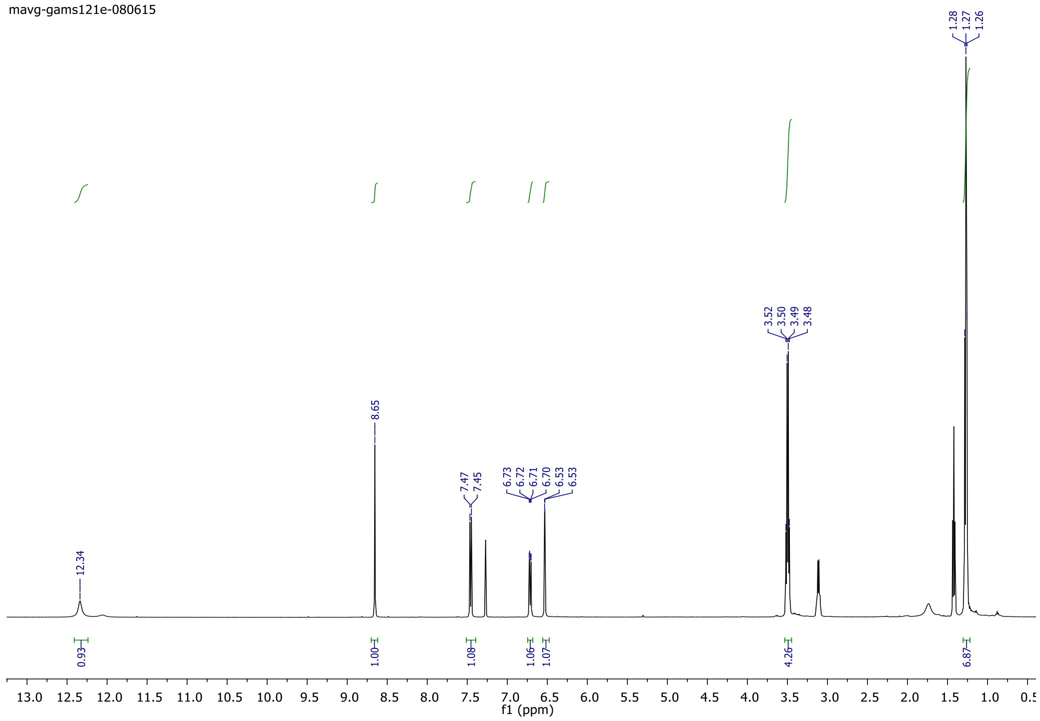

^{1}H spectrum of **3e** in $CDCl_3$, 500 MHz

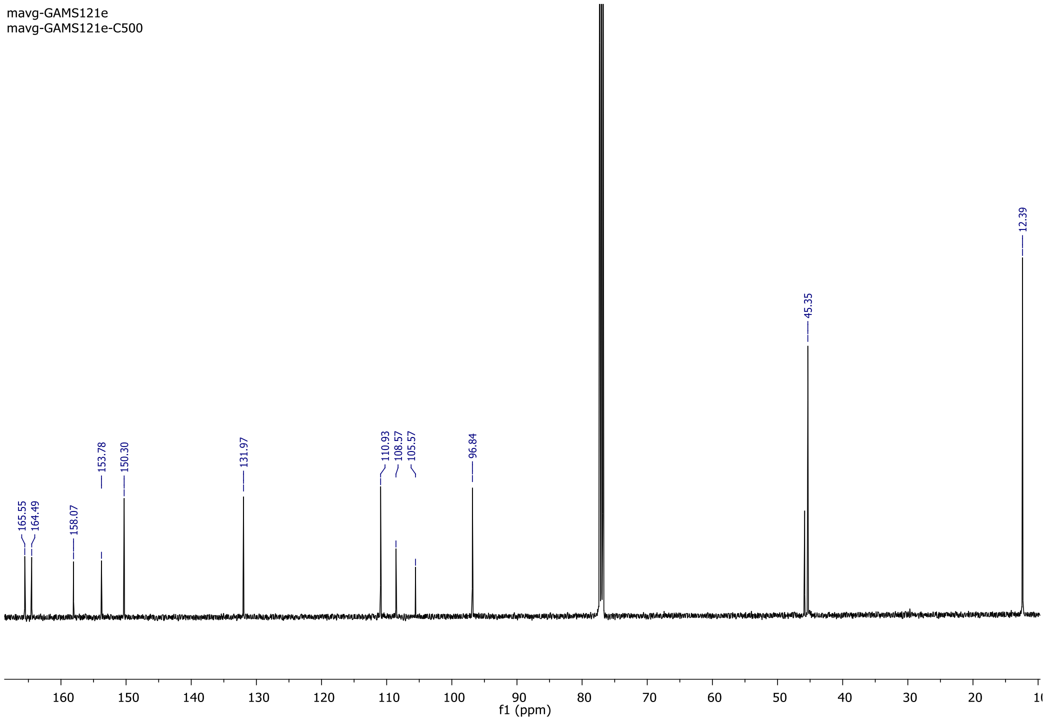

^{13}C spectrum of **3e** in $CDCl_3$, 125 MHz

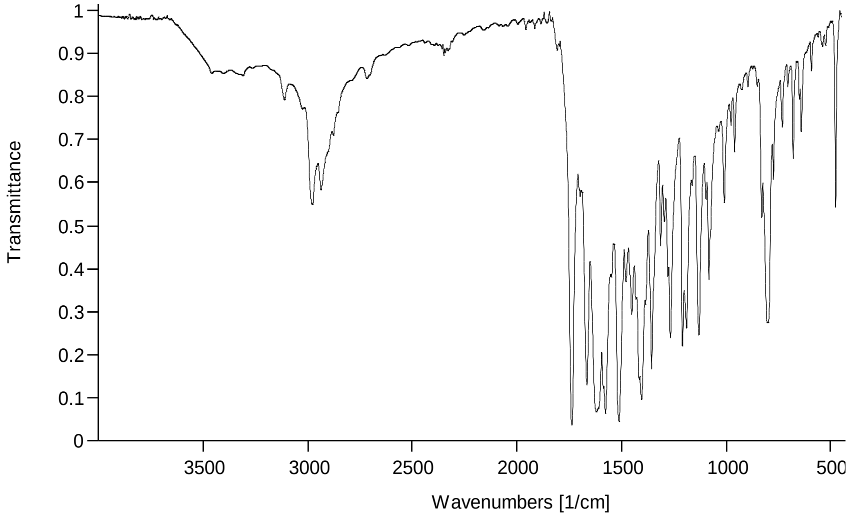

IR spectrum of **3e** in KBr

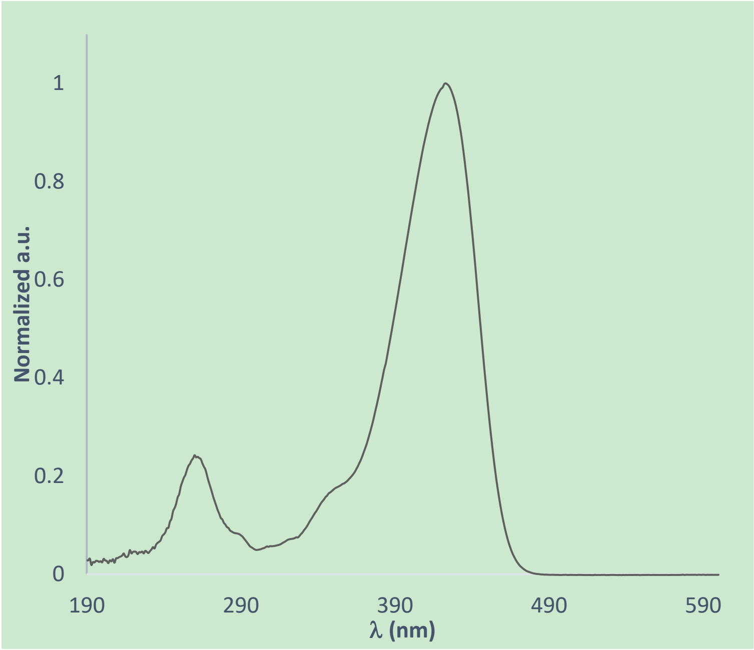

UV spectrum of **3e** in MeOH

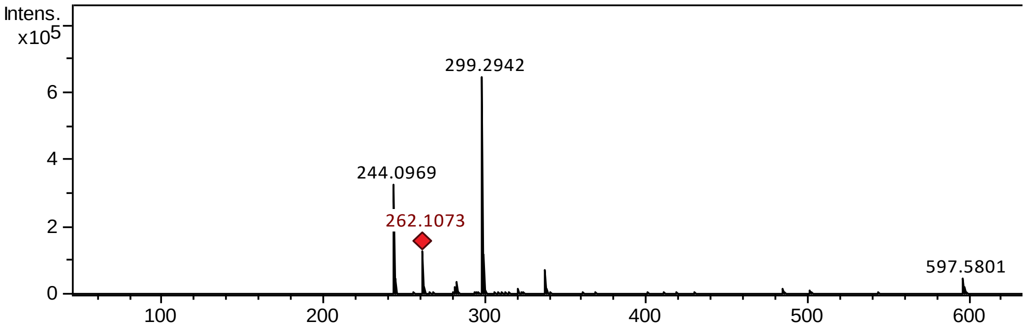

Mass spectrum, ESI(+), of **3e**

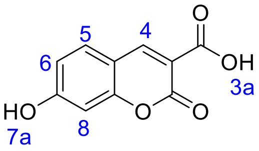

(3f) Brownish solid (90% yield); mp 263-264 ºC; IR (KBr) $\nu_{max/cm-1}$= 3126, 1712, 1683, 1619; UV-vis (MeOH) $\lambda_{max/nm}$= 351; ^{1}H NMR (500 MHz, DMSO-d_6) δ 11.87 (s, 1H,H-7a), 8.64 (s, 1H,H-4), 7.70 (d, *J*= 8.6 Hz, 1H, H-5), 6.82 (dd, *J*= 8.6, 2.1 Hz, 1H, H-6), 6.70 (d, *J*= 2.0 Hz, 1H, H-8); ^{13}C NMR (125 MHz, DMSO-d_6) δ 164.66, 164.39, 158.12, 157.42, 149.87, 132.44, 114.46, 112.83, 111.06, 102.24; HRMS (ESI) m/z calcd. for $C_{10}H_6O_5$ $[M+H]^+$ 207.0288, found 207.0291.

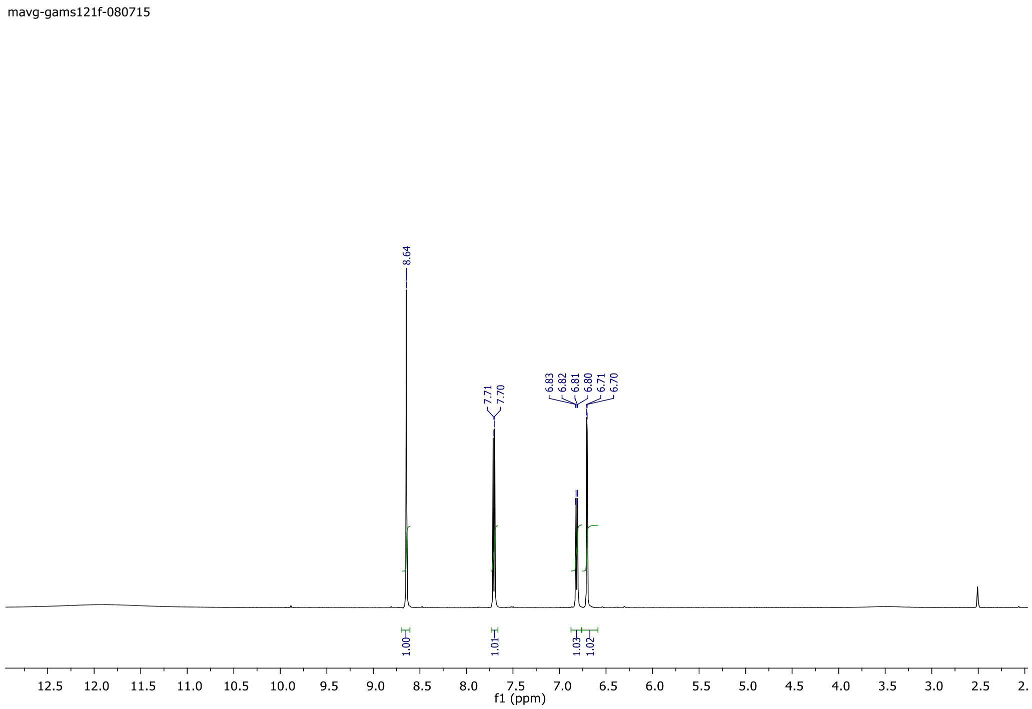

^{1}H spectrum of **3f** in DMSO-d_6, 500 MHz

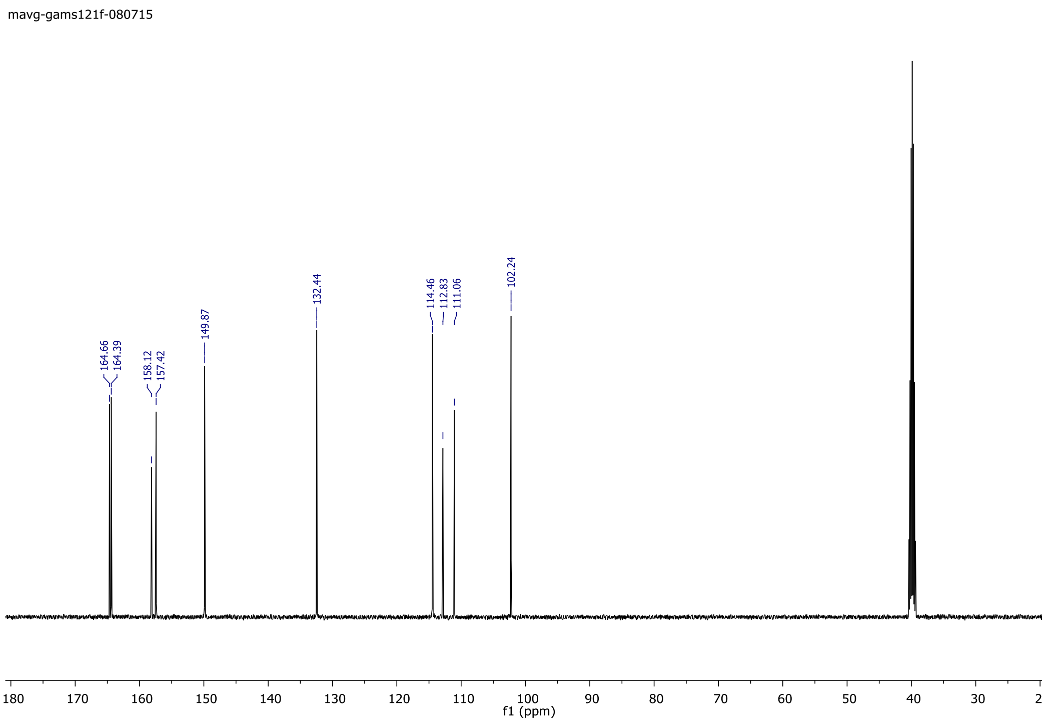

^{13}C spectrum of **3f** in DMSO-d$_6$, 125 MHz

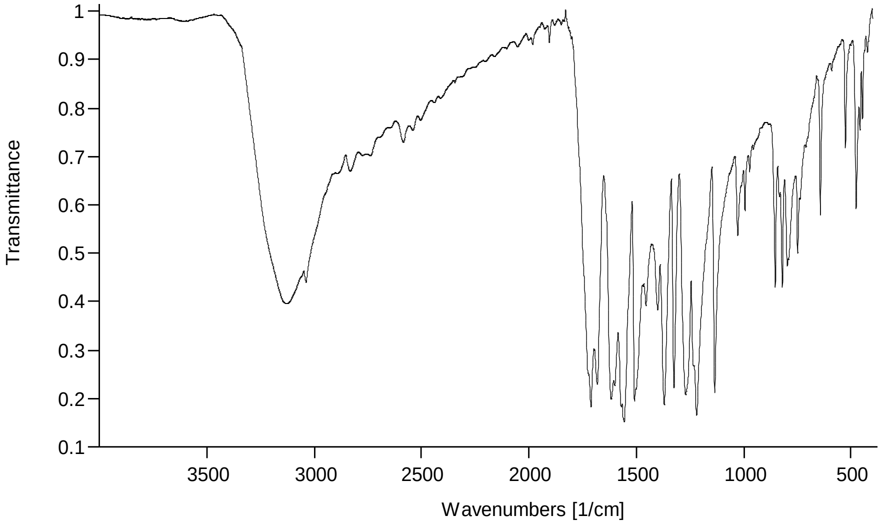

IR spectrum of **3f** in KBr

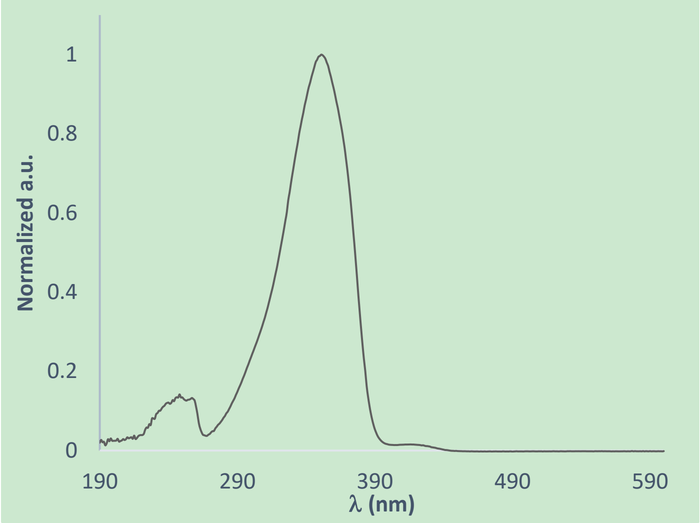

UV spectrum of **3f** in MeOH

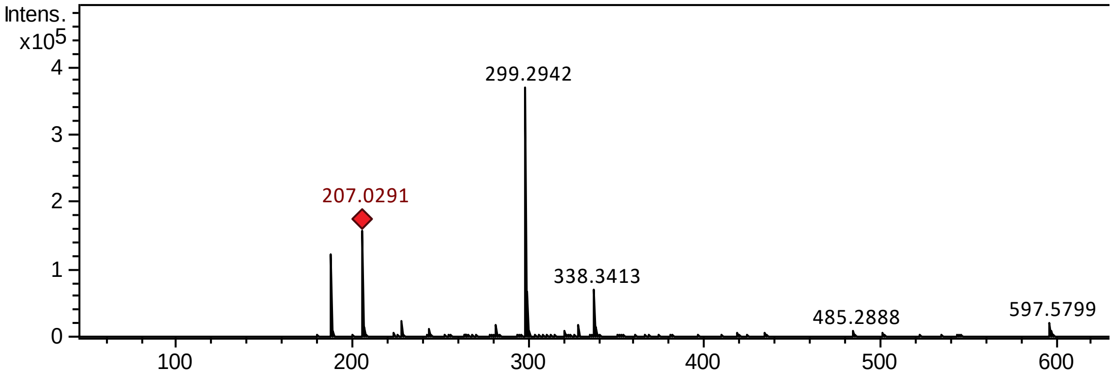

Mass spectrum, ESI(+), of **3f**

General procedure for the preparation of coumarin-3-carboxamides (5a-k)

In a dry round-bottom flask purged with N_2, the corresponding coumarin (1 mmol), 4-(dimethylamino)pyridine (DMAP) (5% mol), and carbonyldiimidazole (CDI) (1.1 mmol) were dissolved in DMF. After 30 min of stirring at room temperature, hydrochloride amine salt was added and stirring continued overnight. Water was added when the reaction was completed. The resulting precipitate was obtained by filtration and purified by recrystallization with MeOH and cold water.

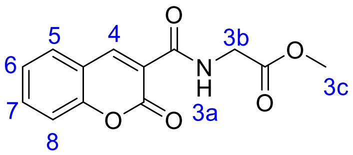

(5a) White solid (84% yield); mp 181-183 °C; IR(KBr) $\nu_{max/cm-1}$= 3328, 3054, 1749, 1710, 1655; UV-vis (MeOH) $\lambda_{max/nm}$= 300; ^{1}H NMR (500 MHz, $CDCl_3$) δ 9.25 (s, 1H, H-3a), 8.91 (s, 1H, H-4), 7.77-7.56 (m, 2H, H-5,7), 7.50-7.31 (m, 2H, H-6,8), 4.26 (d, *J*= 5.6 Hz, 2H, H-3b), 3.80 (s, 3H, H-3c); ^{13}C NMR (125 MHz, $CDCl_3$) δ 169.73, 161.91, 161.24, 154.55,

148.77, 134.30, 129.90, 125.35, 118.52, 117.96, 116.72, 52.42, 41.69; HRMS (ESI) *m/z* calcd. for $C_{13}H_{11}NO_5$ $[M+H]^+$ 262.0710, found 262.0718.

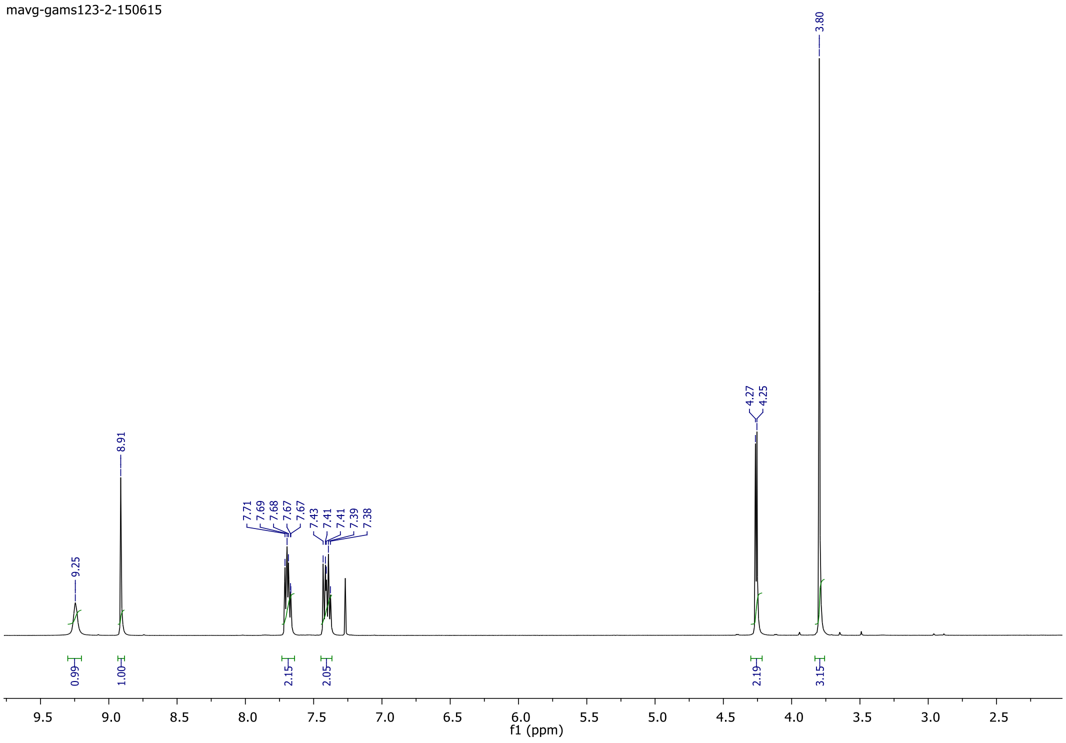

^{1}H spectrum of **5a** in $CDCl_3$, 500 MHz

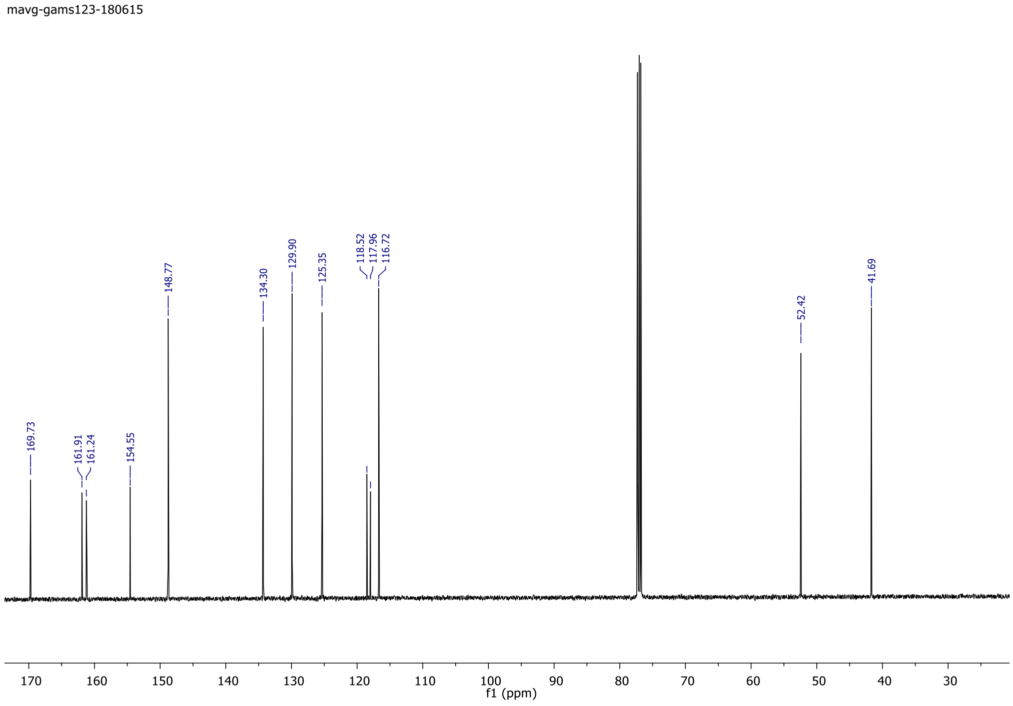

^{13}C spectrum of **5a** in $CDCl_3$, 125 MHz

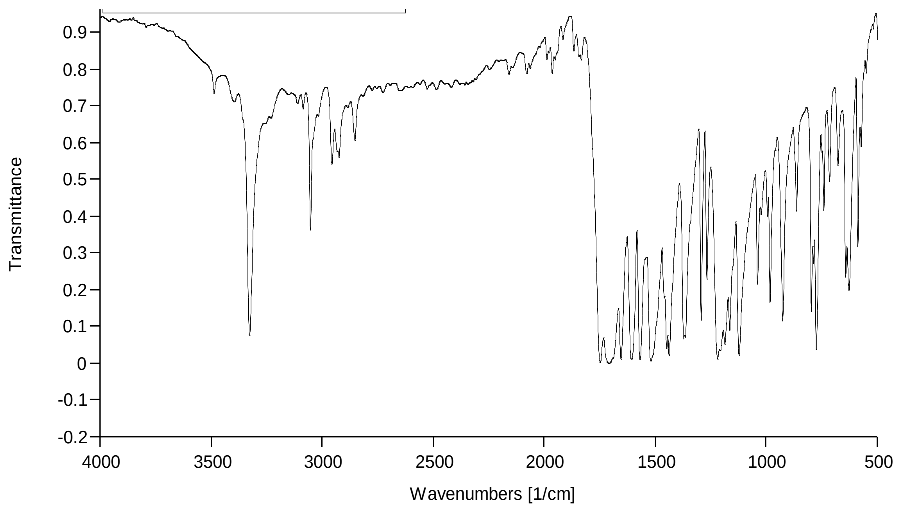

IR spectrum of **5a** in KBr

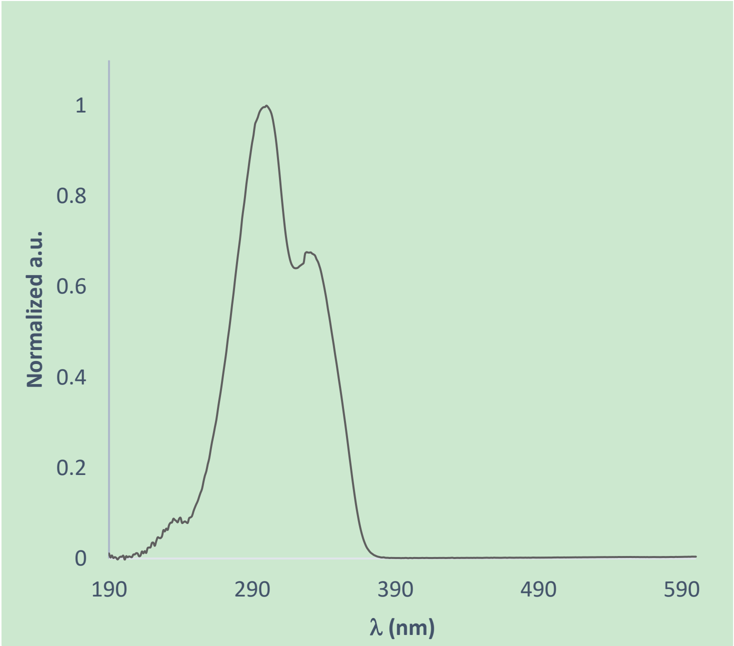

UV spectrum of **5a** in MeOH

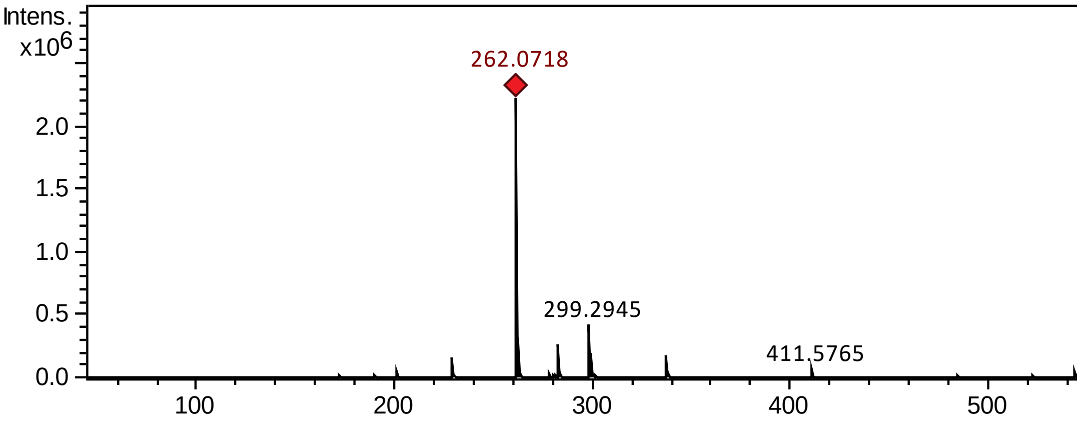

Mass spectrum, ESI(+), of **5a**

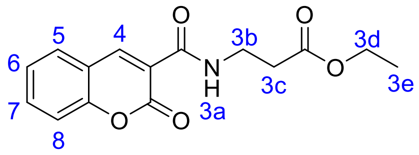

(5b) White solid (60% yield); mp 128-130 ºC; IR (KBr) $\nu_{max/cm-1}$= 3348, 3055, 1726, 1706, 1647, 1612; UV-vis (MeOH) $\lambda_{max/nm}$= 297; ^{1}H NMR (500 MHz, $CDCl_3$) δ 9.14 (s, 1H, H-3a), 8.90 (s, 1H, H-4), 7.81-7.56 (m, 2H, H-6,8), 7.39 (m, 2H, H-5,7), 4.20 (q, *J*= 7.1 Hz, 2H, H-3d), 3.76 (q, *J*= 6.3 Hz, 2H, H-3b), 2.66 (t, *J*= 6.4 Hz, 2H, H-3c), 1.29 (t, *J*= 7.1 Hz, 3H, H-3e); ^{13}C NMR (125 MHz, $CDCl_3$) δ 171.77, 161.59, 161.26, 154.47, 148.36, 134.06, 129.80, 125.27, 118.61, 118.39, 116.65, 60.84, 35.44, 34.21, 14.20; HRMS (ESI) *m/z* calcd. for $C_{15}H_{15}NO_5$ $[M+H]^+$ 290.1023, found 290.1029.

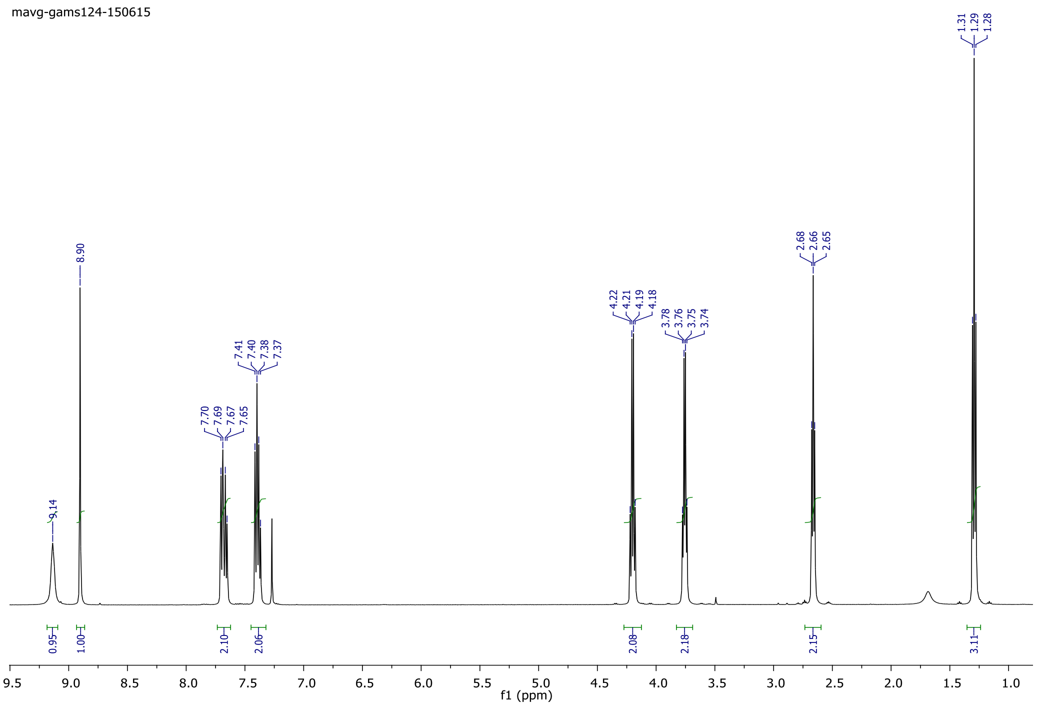

^{1}H spectrum of **5b** in $CDCl_3$, 500 MHz

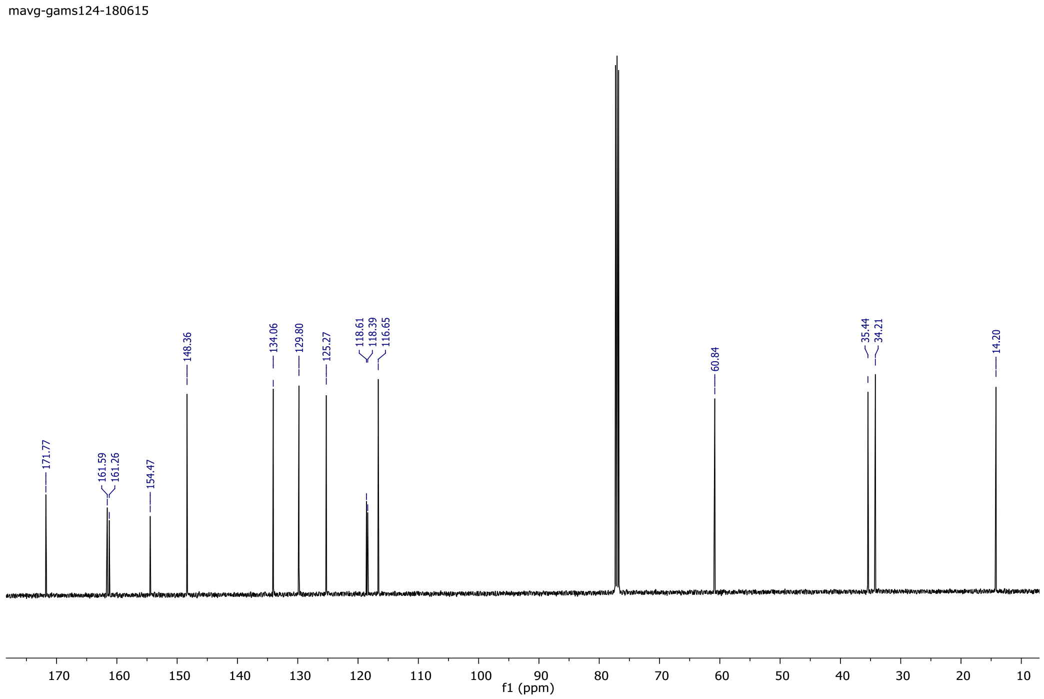

^{13}C spectrum of **5b** in $CDCl_3$, 125 MHz

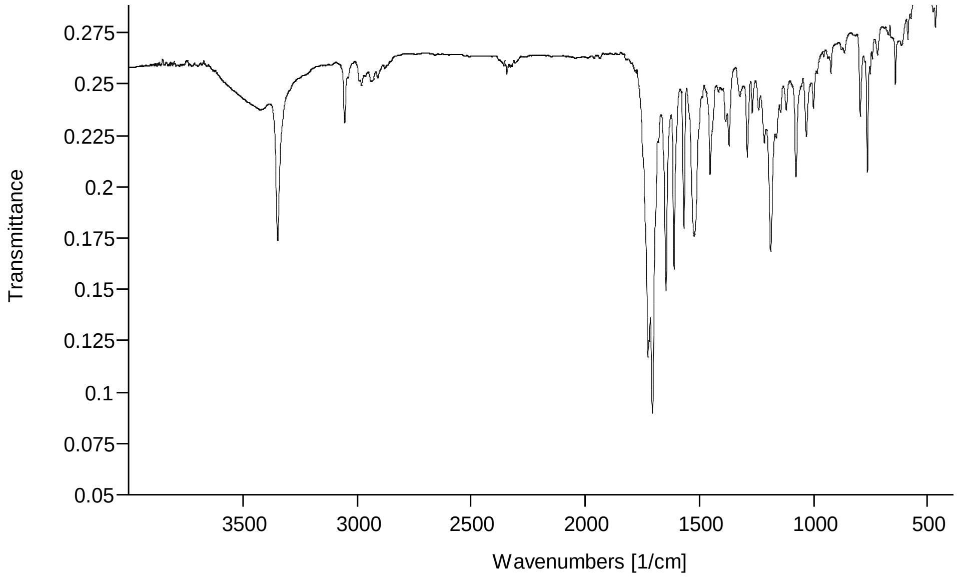

IR spectrum of **5b** in KBr

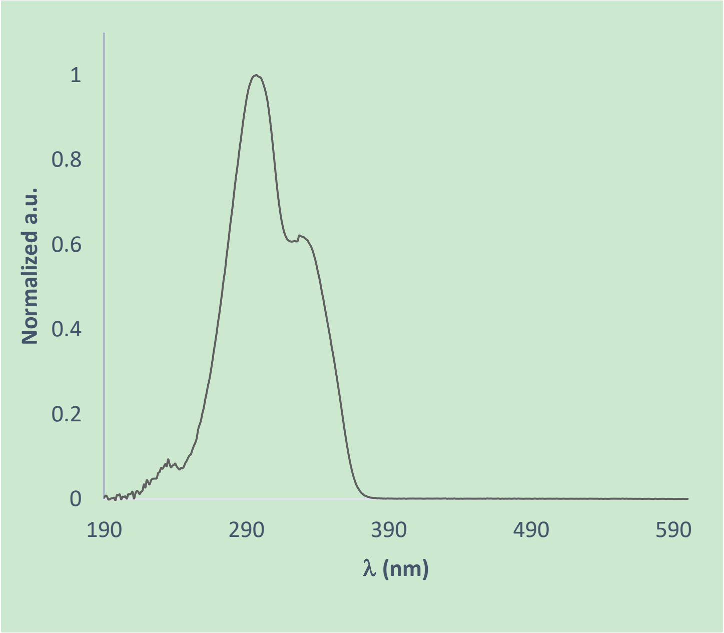

UV spectrum of **5b** in MeOH

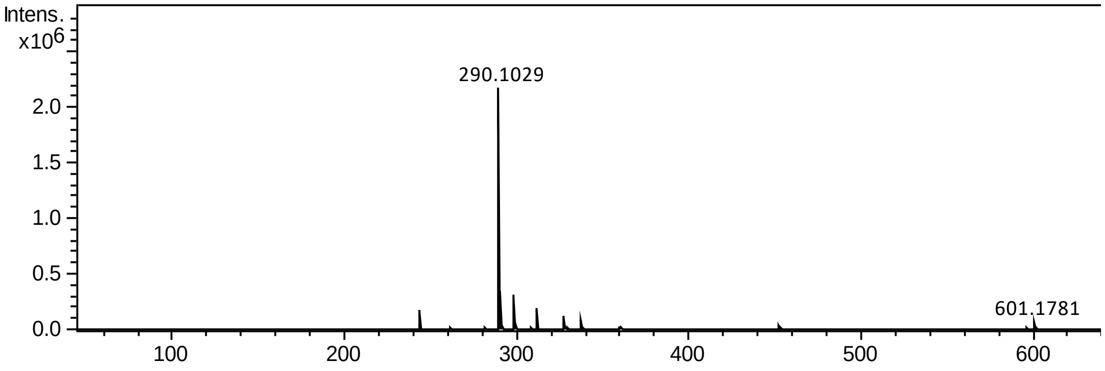

Mass spectrum, ESI(+), of **5b**

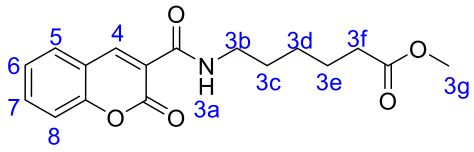

(5c) White solid (85% yield); mp 88-90 ºC; IR (KBr) $\nu_{max/cm-1}$= 3321, 3054, 1742, 1721, 1706, 1607; UV-vis (MeOH) $\lambda_{max/nm}$= 298; ^{1}H NMR (500 MHz, $CDCl_3$) δ8.91 (s, 1H, H-4), 8.83 (s, 1H, H-3a), 7.73 – 7.63 (m, 2H, H-6,8), 7.39 (m, 2H, H-5,7), 3.67 (s, 3H, H-3g), 3.47 (dd, *J*= 13.1, 6.9 Hz, 2H, H-3b), 2.34 (t, *J*=7.5 Hz, 2H, H-3f), 1.68 (tt, *J*= 14.8, 7.4 Hz, 4H, H-3c,3e), 1.49-1.36 (m, 2H, H-3d); ^{13}C NMR (125 MHz, $CDCl_3$) δ 174.02, 161.47, 154.39, 148.26, 133.98, 129.79, 125.28, 118.59, 116.61, 51.51, 39.66, 33.91, 29.09, 26.52, 24.58; HRMS (ESI) *m/z* calcd. for $C_{17}H_{19}NO_5$ $[M+H]^+$ 318.1336, found 318.1345.

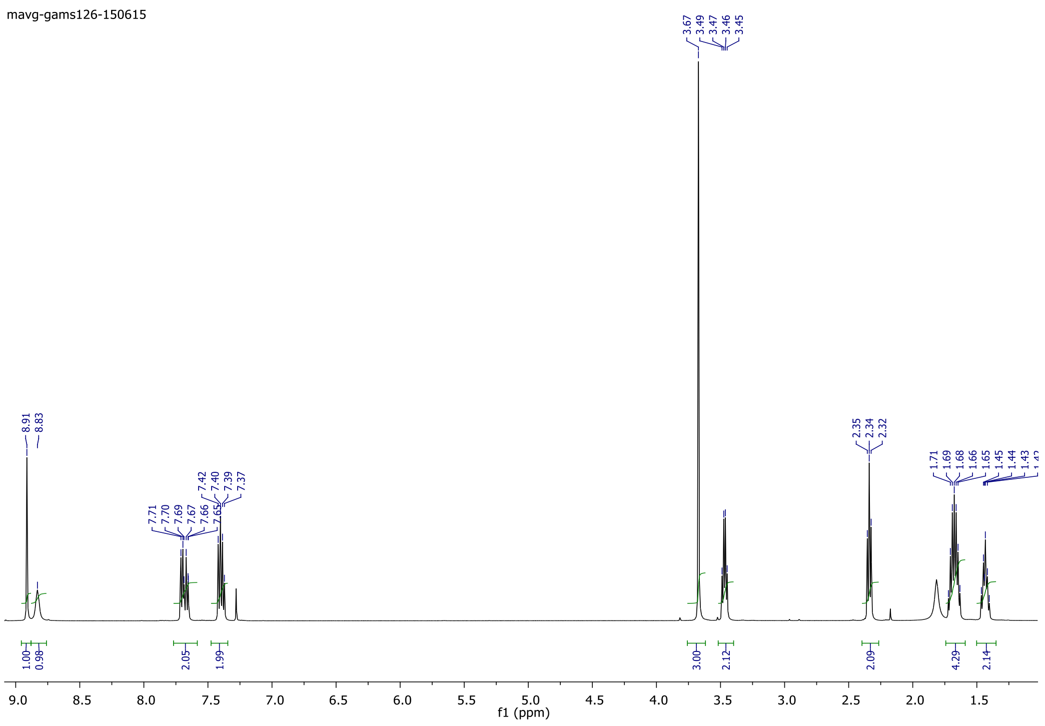

^{1}H spectrum of **5c** in $CDCl_3$, 500 MHz

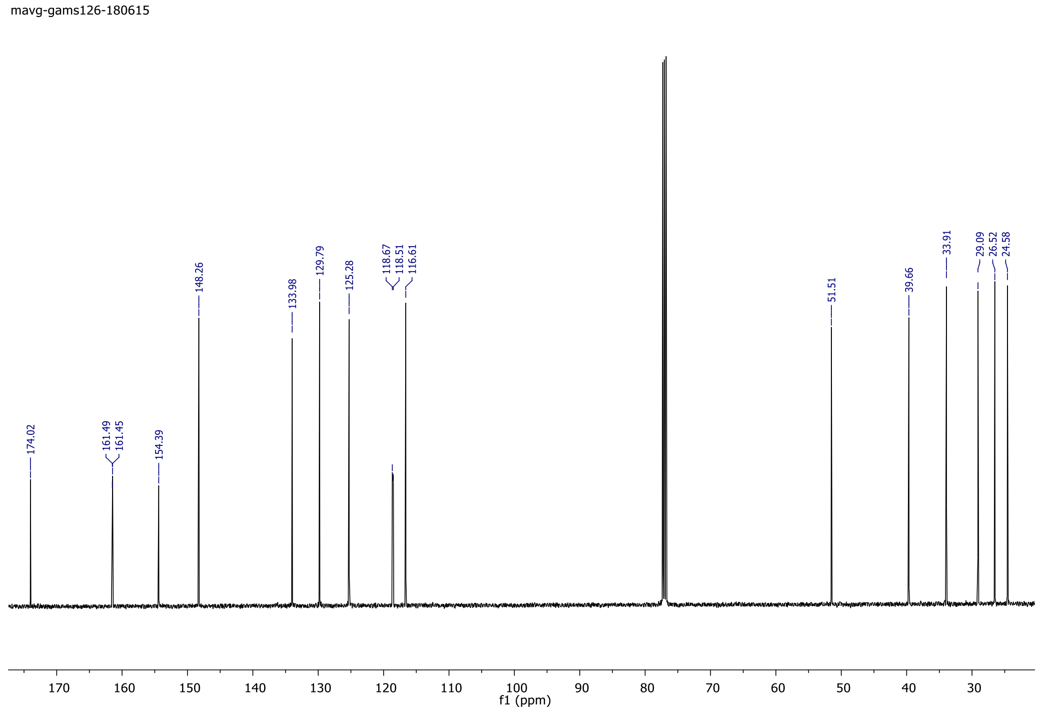

^{13}C spectrum of **5c** in $CDCl_3$, 125 MHz

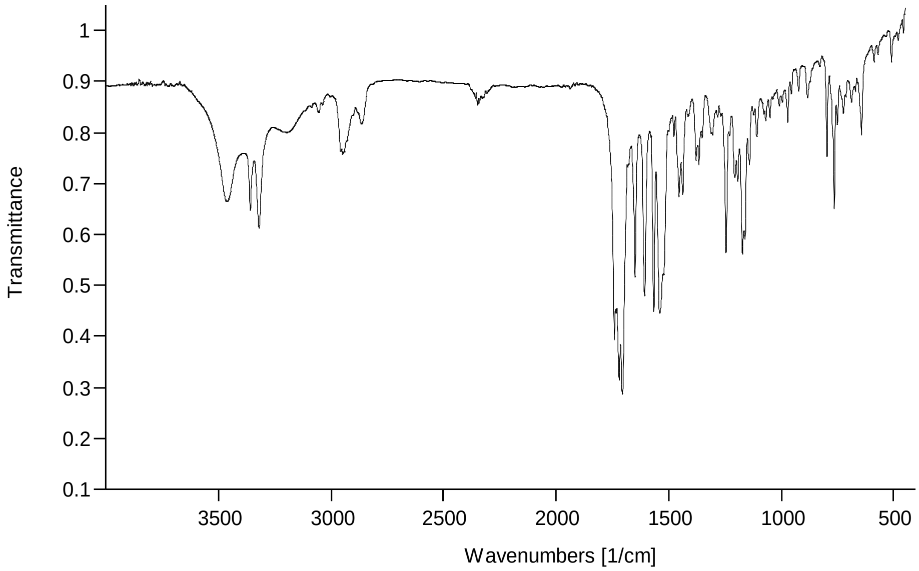

IR spectrum of **5c** in KBr

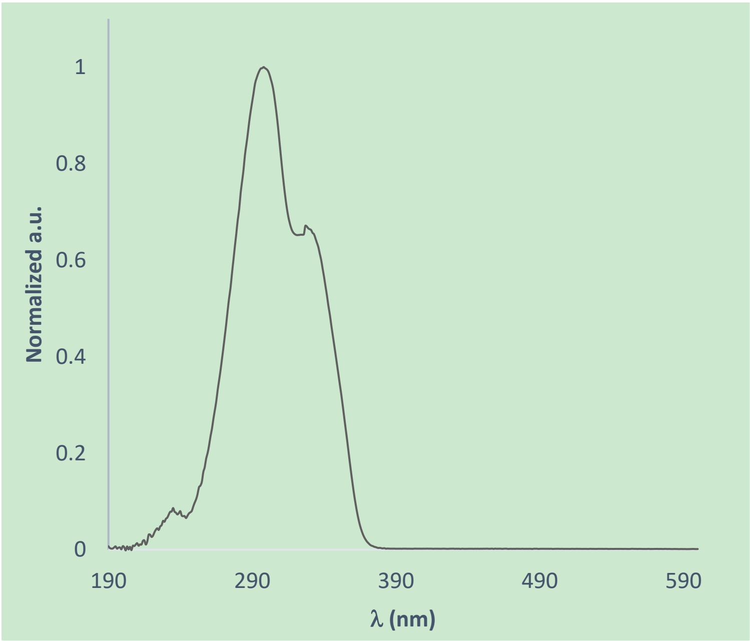

UV spectrum of **5c** in MeOH

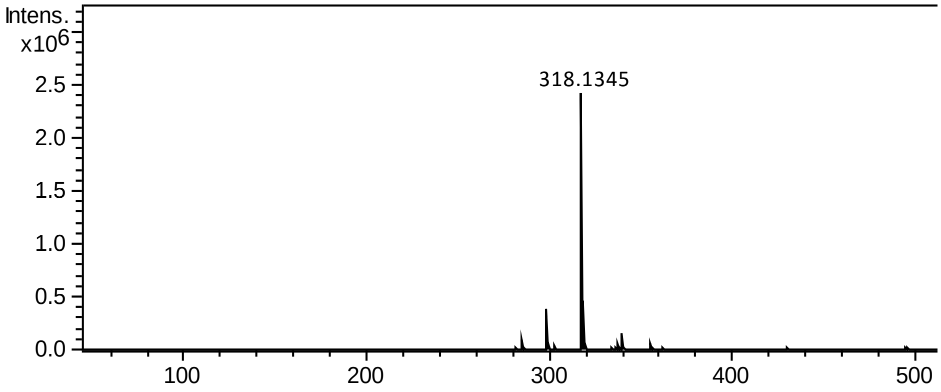

Mass spectrum, ESI(+), of **5c**

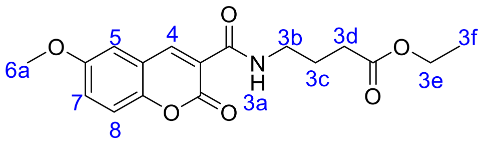

(5d) Yellowish solid (81% yield); mp 131-132 ºC; IR(KBr) $\nu_{max/cm-1}$= 3325, 2981, 2948, 1734, 1698; UV-vis (MeOH) $\lambda_{max/nm}$= 297; ^{1}H NMR (500 MHz, $CDCl_3$) δ 8.92 (s, 1H, H-3a), 8.86 (s, 1H, H-4), 7.34 (d, *J*= 9.1 Hz, 1H, H-8), 7.24 (dd, *J*= 9.1, 2.9 Hz, 1H, H-7), 7.08 (d, *J*= 2.9 Hz, 1H, H-5), 4.15 (q, *J*= 7.1 Hz, 2H, H-3e), 3.88 (s, 3H, H-6a), 3.51 (dd, *J*= 13.2, 6.8 Hz, 2H, H-3b), 2.41 (t, *J*= 7.4 Hz, 2H, H-3d), 1.98 (p, *J*= 7.2 Hz, 2H, H-3c), 1.26 (t, *J*= 7.1 Hz, 3H,H-3f); ^{13}C NMR (125 MHz, $CDCl_3$) δ 172.95, 161.64, 156.61, 149.01, 148.14, 122.57, 118.98, 118.54, 117.73, 110.67, 60.48, 55.92, 39.12, 31.72, 24.79, 14.22; HRMS (ESI) *m/z* calcd. for $C_{17}H_{19}NO_6$ $[M+H]^+$ 334.1285, found 334.1304.

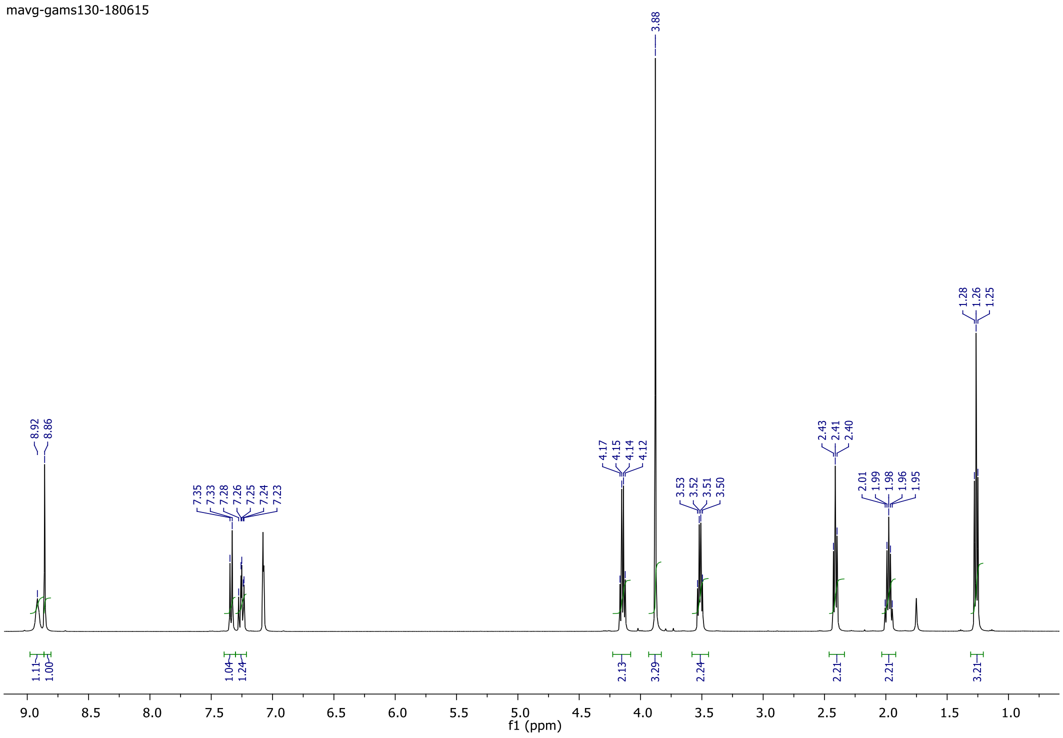

^1H spectrum of **5d** in $CDCl_3$, 500 MHz

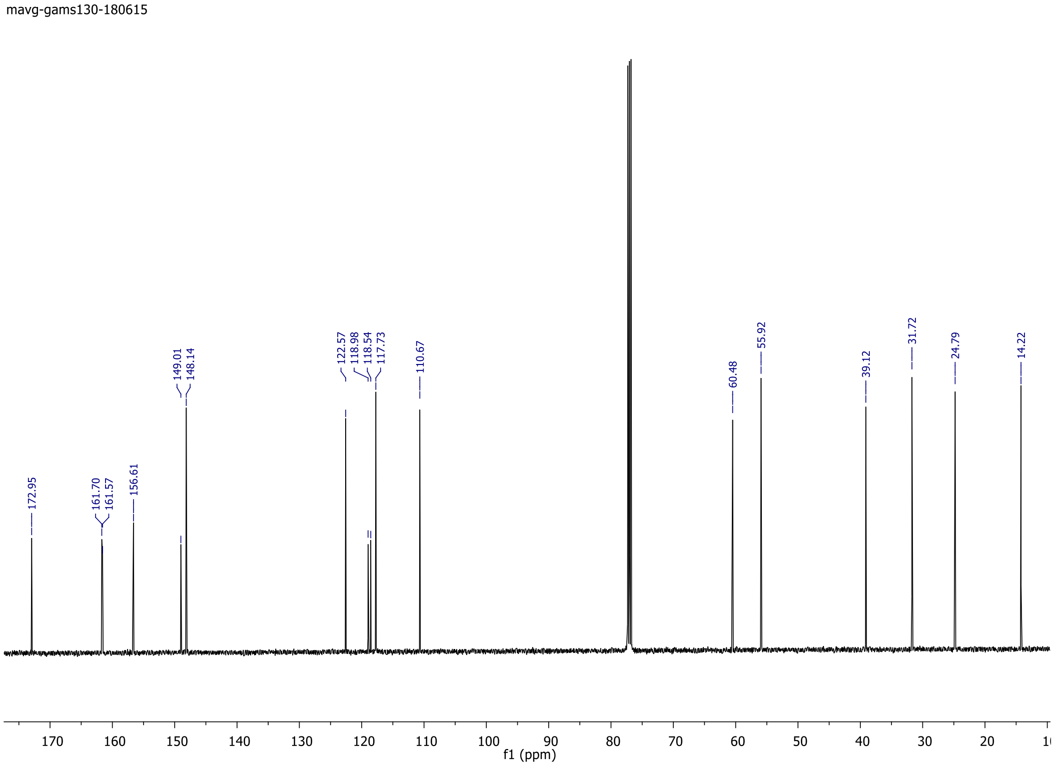

^{13}C spectrum of **5d** in $CDCl_3$, 125 MHz

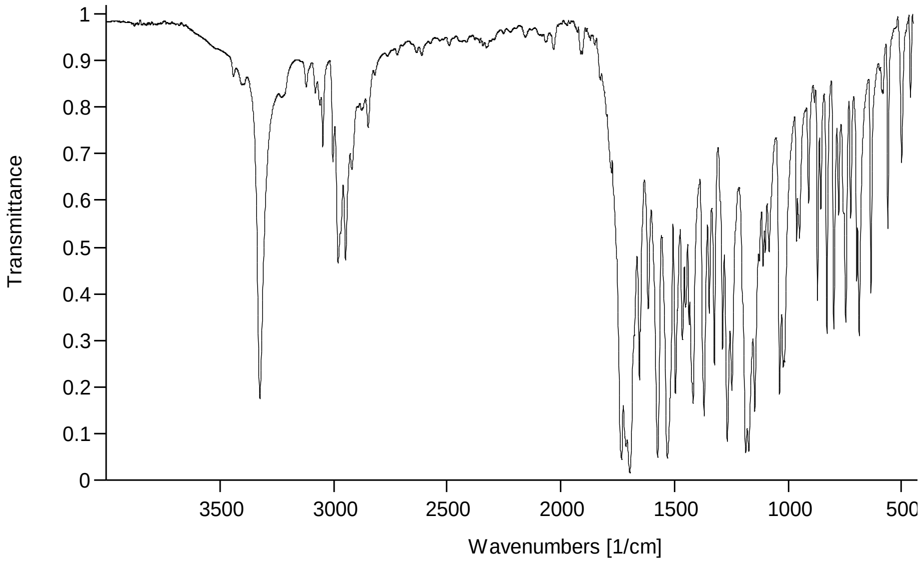

IR spectrum of **5d** in KBr

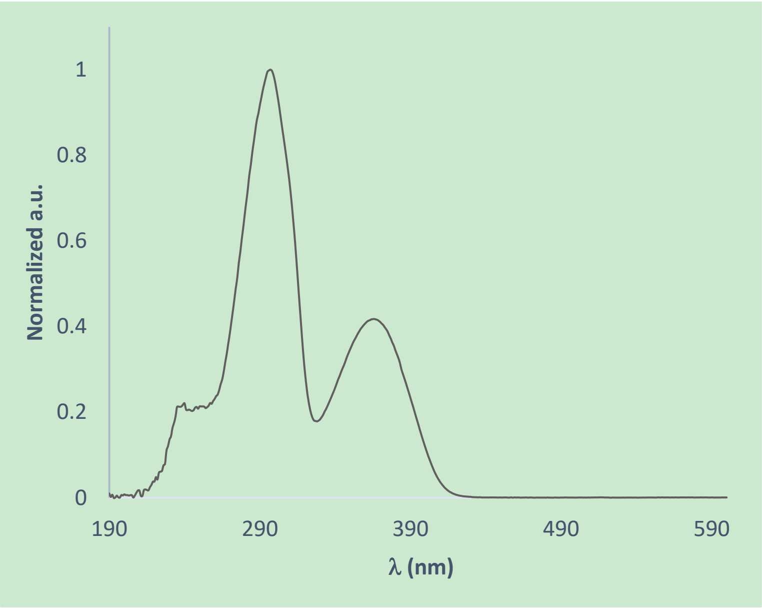

UV spectrum of **5d** in MeOH

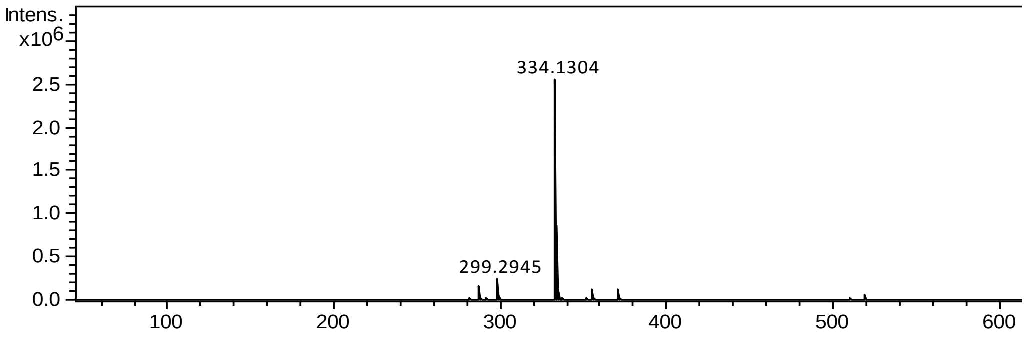

Mass spectrum, ESI(+), of **5d**

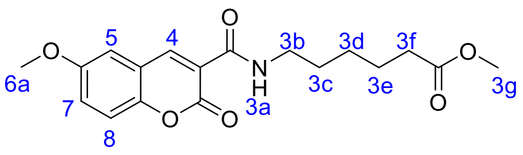

(5e) Pale yellowish solid (90% yield); mp 101-102 ºC; IR(KBr) $\nu_{max/cm-1}$= 3310, 2939, 2859, 1745, 1717, 1697, 1658; UV-vis (MeOH) $\lambda_{max/nm}$= 297; ^{1}H NMR (500 MHz, $CDCl_3$) δ 8.88 (s, 1H, H-3a), 8.86(s, 1H, H-4), 7.33 (d, *J*= 9.1 Hz, 1H, H-8), 7.24 (dd, *J*= 9.1, 2.9 Hz, 1H, H-7), 7.08 (d, *J*= 2.9 Hz, 1H, H-5), 3.88 (s, 3H, H-6a), 3.67 (s, 3H, H-3g), 3.46 (dd, *J*= 13.1, 6.9 Hz, 2H, H-3b), 2.34 (t, *J*= 7.5 Hz, 2H, H-3f), 1.67 (tt, *J*= 15.1, 7.5 Hz, 4H, H-3c,3e), 1.52-1.36 (m, 2H, H-3d); ^{13}C NMR (125 MHz, $CDCl_3$) δ 173.99, 161.55, 156.59, 148.97, 148.02, 122.49, 119.00, 118.63, 117.69, 110.65, 55.91, 51.50, 39.64, 33.90, 29.09, 26.52, 24.58; HRMS (ESI) *m/z* calcd. for $C_{18}H_{21}NO_6$ $[M+H]^+$ 348.1442, found 348.1480.

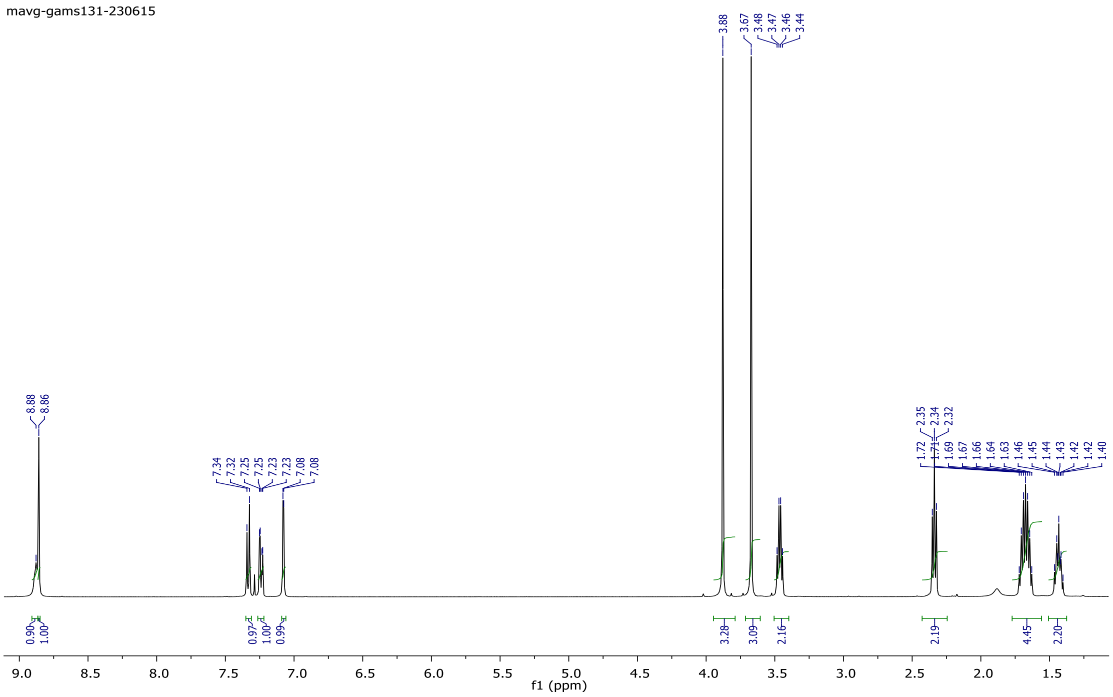

^{1}H spectrum of **5e** in $CDCl_3$, 500 MHz

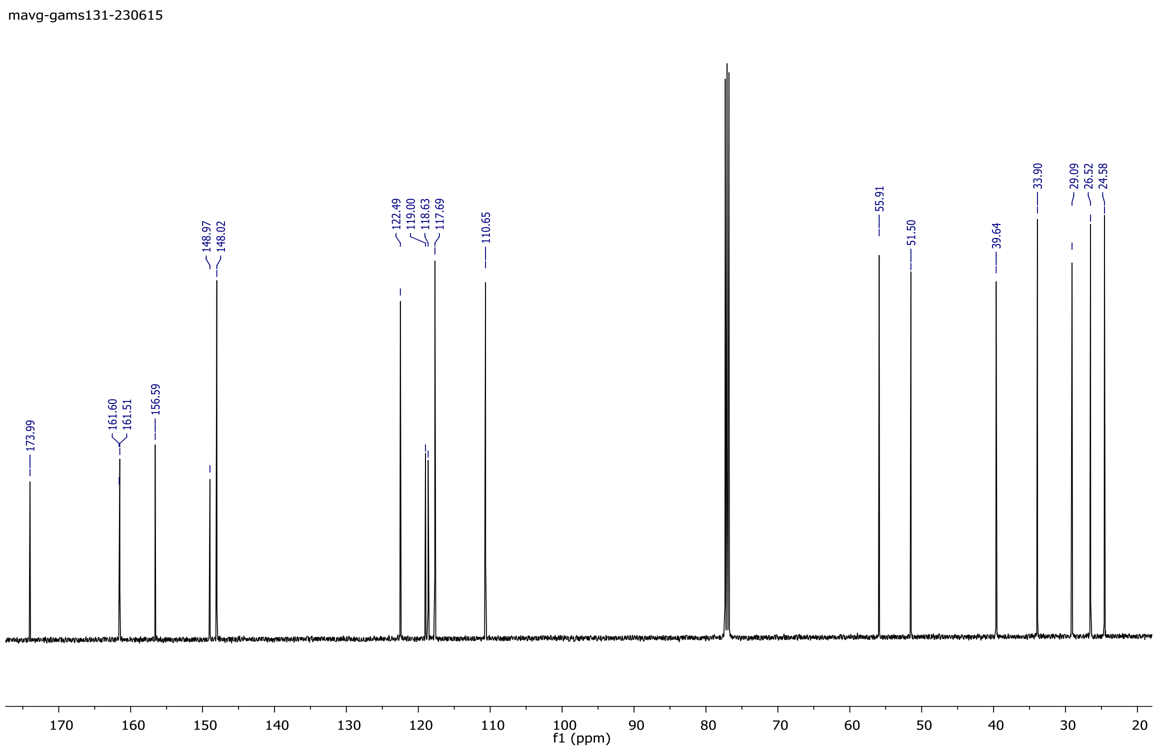

^{13}C spectrum of **5e** in $CDCl_3$, 125 MHz

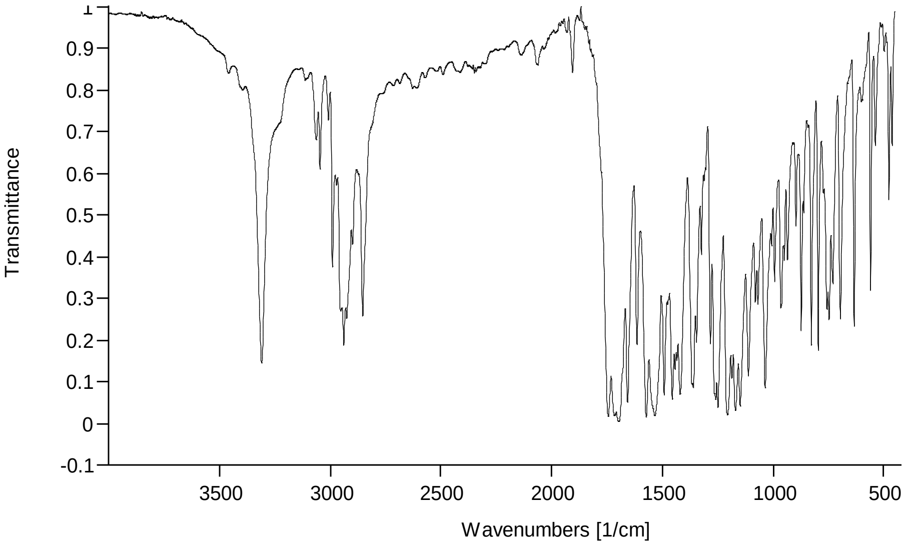

IR spectrum of **5e** in KBr

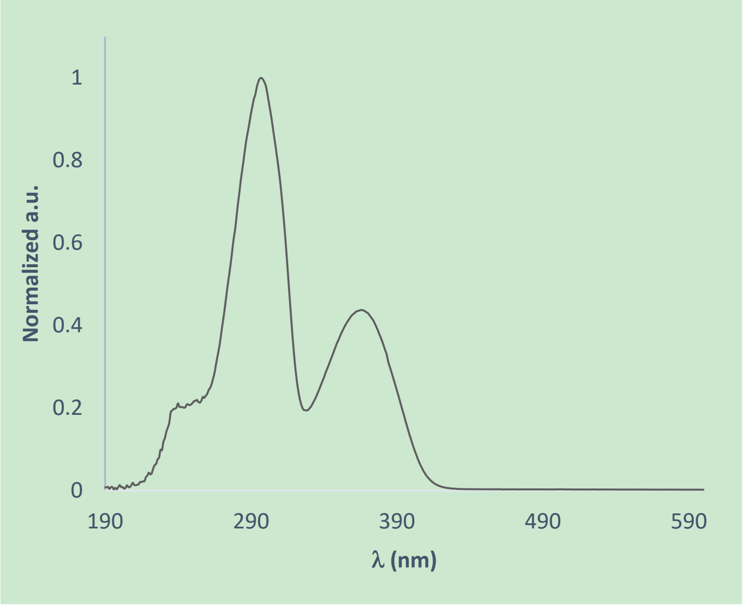

UV spectrum of **5e** in MeOH

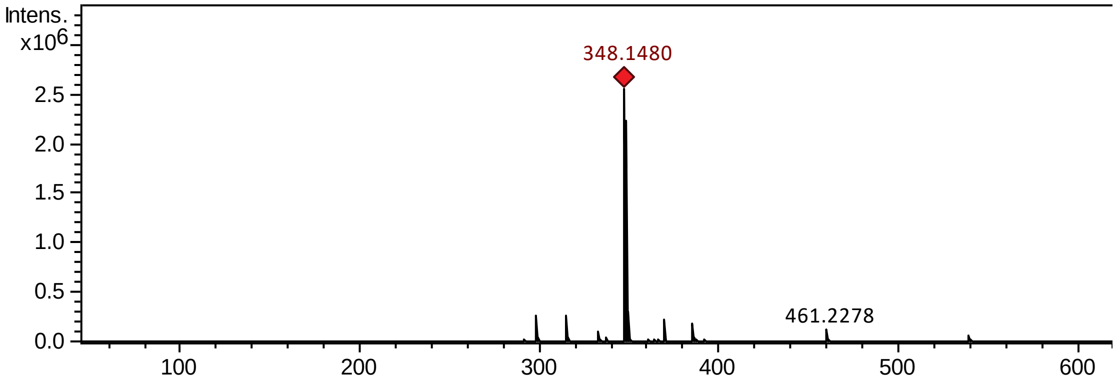

Mass spectrum, ESI(+), of **5e**

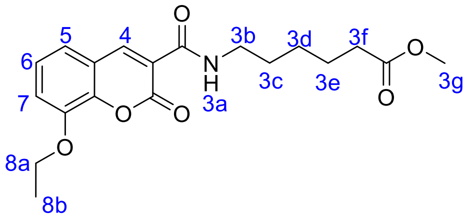

(5f) White solid (68% yield); mp 98-99 ºC; IR(KBr) $\nu_{max/cm-1}$= 3343, 1718, 1655, 1613; UV-vis (MeOH) $\lambda_{max/nm}$= 311; ^{1}H NMR (500 MHz, $CDCl_3$) δ 8.88 (s, 1H, H-4), 8.86 (s, 1H, H-3a), 7.27 (d, *J*= 7.6 Hz, 1H, H-6), 7.25 (t, *J*= 3.9 Hz, 1H, H-7), 7.18 (dd, *J*= 7.7, 1.4 Hz, 1H, H-5), 4.22 (q, *J*= 7.0 Hz, 2H, H-8a), 3.67 (s, 3H, H-3g), 3.46 (dd, *J*= 13.2, 6.9 Hz, 2H, H-3b), 2.34 (t, *J*= 7.5 Hz, 2H, H-3f), 1.67 (tt, *J*= 14.7, 7.5 Hz, 4H,H-3c,3e), 1.52 (t, *J*= 7.0 Hz, 3H, H-8b), 1.47-1.39 (m, 2H, H-3d); ^{13}C NMR (125 MHz, $CDCl_3$) δ 173.96, 161.52, 161.17, 148.46, 146.41, 144.31, 125.07, 120.84,

119.44, 118.62, 116.73, 65.16, 51.45, 39.65, 33.91, 29.09, 26.52, 24.59, 14.68; HRMS (ESI) *m/z* calcd. for $C_{19}H_{23}NO_6$ $[M+H]^+$ 362.1598, found 362.1620.

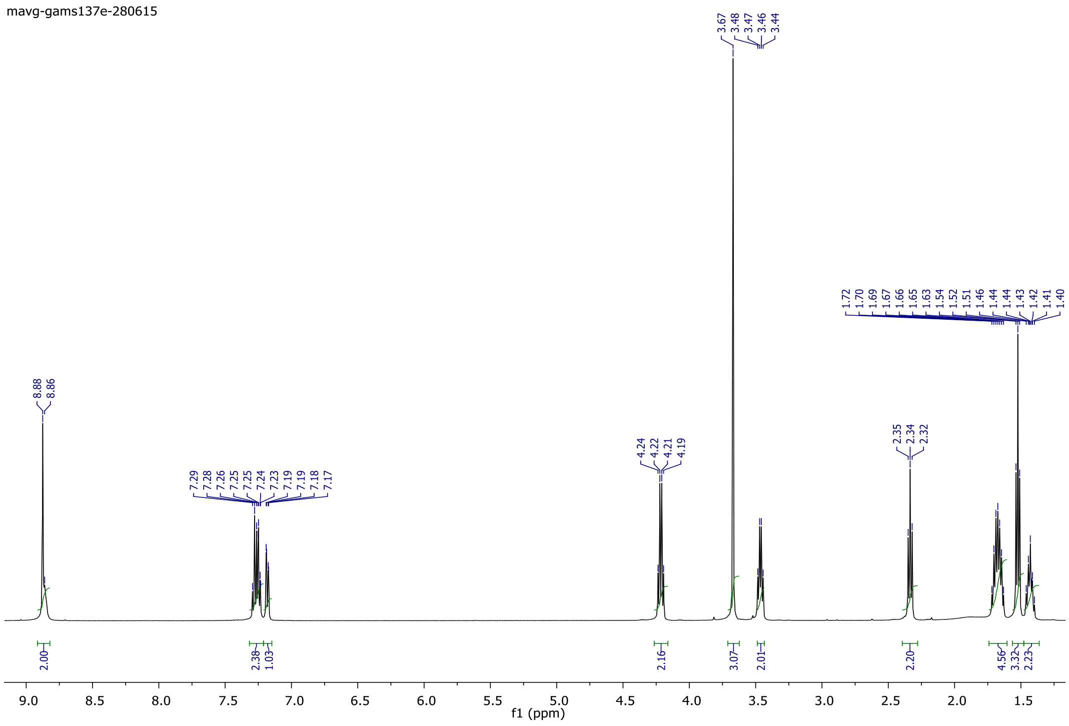

^{1}H spectrum of **5f** in $CDCl_3$, 500 MHz

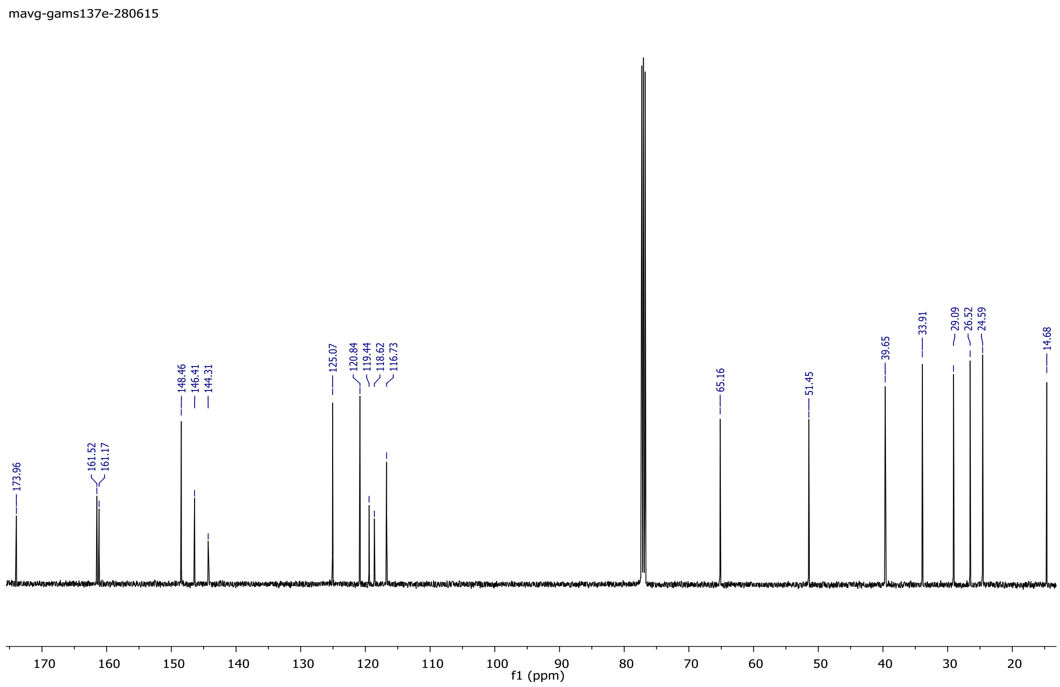

^{13}C spectrum of **5f** in $CDCl_3$, 125 MHz

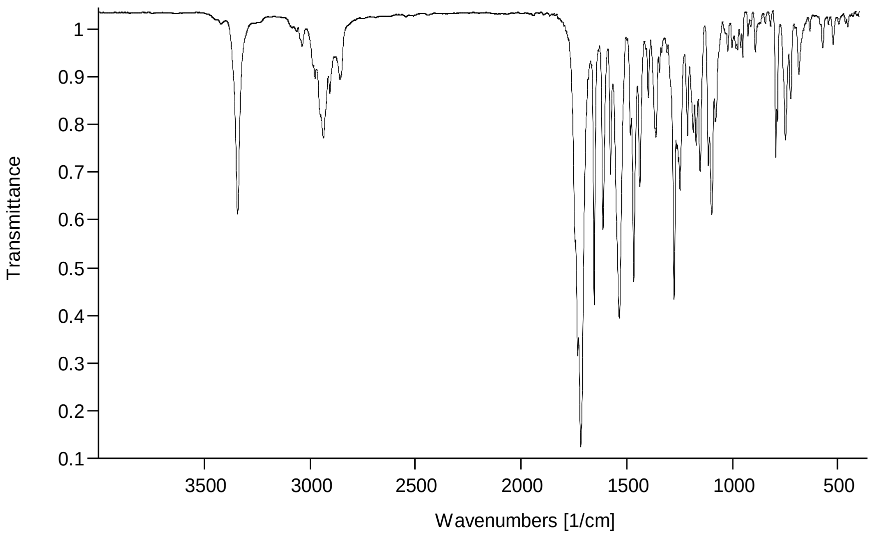

IR spectrum of **5f** in KBr

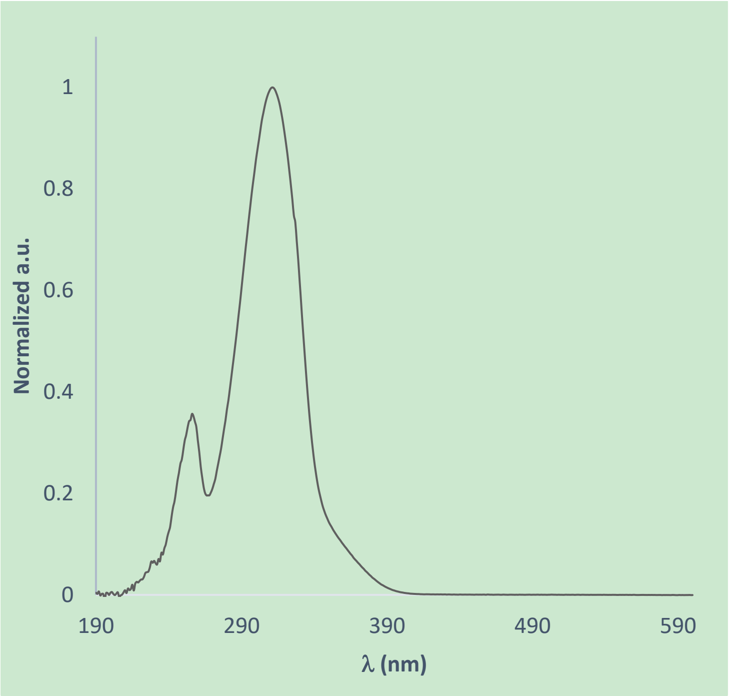

UV spectrum of **5f** in MeOH

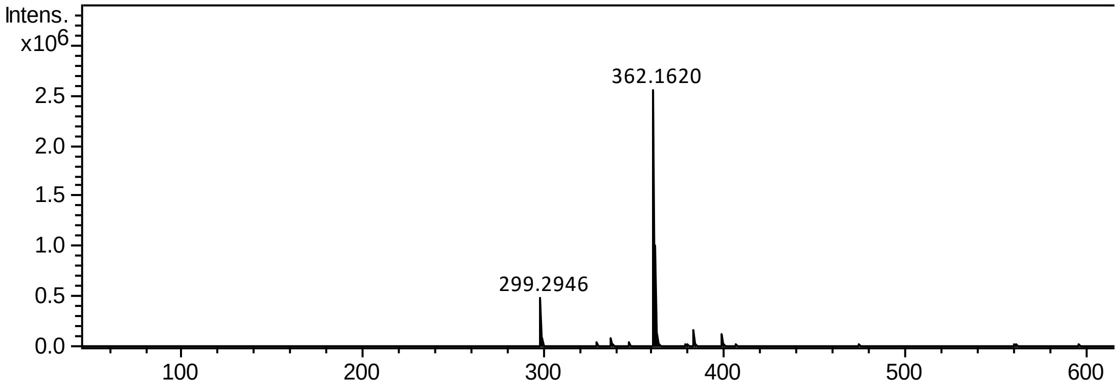

Mass spectrum, ESI(+), of **5f**

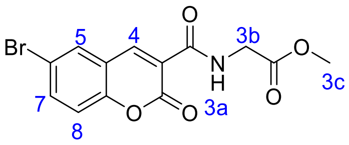

(5g) White solid (46% yield); mp 160-162 ºC; IR(KBr) $\nu_{max/cm-1}$= 3337, 3048, 1740, 1724, 1655; UV-vis (MeOH) $\lambda_{max/nm}$= 290; ^{1}H NMR (500 MHz, $CDCl_3$) δ 9.18 (s, 1H, H-3a), 8.82 (s, 1H, H-4), 7.83 (d, *J*= 2.3 Hz, 1H,H-5), 7.76 (dd, *J*= 8.8, 2.3 Hz, 1H, H-7), 7.31 (d, *J*= 8.8 Hz, 1H, H-8), 4.25 (d, *J*= 5.6 Hz, 2H, H-3b), 3.80 (s, 3H, H-3c); ^{13}C NMR (125 MHz, $CDCl_3$) δ 169.63, 161.39, 160.59, 153.32, 147.40, 137.00, 131.92, 119.96, 119.00, 118.45, 118.01, 52.48, 41.73; HRMS (ESI) *m/z* calcd. for $C_{13}H_{10}NO_5$ $[M+H]^+$ 339.9742, found 339.9802.

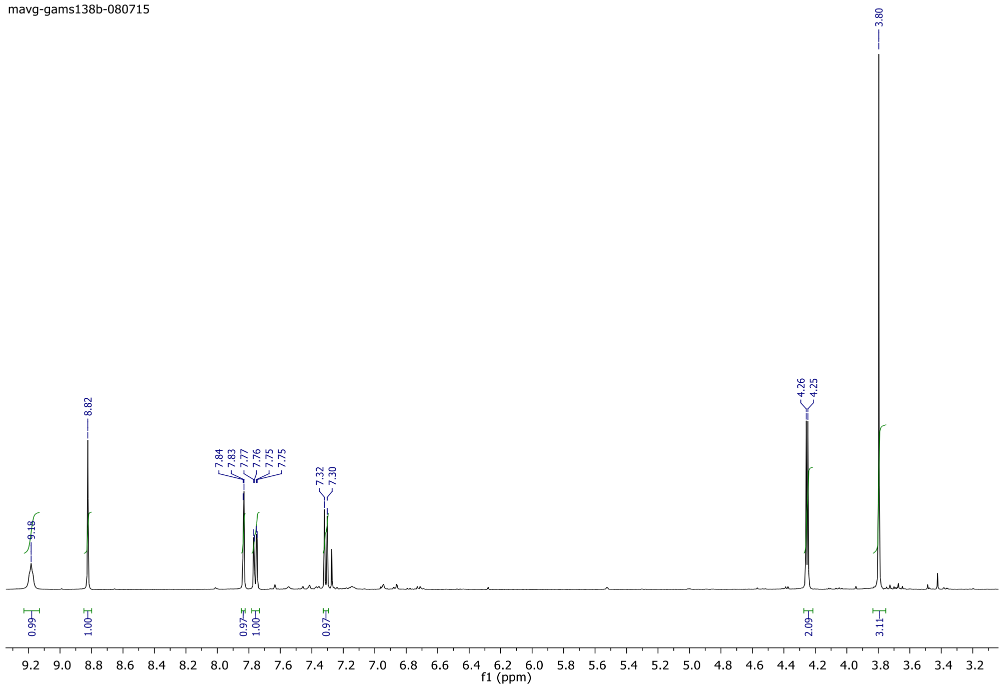

^{1}H spectrum of **5g** in $CDCl_3$, 500 MHz

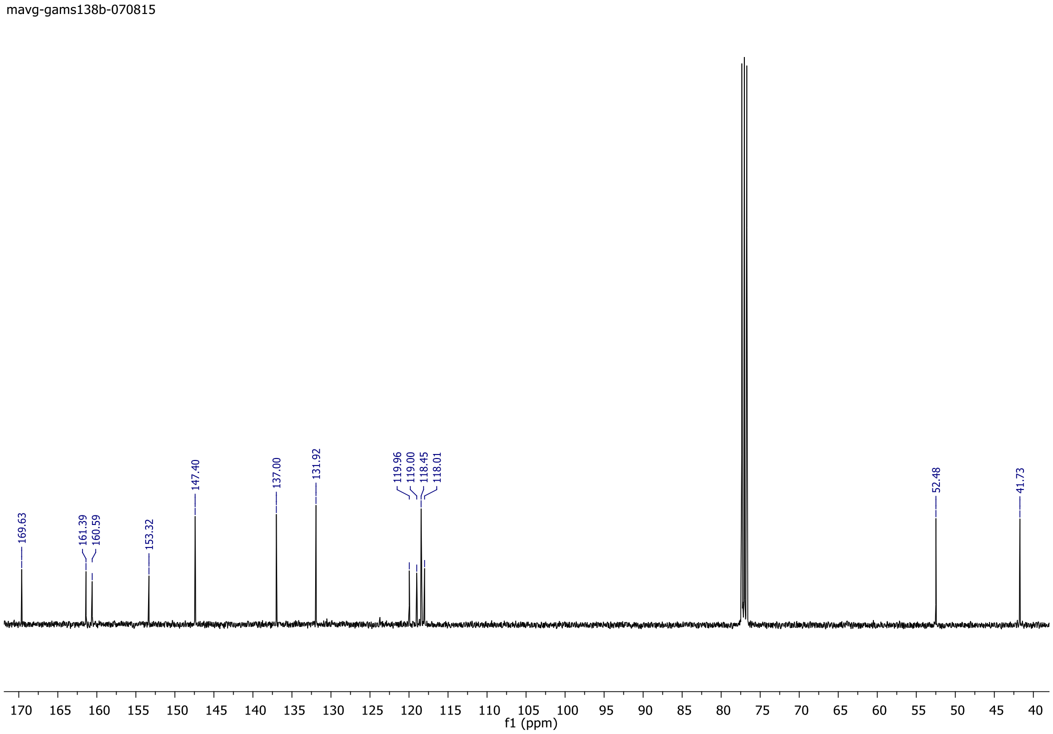

^{13}C spectrum of **5g** in $CDCl_3$, 125 MHz

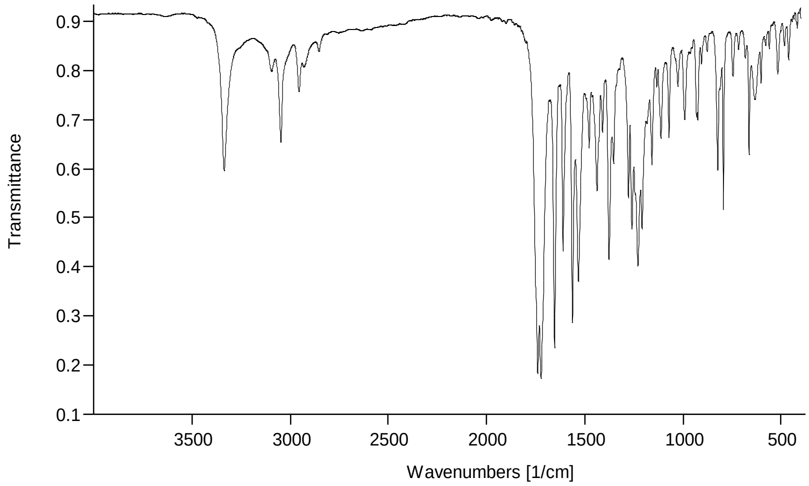

IR spectrum of **5g** in KBr

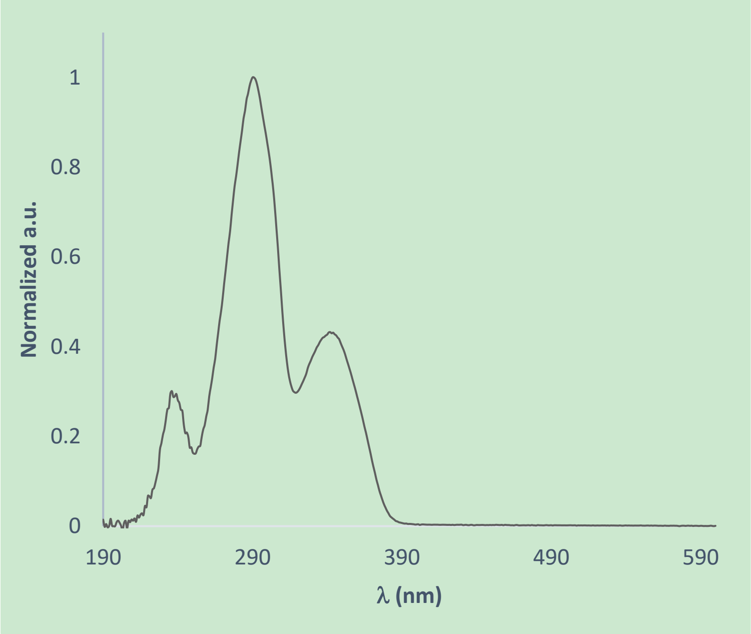

UV spectrum of **5g** in MeOH

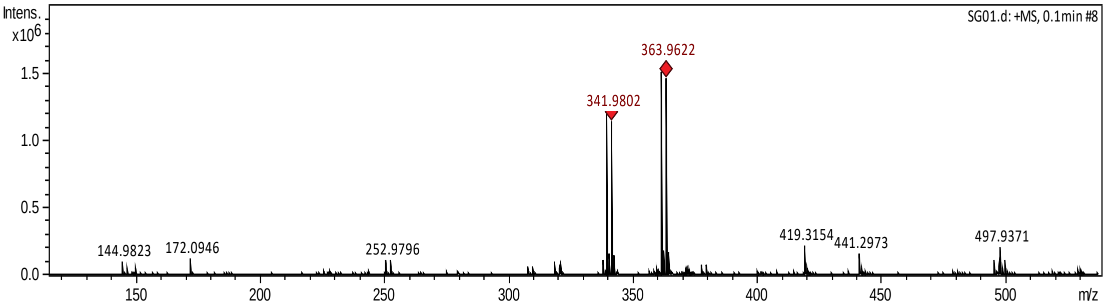

Mass spectrum, ESI(+), of **5g**

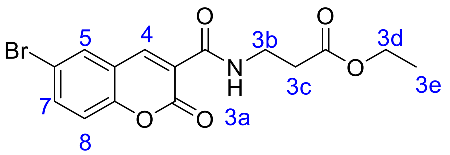

(5h) White solid (58% yield); mp 148-149 ºC; IR(KBr) $\nu_{max/cm-1}$= 3383, 3053, 1734, 1717, 1657; UV-vis (MeOH) $\lambda_{max/nm}$=292; ^{1}H NMR (500 MHz, CDCl$_3$) δ 9.09 (s, 1H, H-3a), 8.82 (s, 1H, H-4), 7.83 (d, *J*= 2.0 Hz, 1H, H-5), 7.74 (dd, *J*= 8.8, 2.1 Hz, 1H, H-7), 7.29 (d, *J*= 8.8 Hz, 1H, H-8), 4.20 (q, *J*= 7.1 Hz, 2H, H-3d), 3.75 (q, *J*= 6.2 Hz, 2H, H-3b), 2.66 (t, *J*= 6.3 Hz, 2H, H-3c), 1.29 (t, *J*= 7.1 Hz, 3H, H-3e); ^{13}C NMR (125 MHz, CDCl$_3$) δ 171.76, 161.06, 160.58, 153.22, 147.00, 136.74, 131.84, 120.07, 119.43, 118.36, 117.90, 60.87, 35.49, 34.11, 14.19; HRMS (ESI) *m/z* calcd. for $C_{15}H_{14}BrNO_5$ $[M+H]^+$ 368.0128, found 368.0132

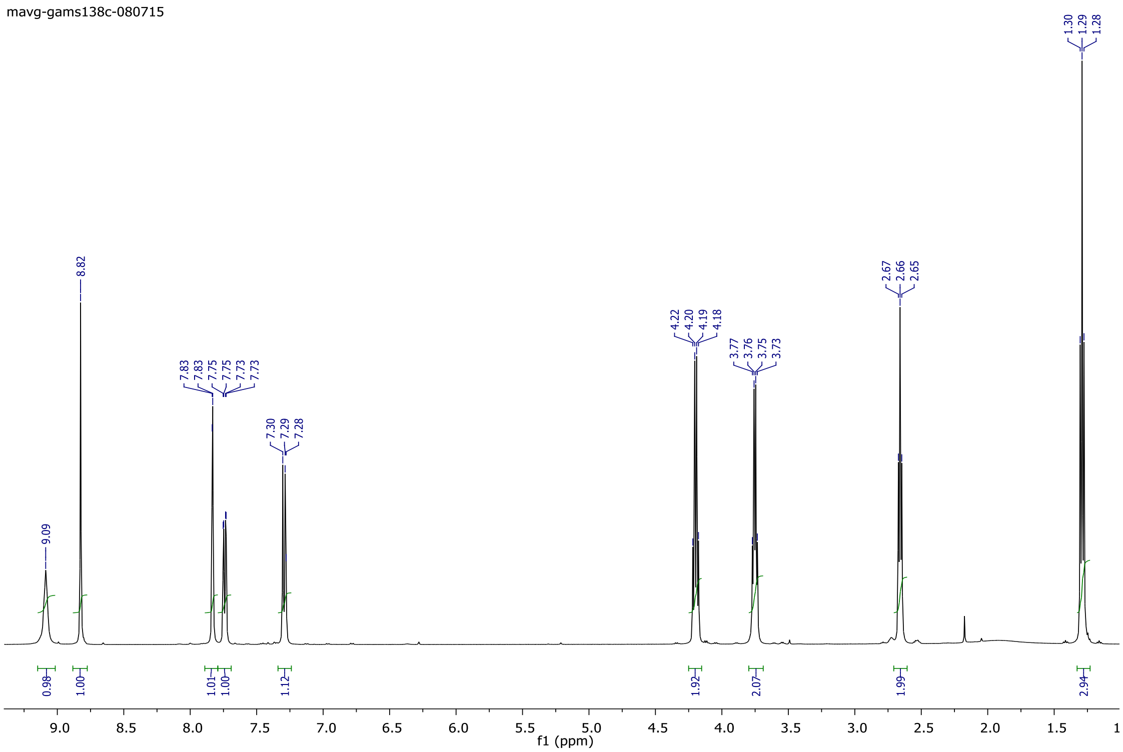

^{1}H spectrum of **5h** in CDCl$_3$, 500 MHz

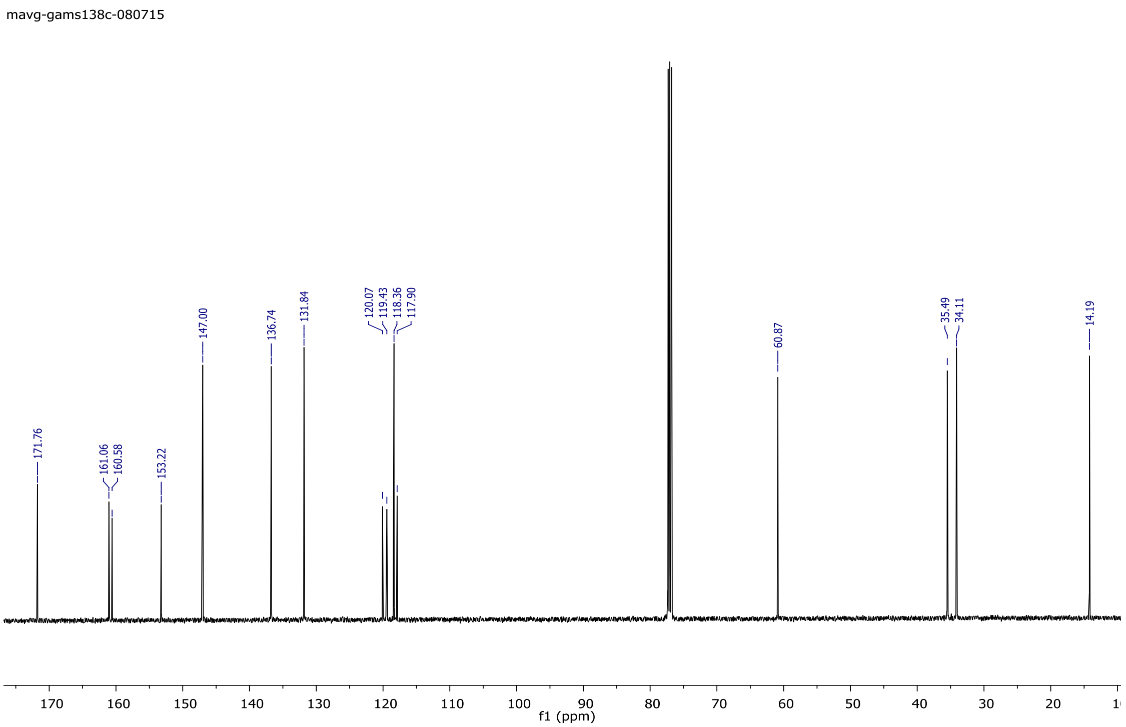

^{13}C spectrum of **5h** in $CDCl_3$, 500 MHz

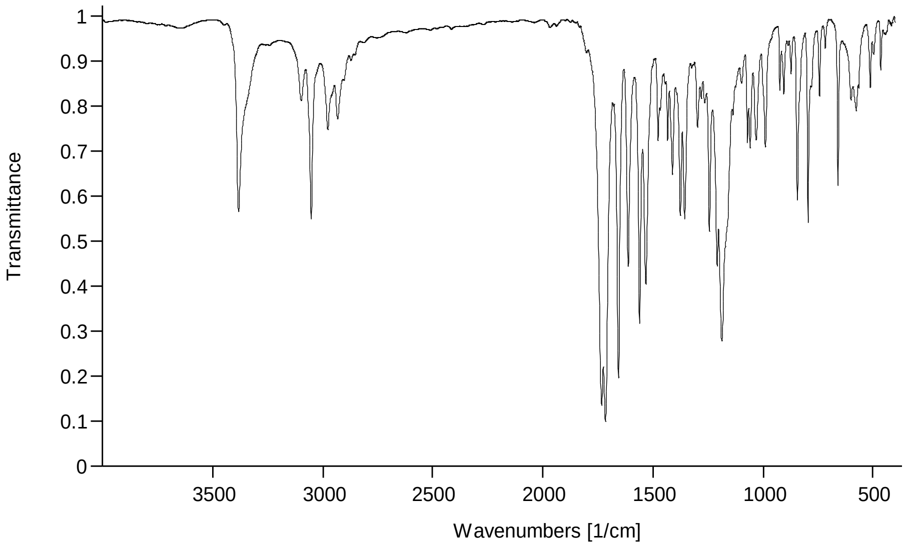

IR spectrum of **5h** in KBr

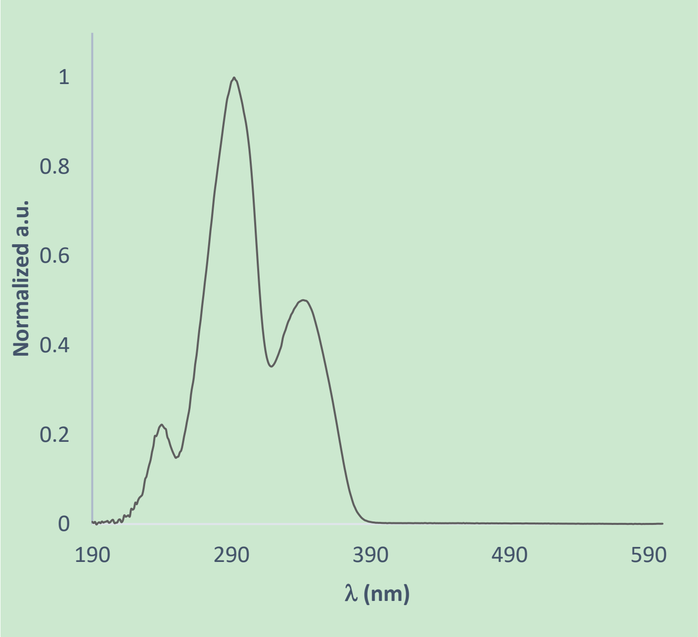

UV spectrum of **5h** in MeOH

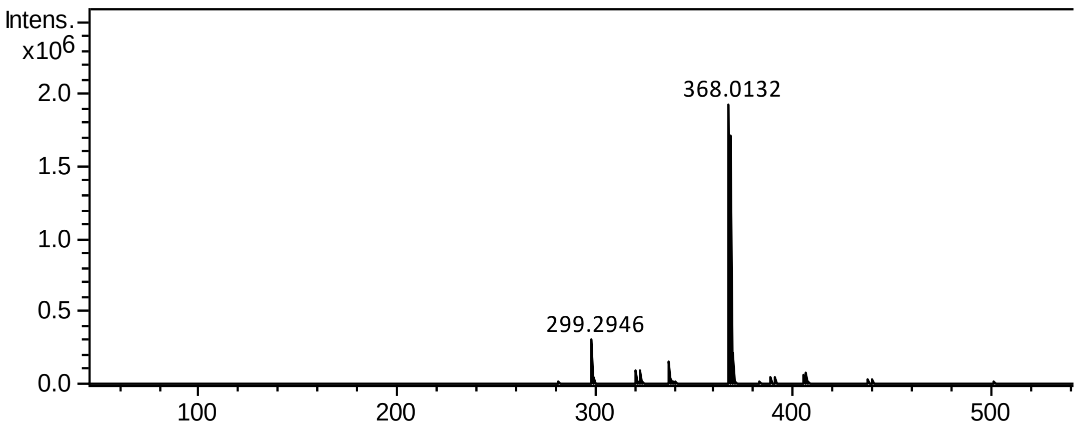

Mass spectrum, ESI(+), of **5h**

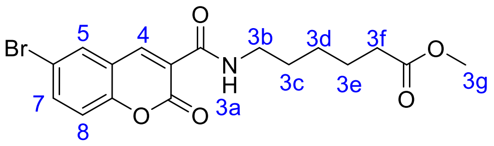

(5i) White solid (77% yield); mp 147-149 ºC; IR(KBr) $\nu_{max/cm-1}$= 3336, 3051, 1734, 1715, 1658; UV-vis (MeOH) $\lambda_{max/nm}$= 292; ^{1}H NMR (500 MHz, $CDCl_3$) δ 8.84 (s, 1H, H-4), 8.75 (s, 1H, H-3a), 7.84 (d, *J*= 2.2 Hz, 1H, H-5), 7.74 (dd, *J*= 8.8, 2.3 Hz, 1H, H-7), 7.30 (d, *J*= 8.8 Hz, 1H, H-8), 3.67 (s, 3H, H-3g), 3.46 (dd, *J*= 13.1, 7.0 Hz, 2H, H-3b), 2.34 (t, *J*= 7.5 Hz, 2H, H-3f), 1.67 (tt, *J*= 15.0, 7.5 Hz, 4H, H-3c,3e), 1.50-1.37 (m, 2H, H-3d); ^{13}C NMR (125 MHz, $CDCl_3$) δ 173.98, 160.91, 160.84, 153.16, 146.89, 136.67, 131.83, 120.14, 119.56, 118.33,

117.92, 51.52, 39.75, 33.89, 29.05, 26.50, 24.57; HRMS (ESI) *m/z* calcd. for $C_{17}H_{18}BrNO_5$ [M+H]$^+$ 396.0441, found 396.0448.

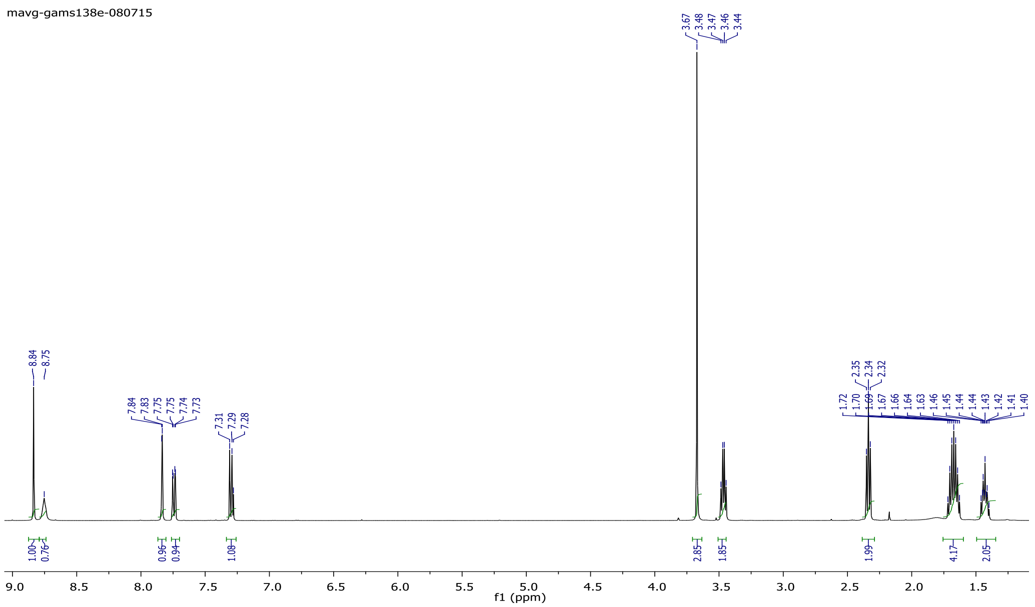

^{1}H spectrum of **5i** in $CDCl_3$, 500 MHz

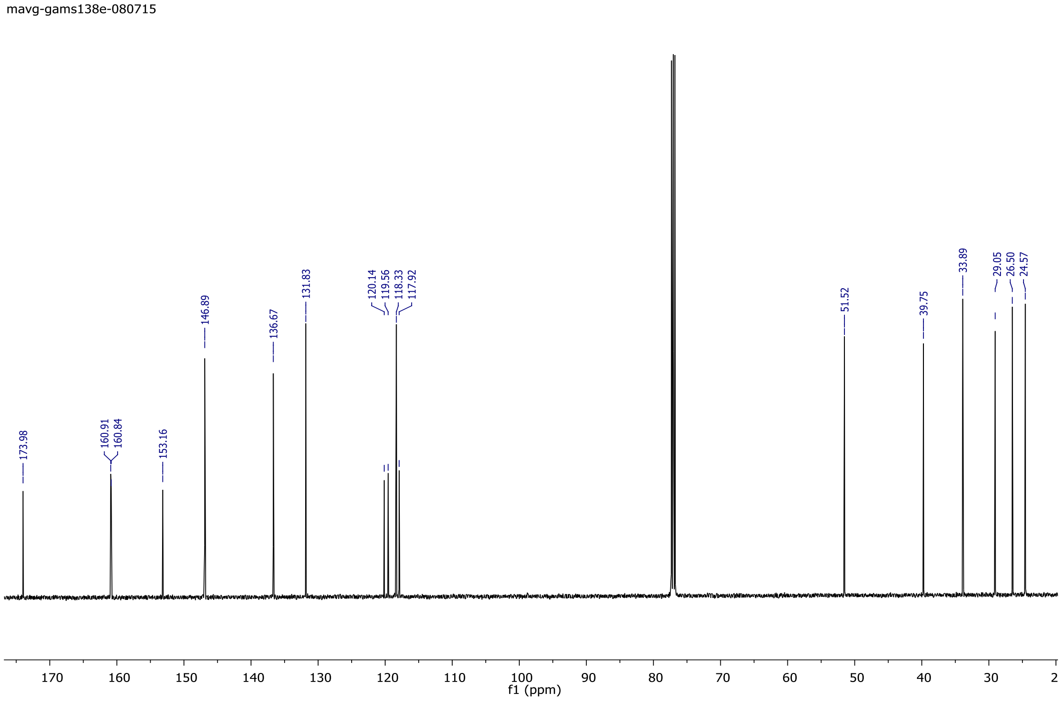

^{13}C spectrum of **5i** in $CDCl_3$, 125 MHz

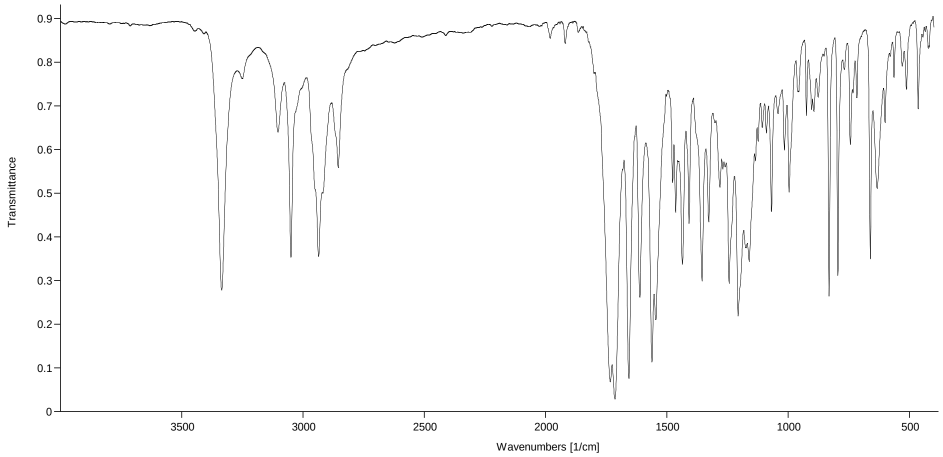

IR spectrum of **5i** in KBr

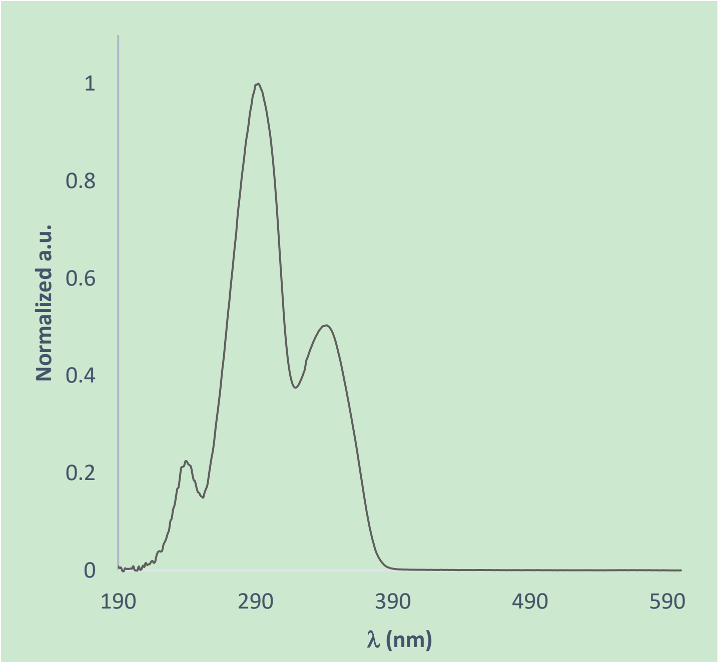

UV spectrum of **5i** in MeOH

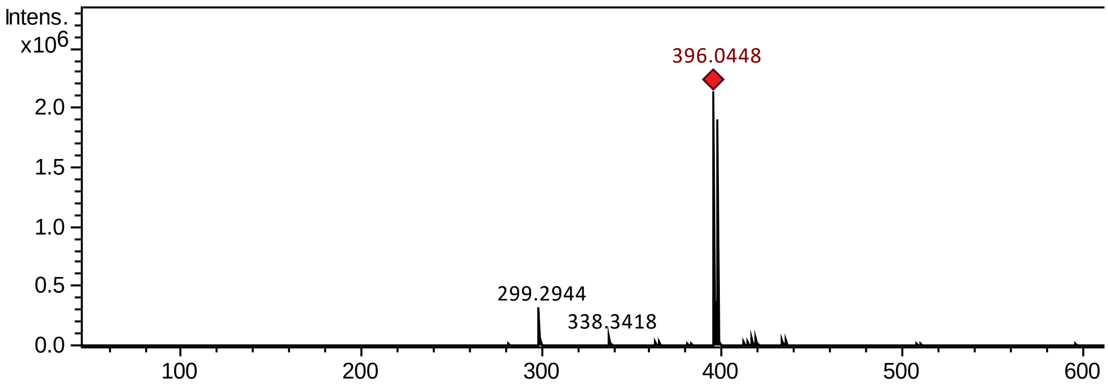

Mass spectrum, ESI(+), **5i**

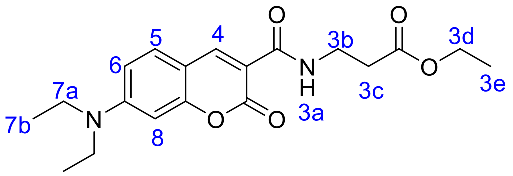

(5j) Yellow solid (40% yield); mp 134-136 ºC; IR(KBr) $\nu_{max/cm-1}$= 3444, 3320, 2940, 1739, 1702; UV-vis (MeOH) $\lambda_{max/nm}$= 419; ^{1}H NMR (500 MHz, $CDCl_3$) δ 9.08 (s, 1H,H-3a), 8.69 (s, 1H, H-4), 7.42 (d, *J*= 9.0 Hz, 1H, H-5), 6.64 (dd, *J*= 9.0, 2.1 Hz, 1H, H-6), 6.49 (d, *J*= 1.8 Hz, 1H,H-8), 4.19 (q, *J*= 7.1 Hz, 4H, H-7a), 3.73 (q, *J*= 6.3 Hz, 2H,H-3d), 3.45 (q, 2H, H-3b), 2.64 (t, *J*= 6.5 Hz, 2H,H-3c), 1.29 (t, *J*= 7.2 Hz, 3H, H-3e), 1.24 (t, *J*= 7.2 Hz, 6H, H-7b); ^{13}C NMR (125 MHz, $CDCl_3$) δ 171.94, 163.25, 162.61, 157.67, 152.55, 148.09, 131.13, 110.21, 109.91, 108.35, 96.59, 60.72, 45.07, 35.25, 34.49, 14.20, 12.42. HRMS (ESI) *m/z* calcd. for $C_{19}H_{24}N_2O_5$ $[M+H]^+$ 361.1758, found 361.1780.

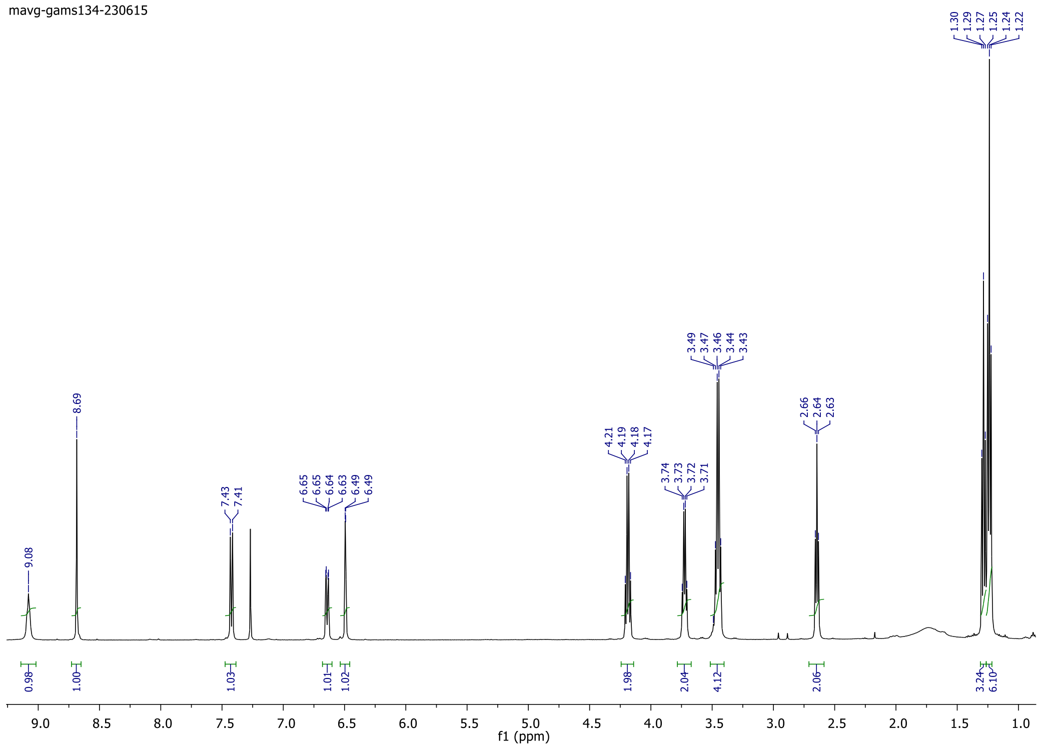

^{1}H spectrum of **5j** in $CDCl_3$, 500 MHz

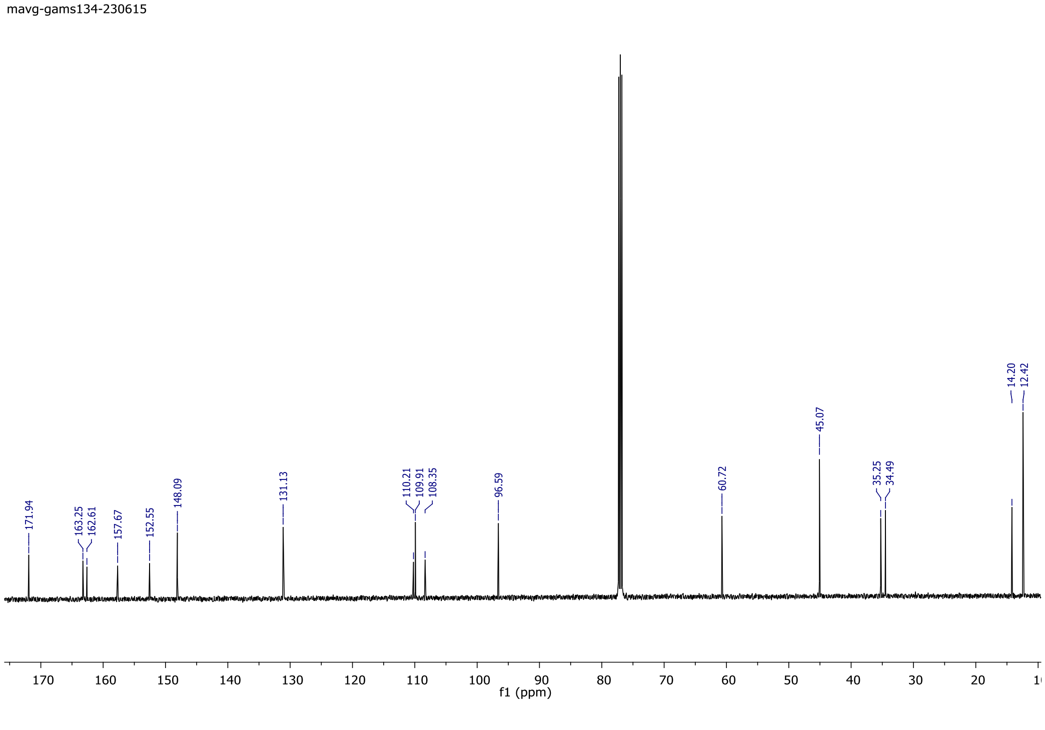

^{13}C spectrum of **5j** in $CDCl_3$, 125 MHz

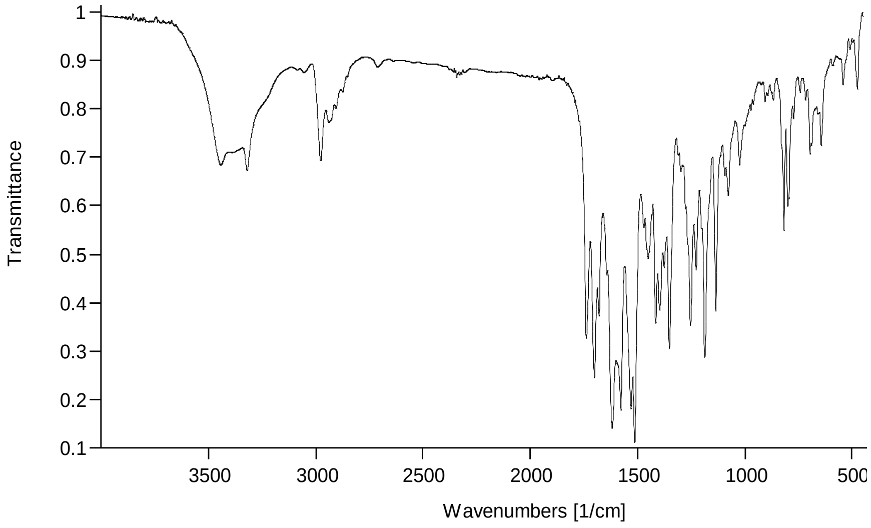

IR spectrum of **5j** in KBr

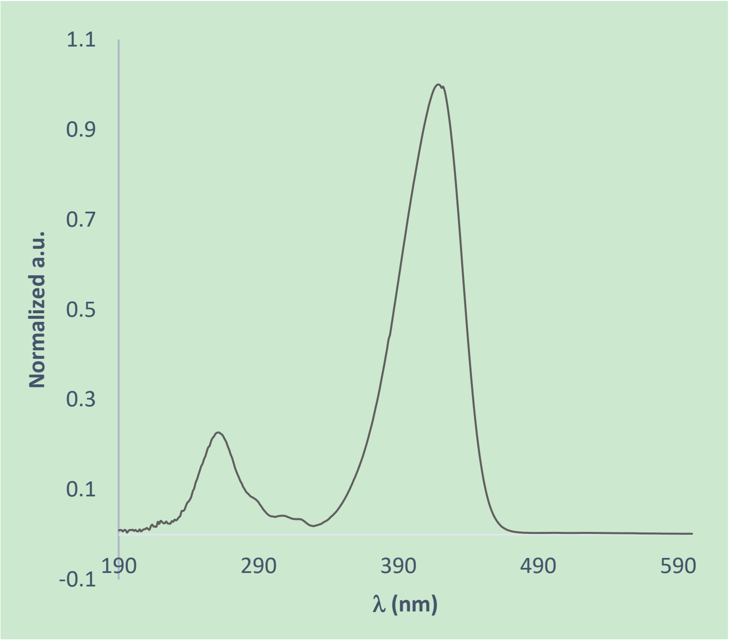

UV spectrum of **5j** in MeOH

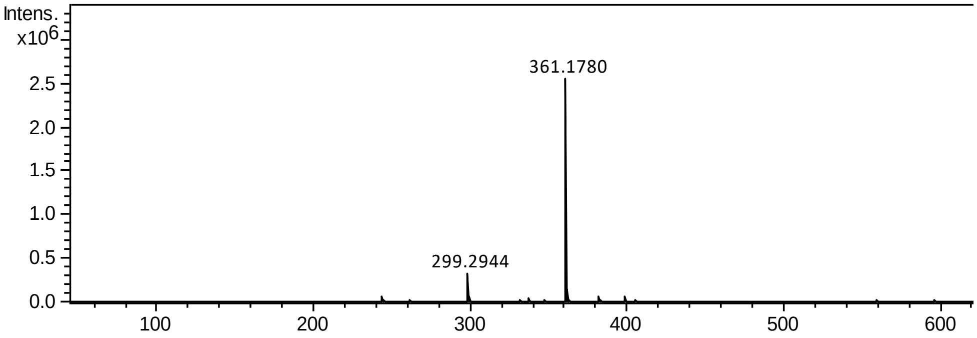

Mass spectrum, ESI(+), of **5j**

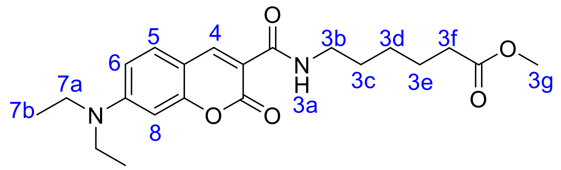

(5k) Yellow solid (61% yield); mp 90-91 ºC, IR(KBr) $\nu_{max/cm-1}$= 3367, 2978, 1735, 1688, 1650; UV-vis (MeOH) $\lambda_{max/nm}$= 418; ^{1}H NMR (500 MHz, $CDCl_3$) δ 8.80 (s, 1H,H-3a), 8.70 (s, 1H, H-4), 7.43 (d, *J*= 8.9 Hz, 1H, H-5), 6.64 (dd, *J*= 8.9, 2.3 Hz, 1H, H-6), 6.50 (d, *J*= 2.0 Hz, 1H, H-8), 3.67 (s, 3H, H-3g), 3.49-3.38 (m, 6H, H-7a,3b), 2.33 (t, *J*= 7.5 Hz, 2H, H-3f), 1.66 (qd, *J*= 15.0, 7.4 Hz, 4H, H-3c,3e), 1.51-1.36 (m, 2H, H-3d), 1.24 (t, *J*= 7.1 Hz, 6H,H-7b); ^{13}C NMR (125 MHz, $CDCl_3$) δ 174.08, 163.08, 162.80, 157.60, 152.47, 148.01, 131.08, 110.43, 109.91, 108.40, 96.56, 51.49, 45.06, 39.42, 33.97, 29.28, 26.58, 24.66. HRMS (ESI) *m/z* calcd. for $C_{21}H_{28}N_2O_5$ $[M+H]^+$ 389.2071, found 389.2102.

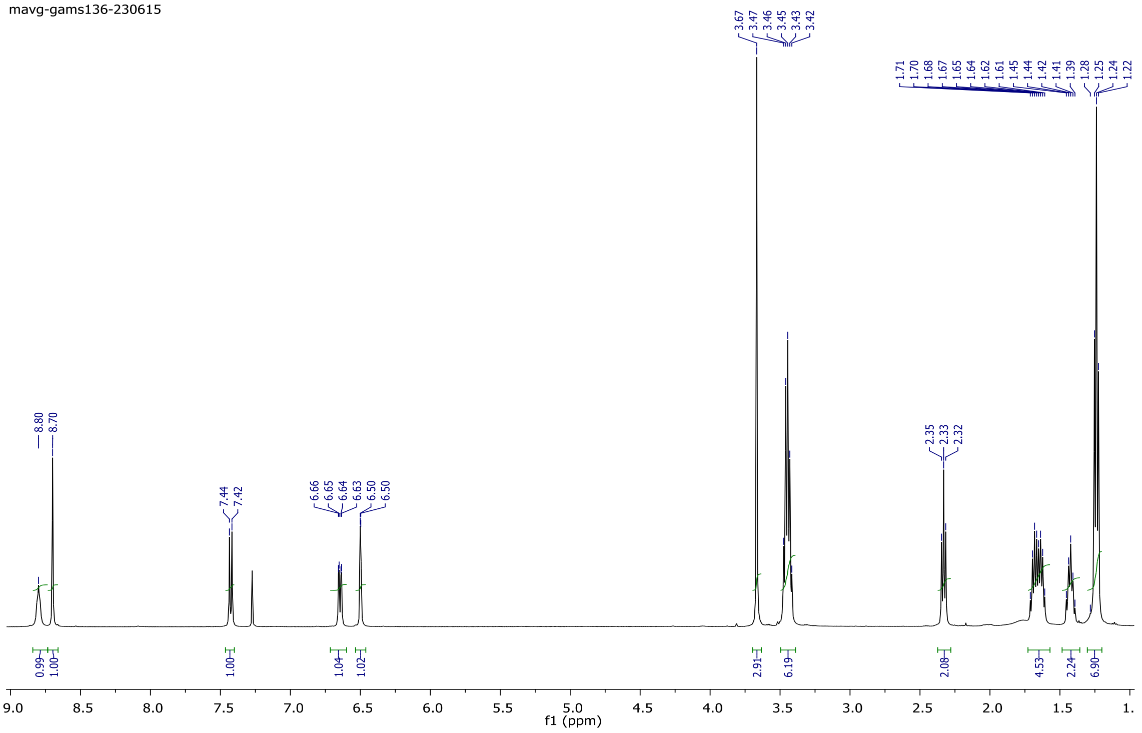

^{1}H spectrum of **5k** in $CDCl_3$, 500 MHz

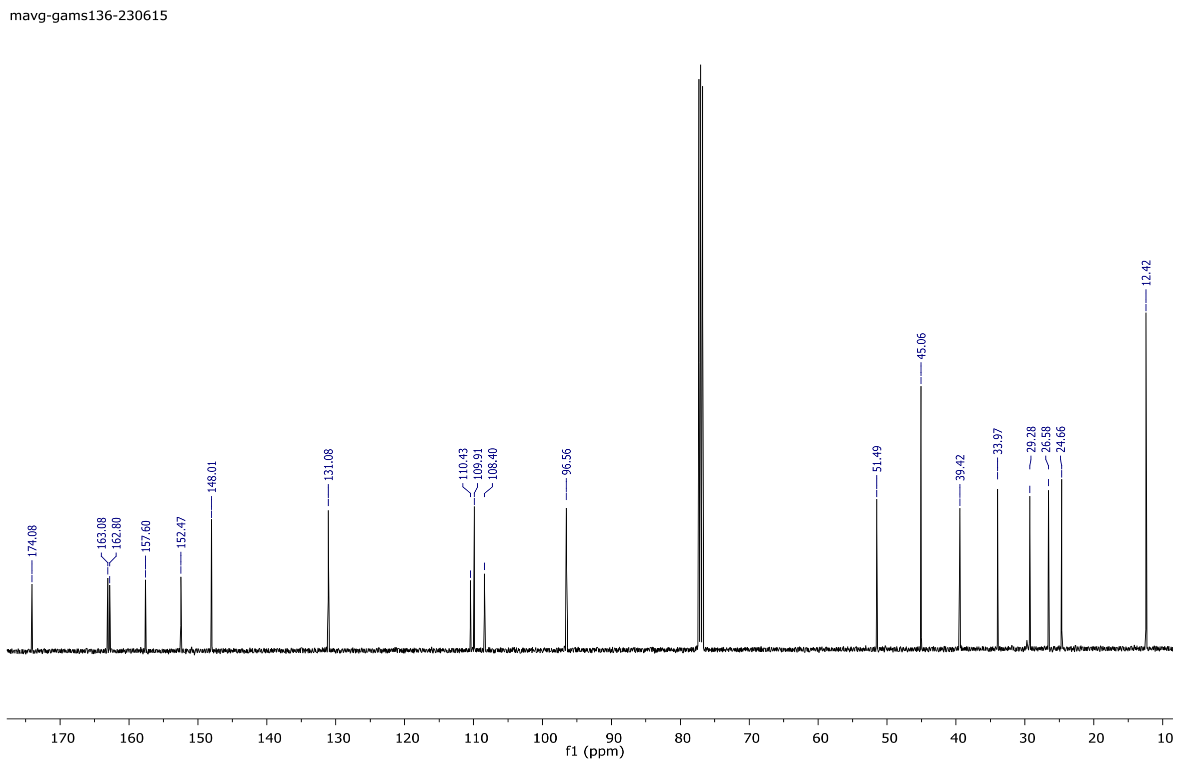

^{13}C spectrum of **5k** in $CDCl_3$, 125 MHz

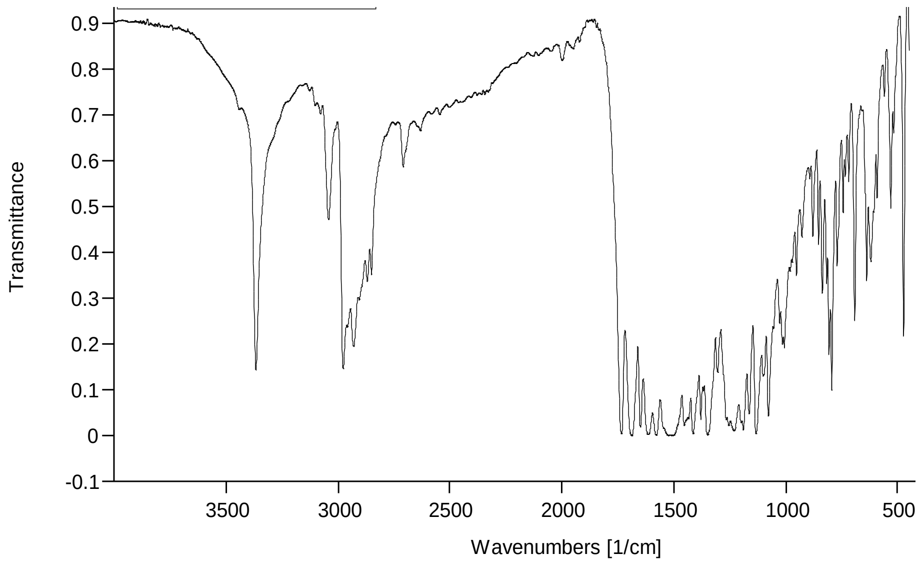

IR spectrum of **5k** in KBr

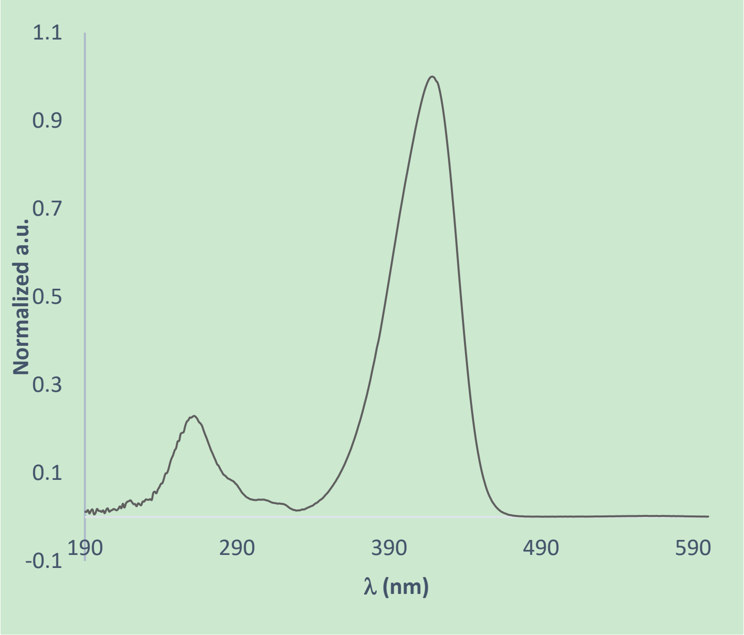

UV spectrum of **5k** in MeOH

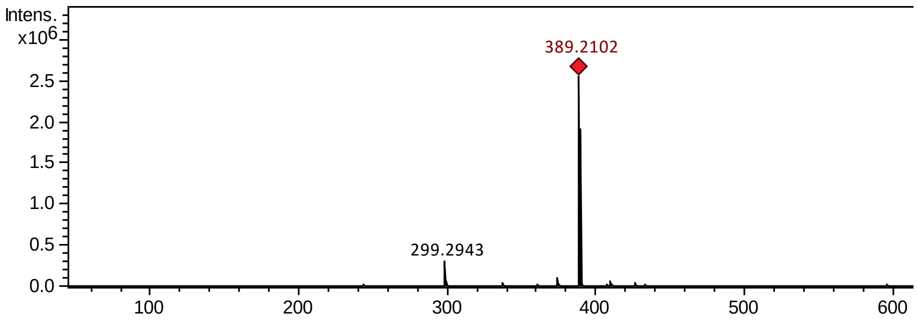

Mass spectrum, ESI(+), of **5k**

General procedure for the preparation of N-(2-oxo-2H-chromene-3-carboxamido) acids (6a-k)

In a round-bottom flask, the corresponding ester (1 mmol) was added and dissolved in 10 ml of THF:H_2O (1:2). The mixture was stirred at room temperature before adding LiOH (7 mmol) and stirring was continued overnight. Upon completion of the reaction, HCl was slowly added until a precipitate appeared, which was filtered and purified by recrystallization with MeOH and cold water to afford the desired product. Diethylamine derivatives were synthesized by using the Mravljak, J. *et al.* methodology (*Tetrahedron Lett.* **2013**, *54* (38), 5236-5238).

.

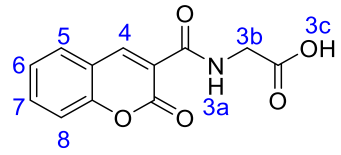

(6a) White solid (93% yield); mp 246-247 ºC; IR(KBr) $\nu_{max/cm-1}$= 3315, 2967, 2686, 1759, 1712, 1638; UV-vis (MeOH) $\lambda_{max/nm}$=297; ^{1}H NMR (400 MHz, DMSO-d$_6$) δ 12.78 (s, 1H, H-3c), 8.99 (s, 1H, H-3a), 8.86 (s, 1H, H-4), 7.95 (d, *J*= 7.4 Hz, 1H, H-8), 7.71 (t, *J*= 7.5 Hz, 1H, H-7), 7.46 (d, *J*= 8.1 Hz, 1H, H-5), 7.39 (t, *J*= 7.0 Hz, 1H, H-6), 4.00 (s, 2H, H-3b); ^{13}C NMR (100 MHz, DMSO-d$_6$) δ 171.27, 161.63, 160.84, 154.46, 148.58, 134.77, 130.89, 125.64, 118.89, 118.66, 116.64, 41.97; HRMS (ESI) *m/z* calcd. for $C_{12}H_9NO_5$ [M+H]$^+$ 248.0553, found 248.0561.

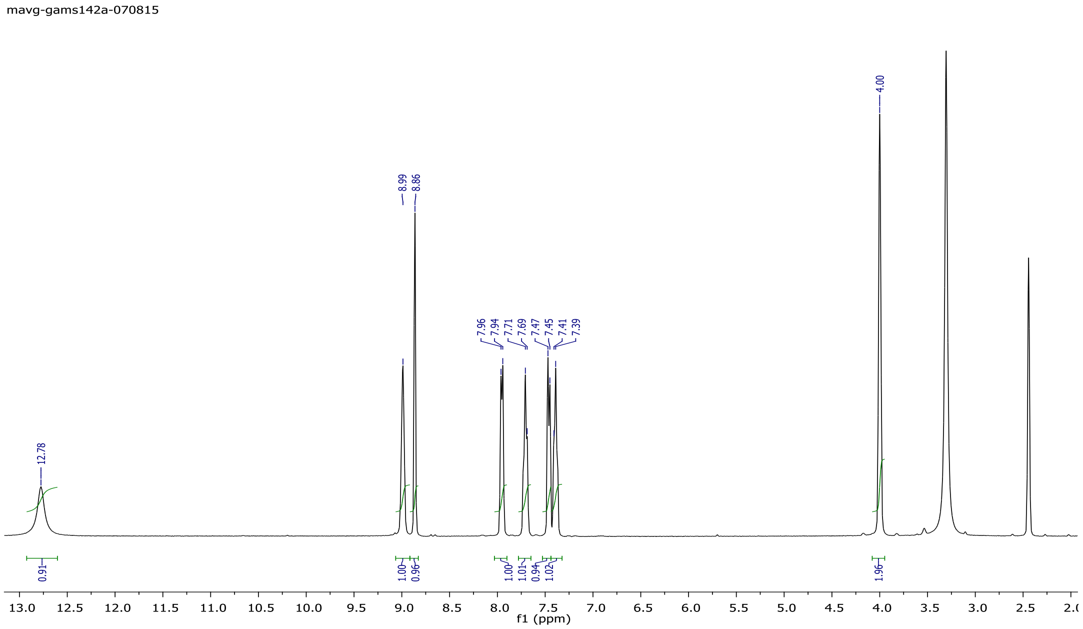

^{1}H spectrum of **6a** in DMSO-d$_6$, 400 MHz

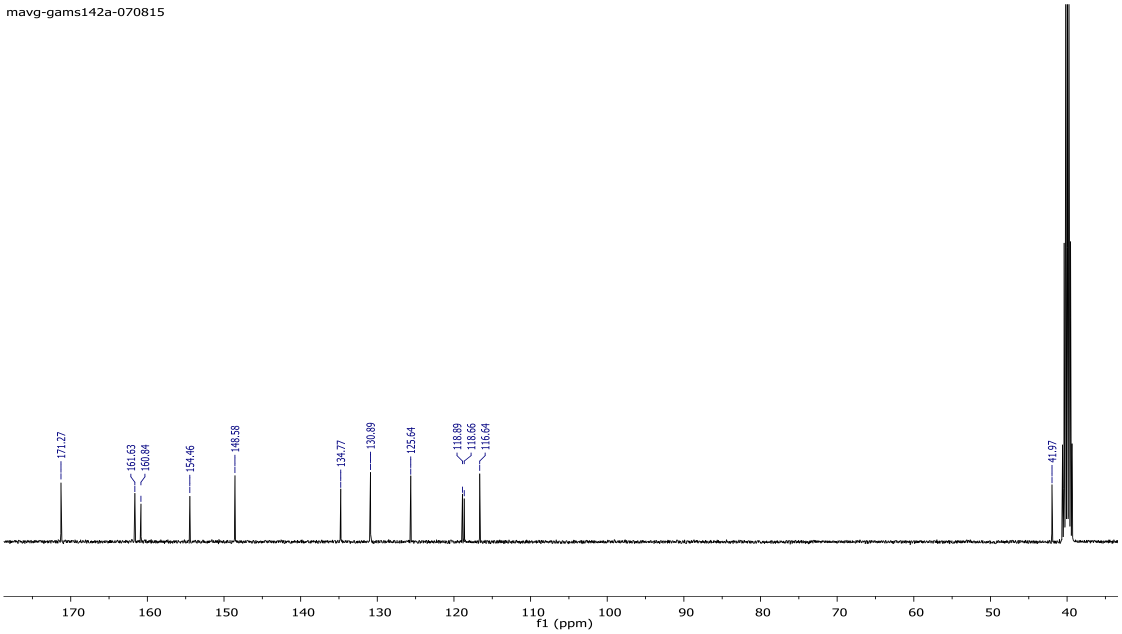

^{13}C spectrum of **6a** in DMSO-d_6, 100 MHz

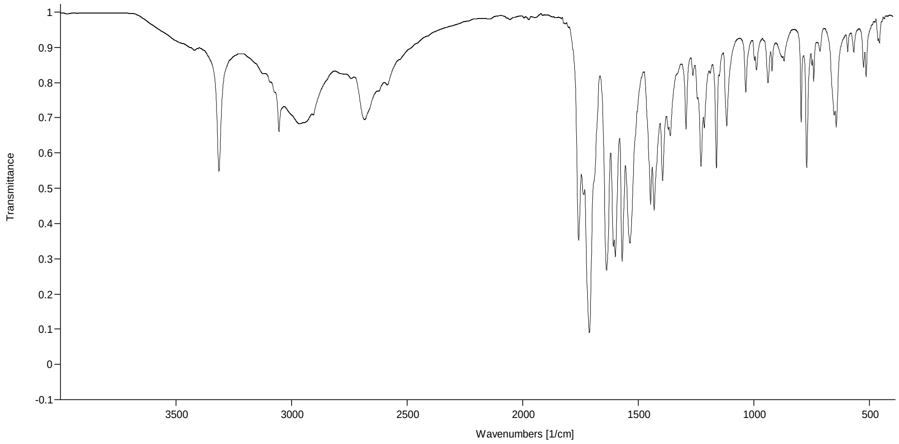

IR spectrum of **6a** in KBr

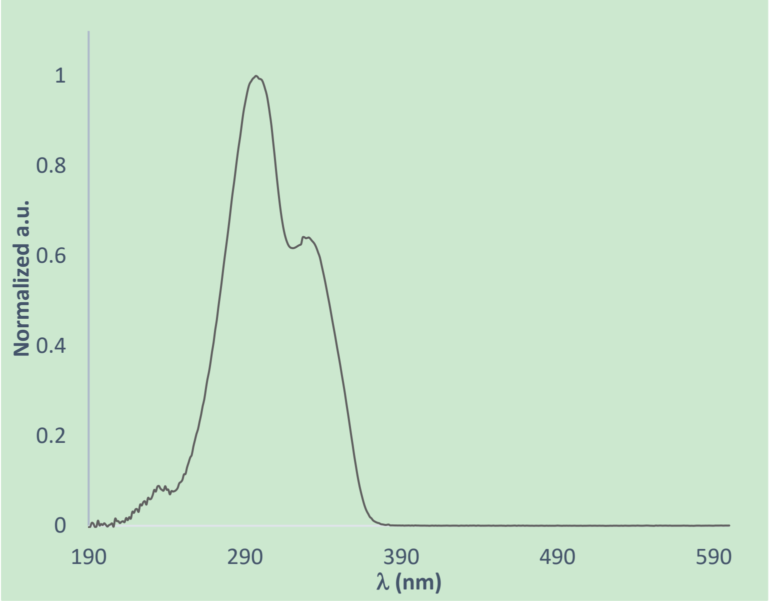

UV spectrum of **6a** in MeOH

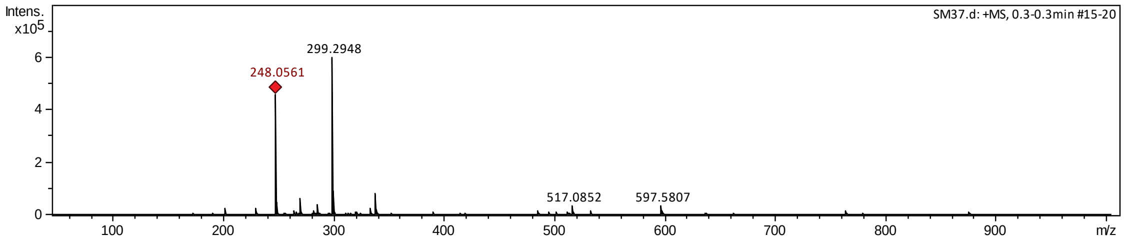

Mass spectrum, ESI(+), of **6a**

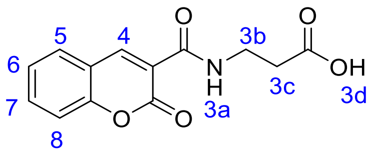

(6b) White solid (70% yield); mp 199-200 ºC; IR(KBr) $\nu_{max/cm-1}$= 3323, 3068, 1725, 1706, 1635; UV-vis (MeOH) $\lambda_{max/nm}$= 297; ^{1}H NMR (400 MHz, DMSO-d$_6$) δ 12.34 (s, 1H, H-3d), 8.85 (s, 1H, H-3a), 8.82 (s, 1H, H-4), 7.93 (d, *J*= 7.4 Hz, 1H, H-8), 7.69 (t, *J*= 7.4 Hz, 1H, H-7), 7.44 (d, *J*= 8.2 Hz, 1H, H-5), 7.38 (t, *J*= 7.1 Hz, 1H, H-4), 3.47 (d, *J*= 5.0 Hz, 2H, H-3b), 2.46 (d, *J*= 7.7 Hz, 2H, H-3c); ^{13}C NMR (100 MHz, DMSO-d$_6$) δ 173.58, 161.46, 160.86, 154.35, 148.14, 134.58, 130.76, 125.59, 119.08, 118.92, 116.59, 35.55, 34.13; HRMS (ESI) *m/z* calcd. for $C_{13}H_{11}NO_5$ $[M+H]^+$ 262.0710, found 262.0719.

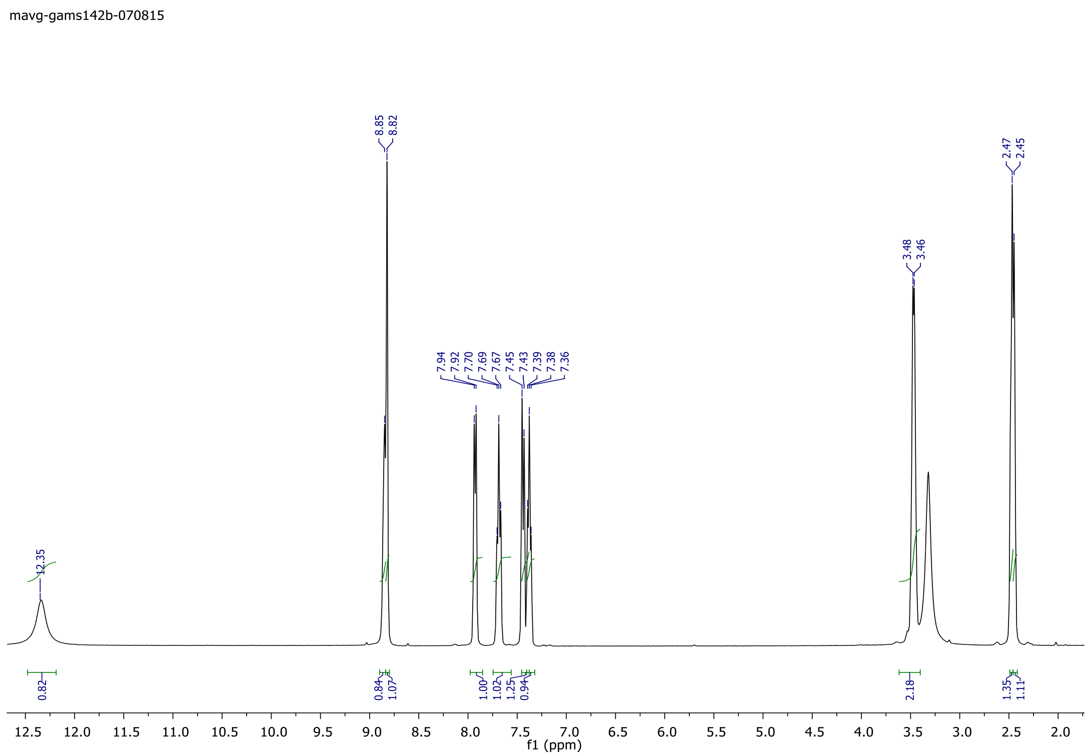

^{1}H spectrum of **6b** in DMSO-d_6, 400 MHz

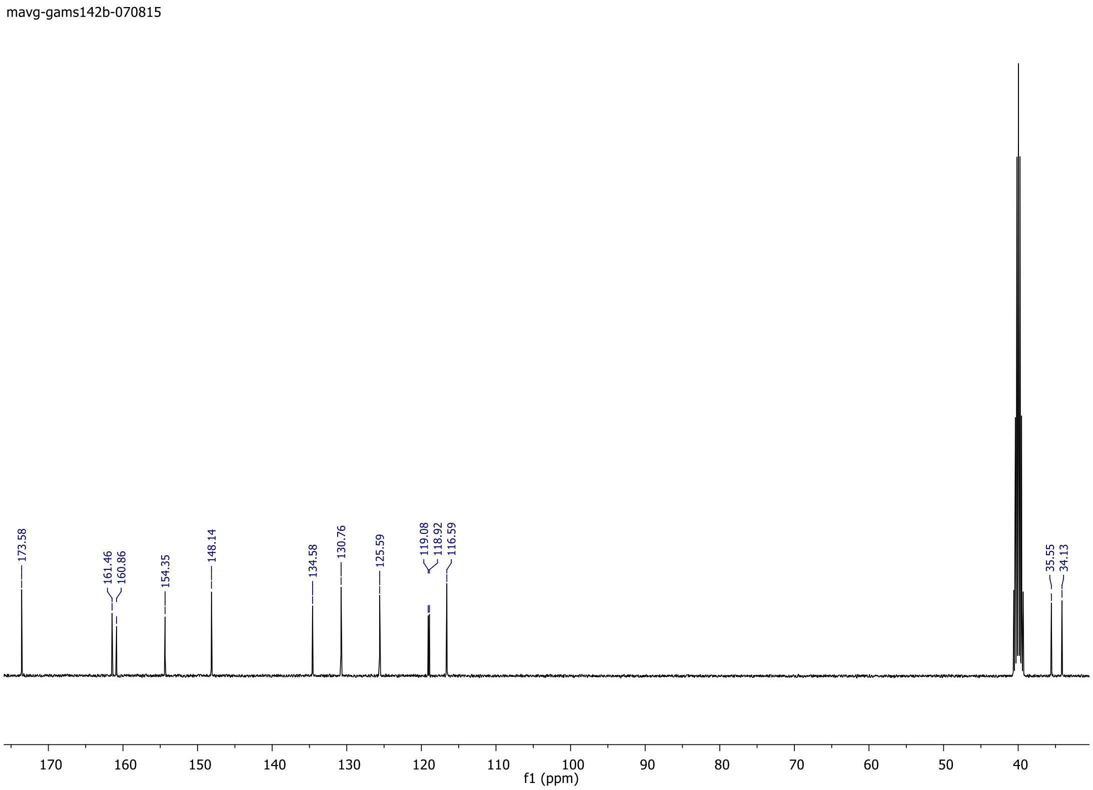

^{13}C spectrum of **6b** in DMSO-d_6, 100 MHz

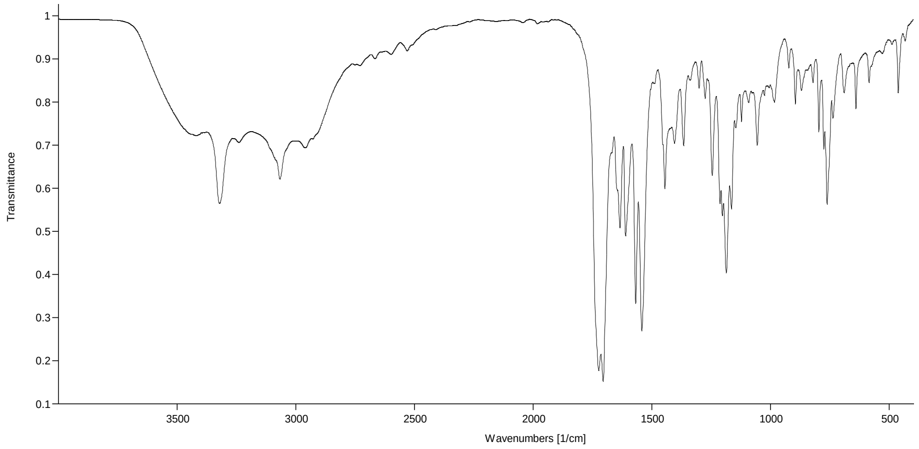

IR spectrum of **6b** in KBr

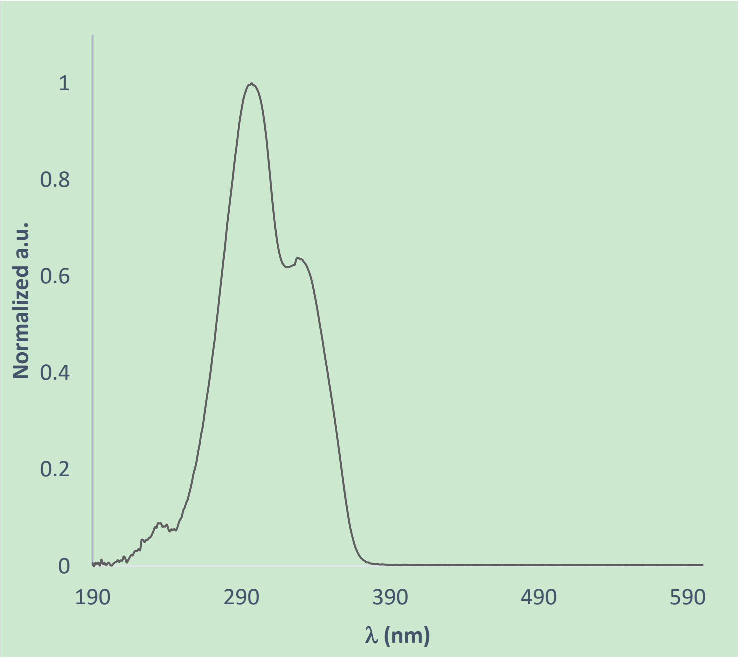

UV spectrum of **6b** in MeOH

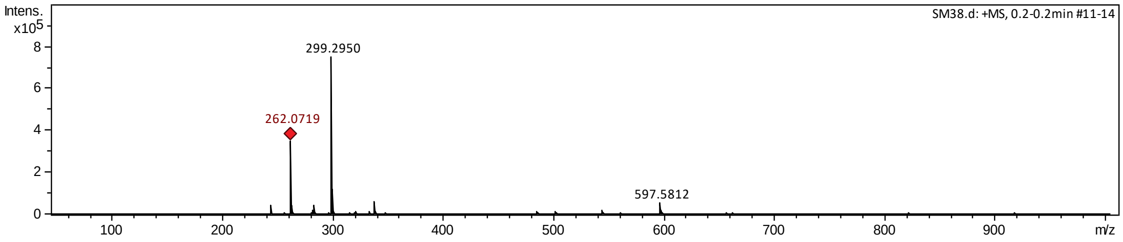

Mass spectrum, ESI(+), of **6b**

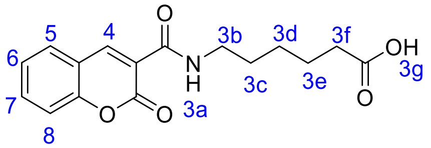

(6c) White solid (75% yield); mp 145-146 ºC; IR(KBr) $\nu_{max/cm-1}$= 3357, 1710, 1694, 1527; UV-vis (MeOH) $\lambda_{max/nm}$= 299; ^{1}H NMR (500 MHz, DMSO-d$_6$) δ 12.01 (s, 1H, H-3g), 8.85 (s, 1H, H-4), 8.69 (t, *J*= 5.6 Hz, 1H, H-3a), 7.99 (d, *J*= 7.4 Hz, 1H, H-8), 7.88-7.65 (m, 1H, H-7), 7.51 (d, *J*= 8.3 Hz, 1H, H-5), 7.44 (t, *J*= 7.4 Hz, 1H, H-6), 3.39-3.29 (m, 2H, H-3b), 2.22 (t, *J*= 7.3 Hz, 2H, H-3f), 1.62-1.47 (m, 4H, H-3c,3e), 1.36-1.27 (m, 2H, H-3d); ^{13}C NMR (125 MHz, DMSO-d$_6$) δ 174.86, 161.48, 160.85, 154.31, 147.72, 134.45, 130.67, 125.57, 119.62, 118.96, 116.58, 39.42, 34.04, 29.15, 26.43, 24.64; HRMS (ESI) *m/z* calcd. for $C_{16}H_{17}NO_5$ $[M+H]^+$ 304.1179, found 304.1184.

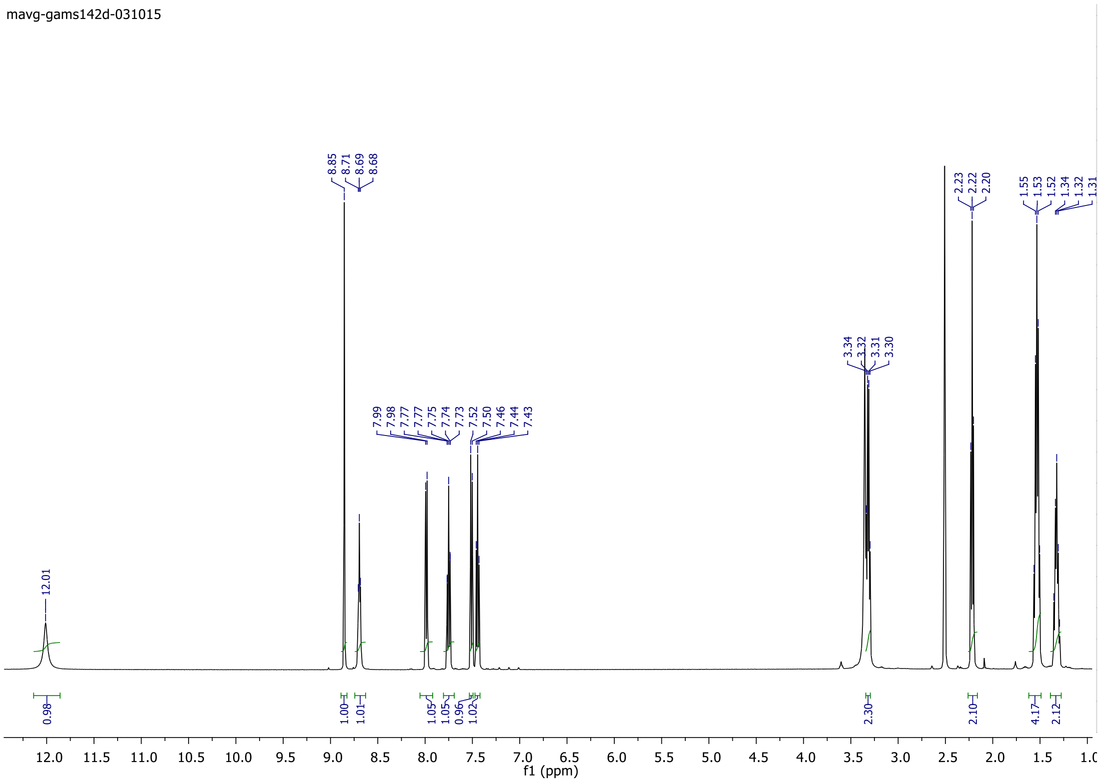

^{1}H spectrum of **6c** in DMSO-d$_6$, 500 MHz

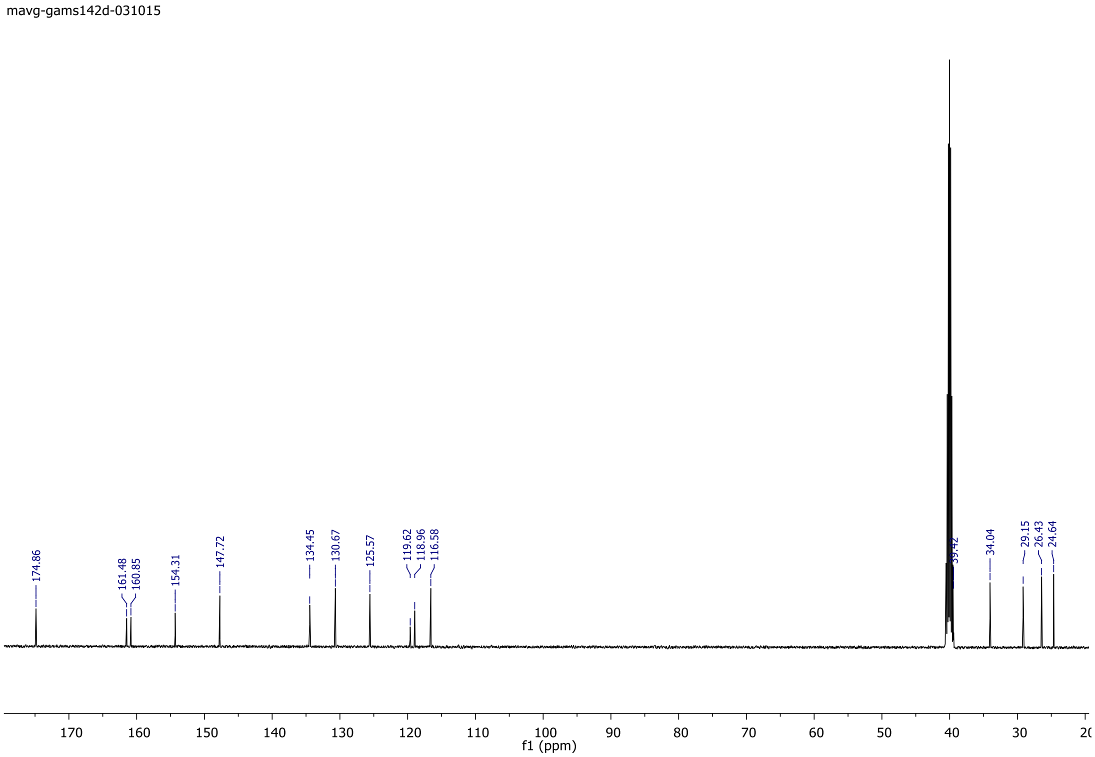

^{13}C spectrum of **6c** in DMSO-d$_6$, 125 MHz

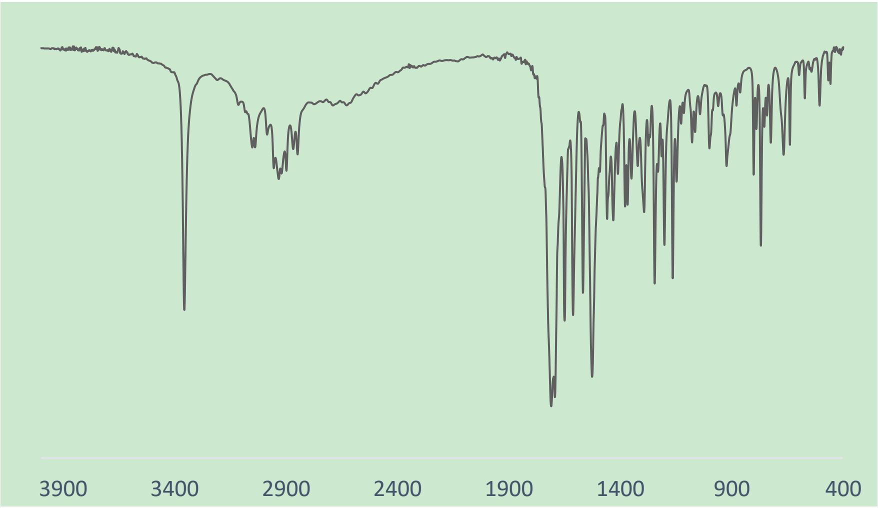

IR spectrum of **6c** in KBr

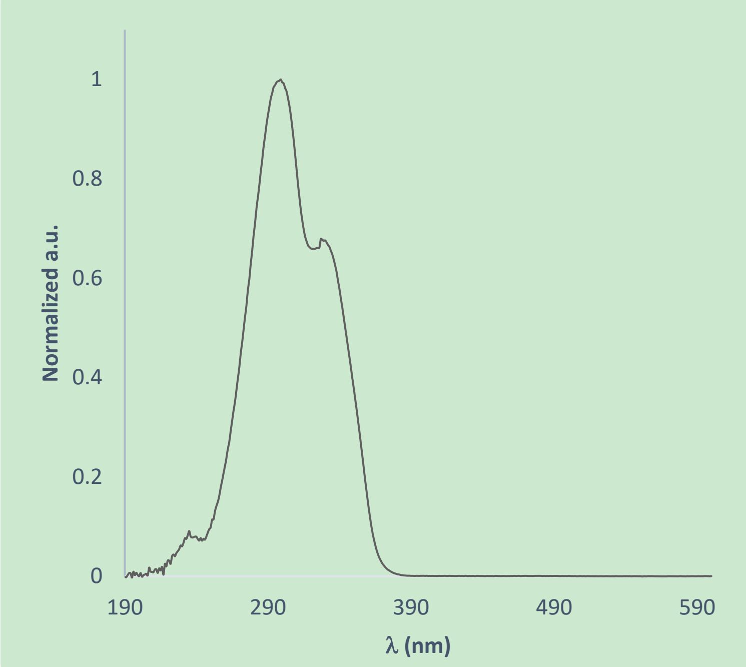

UV spectrum of **6c** in MeOH

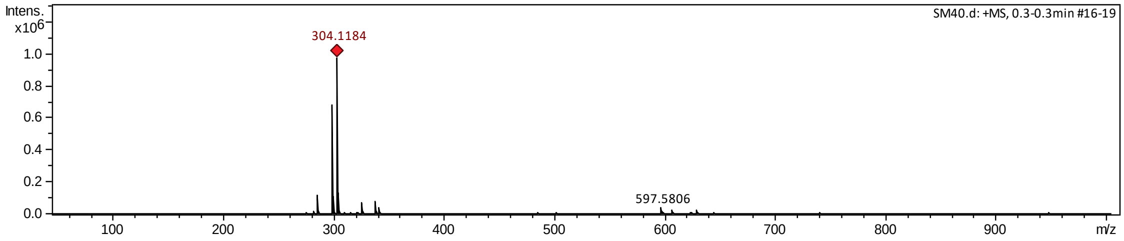

Mass spectrum, ESI(+), of **6c**

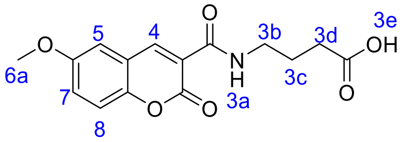

(6d) Light yellow solid (81% yield); mp 200-203 ºC; IR(KBr) $\nu_{max/cm-1}$= 3311, 1700, 1531; UV-vis (MeOH) $\lambda_{max/nm}$= 298; ^{1}H NMR (500 MHz, DMSO-d$_6$) δ 12.12 (s, 1H, H-3e), 8.78 (s, 1H, H-4), 8.74 (t, *J*= 5.8 Hz, 1H, H-3a), 7.52 (d, *J*= 2.9 Hz, 1H, H-5), 7.43 (d, *J*= 9.1 Hz, 1H, H-8), 7.32 (dd, *J*= 9.1, 3.0 Hz, 1H, H-7), 3.82 (s, 3H, H-6a), 3.36 (dd, *J*= 13.1, 6.7 Hz, 2H, H-3b), 2.30 (t, *J*= 7.4 Hz, 2H, H-3d), 1.78 (p, *J*= 7.2 Hz, 2H, H-3c); ^{13}C NMR (125 MHz, DMSO-d$_6$) δ 174.54, 161.66, 160.88, 156.40, 148.78, 147.54, 122.29, 119.64, 119.35, 117.65, 112.24, 56.27, 39.00, 31.59, 24.99; HRMS (ESI) *m/z* calcd. for $C_{15}H_{15}NO_6$ $[M+H]^+$ 306.0972, found 306.0974.

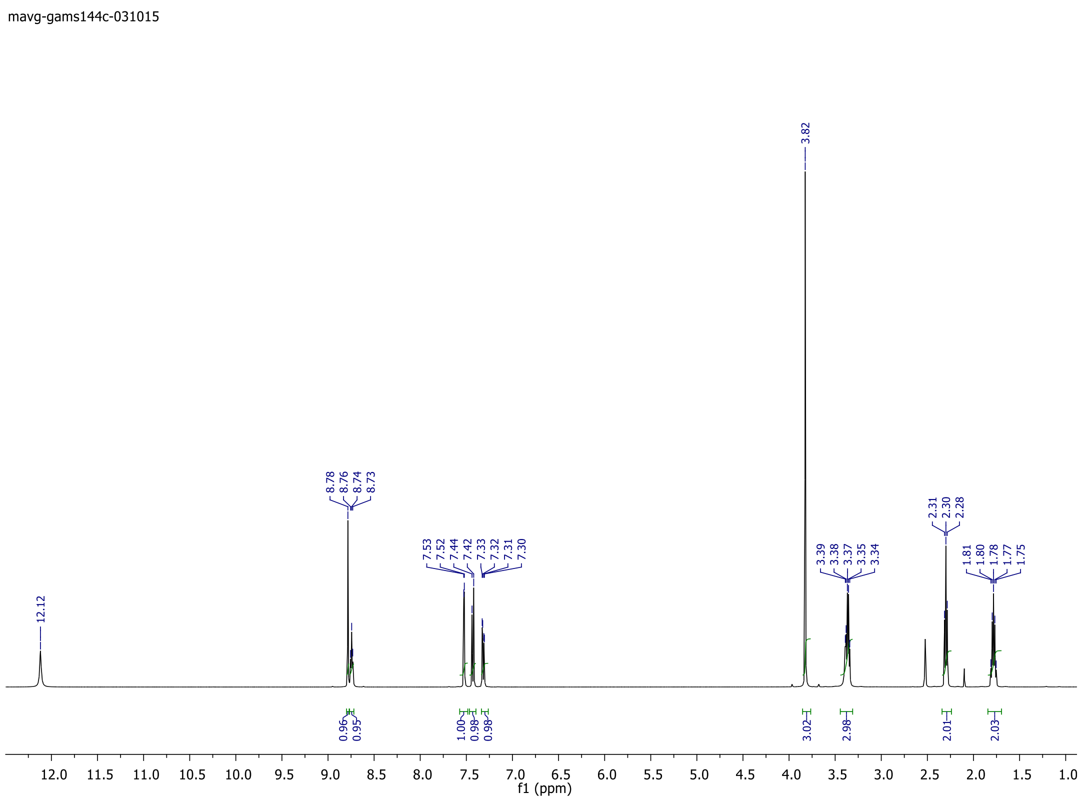

^{1}H spectrum of **6d** in DMSO-d$_6$, 500 MHz

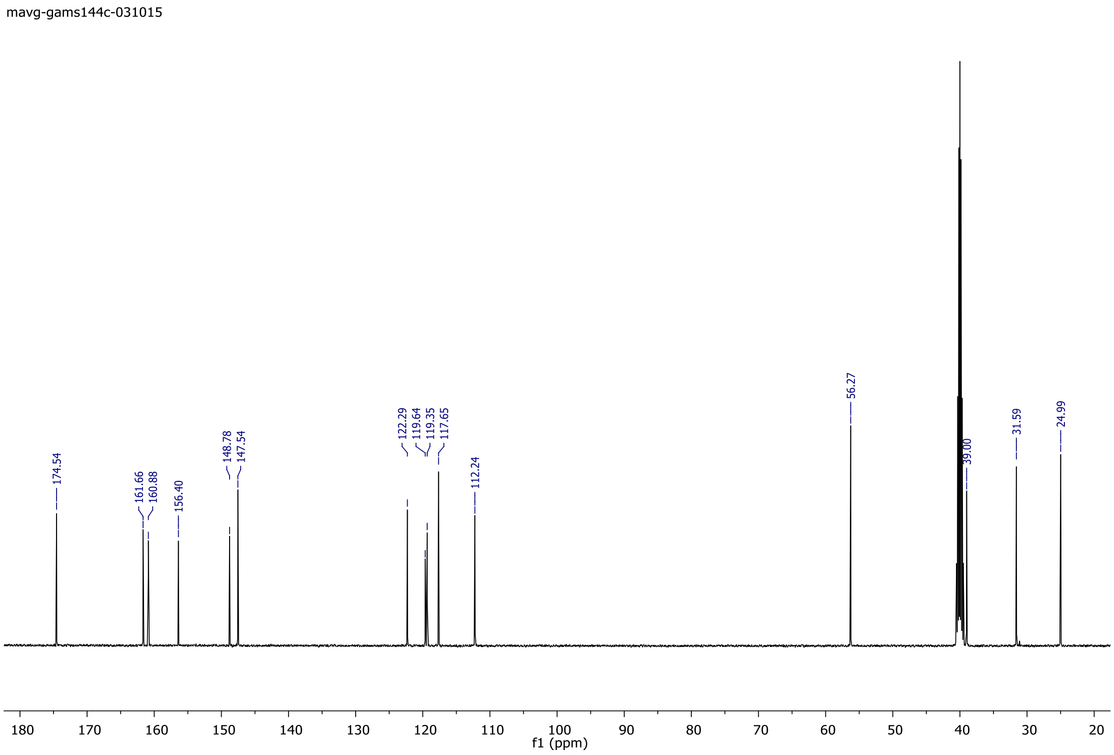

^{13}C spectrum of **6d** in DMSO-d$_6$, 500 MHz

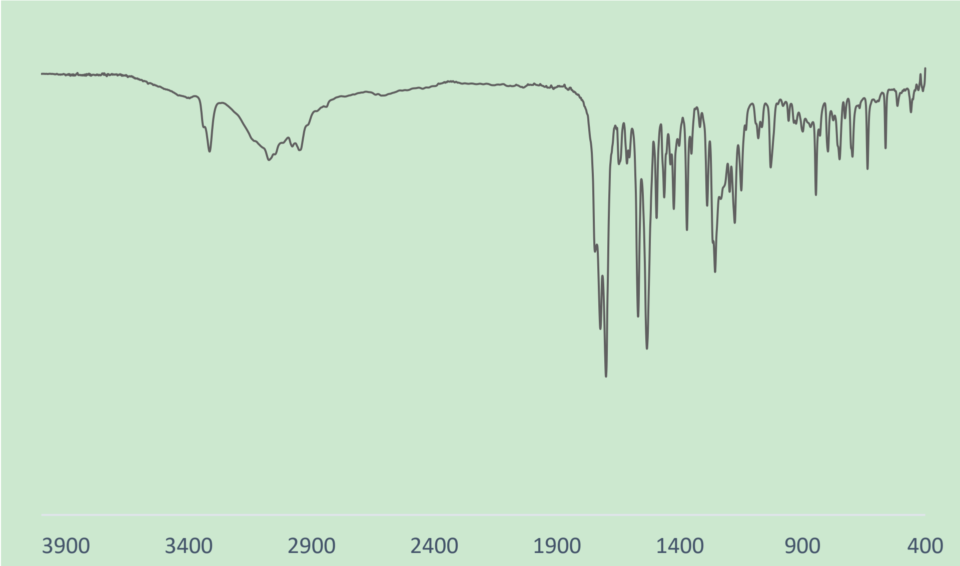

IR spectrum of **6d** in KBr

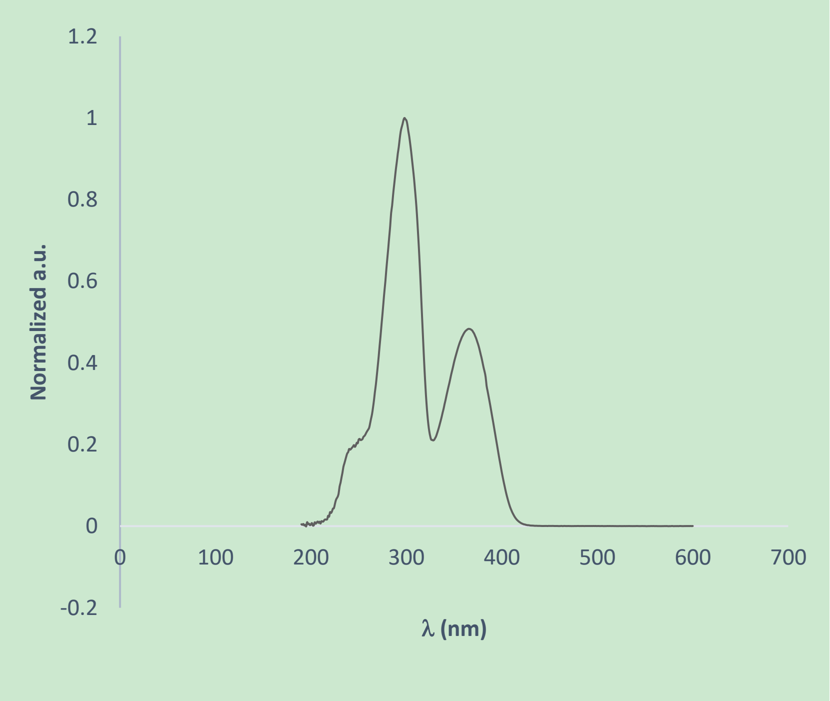

UV spectrum of **6d** in MeOH

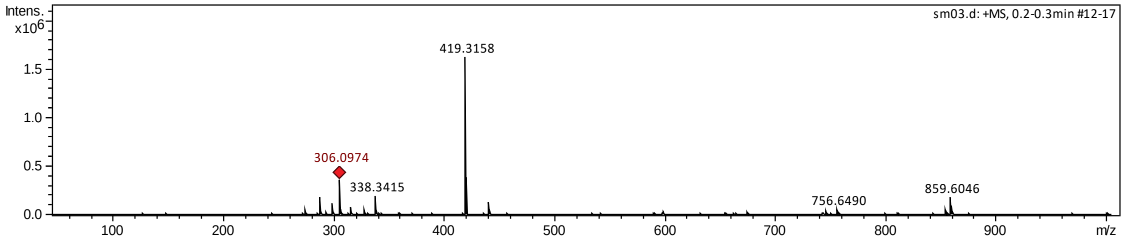

Mass spectrum, ESI (+), of **6d**

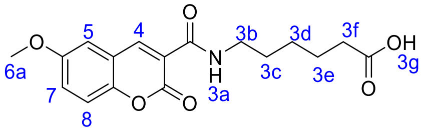

(6e) Light yellow solid (81% yield); mp 142-144 ºC; IR(KBr) $\nu_{max/cm-1}$= 3322, 1721, 1702, 1575 ; UV-vis (MeOH) $\lambda_{max/nm}$= 298; ^{1}H NMR (500 MHz, DMSO-d_6) δ 8.79 (s, 1H, H-4), 8.72 (s, 1H, H-3a), 7.52 (s, 1H, H-5), 7.42 (d, *J*= 9.2 Hz, 1H, H-8), 7.38-7.27 (m, 1H, H-7), 3.82 (s, 3H, H-6a), 3.32 (dd, *J*= 13.0, 6.7 Hz, 2H, H-3b), 2.23 (t, *J*= 7.3 Hz, 2H, H-3f), 1.61-1.49 (m, 4H, H-3c,3e), 1.40-1.30 (m, 2H, H-3d); ^{13}C NMR (125 MHz, DMSO-d_6) δ 174.85, 161.45, 160.98, 156.41, 148.77, 147.61, 122.32, 119.52, 119.36, 117.66, 112.23, 56.26, 39.42, 34.04, 29.14, 26.44, 24.64; HRMS (ESI) *m/z* calcd. for $C_{17}H_{19}NO_6$ $[M+H]^+$ 334.1285, found 334.1288.

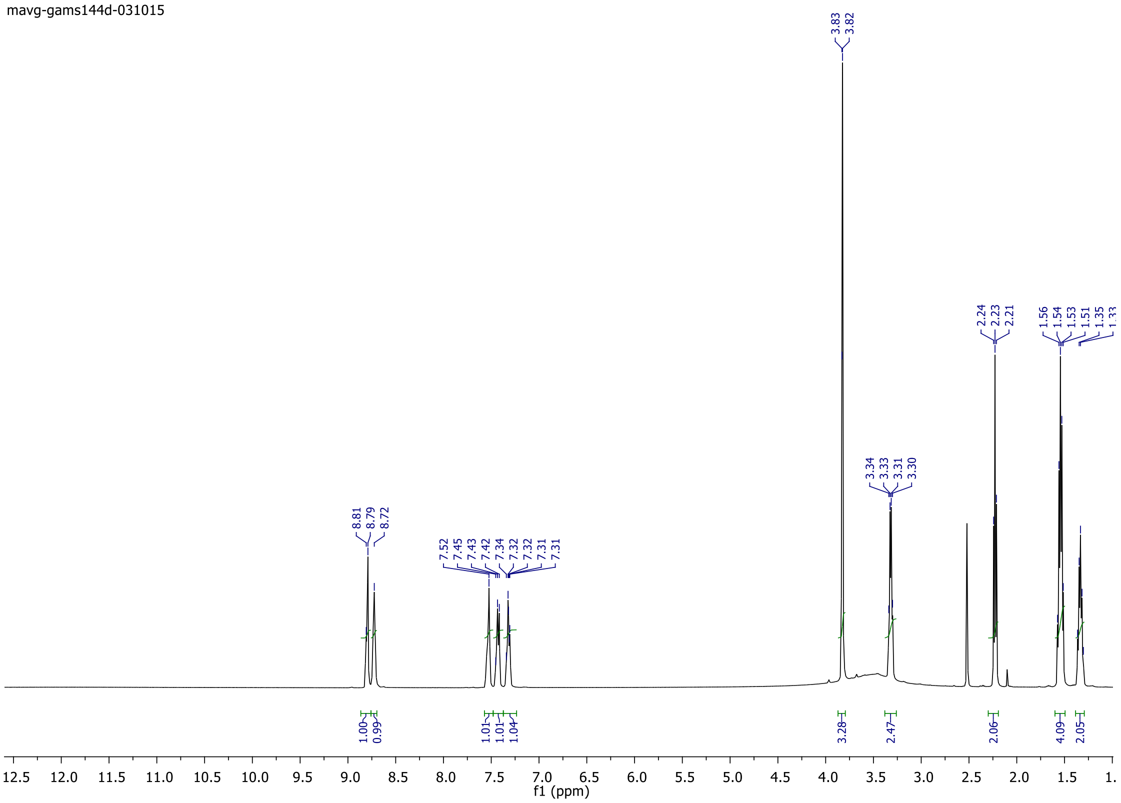

^{1}H spectrum of **6e** in DMSO-d_6, 500 MHz

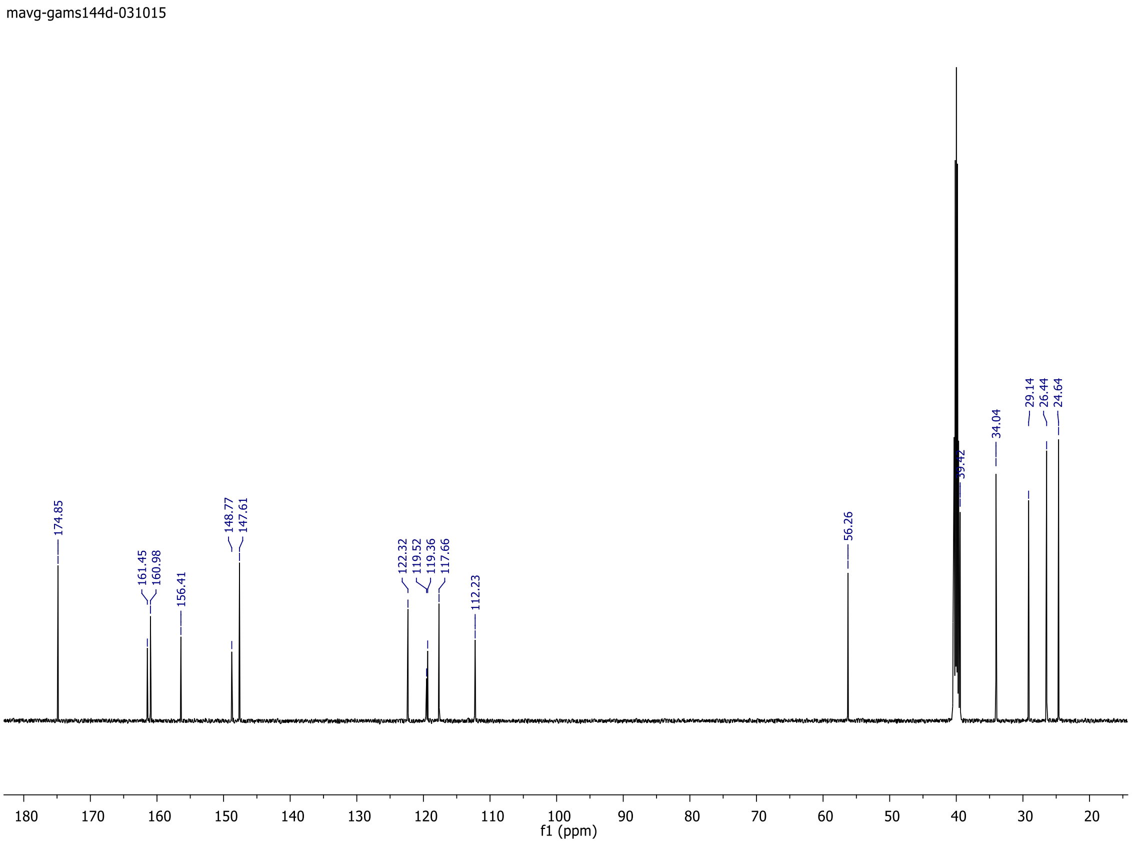

^{13}C spectrum of **6e** in DMSO-d$_6$, 125 MHz

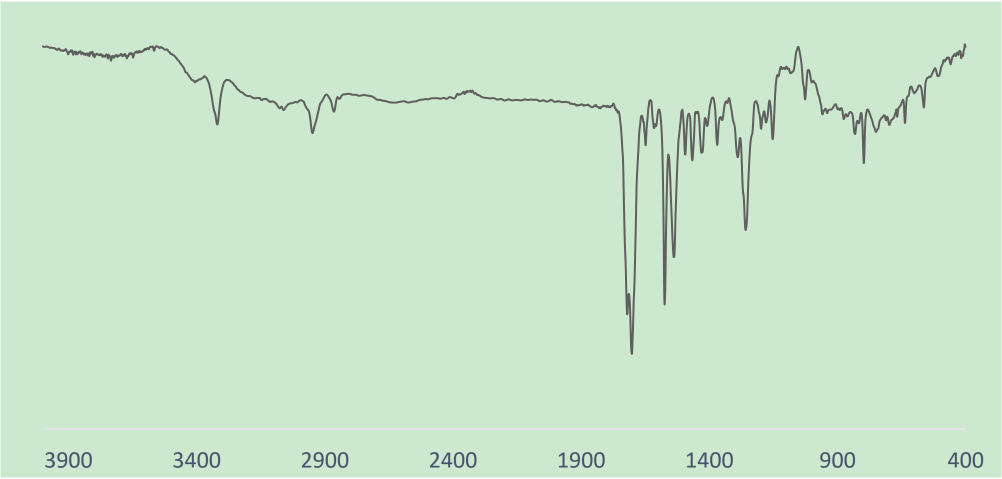

IR spectrum of **6e** in KBr

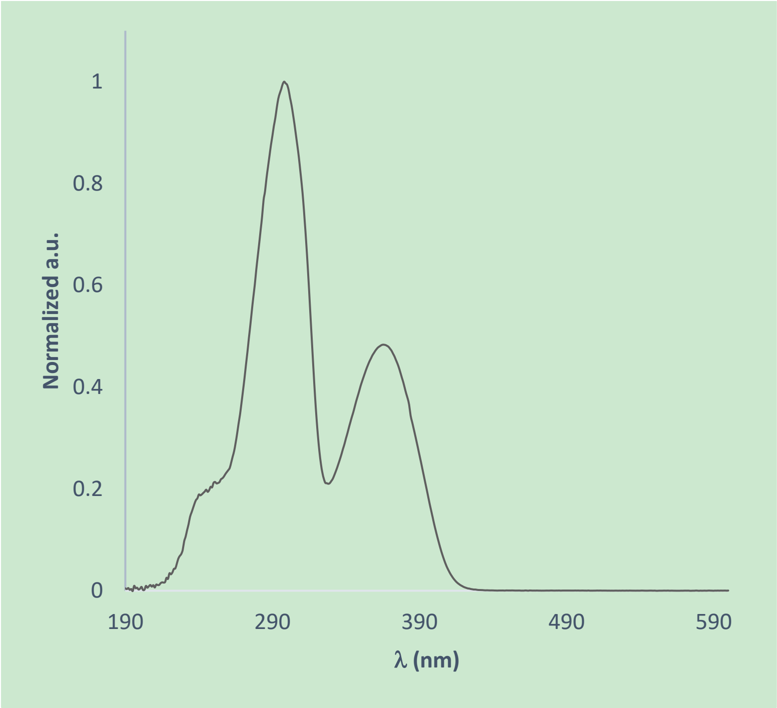

UV spectrum of **6e** in MeOH

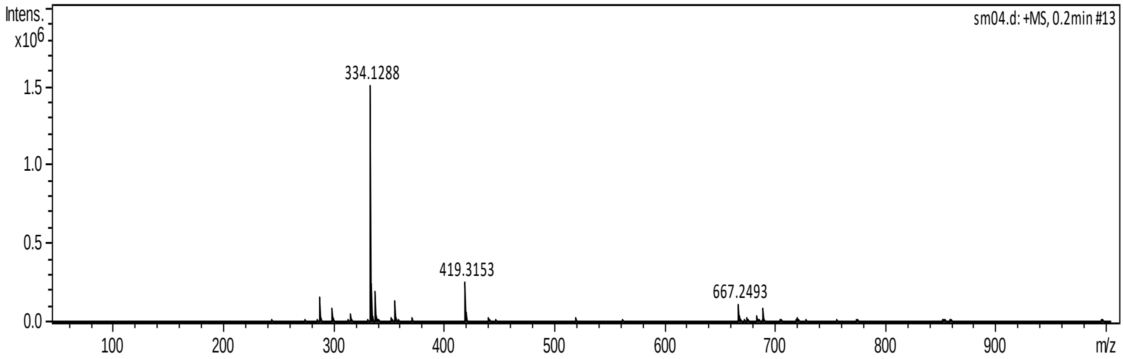

Mass spectrum, ESI(+), of **6e**

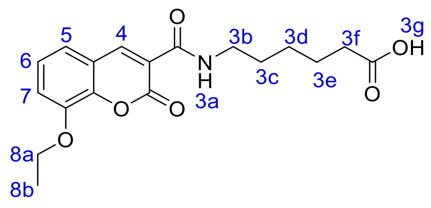

(6f) White solid (83% yield); mp 169-170 ºC; IR(KBr) $\nu_{max/cm^{-1}}$= 3337, 1716, 1605, 1542; UV-vis (MeOH) $\lambda_{max/nm}$= 312; ^{1}H NMR (500 MHz, DMSO-d$_6$) δ 12.02 (s, 1H, H-3g), 8.80 (s, 1H, H-4), 8.69 (t, *J*= 5.7 Hz, 1H, H-3a), 7.49 (d, *J*= 7.1 Hz, 1H, H-7), 7.40 (d, *J*= 7.6 Hz, 1H, H-5), 7.34 (t, *J*= 7.9 Hz, 1H, H-6), 4.20 (q, *J*= 6.9 Hz, 2H, H-8a), 3.32 (dd, *J*= 13.2, 6.7 Hz, 2H, H-3b), 2.22 (t, *J*= 7.3 Hz, 2H, H-3f), 1.59-1.49 (m, 4H, H-3c,3e), 1.42 (t, *J*= 7.0 Hz, 3H, H-8b), 1.38-1.29 (m, 2H, H-3d); ^{13}C NMR (125 MHz, DMSO-d$_6$) δ 174.84, 161.45, 160.63, 147.96, 145.97, 143.74, 125.49, 121.55, 119.60, 117.31, 65.03, 39.43, 34.05, 29.15, 26.44, 24.64, 15.01; HRMS (ESI) *m/z* calcd. for $C_{18}H_{21}NO_6$ $[M+H]^+$ 348.1442, found 348.1452.

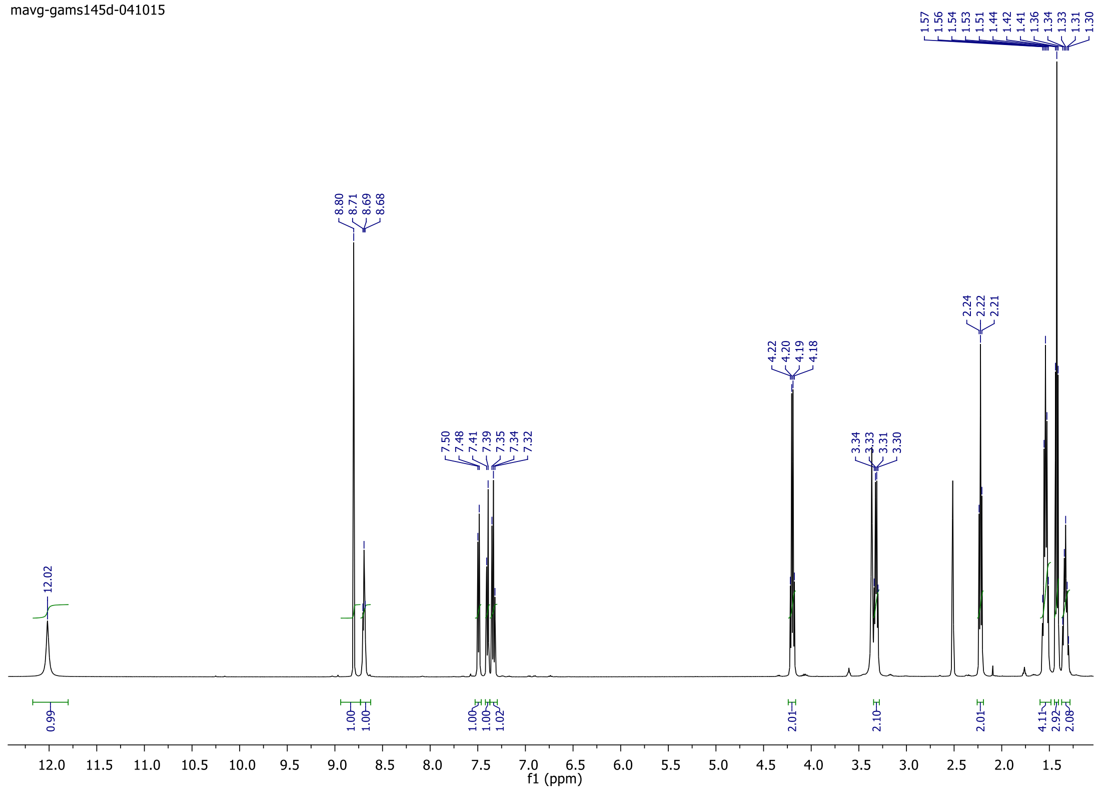

^{1}H spectrum of **6f** in DMSO-d$_6$, 500 MHz

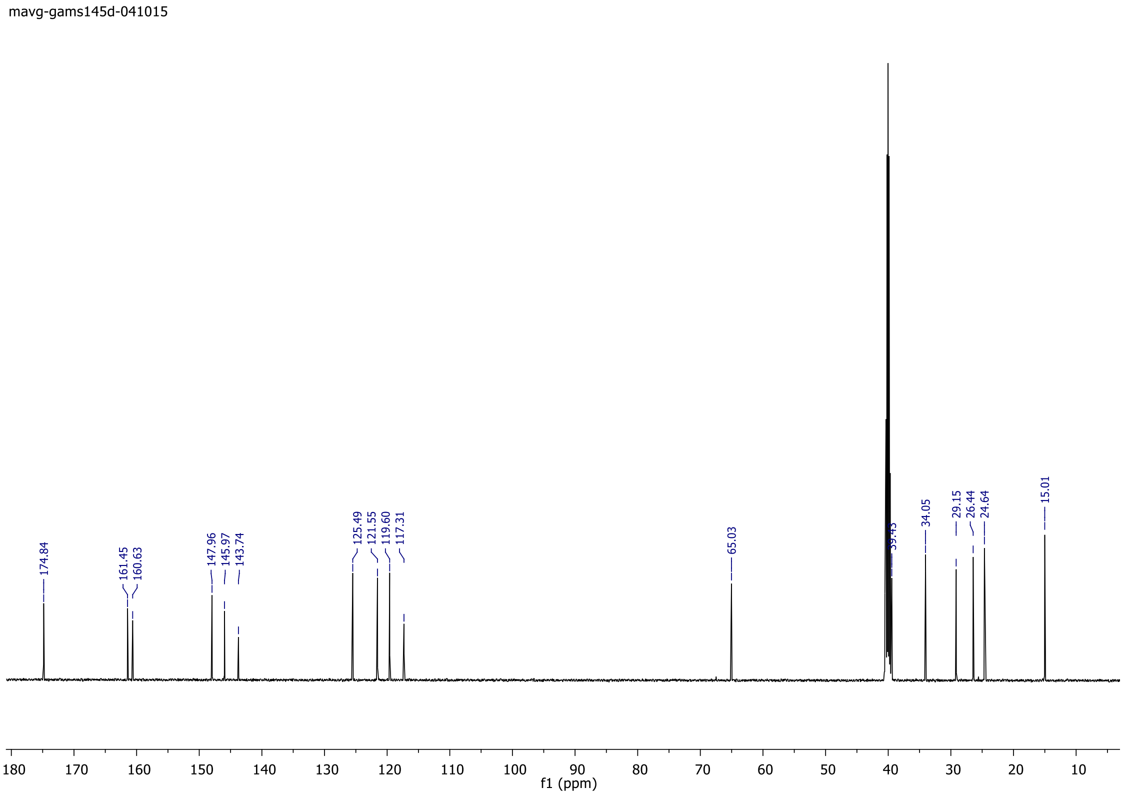

^{13}C spectrum of **6f** in DMSO-d_6, 125 MHz

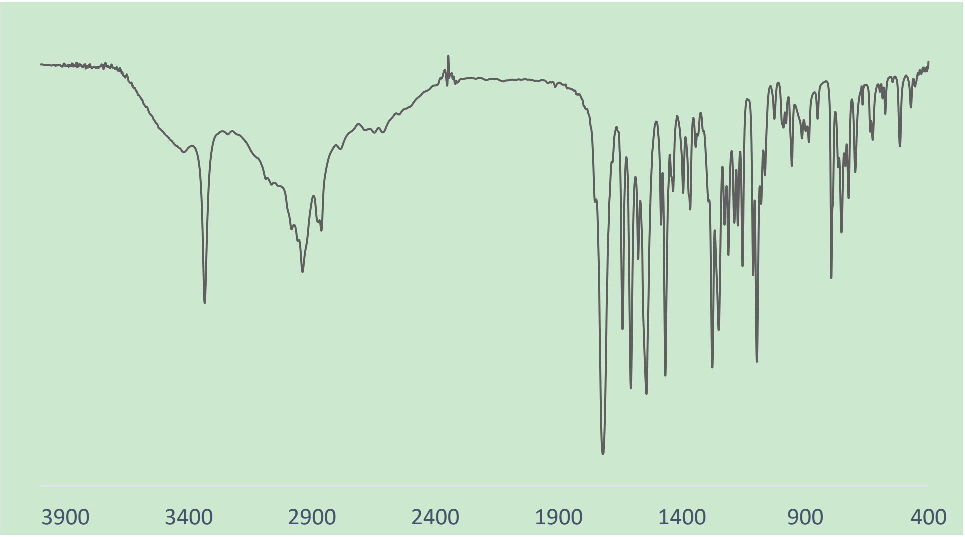

IR spectrum of **6f** in KBr

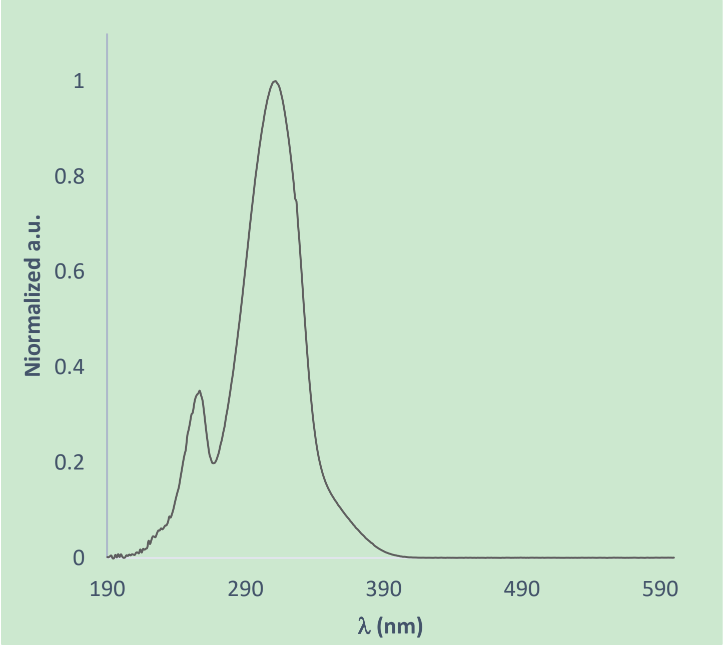

UV spectrum of **6f** in MeOH

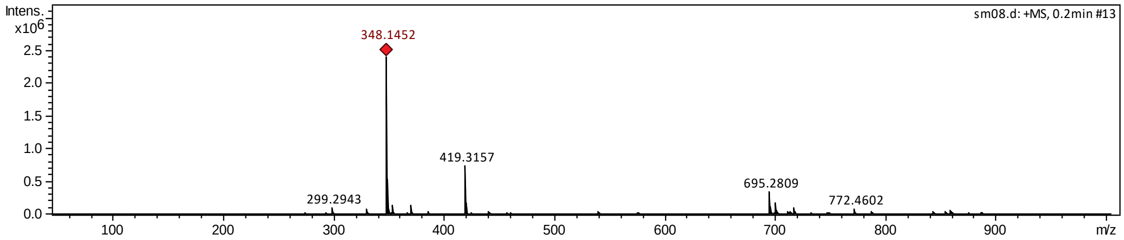

Mass spectrum, ESI(+), of **6f**

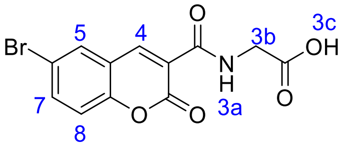

(6g) White solid (83% yield); mp 269-270 ºC; IR(KBr) $\nu_{max/cm-1}$= 3341, 1726, 1648, 1560; UV-vis (MeOH) $\lambda_{max/nm}$=291; ^{1}H NMR (500 MHz, DMSO-d$_6$) δ 12.84 (s, 1H, H-3c), 9.02 (t, *J*= 5.5 Hz, 1H, H-3a), 8.87 (s, 1H, H-4), 8.27 (d, *J*= 2.3 Hz, 1H, H-5), 7.90 (dd, *J*= 8.8, 2.3 Hz, 1H, H-7), 7.49 (d, *J*= 8.9 Hz, 1H, H-8), 4.07 (d, *J*= 5.5 Hz, 2H, H-3b). ^{13}C NMR (125 MHz, DMSO-d$_6$) δ 171.16, 161.32, 160.33, 153.48, 147.25, 136.87, 132.70, 120.75, 119.79, 118.89, 117.14, 42.00. HRMS (ESI) *m/z* calcd. for $C_{12}H_8NO_5$ $[M+H]^+$ 325.9659, found 325.9668.

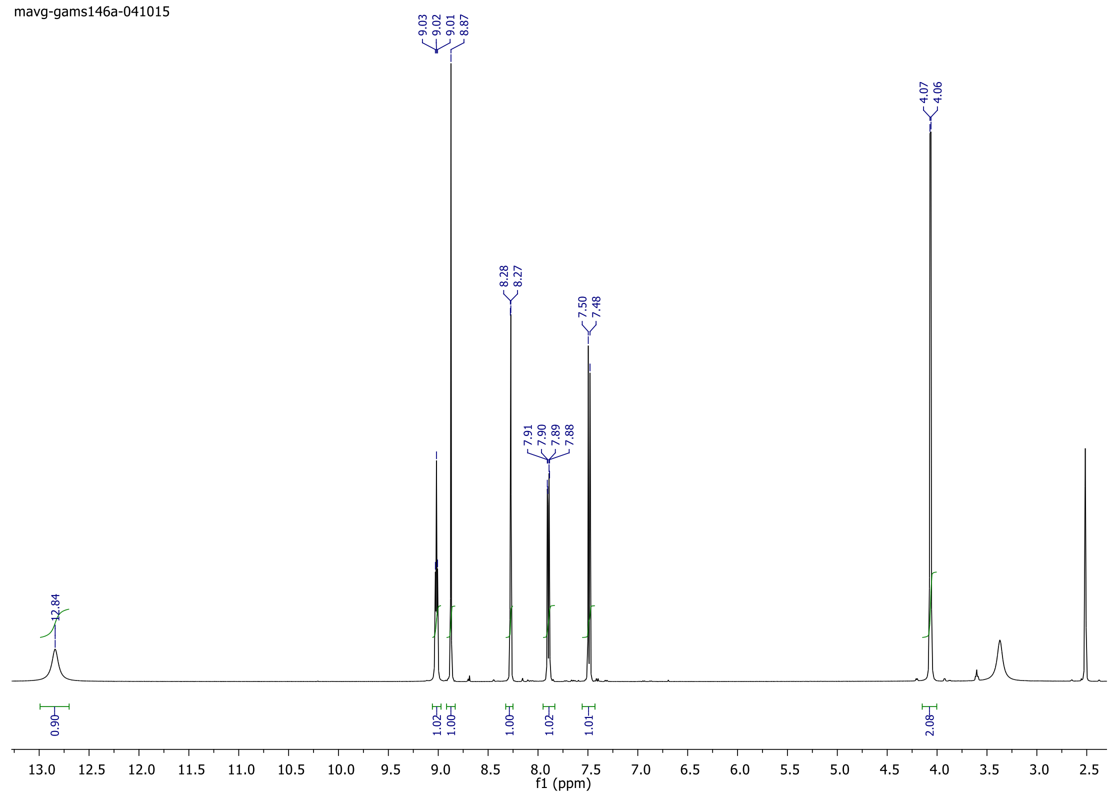

^{1}H spectrum of **6g** in DMSO-d_6, 500 MHz

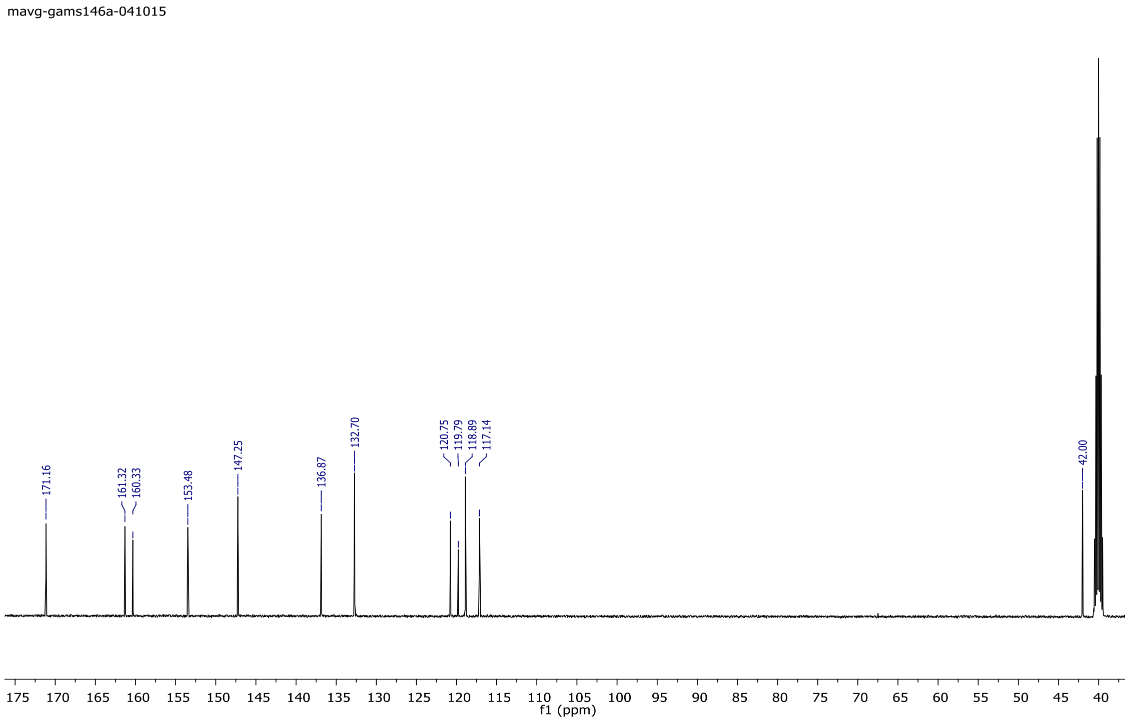

^{13}C spectrum of **6g** in DMSO-d_6, 125 MHz

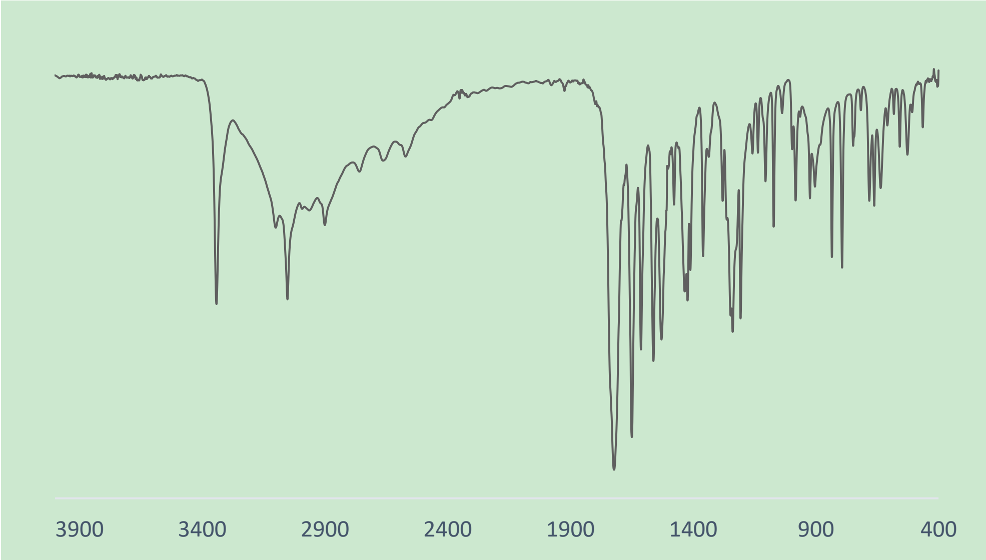

IR spectrum of **6g** in KBr

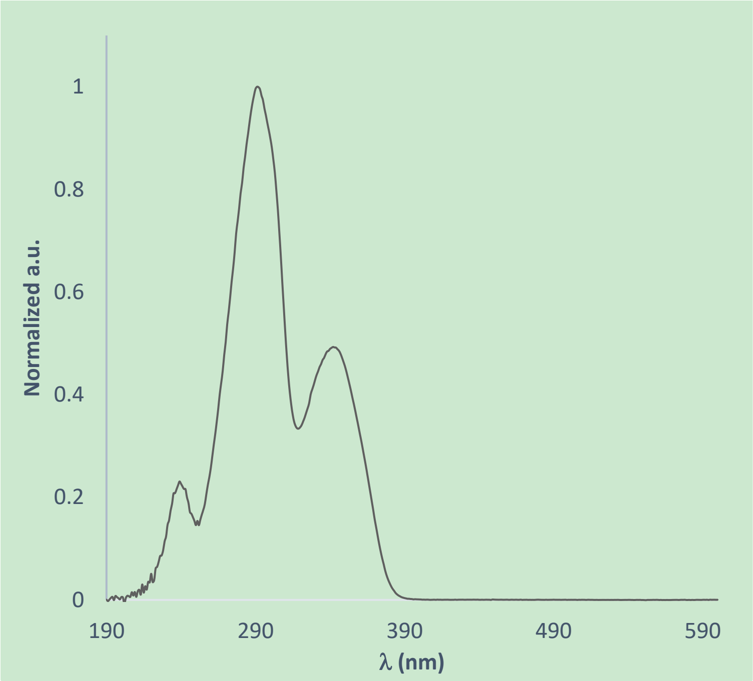

UV spectrum of **6g** in MeOH

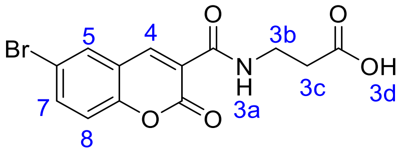

(6h) White solid (50% yield); mp 251-252 ºC; IR(KBr) $\nu_{max/cm-1}$= 3380, 1705, 1657, 1533; UV-vis (MeOH) $\lambda_{max/nm}$= 292; ^{1}H NMR (500 MHz, DMSO-d$_6$) δ 12.39 (s, 1H, H-3d), 8.88 (t, *J*= 5.8 Hz, 1H, H-3a), 8.84 (s, 1H, H-4), 8.25 (d, *J*= 2.2 Hz, 1H, H-5), 7.88 (dd, *J*= 8.8, 2.3 Hz, 1H, H-7), 7.47 (d, *J*= 8.9 Hz, 1H, H-8), 3.54 (q, *J*= 6.4 Hz, 2H, H-3b), 2.54 (t, *J*= 6.7 Hz, 2H, H-3c); ^{13}C NMR (125 MHz, DMSO-d$_6$) δ 173.50, 161.16, 160.35, 153.38, 146.79, 136.70, 132.59, 120.80, 120.25, 118.85, 117.10, 35.62, 34.11; HRMS (ESI) *m/z* calcd. for $C_{13}H_{10}NO_5$ $[M+H]^+$ 339.9815, found 339.9817.

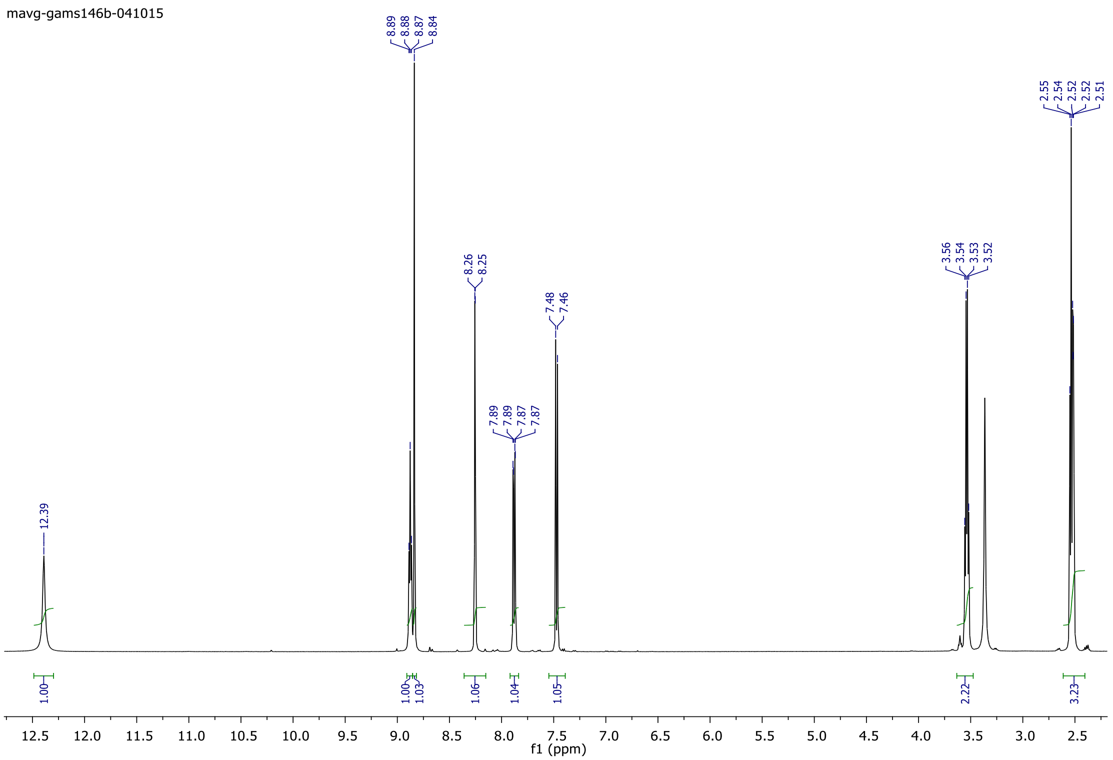

^{1}H spectrum of **6h** in DMSO-d$_6$, 500 MHz

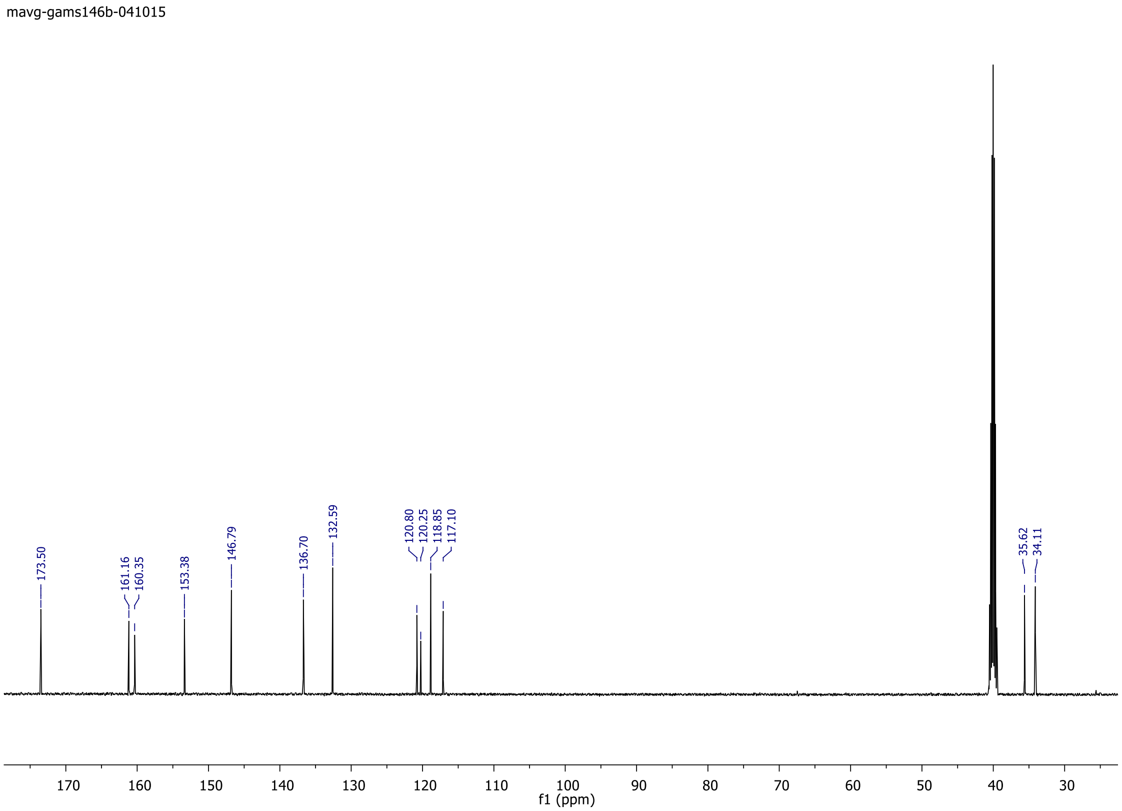

^{13}C spectrum of **6h** in DMSO-d$_6$, 125 MHz

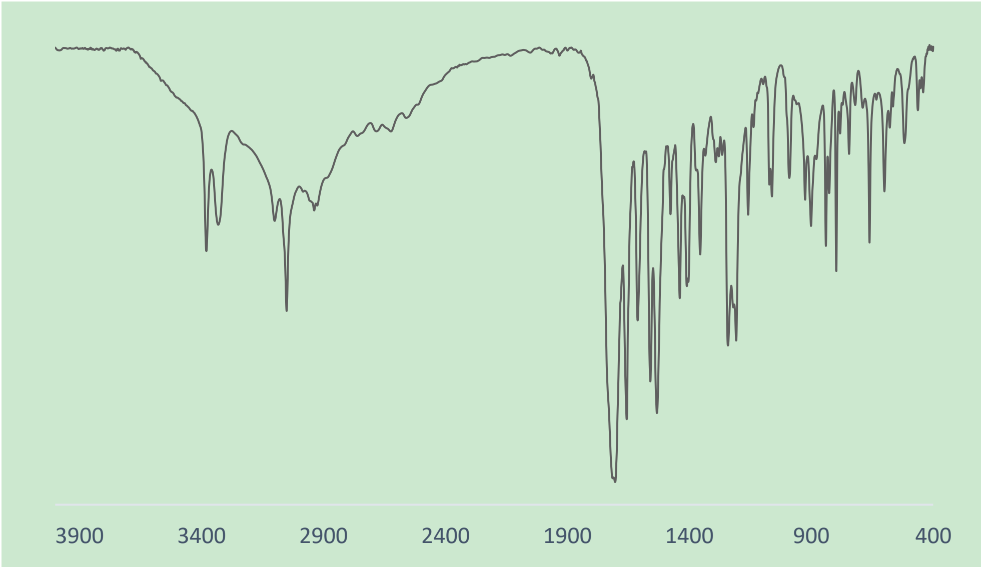

IR spectrum of **6h** in KBr

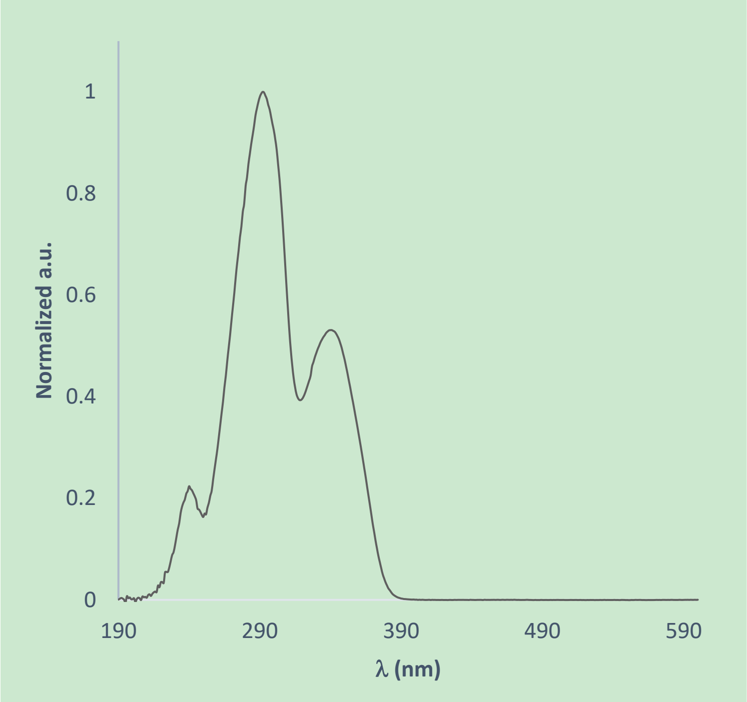

UV spectrum of **6h** in MeOH

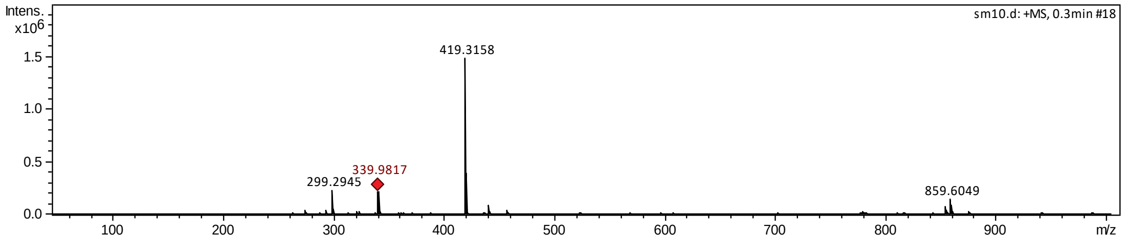

Mass spectrum, ESI(+), of **6h**

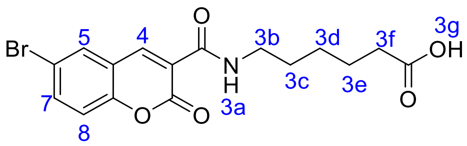

(6i) White solid (93% yield); mp 182-183 ºC; IR(KBr) $\nu_{max/cm-1}$= 3347, 1715, 1657, 1562; UV-vis (MeOH) $\lambda_{max/nm}$= 293; ^{1}H NMR (500 MHz, DMSO-d$_6$) δ 12.01 (s, 1H, H-3g), 8.80 (s, 1H, H-4), 8.66 (t, *J*= 5.7 Hz, 1H, H-3a), 8.25 (d, *J*= 2.2 Hz, 1H, H-5), 7.88 (dd, *J*= 8.8, 2.3 Hz, 1H, H-7), 7.47 (d, *J*= 8.8 Hz, 1H, H-8), 3.31 (dd, *J*= 13.1, 6.7 Hz, 2H, H-3b), 2.22 (t, *J*= 7.4 Hz, 2H, H-3f), 1.59-1.47 (m, 4H, H-3c,3e), 1.37-1.28 (m, 2H, H-3d); ^{13}C NMR (125 MHz, DMSO-d$_6$) δ 174.84, 161.18, 160.32, 153.33, 146.37, 136.59, 132.51, 120.83, 120.78, 118.85, 117.08, 39.47, 34.05, 29.11, 26.42, 24.64; HRMS (ESI) *m/z* calcd. for $C_{16}H_{16}NO_5$ $[M+H]^+$ 382.0212, found 382.0289.

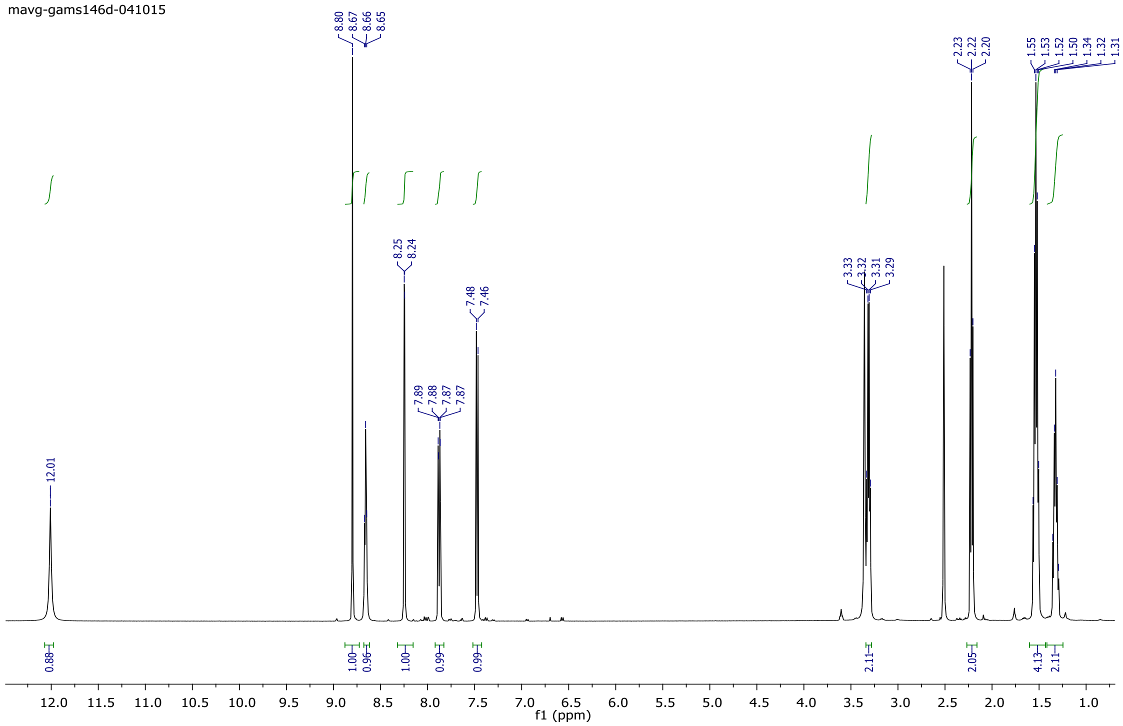

^{1}H spectrum of **6i** in DMSO-d_6, 500 MHz

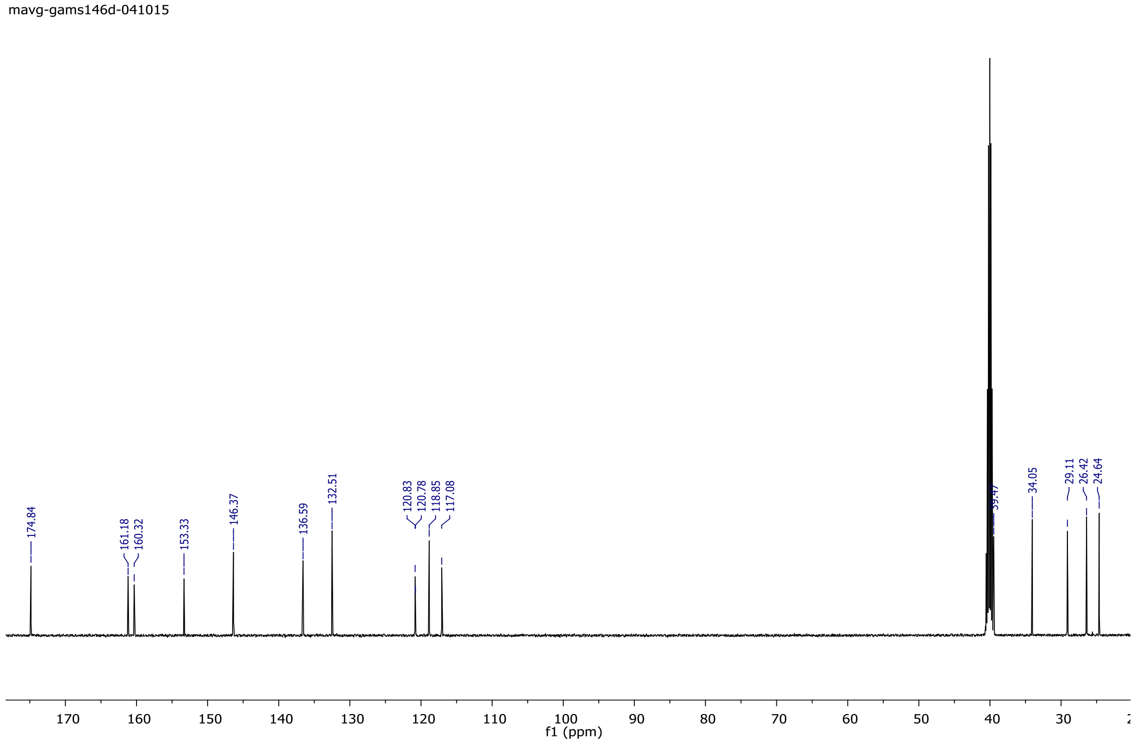

^{13}C spectrum of **6i** in DMSO-d_6, 125 MHz

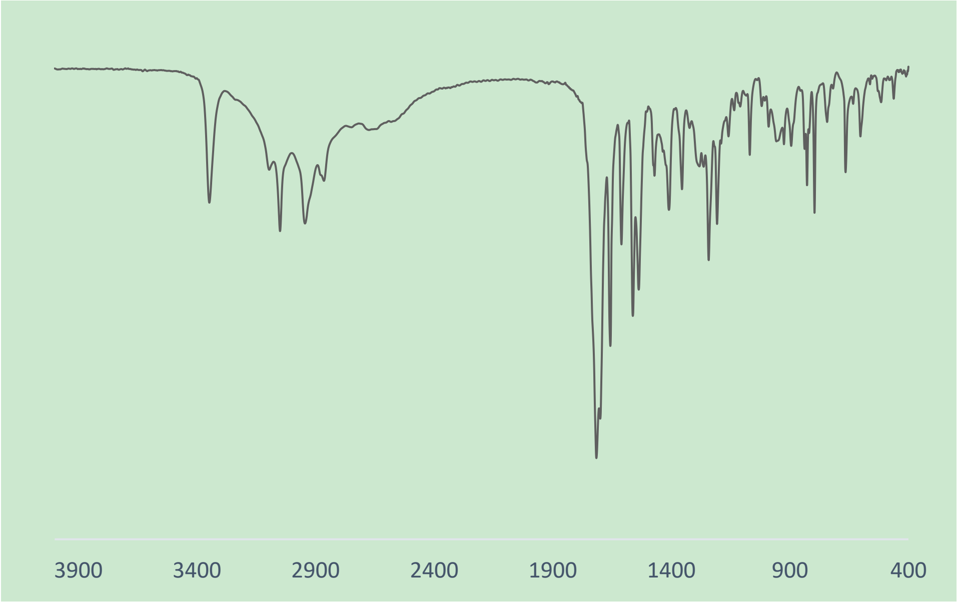

IR spectrum of **6i** in KBr

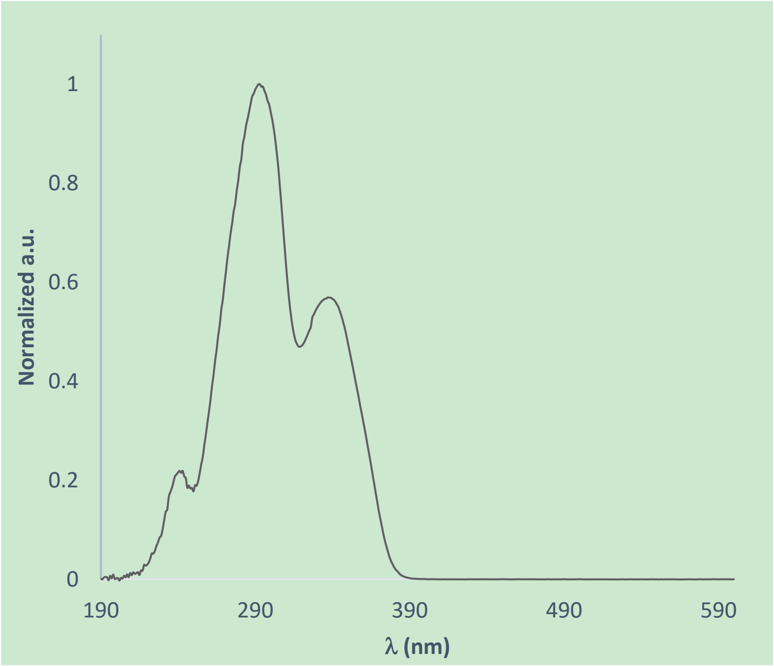

UV spectrum of **6i** in MeOH

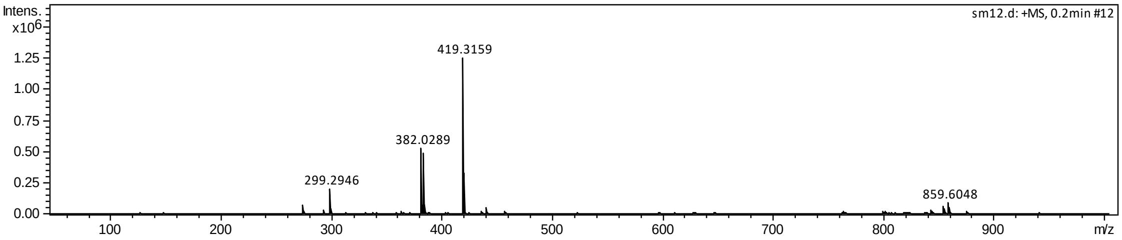

Mass spectrum, ESI(+), of **6i**

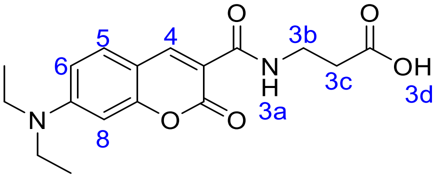

(6j) [3] Yellow crystals (75% yield); mp 202–205 °C; ^{1}H-NMR (DMSO-d_6, 500 MHz) δ 8.84 (t, *J*= 5.1 Hz, 1H), 8.65 (s, 1H), 7.66 (d, *J*= 8.9 Hz, 1H), 6.78 (d, *J*= 8.6 Hz, 1H), 6.59 (s, 1H), 3.54-3.50 (m, 2H), 3.47 (dd, *J*= 13.7, 6.8 Hz, 4H), 2.49 (t, *J*= 6.2 Hz, 2H), 1.14 (t, *J*= 6.9 Hz, 6H).

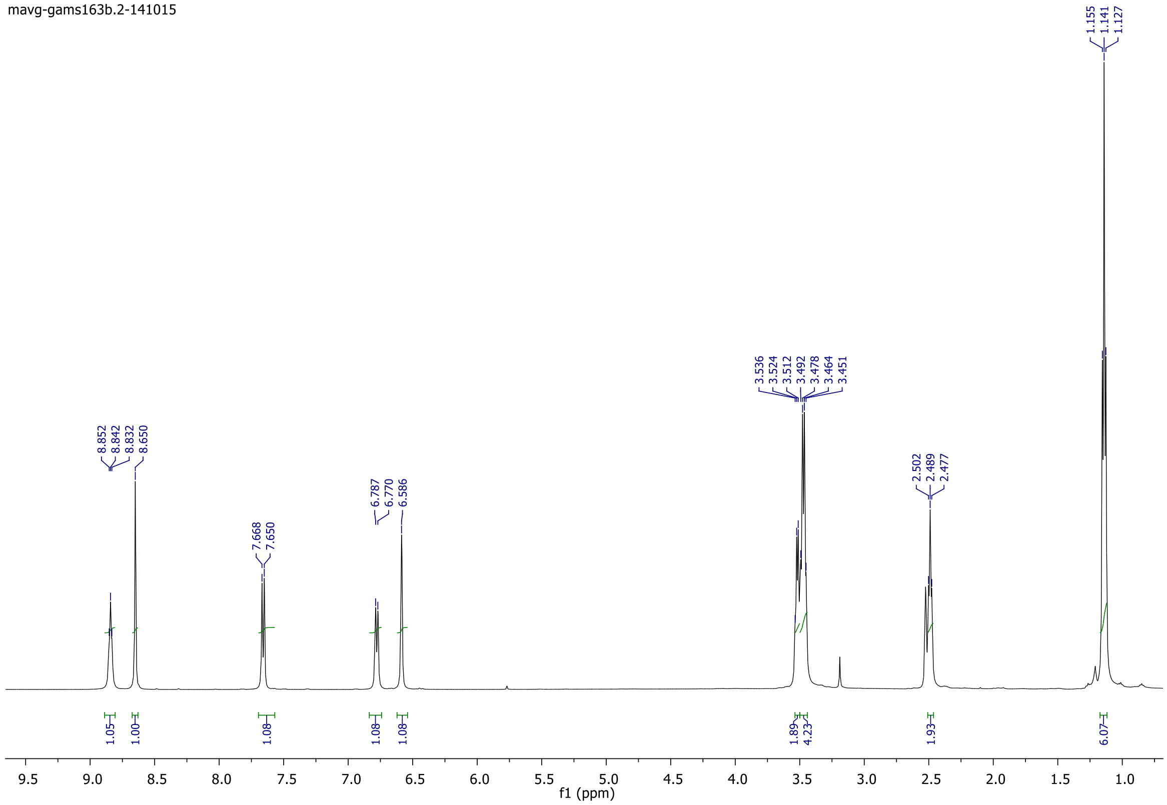

^{1}H spectrum of **6j** in DMSO-d_6, 500 MHz

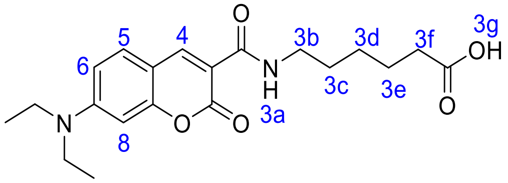

(6k) [3] Yellow crystals (73% yield); mp 150-152 °C; ^{1}H-NMR (DMSO-d_6, 500 MHz) δ 8.67-8.62 (m, 2H), 7.68 (d, *J*= 9.0 Hz, 1H), 6.80 (dd, *J*= 9.0, 2.2 Hz, 1H), 6.62 (d, *J*= 2.0 Hz, 1H), 3.48 (dd, *J*= 14.0, 7.0 Hz, 4H), 3.31-3.27 (m, 2H), 2.21 (t, *J*= 7.3 Hz, 2H), 1.51 (dq, *J*= 14.8, 7.4 Hz, 4H), 1.31 (dd, *J*= 15.2, 8.1 Hz, 2H), 1.14 (t, *J*= 7.0 Hz, 6H).

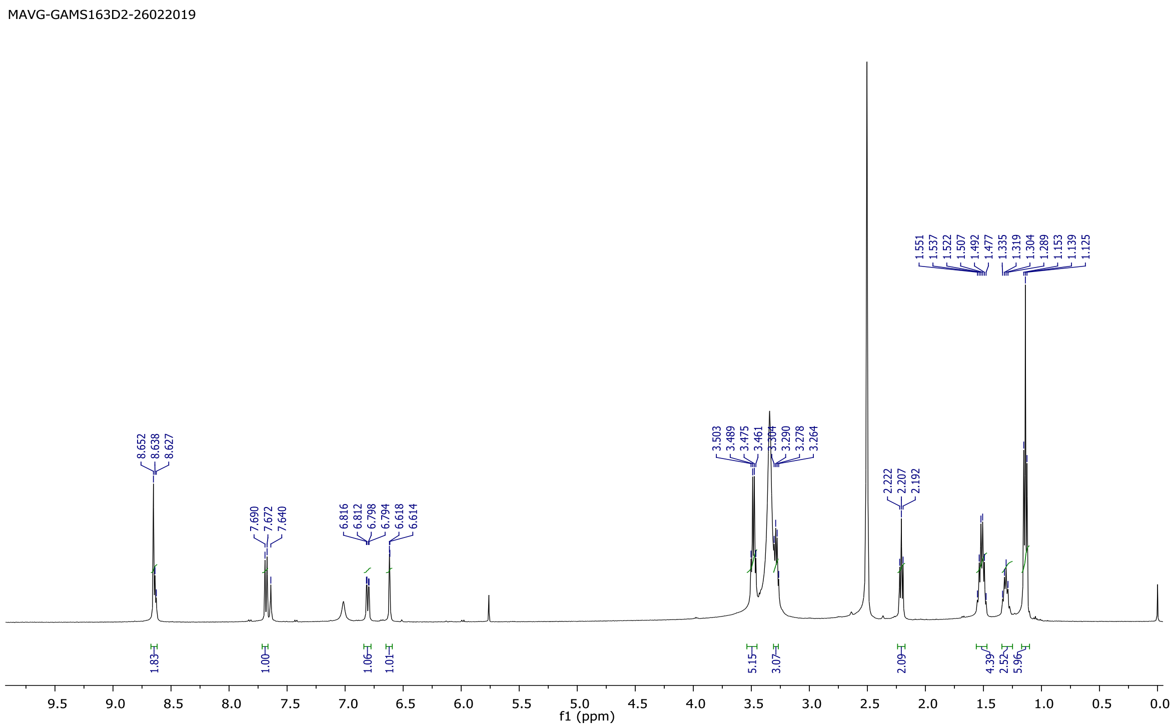

^{1}H spectrum of **6k** in DMSO-d$_6$, 500 MHz

General procedure for the preparation of the title compounds (7a-k)

In a dry round-bottom flask purged with N_2, the corresponding carboxylic acid (1 mmol), DMAP (5% mol) and CDI (1.1 mmol) were dissolved in DMF. After 30 min of stirring at room temperature, hydroxylamine hydrochloride was added and stirring continued overnight. Upon completion of the reaction, sodium bicarbonate was added. The resulting precipitate was obtained by filtration and purified by recrystallization with MeOH and cold water.

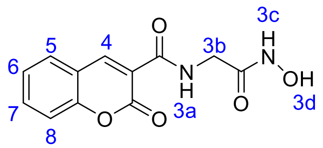

(7a) White solid (50% yield); mp 226-227 ºC; IR(KBr) $\nu_{max/cm-1}$= 3304, 3240, 3043, 1709, 1668; UV-vis (MeOH) $\lambda_{max/nm}$= 297; Em (MeOH) $\lambda_{max/nm}$= 406; ^{1}H NMR (400 MHz, DMSO-d$_6$) δ 10.68 (s, 1H), 9.04 (s, 1H), 8.90 (s, 1H), 8.00 (s, 1H), 7.76 (s, 1H), 7.61-7.35 (m, 2H), 3.92 (s, 2H); ^{13}C NMR (100 MHz, DMSO-d$_6$) δ 165.66, 161.61, 160.84, 154.38, 148.16, 134.71, 130.84, 125.64, 118.92, 118.88, 116.62, 41.09. HRMS (ESI) *m/z* calcd. for $C_{12}H_{10}N_2O_5$ $[M+Na]^+$ 285.0488, found 285.0505.

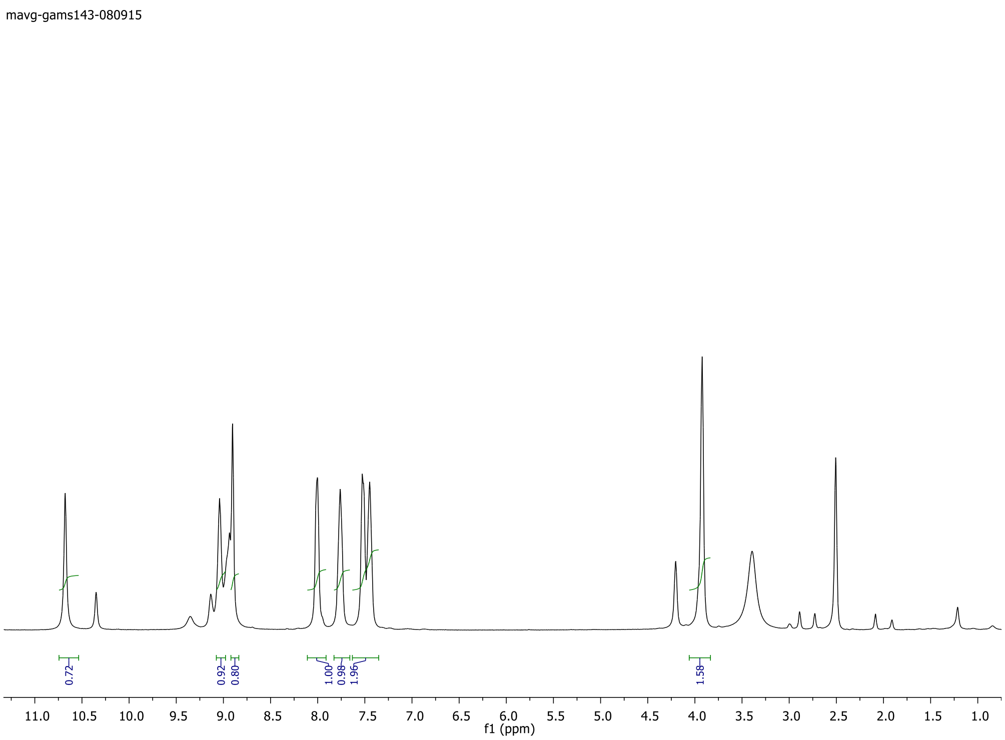

^{1}H spectrum of **7a** in DMSO-d$_6$, 500 MHz

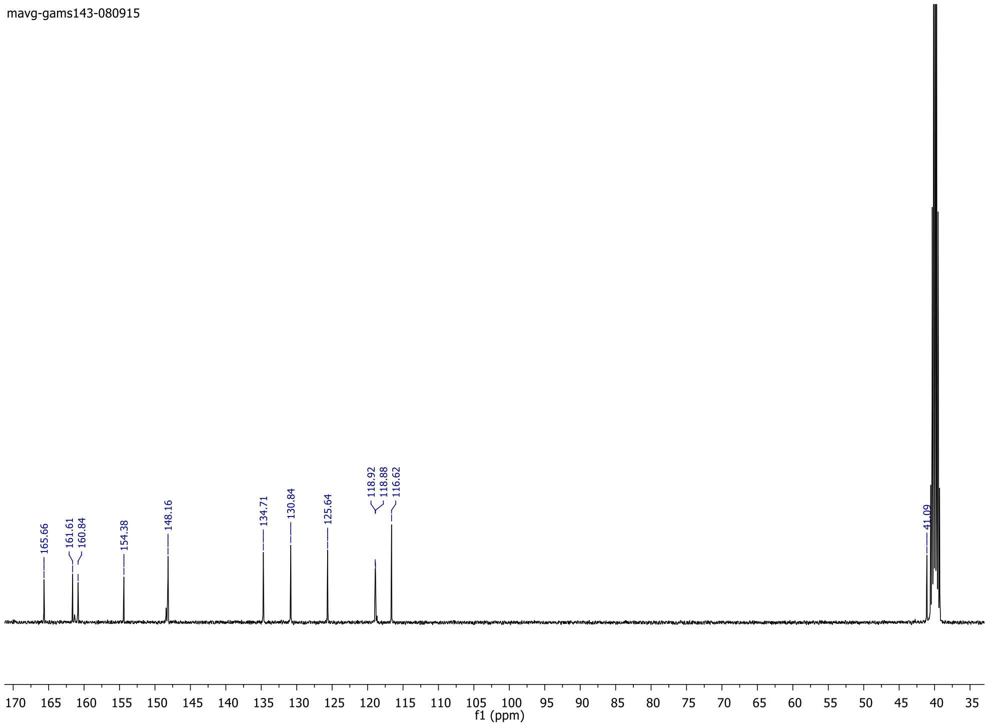

^{13}C spectrum of **7a** in DMSO-d$_6$, 125 MHz

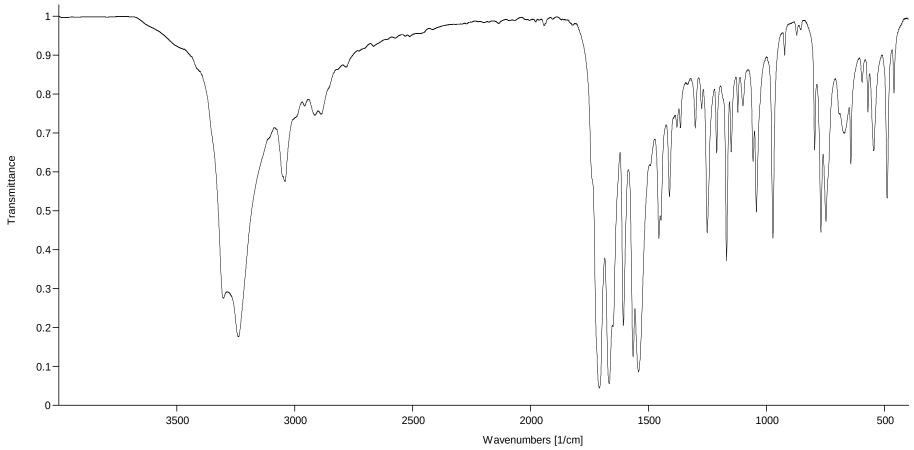

IR spectrum of **7a** in KBr

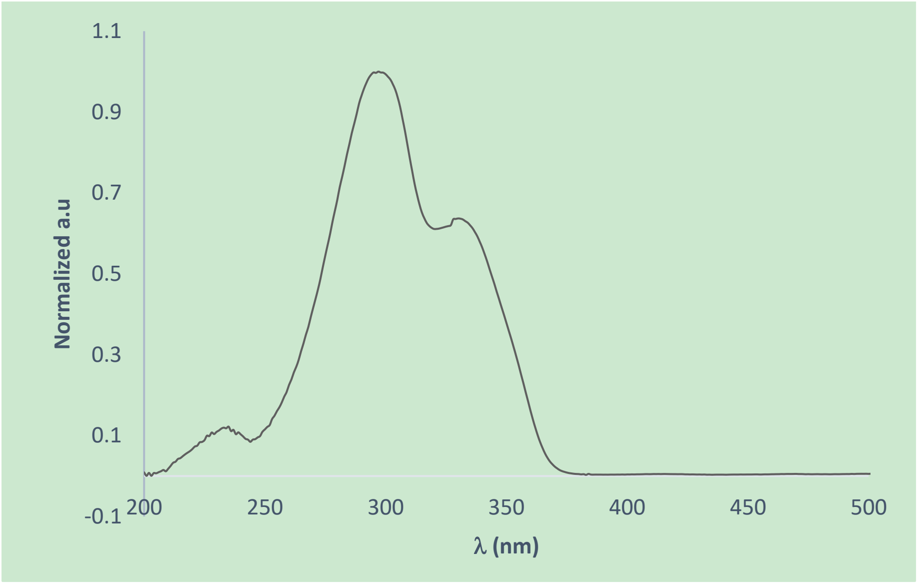

UV spectrum of **7a** in MeOH

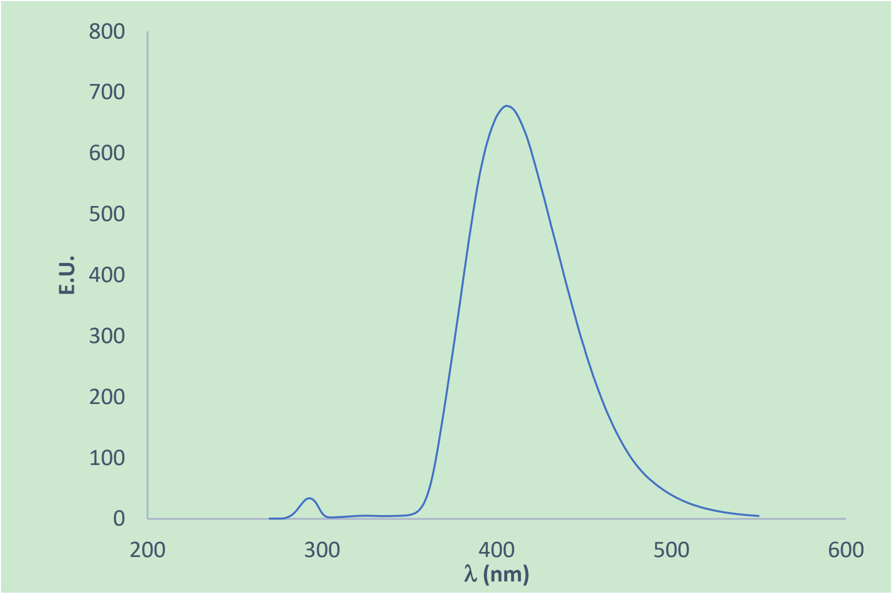

Emission spectrum of **7a** in MeOH at 10^{-6}M

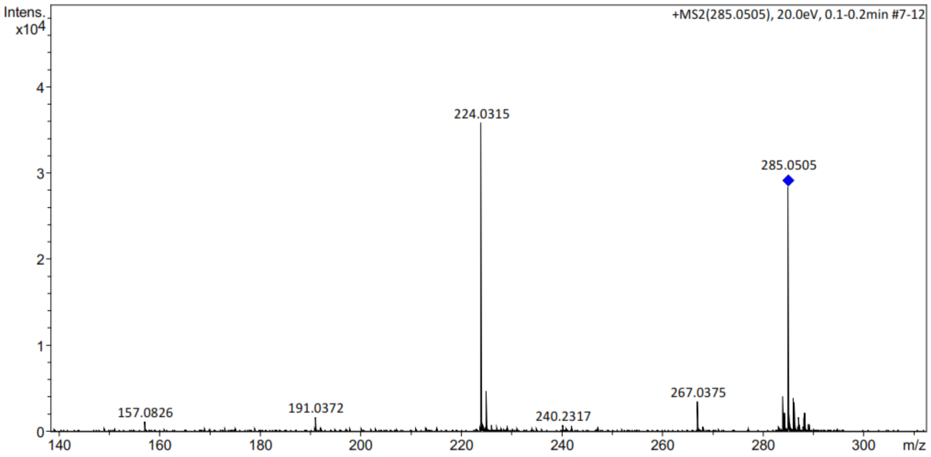

Mass spectrum, ESI(+), of **7a**

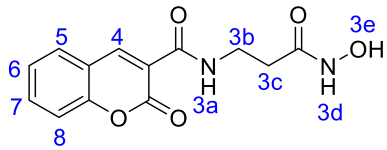

(7b) White solid (45% yield); mp 170-173 ºC; IR(KBr) $\nu_{max/cm-1}$= 3469, 3302, 3194, 1708, 1648 ;UV-vis (MeOH) $\lambda_{max/nm}$= 303.12; Em (MeOH) $\lambda_{max/nm}$= 405; ^{1}H NMR (500 MHz, DMSO-d$_6$) δ 8.88 (s, 1H), 8.87 (s,1H), 7.99 (d, *J*= 6.6 Hz, 1H), 7.76 (dd, *J*= 11.5, 4.2 Hz, 1H), 7.51 (d, *J*= 8.3 Hz, 1H), 7.45 (t, *J*= 7.2 Hz, 1H), 3.54 (dd, *J*= 12.7, 6.5 Hz, 2H), 2.29 (t, *J*= 6.7 Hz, 2H); ^{13}C NMR (125 MHz, DMSO-d$_6$) δ 167.78, 161.45, 160.78, 154.36, 148.09, 134.59, 130.76, 125.60, 119.11, 118.91, 116.60, 116.56, 36.12, 32.39. HRMS (ESI) *m/z* calcd. for $C_{13}H_{12}N_2O_5$ $[M+H]^+$ 277.0819, found 277.0860.

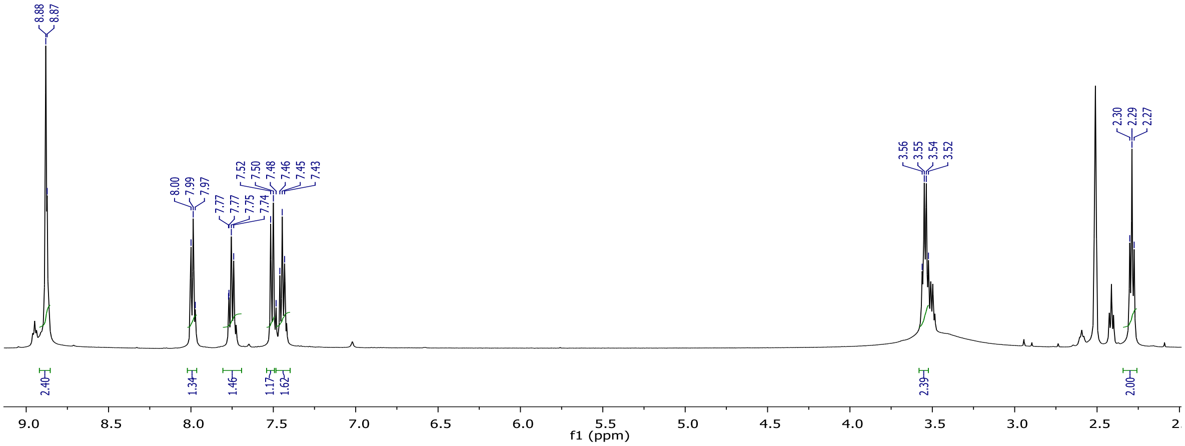

^{1}H spectrum of **7b** in DMSO-d$_6$, 500 MHz

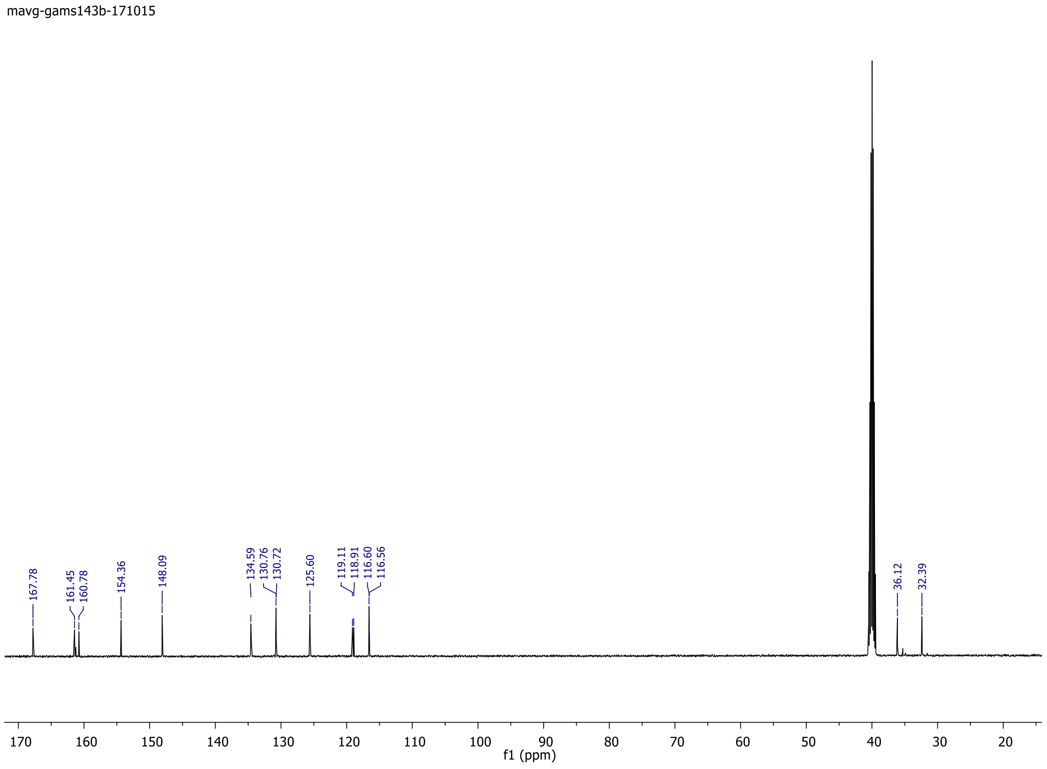

^{13}C spectrum of **7b** in DMSO-d$_6$, 125 MHz

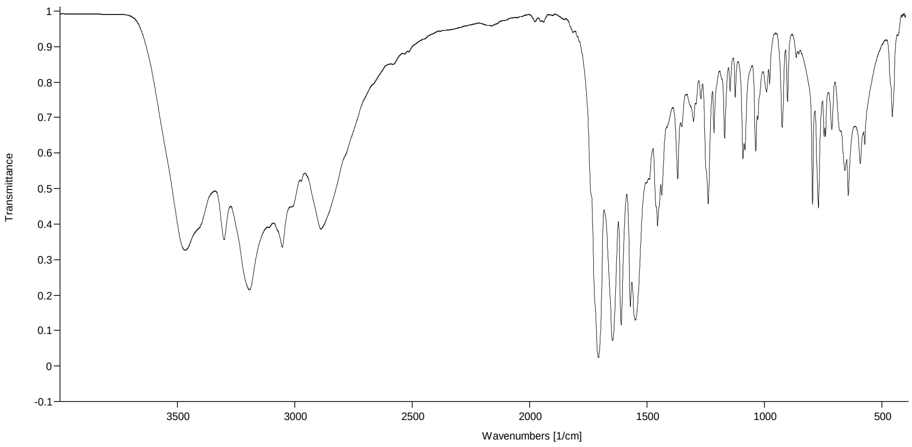

IR spectrum of **7b** in KBr

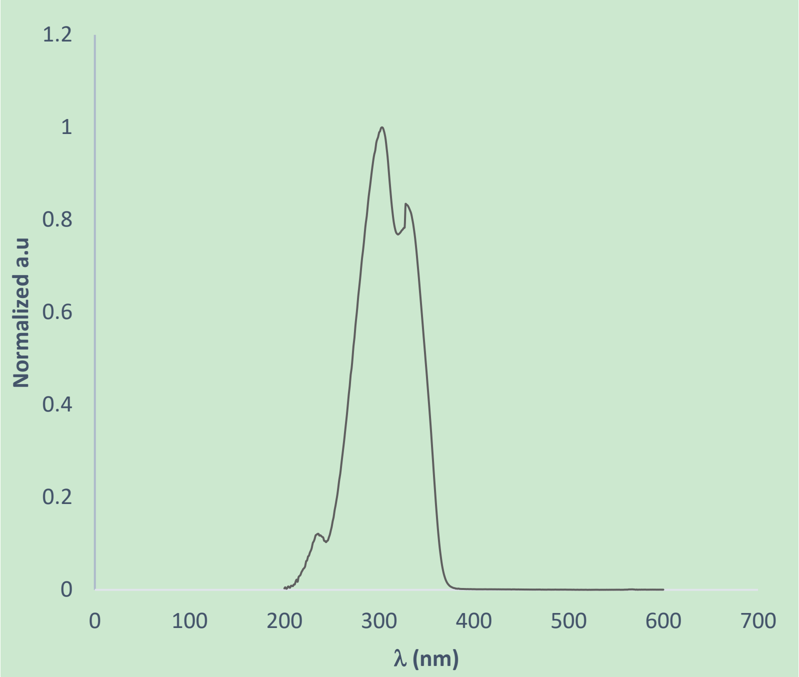

UV spectrum of **7b** in MeOH

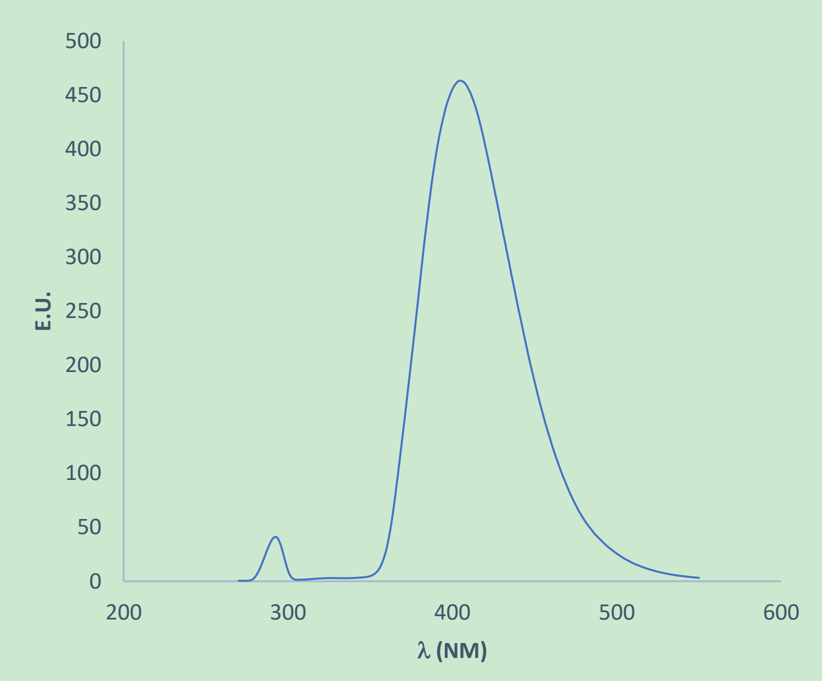

Emission spectrum of **7b** in MeOH at 10^{-6}M

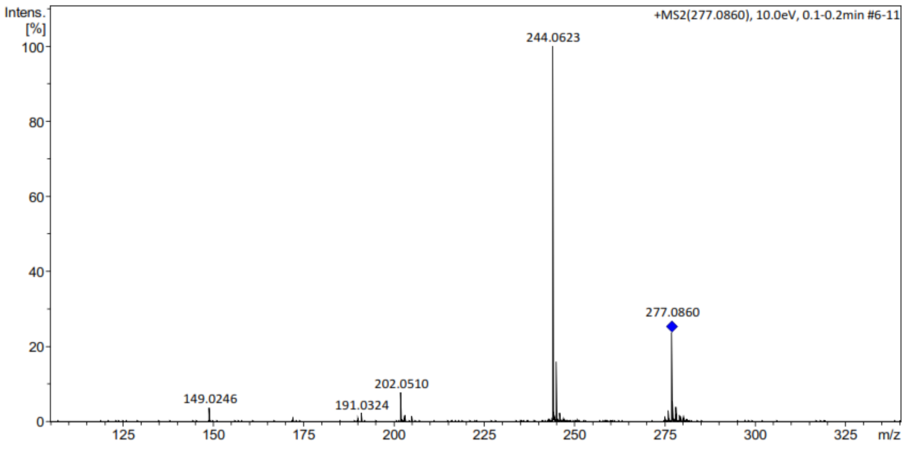

Mass spectrum, ESI(+), of **7b**

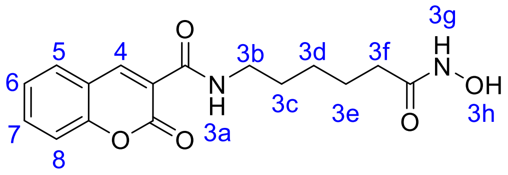

(7c) White solid (50% yield); mp 110-112 °C; IR(KBr) $\nu_{max/cm-1}$= 3336, 3207, 1706, 1537;UV-vis (MeOH) $\lambda_{max/nm}$=329.96; Em (MeOH) $\lambda_{max/nm}$= 405; ^{1}H NMR (500 MHz, DMSO-d_6) δ 8.84 (s, 1H), 8.68 (s, 1H), 7.97 (d, *J*= 6.3 Hz, 1H), 7.74 (s, 1H), 7.50 (d, *J*= 7.4 Hz, 1H), 7.44 (s, 2H), 3.31 (d, *J*= 3.4 Hz, 3H), 1.96 (s, 2H), 1.53 (s, 5H), 1.29 (s, 2H); ^{13}C NMR (125 MHz, DMSO-d_6) δ 169.47, 161.48, 160.85, 154.30, 147.71, 134.46, 130.66, 125.58, 119.59, 118.94, 116.57, 39.49, 32.66, 29.17, 26.50, 25.31; HRMS (ESI) *m/z* calcd. for $C_{16}H_{18}N_2O_5$ $[M+Na]^+$ 341.1114, found 341.1019.

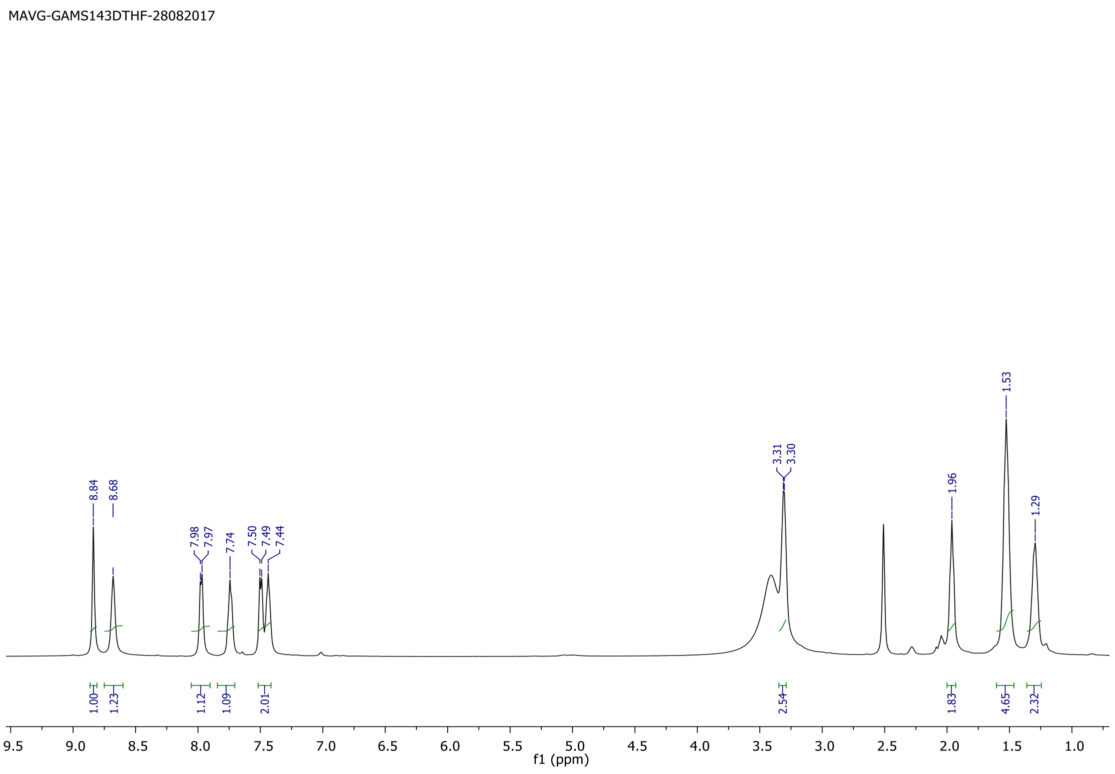

^{1}H spectrum of **7c** in DMSO-d_6, 125 MHz

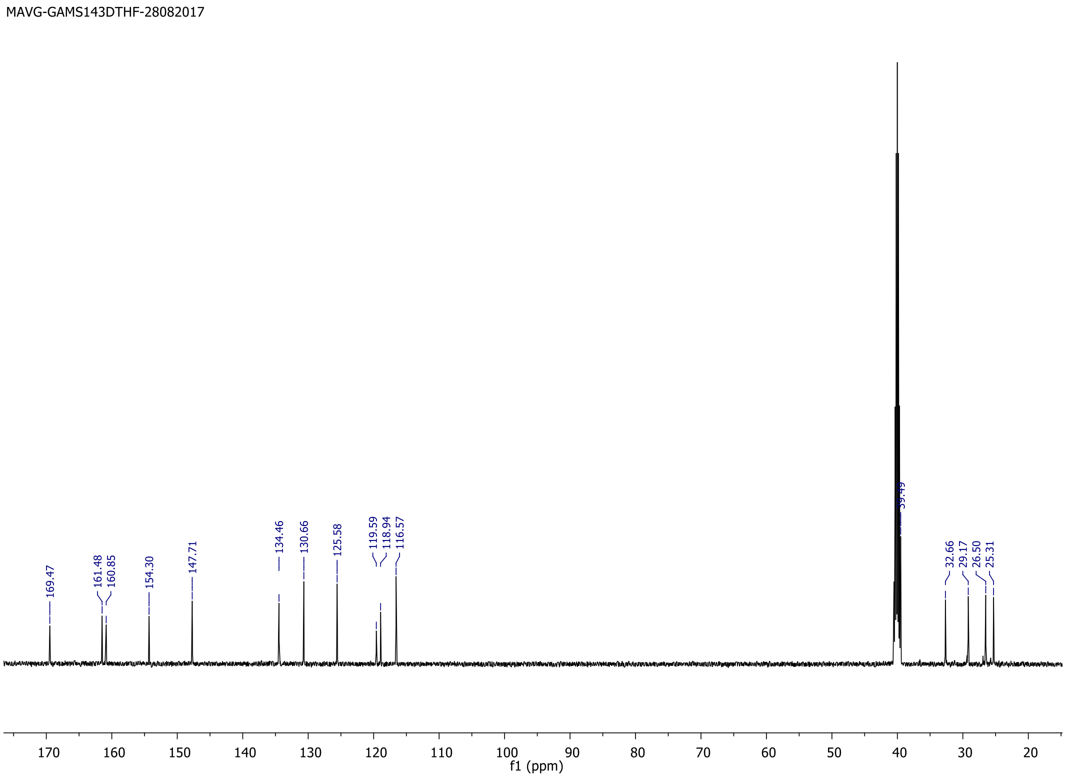

^{13}C spectrum of **7c** in DMSO-d_6, 125 MHz

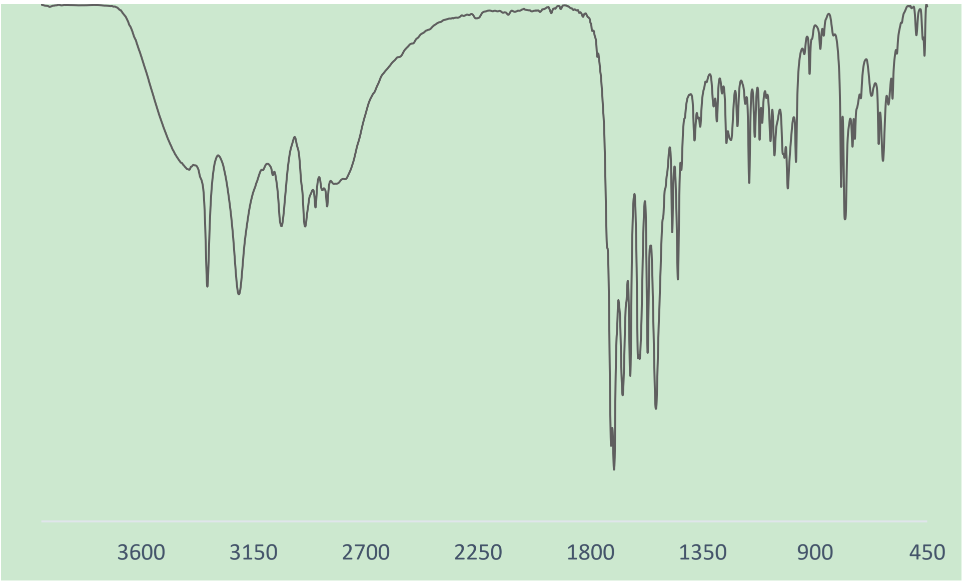

IR spectrum of **7c** in KBr

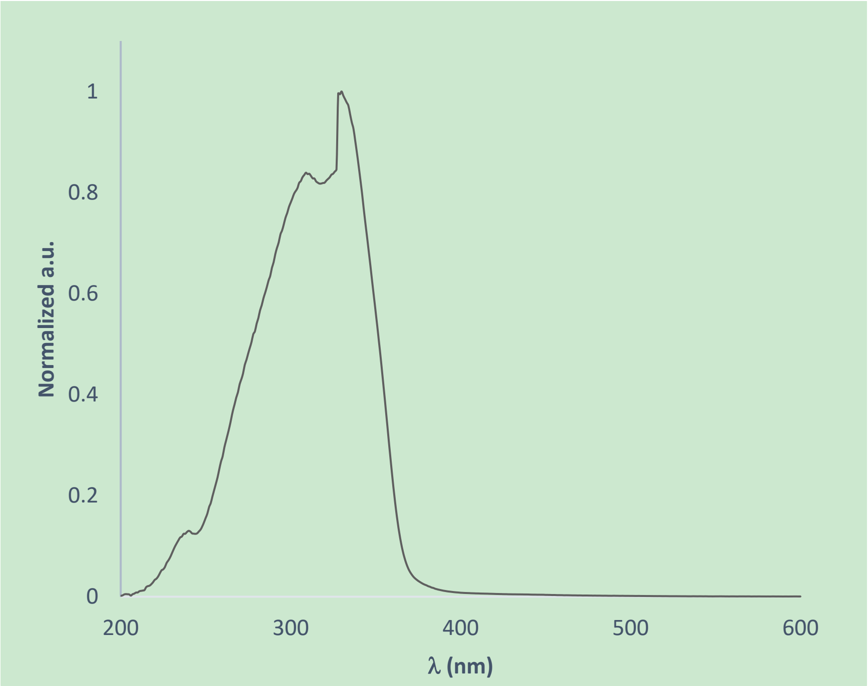

UV spectrum of **7c** in MeOH

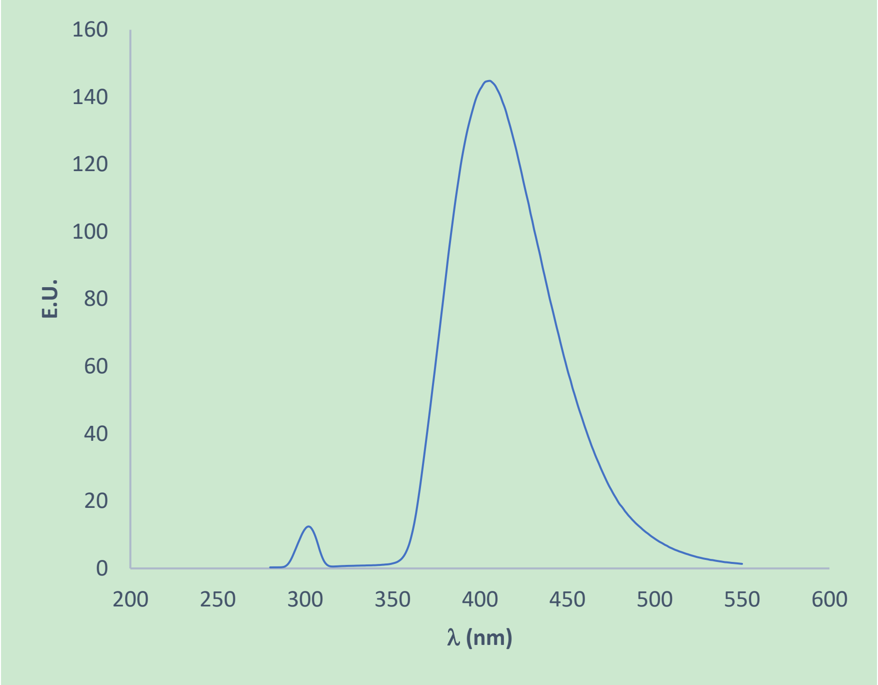

Emission spectrum of **7c** in MeOH at 10^{-6}M

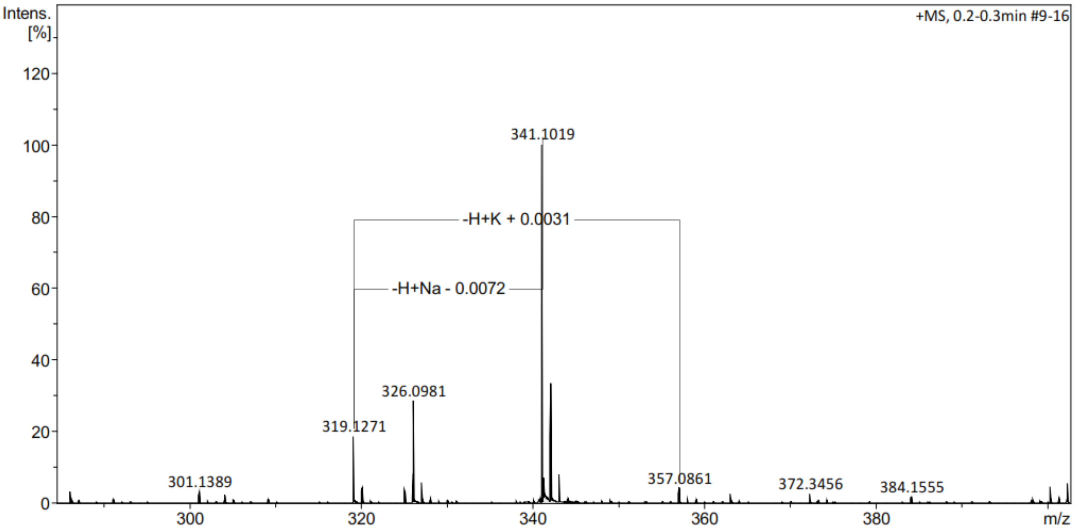

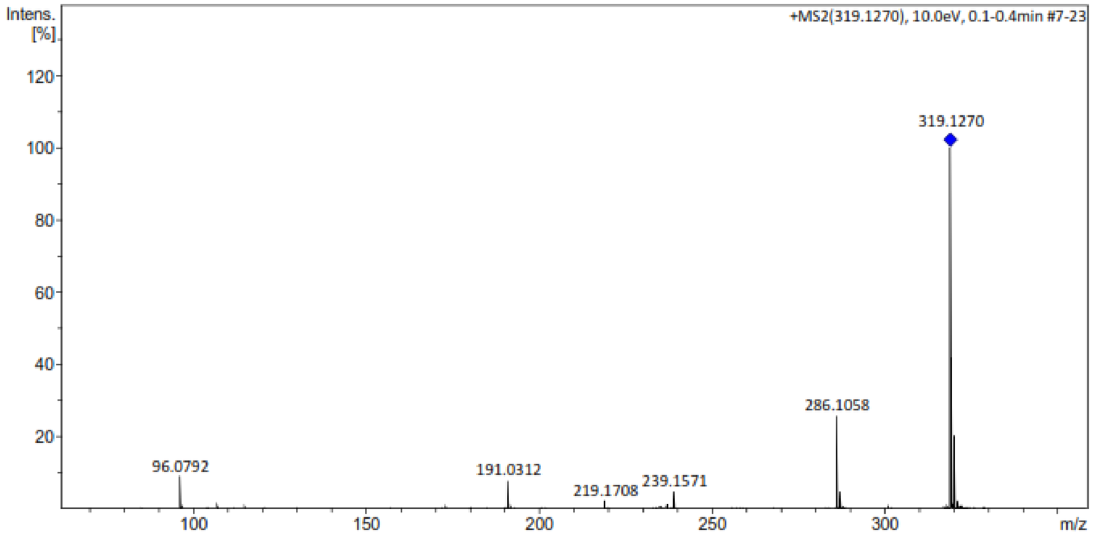

Mass spectrum, ESI(+), of **7c**

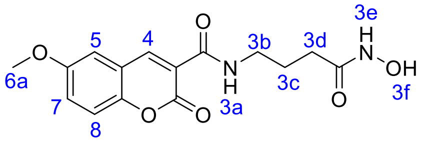

(7d) Light green solid (30% yield); mp 154-156 ºC; IR(KBr) $\nu_{max/cm-1}$= 3531, 3241, 1706, 1554; UV-vis (MeOH) $\lambda_{max/nm}$= 363; Em (MeOH) $\lambda_{max/nm}$= 458; ^{1}H NMR (500 MHz, DMSO-d_6) δ 8.80 (s, 1H), 8.75 (s, 1H), 7.54 (d, *J*= 2.2 Hz, 1H), 7.44 (d, *J*= 9.1 Hz, 1H), 7.32 (dd, *J*= 9.1, 2.5 Hz, 1H), 3.82 (s, 3H), 3.32 (dd, *J*= 13.1, 6.6 Hz, 2H), 2.03 (t, *J*= 7.5 Hz, 2H), 1.82-1.72 (m, 2H); ^{13}C NMR (125 MHz, DMSO-d_6) δ 169.09, 161.64, 160.89, 156.40, 148.79, 147.62, 122.35, 119.63, 119.37, 117.70, 112.25, 56.28, 39.22, 30.32, 25.72; HRMS (ESI) *m/z* calcd. for $C_{15}H_{16}N_2O_6$ $[M+H]^+$ 321.1081, found 321.1132.

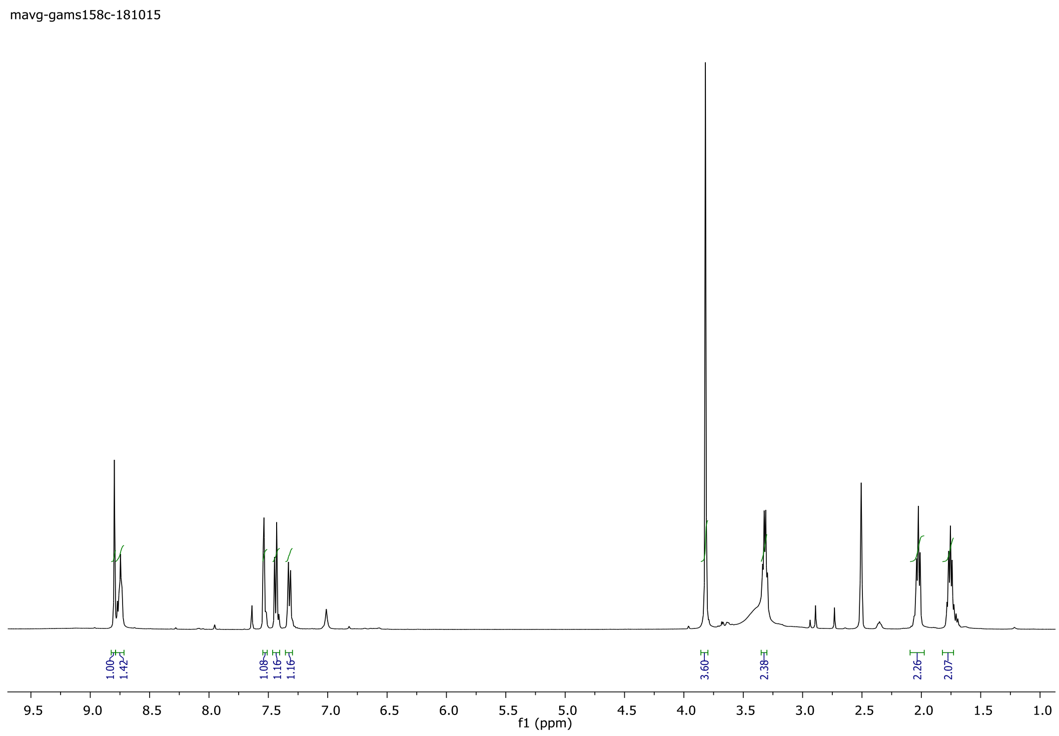

^{1}H spectrum of **7d** in DMSO-d_6, 500 MHz

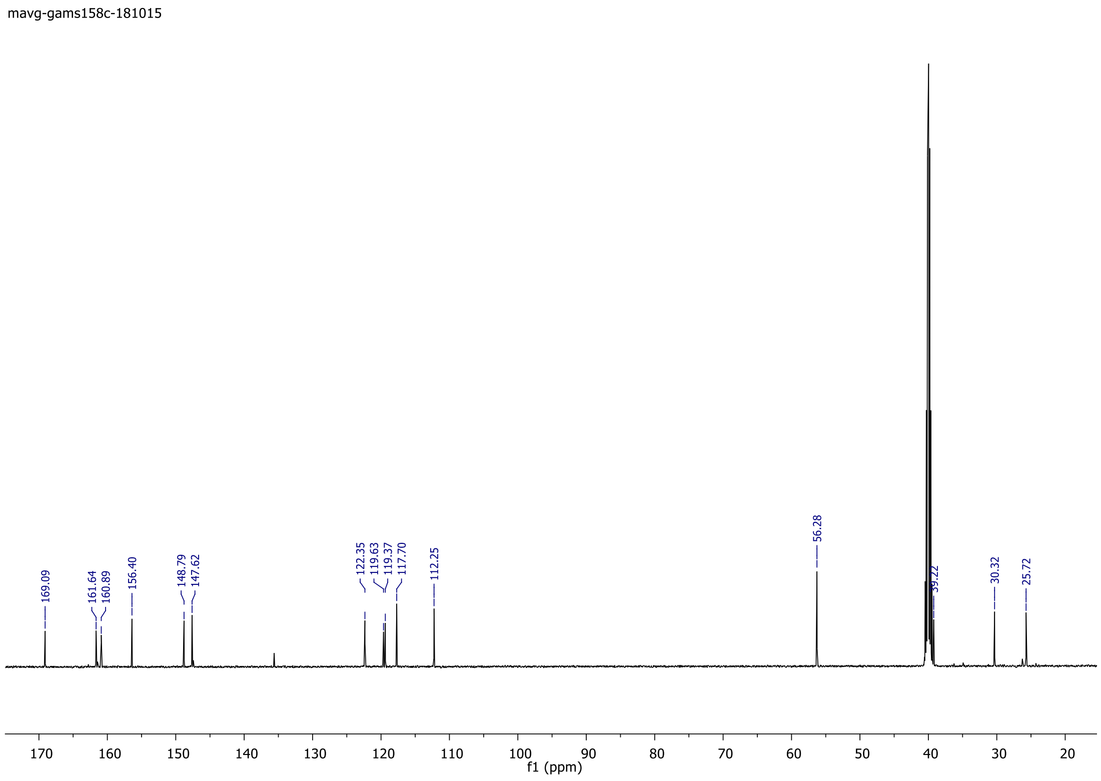

^{13}C spectrum of **7d** in DMSO-d_6, 125 MHz

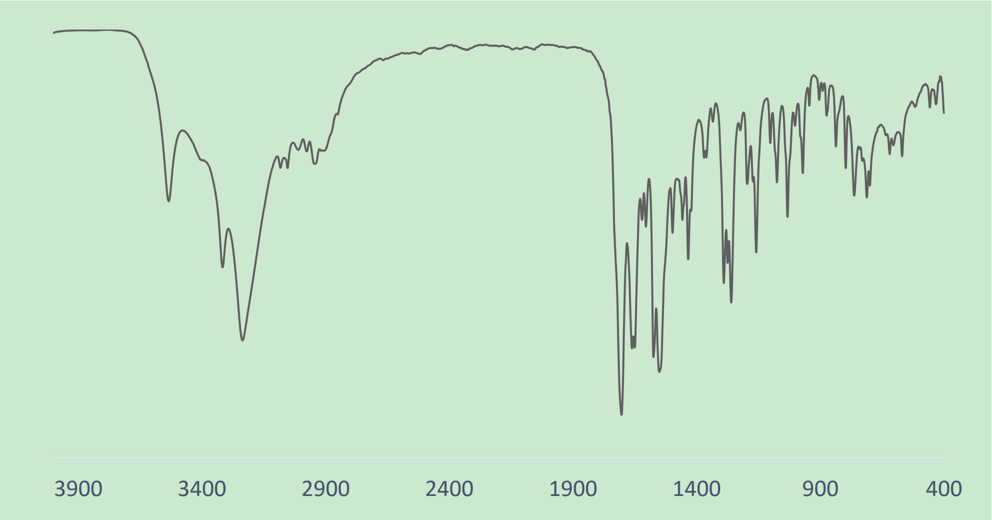

IR spectrum of **7d** in KBr

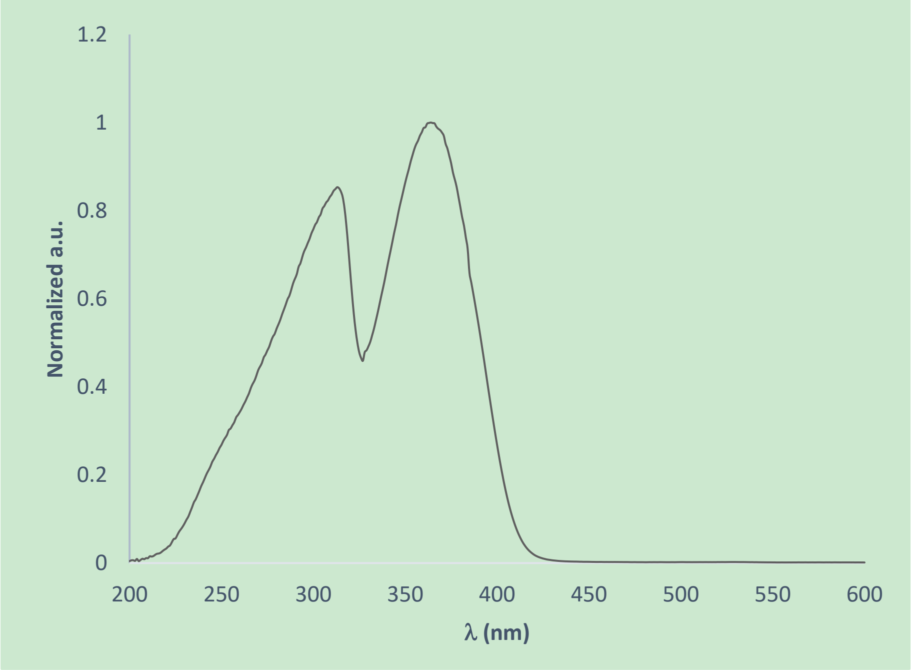

UV spectrum of **7d** in MeOH

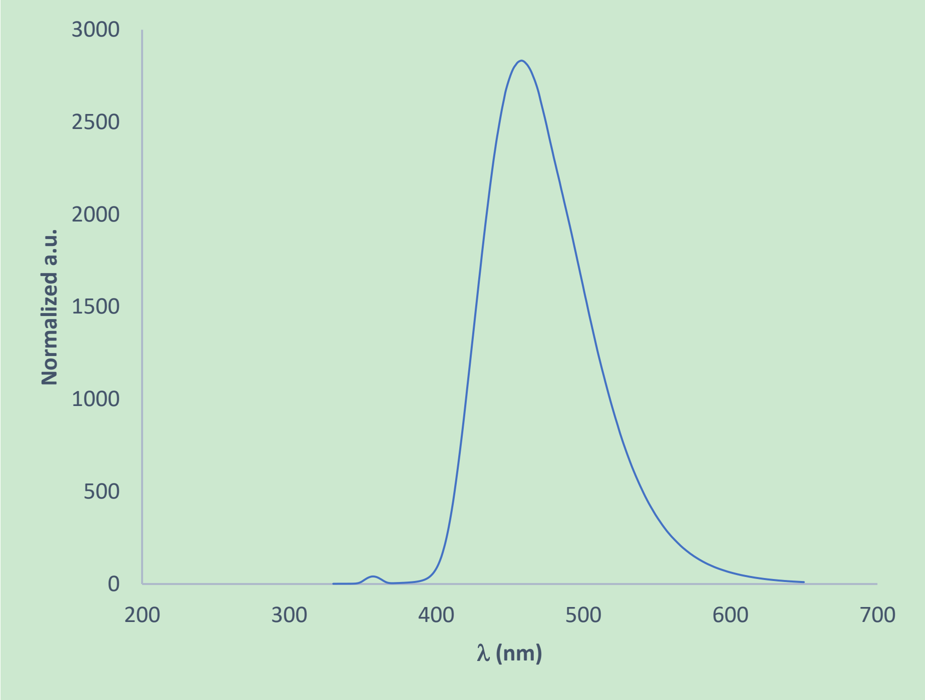

Emission spectrum of **7d** in MeOH at 10^{-6}M

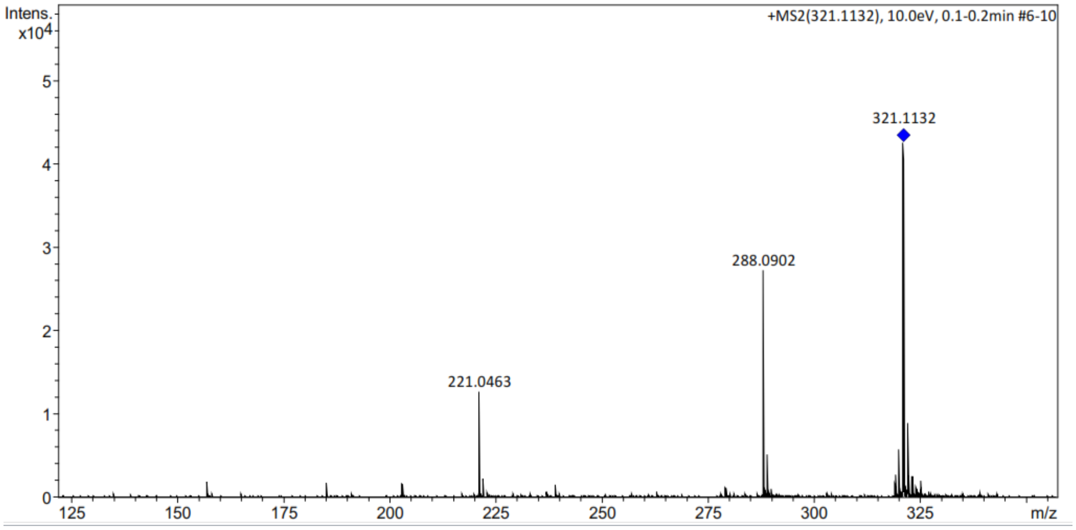

Mass spectrum, ESI(+), of **7d**

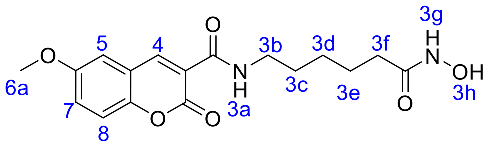

(7e) Light green solid (87% yield); mp 75-77 °C; IR(KBr) $\nu_{max/cm-1}$= 3318, 2935, 1702, 1575; UV-vis (MeOH) $\lambda_{max/nm}$= 299; Em (MeOH) $\lambda_{max/nm}$= 458; ^{1}H NMR (500 MHz, DMSO-d$_6$) δ 10.36 (s, 1H), 8.81 (s, 1H), 8.72 (s, 1H), 7.54 (s, 1H), 7.44 (d, *J*= 9.0 Hz, 1H), 7.33 (d, *J*= 8.9 Hz, 1H), 3.82 (s, 3H), 3.31 (s, 2H), 2.21 (t, *J*= 7.2 Hz, 1H), 1.97 (d, *J*= 7.2 Hz, 1H), 1.53 (d, *J*= 6.3 Hz, 4H), 1.37-1.27 (m, 2H); ^{13}C NMR (125 MHz, DMSO-d$_6$) δ 169.52, 161.48, 161.01, 156.43, 148.80, 147.65, 122.36, 119.60, 119.40, 117.70, 112.28, 56.30, 39.48, 32.65, 29.18, 26.50, 25.30; HRMS (ESI) *m/z* calcd. for $C_{18}H_{20}N_2O_5$ $[M+Na]^+$ 371.1219 found 371.1194.

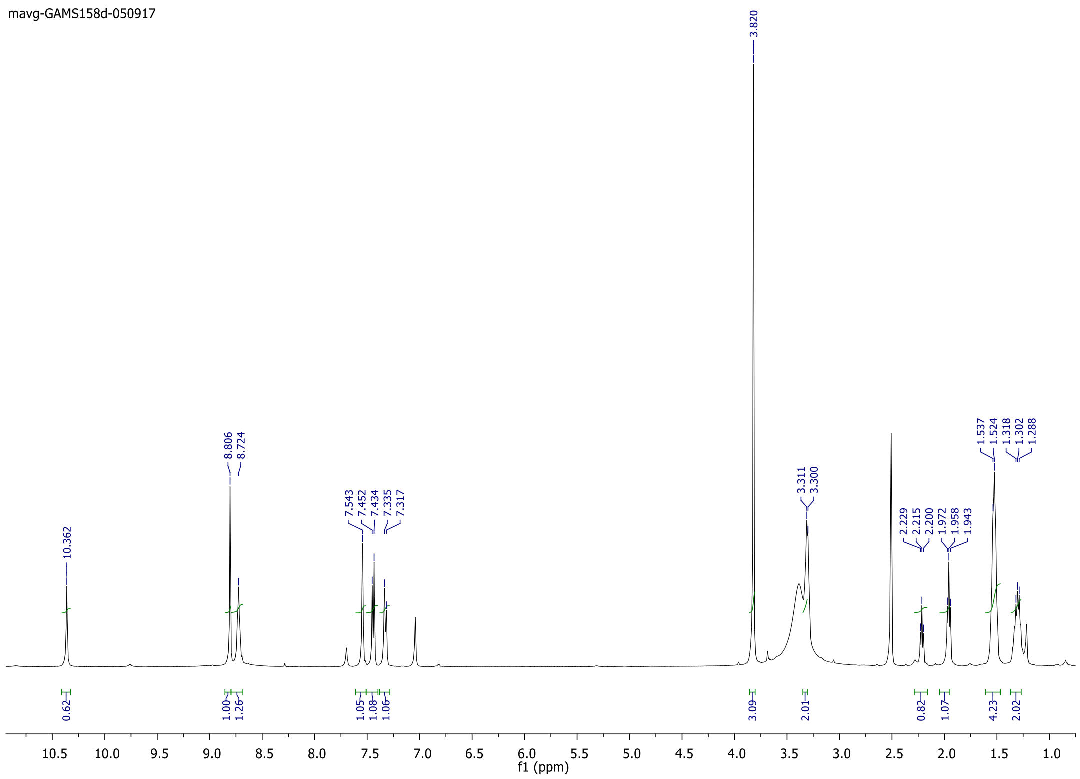

^{1}H spectrum of **7e** in DMSO-d$_6$, 500 MHz

mavg-GAMS158d-050917

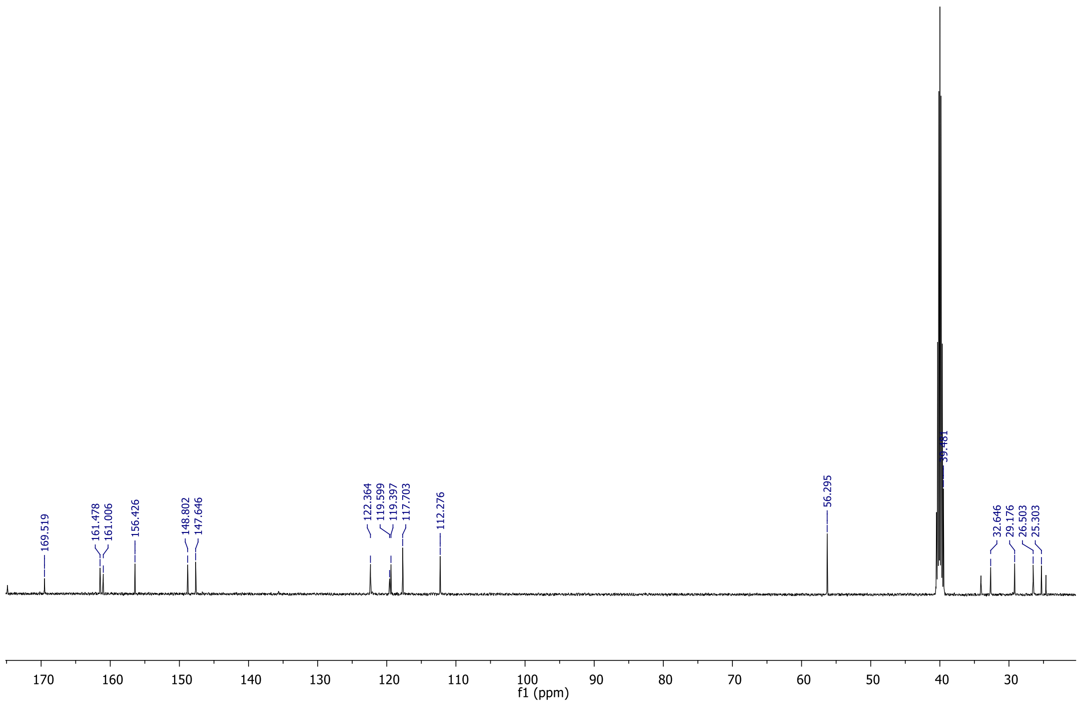

^{13}C spectrum of **7e** in DMSO-d$_6$, 125 MHz

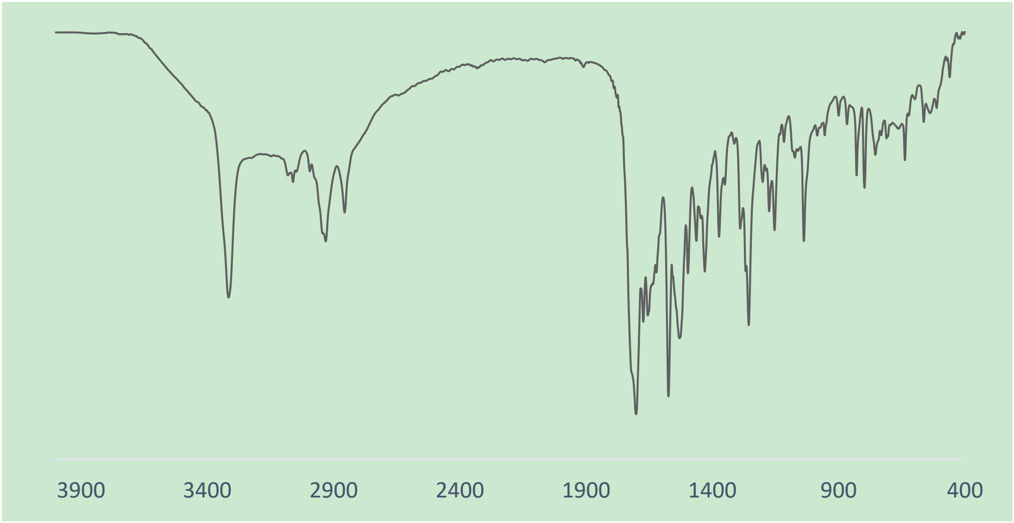

IR spectrum of **7e** in KBr

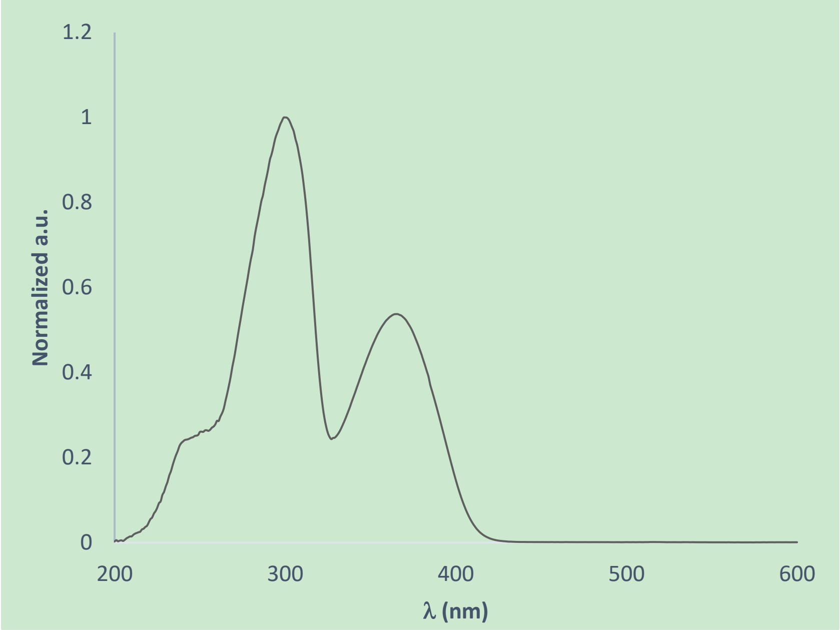

UV spectrum of **7e** in MeOH

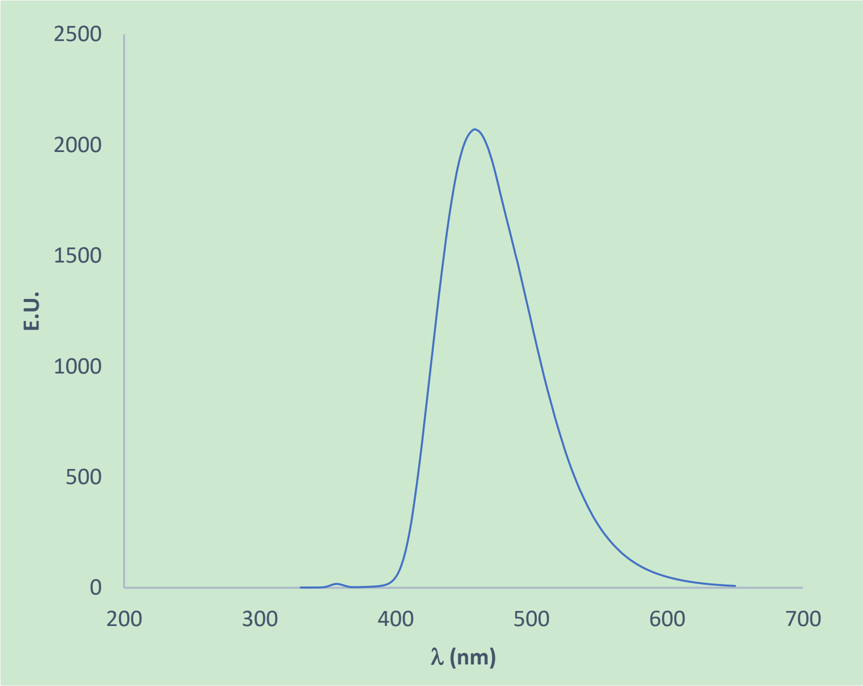

Emission spectrum of **7e** in MeOH at 10^{-6}M

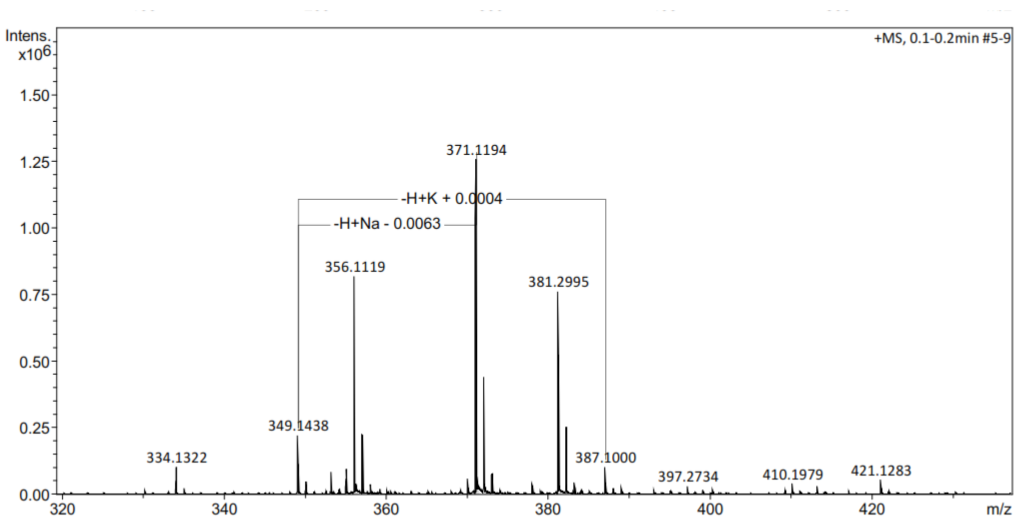

Mass spectrum, ESI(+), of **7e**

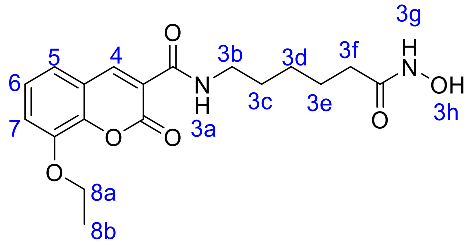

(7f) Light green solid (48% yield); mp 167-169 °C; IR (KBr) $\nu_{max/cm-1}$= 3335, 1933, 1716, 1539; UV-vis (MeOH) $\lambda_{max/nm}$= 311; Em (MeOH) $\lambda_{max/nm}$= 486; ^{1}H NMR (500 MHz, DMSO-d$_6$) δ 8.81 (s, 3H), 8.69 (s, 3H), 7.50 (d, *J*= 7.6 Hz, 3H), 7.41 (d, *J*= 8.0 Hz, 3H), 7.34 (t, *J*= 7.9 Hz, 3H), 4.20 (q, *J*= 6.8 Hz, 6H), 1.53 (s, 12H), 1.42 (t, *J*= 6.9 Hz, 11H), 1.32 (dd, *J*= 17.8, 7.6 Hz, 7H); ^{13}C NMR (125 MHz, DMSO-d$_6$) δ 169.51, 161.47, 160.65, 147.99, 145.97, 143.72, 125.53, 121.55, 119.61, 117.32, 65.02, 34.06, 32.64, 29.19, 26.50, 25.30, 15.02; HRMS (ESI) *m/z* calcd. for $C_{18}H_{22}N_2O_6$ $[M+H]^+$ 362.3820, found 362.3843.

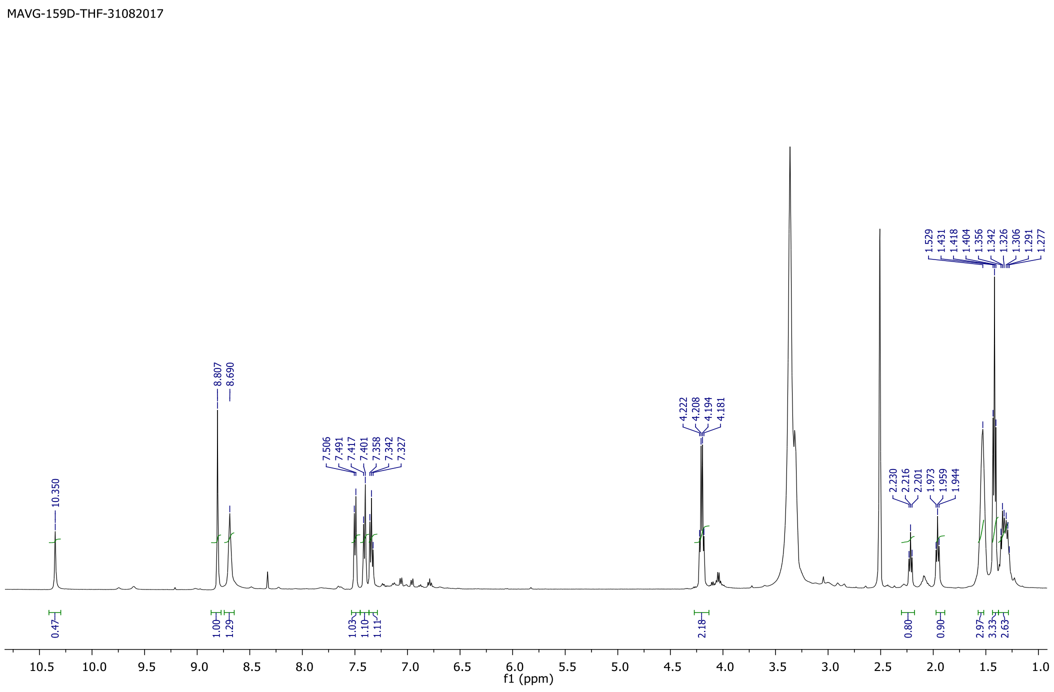

^{1}H spectrum of **7f** in DMSO-d_6, 500 MHz

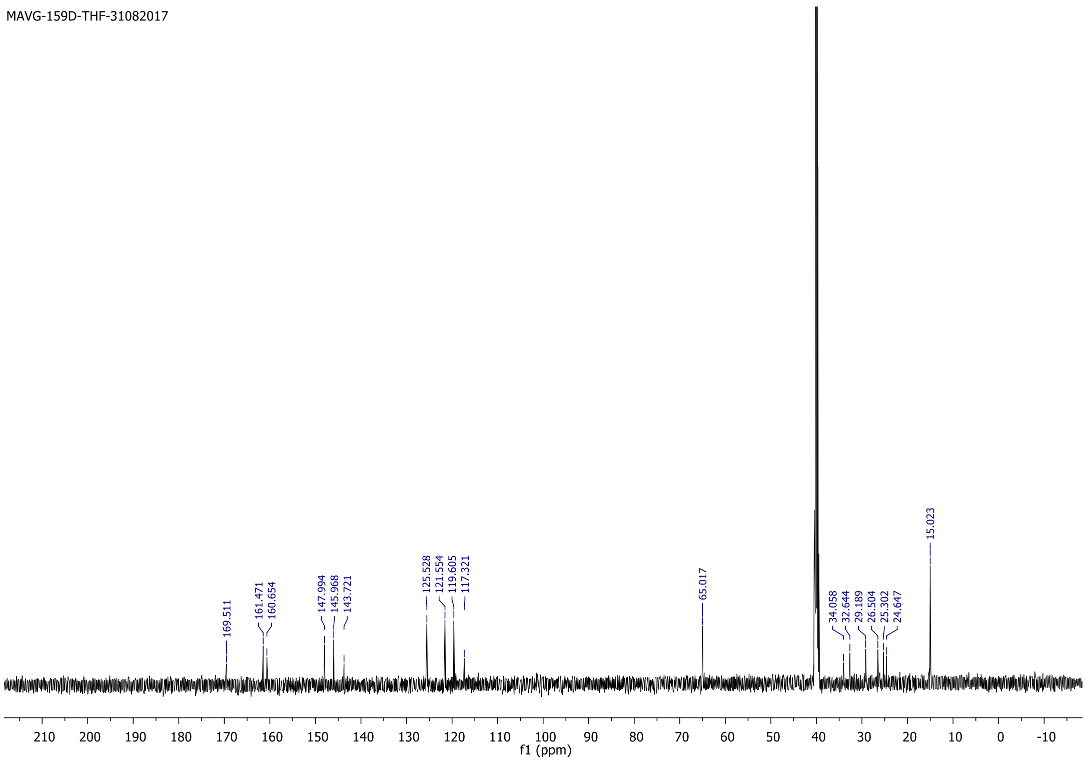

^{13}C spectrum of **7f** in DMSO-d_6, 125 MHz

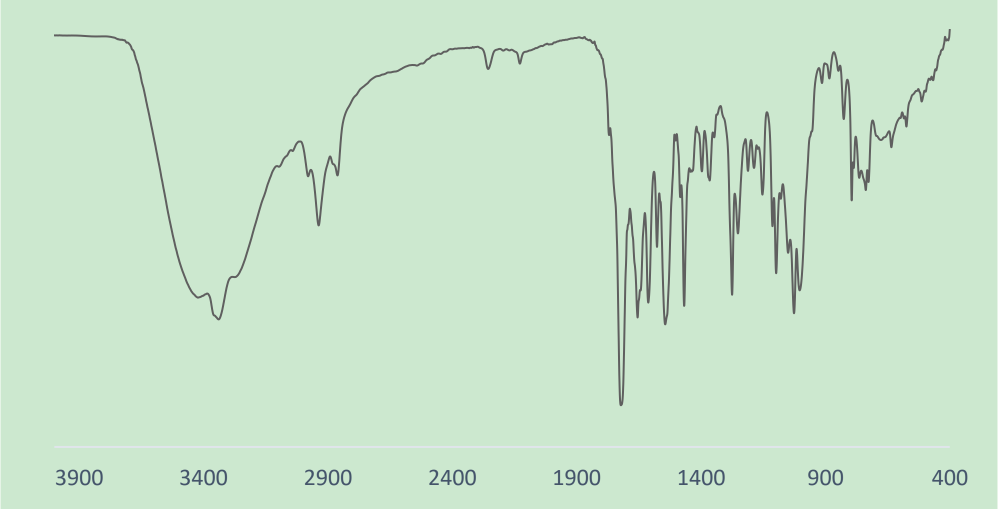

IR spectrum of **7f** in KBr

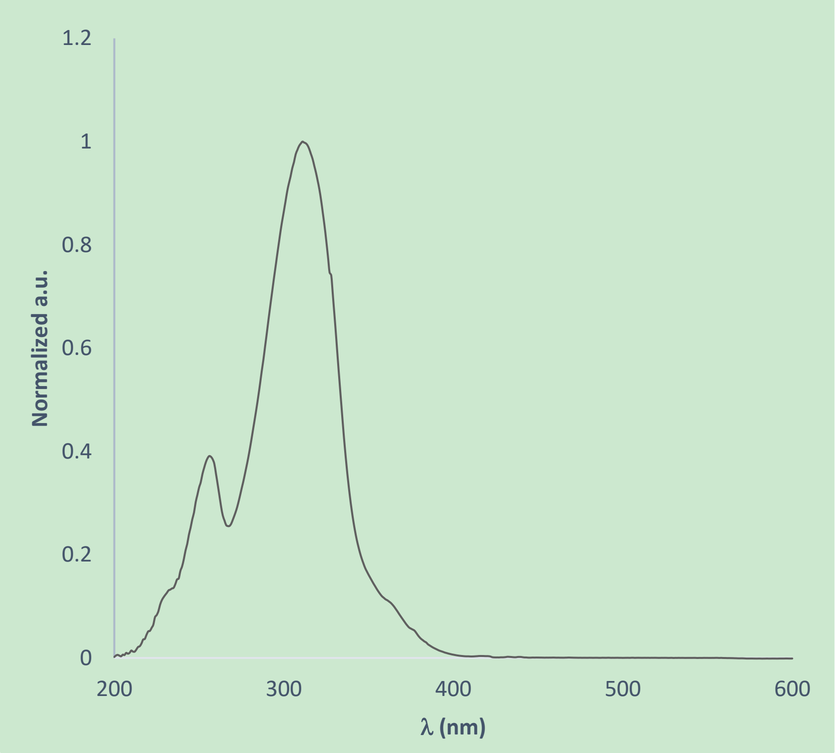

UV spectrum of **7f** in MeOH

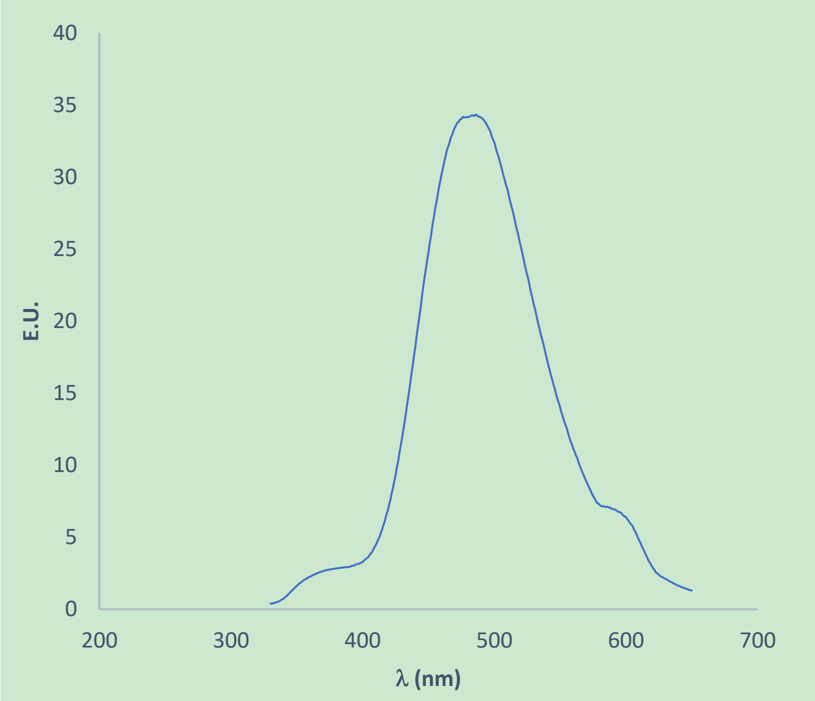

Emission spectrum of **7f** in MeOH at 10^{-6}M

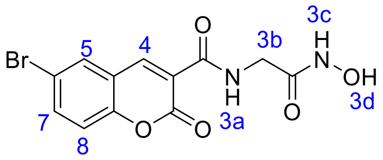

(7g) White solid (46% yield); mp 202-205 °C; IR (KBr) $\nu_{max/cm^{-1}}$= 3417, 3272, 1712, 1537; UV-vis (MeOH) $\lambda_{max/nm}$= 294; Em (MeOH) $\lambda_{max/nm}$= 424; ^{1}H NMR (500 MHz, DMSO-d_6) δ 9.01 (d, *J*= 4.6 Hz, 1H), 8.86 (s, 1H), 8.28 (s, 1H), 7.90 (d, *J*= 8.8 Hz, 1H), 7.50 (d, *J*= 8.9 Hz, 1H), 3.92 (d, *J*= 5.2 Hz, 2H); ^{13}C NMR (125 MHz, DMSO-d_6) δ 165.55, 161.34, 160.34, 153.43, 146.83, 136.83, 132.67, 120.78, 120.13, 118.91, 117.14, 41.13; HRMS (ESI) *m/z* calcd. for $C_{12}H_9BrN_2O_5$ $[M+H]^+$ 339.9695, found 339.9711.

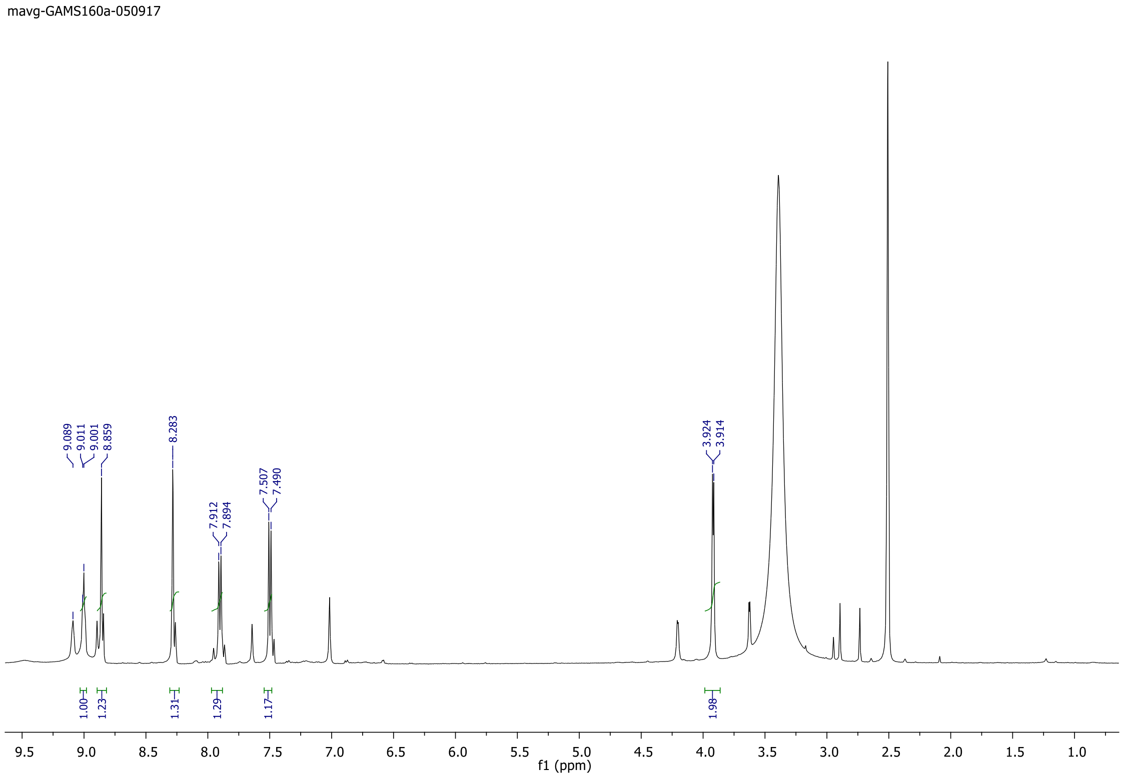

^{1}H spectrum of **7g** in DMSO-d$_6$, 500 MHz

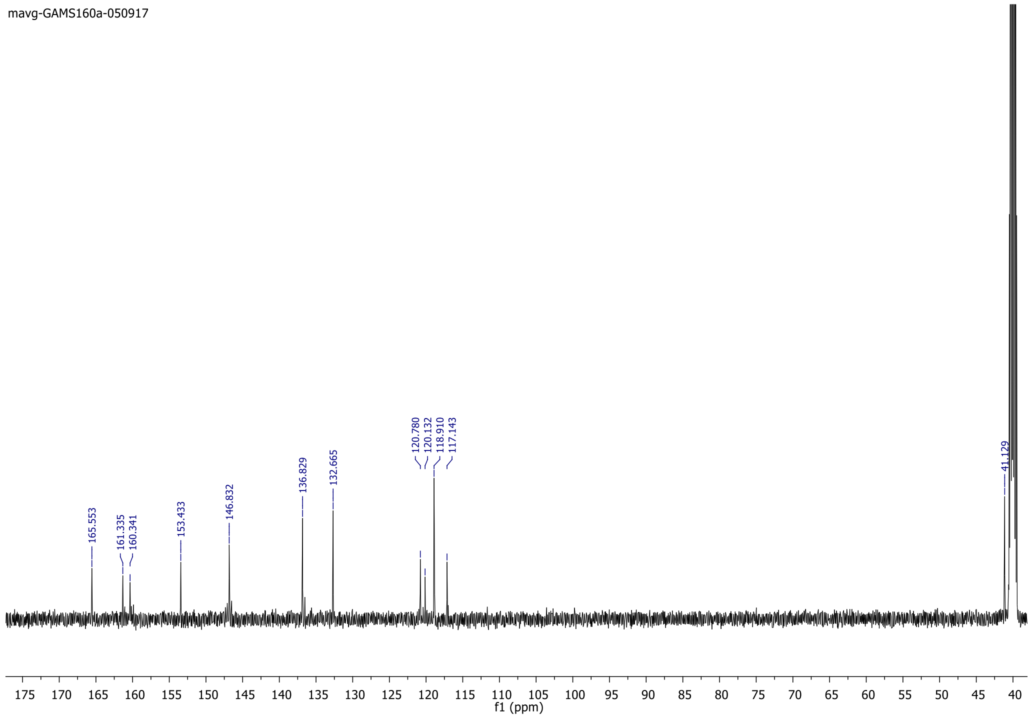

^{13}C spectrum of **7g** in DMSO-d$_6$, 125 MHz

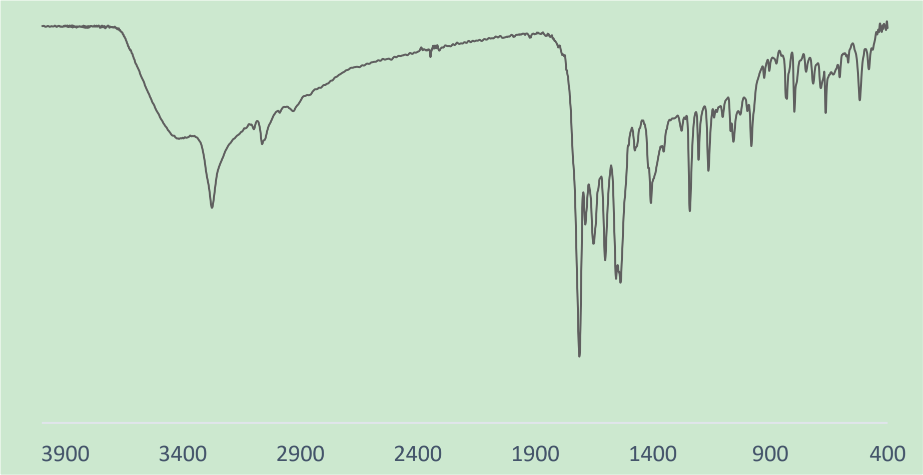

IR spectrum of **7g** in KBr

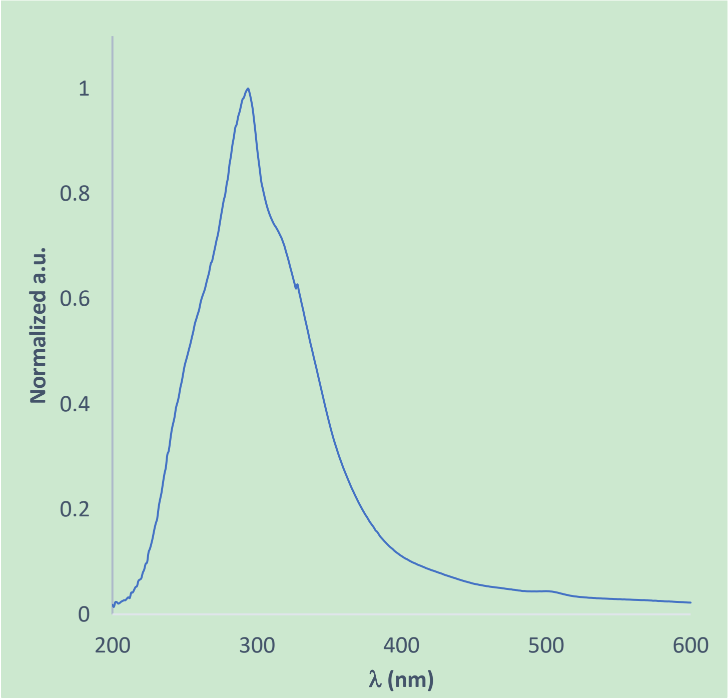

UV spectrum of **7g** in MeOH at 10^{-6} M

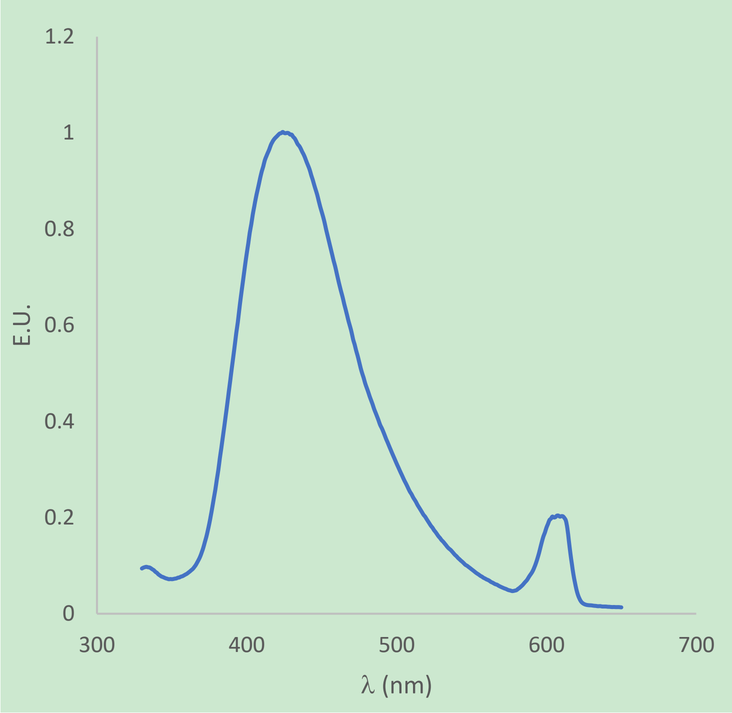

Emission spectrum of **7g** in MeOH at 10^{-6} M

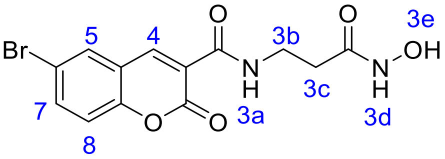

(7h) White solid (45% yield); mp 185-187 °C; IR (KBr) $\nu_{max/cm-1}$= 3354, 3040, 2854, 1708, 1538; UV-vis (MeOH) $\lambda_{max/nm}$= 295; Em (MeOH) $\lambda_{max/nm}$= 415; ^{1}H NMR (500 MHz, DMSO-d$_6$) δ 8.86 (d, *J*= 5.4 Hz, 1H), 8.83 (s, 1H), 8.26 (s, 2H), 7.89 (dd, *J*= 8.8, 1.8 Hz, 1H), 7.48 (d, *J*= 8.8 Hz, 1H), 3.53 (dd, *J*= 12.6, 6.4 Hz, 3H), 2.28 (t, *J*= 6.7 Hz, 2H; ^{13}C NMR (125 MHz, DMSO-d$_6$) δ 161.16, 160.30, 153.40, 146.78, 146.58, 136.72, 136.54, 132.57, 120.82, 118.86, 117.06, 36.19, 32.31; MS (ESI) *m/z* calcd. for $C_{13}H_{11}BrN_2O_6$ $[M+H]^+$ 356.1440, found 356.1252.

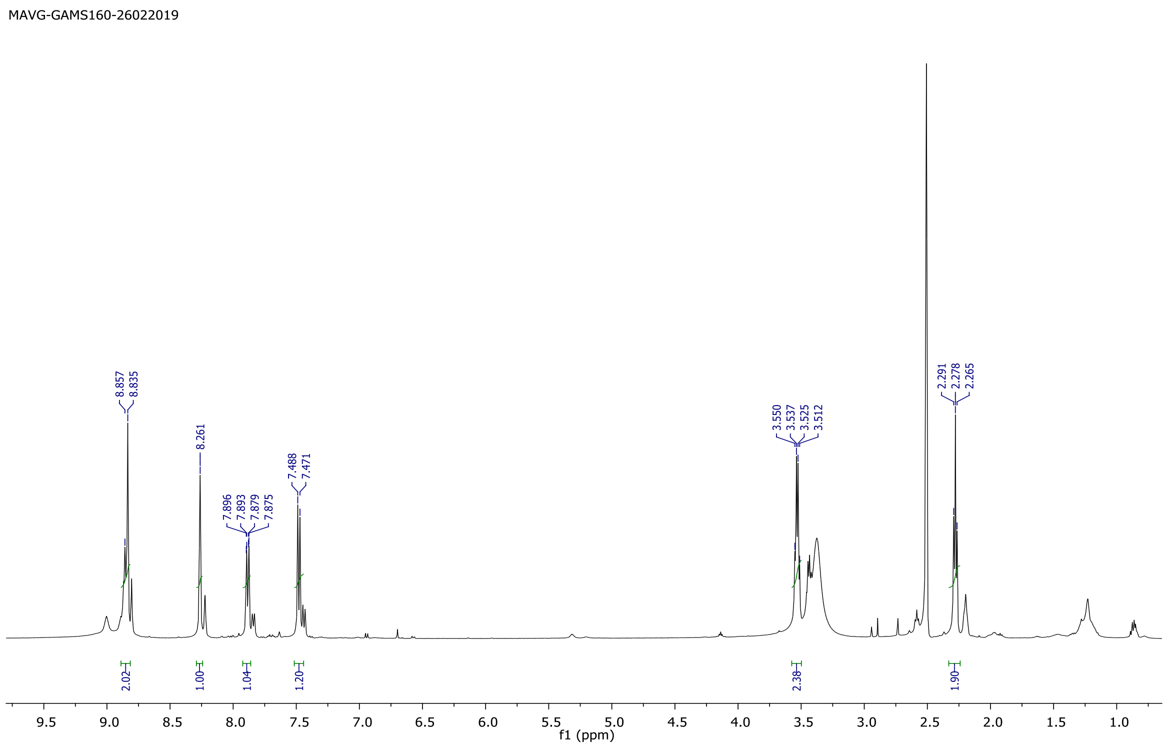

^{1}H spectrum of **7h** in DMSO-d_6, 500 MHz

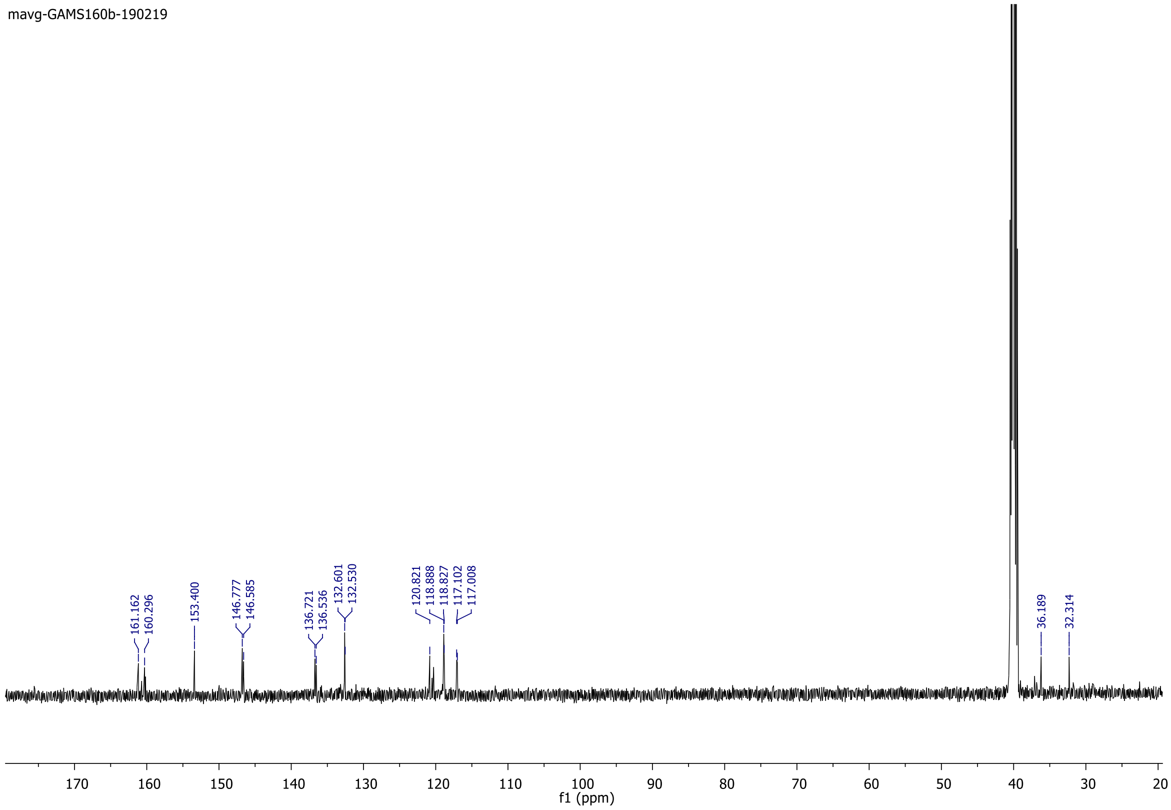

^{13}C spectrum of **7h** in DMSO-d_6, 125 MHz

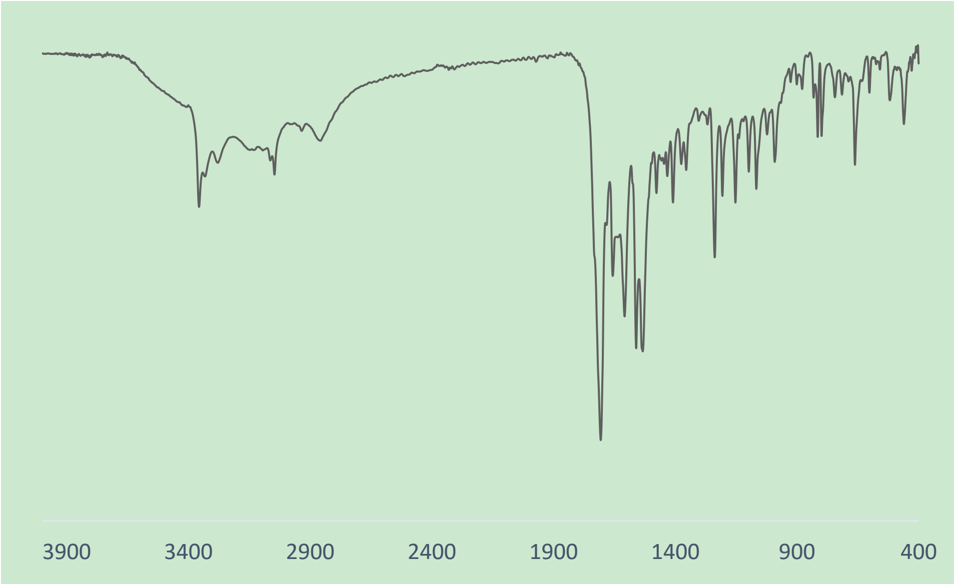

IR spectrum of **7h** in KBr

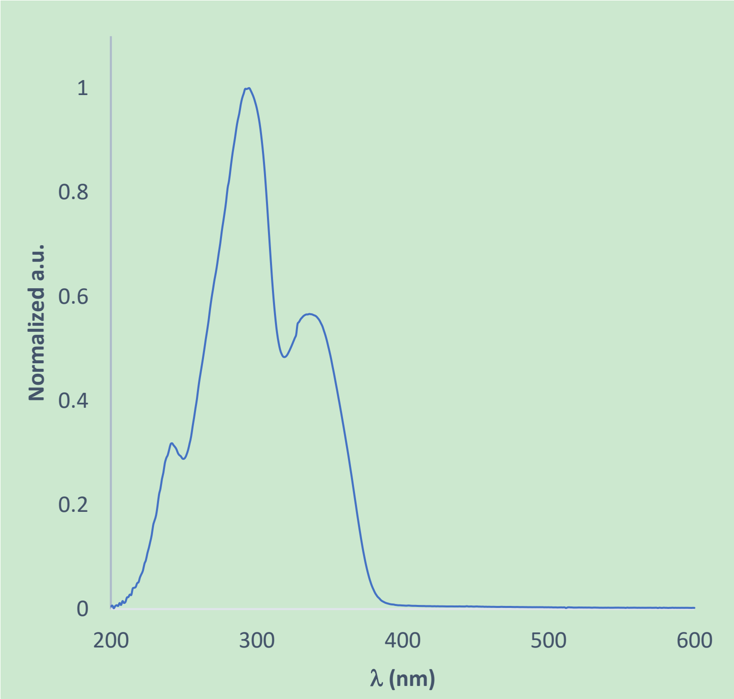

UV spectrum of **7h** in MeOH at 10^{-6} M

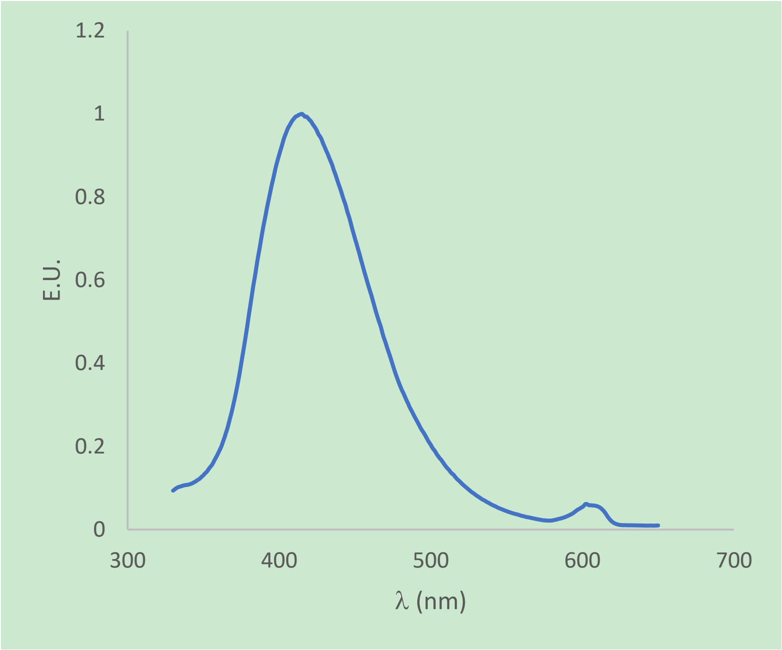

Emission spectrum of **7h** in MeOH at 10^{-6} M

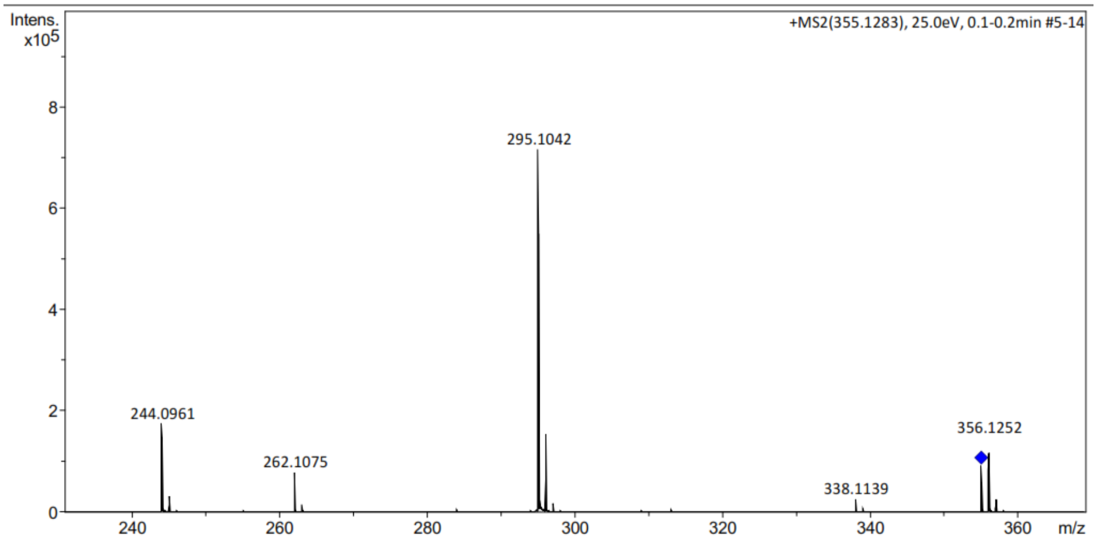

Mass spectrum, ESI(+), of **7h**

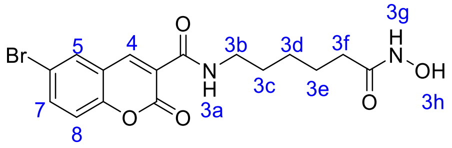

(7i) White solid (65% yield); mp 202-205 °C; IR (KBr) $\nu_{max/cm-1}$= 3344, 2934, 1724, 1559; UV-vis (MeOH) $\lambda_{max/nm}$= 283; Em (MeOH) $\lambda_{max/nm}$= 424; ^{1}H NMR (500 MHz, DMSO-d_6) δ 8.80 (d, *J*= 4.2 Hz, 2H), 8.67 (t, *J*= 5.7 Hz, 2H), 8.25 (dd, *J*= 6.4, 2.2 Hz, 2H), 7.88 (dd, *J*= 8.8, 2.3 Hz, 2H), 7.47 (dd, *J*= 8.6, 5.5 Hz, 2H), 3.30 (dd, *J*= 12.6, 6.4 Hz, 5H), 1.96 (t, *J*= 7.3 Hz, 4H), 1.52 (dt, *J*= 14.9, 7.4 Hz, 8H), 1.29 (dd, *J*= 14.7, 7.7 Hz, 4H); ^{13}C NMR (125 MHz, DMSO-d_6) δ 169.41, 162.78, 161.19, 160.36, 153.32, 146.40, 136.61, 132.51, 120.79, 118.87, 117.09, 39.52, 36.25, 29.16, 26.48, 25.32; HRMS (ESI) *m/z* calcd. for $C_{16}H_{17}BrN_2O_5$ $[M+H]^+$ 397.0321, found 397.1750.

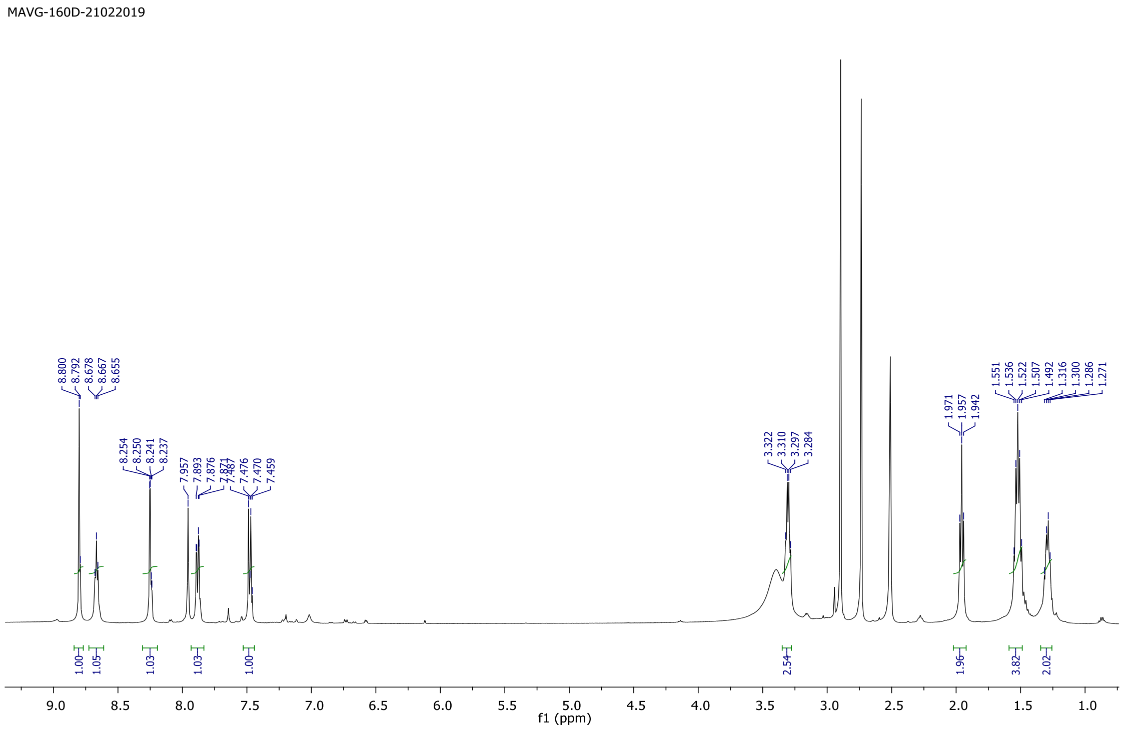

^{1}H spectrum of **7i** in DMSO-d_6, 500 MHz

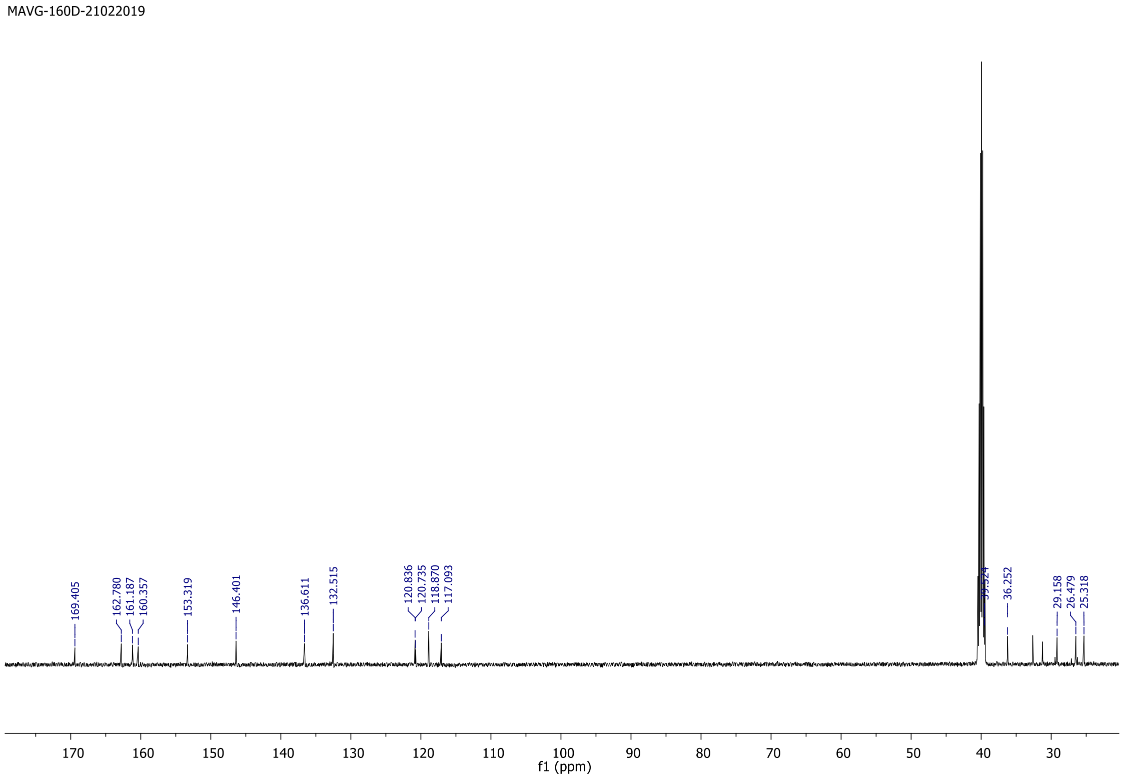

^{13}C spectrum of **7i** in DMSO-d_6, 125 MHz

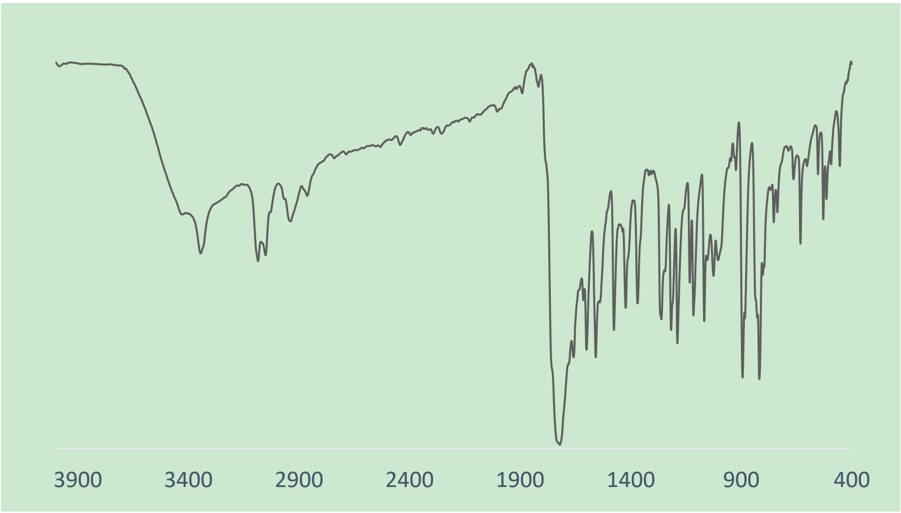

IR spectrum of **7i** in KBr

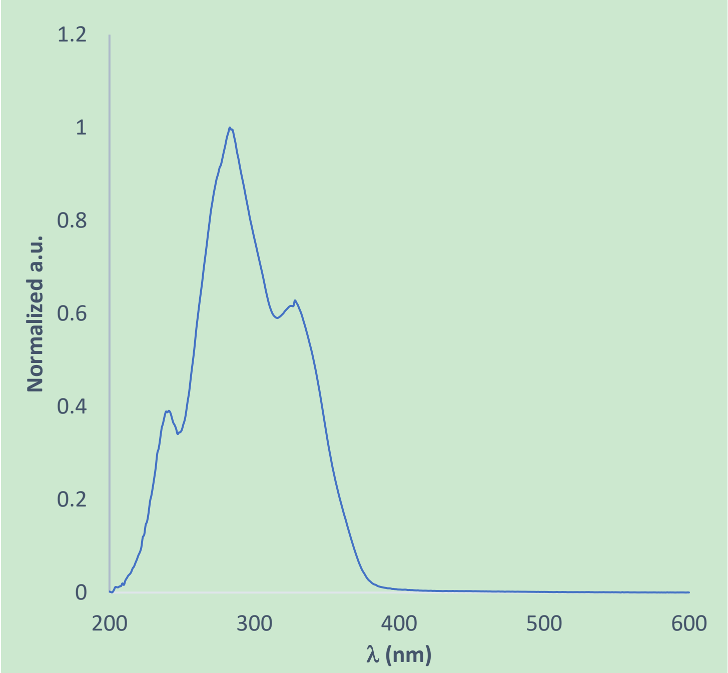

UV spectrum of **7i** in MeOH at 10^{-6} M

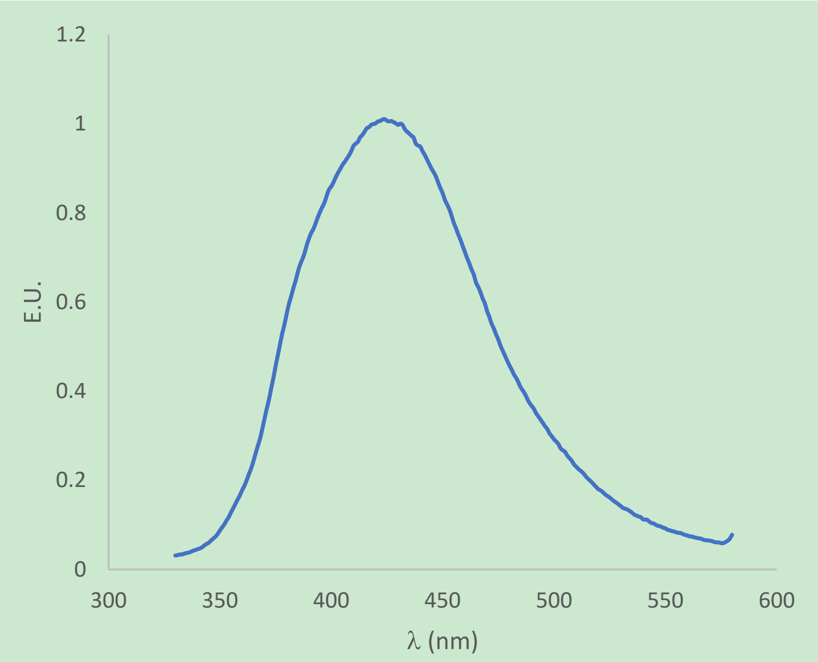

Emission spectrum of **7i** in MeOH at 10^{-6} M

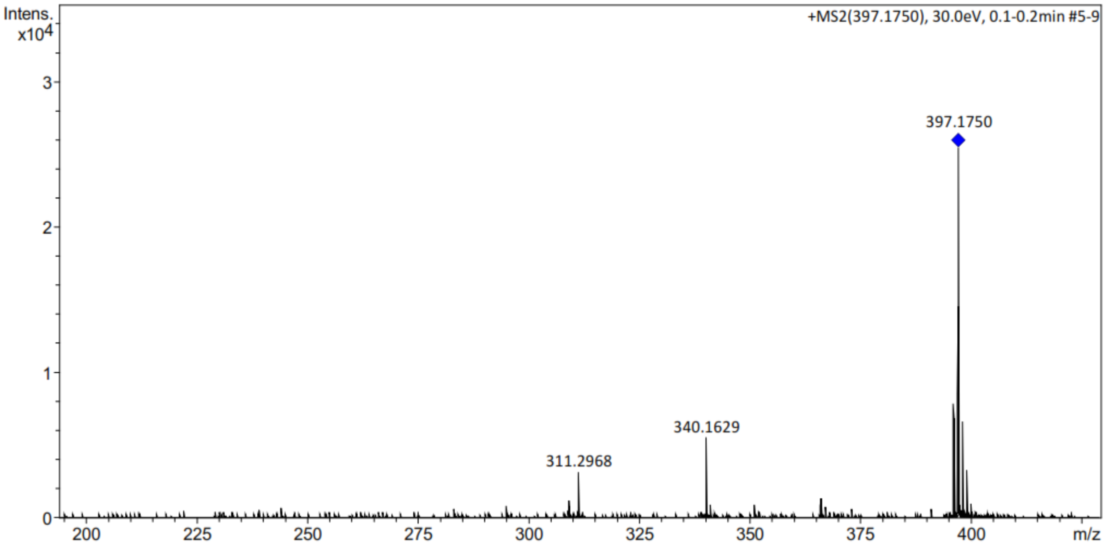

Mass spectrum, ESI(+), of **7i**

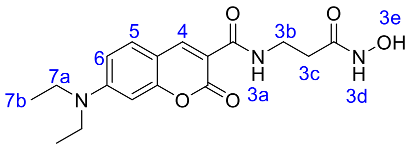

(7j) Yellow solid (82% yield); mp 126-128 ºC; IR (KBr) $\nu_{max/cm-1}$= 3231, 1699, 1616; UV-vis (MeOH) $\lambda_{max/nm}$= 419; Em (MeOH) $\lambda_{max/nm}$= 468; ^{1}H NMR (500 MHz, DMSO-d_6) δ 8.78 (t, *J*= 5.7 Hz, 1H), 8.65 (s, 2H), 7.67 (d, *J*= 9.0 Hz, 1H), 6.78 (d, *J*= 9.0 Hz, 1H), 6.60 (d, *J*= 1.3 Hz, 1H), 3.47 (q, *J*= 6.8 Hz, 4H), 2.25 (t, *J*= 6.6 Hz, 2H), 2.01 (t, *J*= 7.5 Hz, 2H), 1.13 (t, *J*= 6.9 Hz, 6H); ^{13}C NMR (125 MHz, DMSO-d_6) δ 169.13, 167.85, 162.60, 162.05, 157.70, 152.87, 148.16, 132.02, 110.57, 108.12, 108.10, 96.34, 44.79, 12.77; HRMS (ESI) *m/z* calcd. for $C_{17}H_{21}N_3O_5$ $[M+H]^+$ 348.1554, found 348.1570.

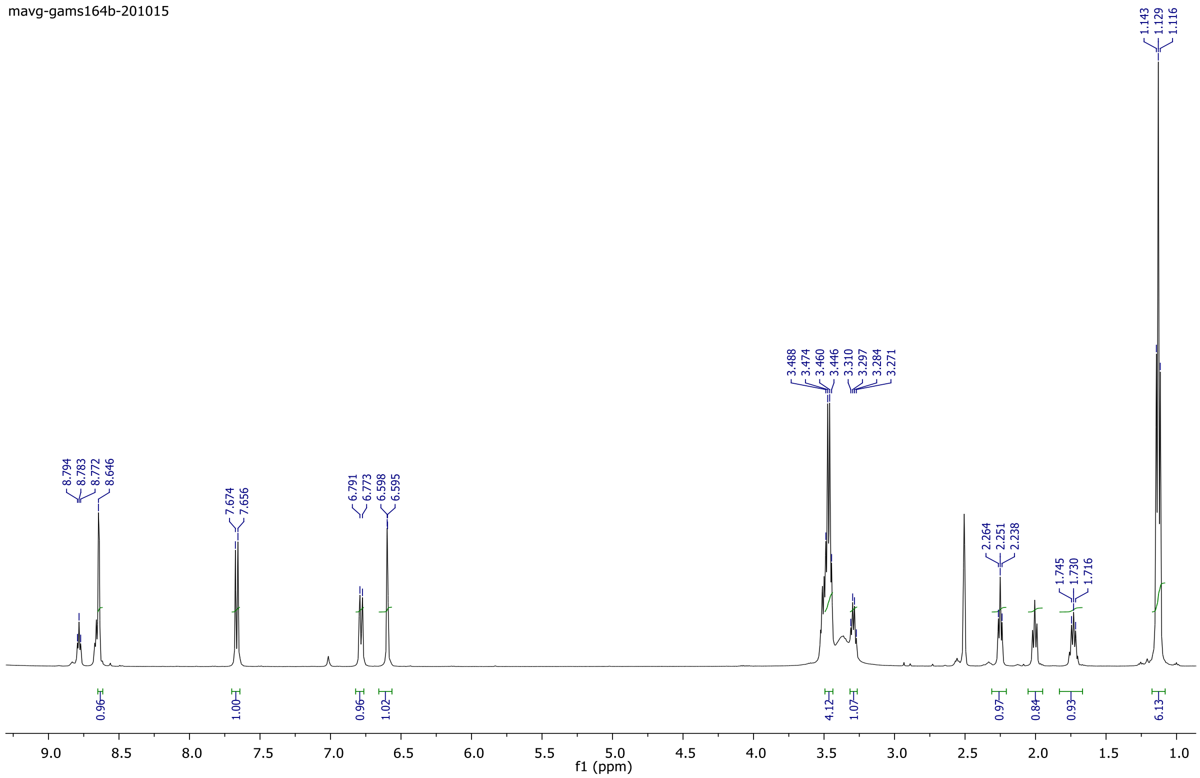

^{1}H spectrum of **7j** in DMSO-d$_6$, 500 MHz

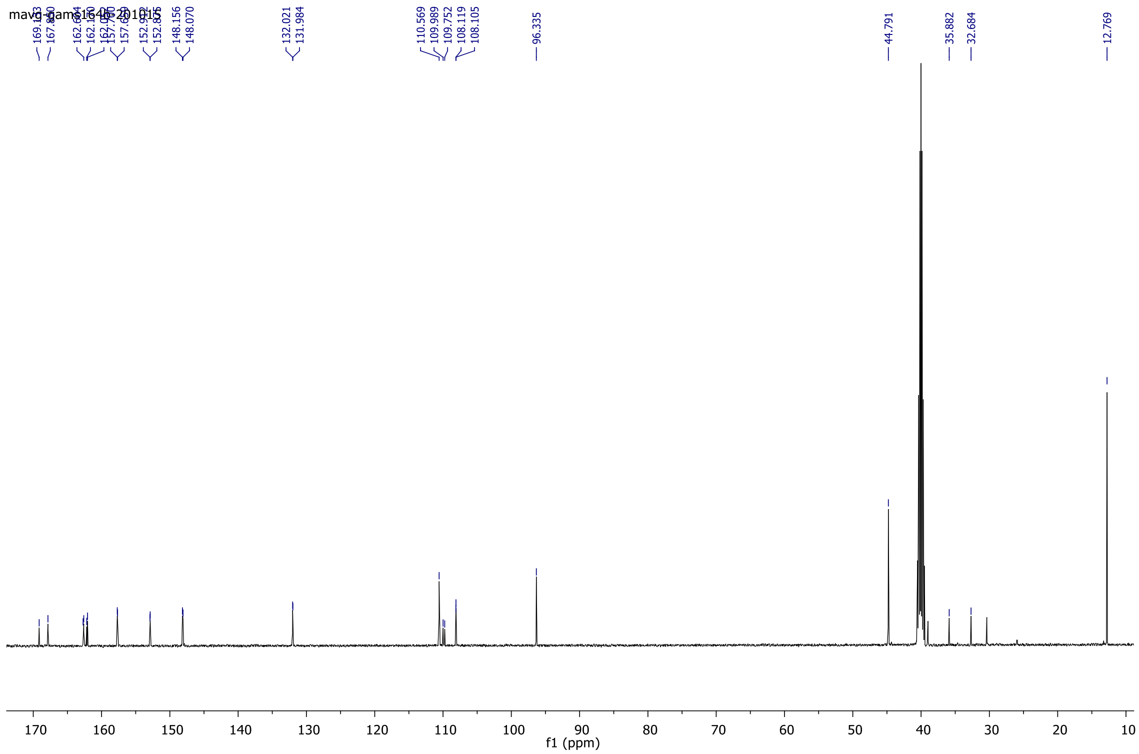

^{13}C spectrum of **7j** in DMSO-d$_6$, 125 MHz

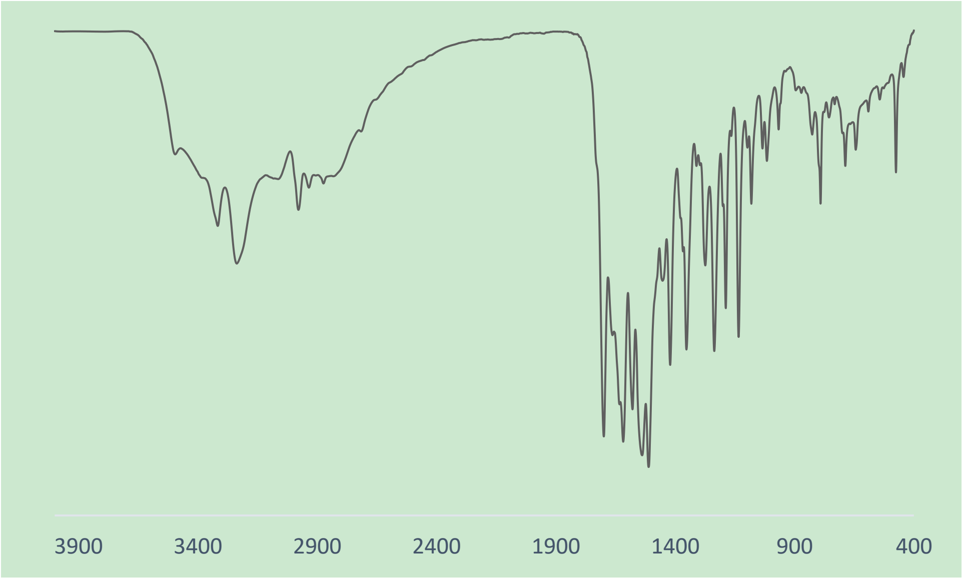

IR spectrum of **7j** in KBr

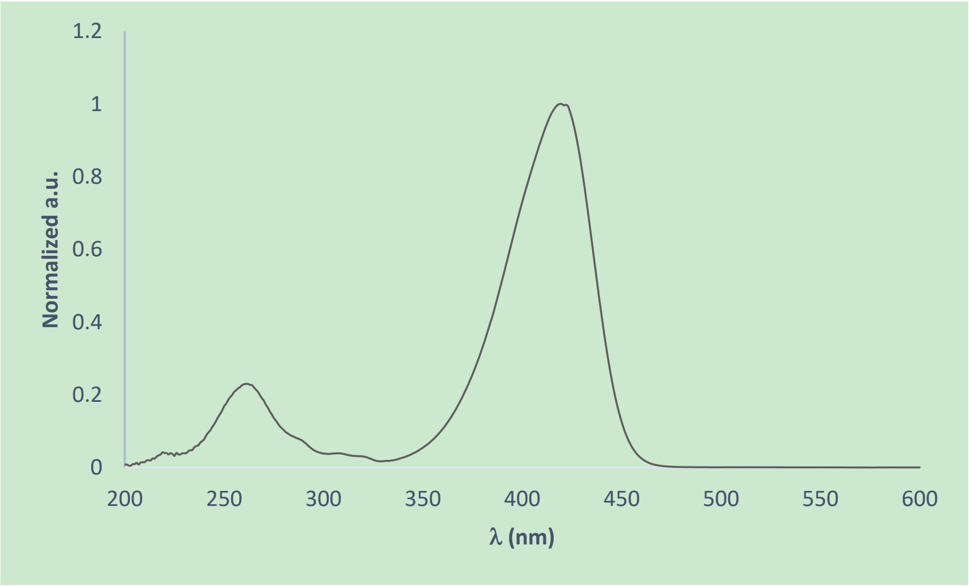

Absorption spectrum of **7j** in MeOH at 10^{-6}M

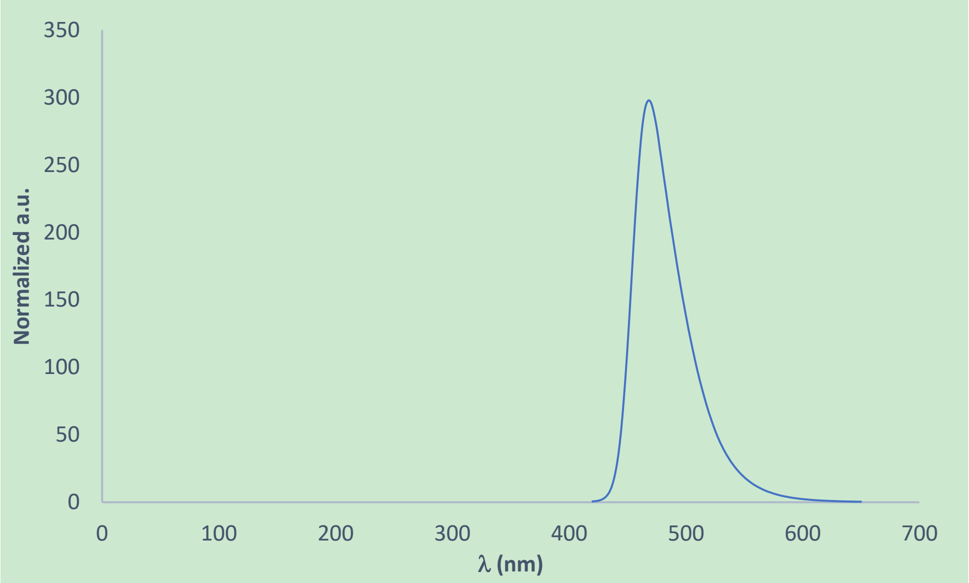

Emission spectrum of **7j** in MeOH at 10^{-6}M

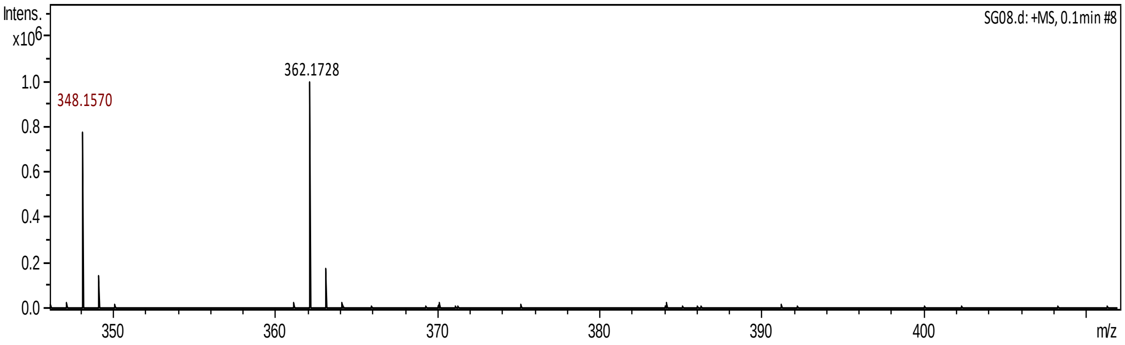

Mass spectrum, ESI(+), of **7j**

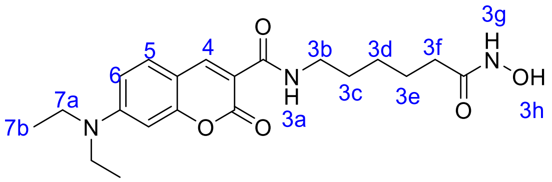

(7k) Yellow solid (82% yield); mp 173-174°C; IR (KBr) $\nu_{max/cm-1}$= 3122, 1700, 1618; UV-vis (MeOH) $\lambda_{max/nm}$= 418; Em (MeOH) $\lambda_{max/nm}$= 470; ^{1}H NMR (500 MHz, DMSO-d$_6$) δ 8.78 (t, *J*= 5.7 Hz, 1H), 8.65 (s, 2H), 7.67 (d, *J*= 9.0 Hz, 1H), 6.78 (d, *J*= 9.0 Hz, 1H), 6.60 (d, *J*= 1.3 Hz, 1H), 3.47 (q, *J*= 6.8 Hz, 4H), 2.25 (t, *J*= 6.6 Hz, 2H), 2.01 (t, *J*= 7.5 Hz, 2H), 1.13 (t, *J*= 6.9 Hz, 6H); ^{13}C NMR (125 MHz, DMSO-d$_6$) δ 169.13, 167.85, 162.60, 162.05, 157.70, 152.87, 148.16, 132.02, 110.57, 108.12, 108.10, 96.34, 44.79, 35.87, 32.68, 12.77. HRMS (ESI) *m/z* calcd. for $C_{20}H_{27}N_3O_5$ $[M+H]^+$ 389.1951, found 389.1969.

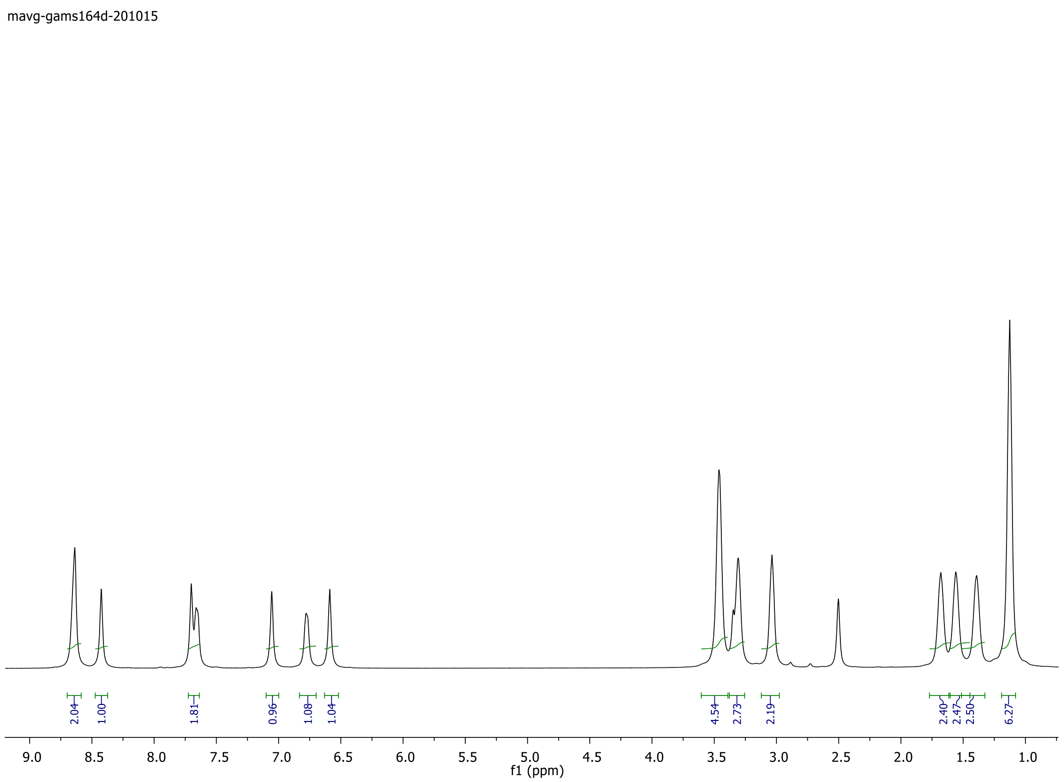

^{1}H spectrum of **7k** in DMSO-d$_6$, 500 MHz

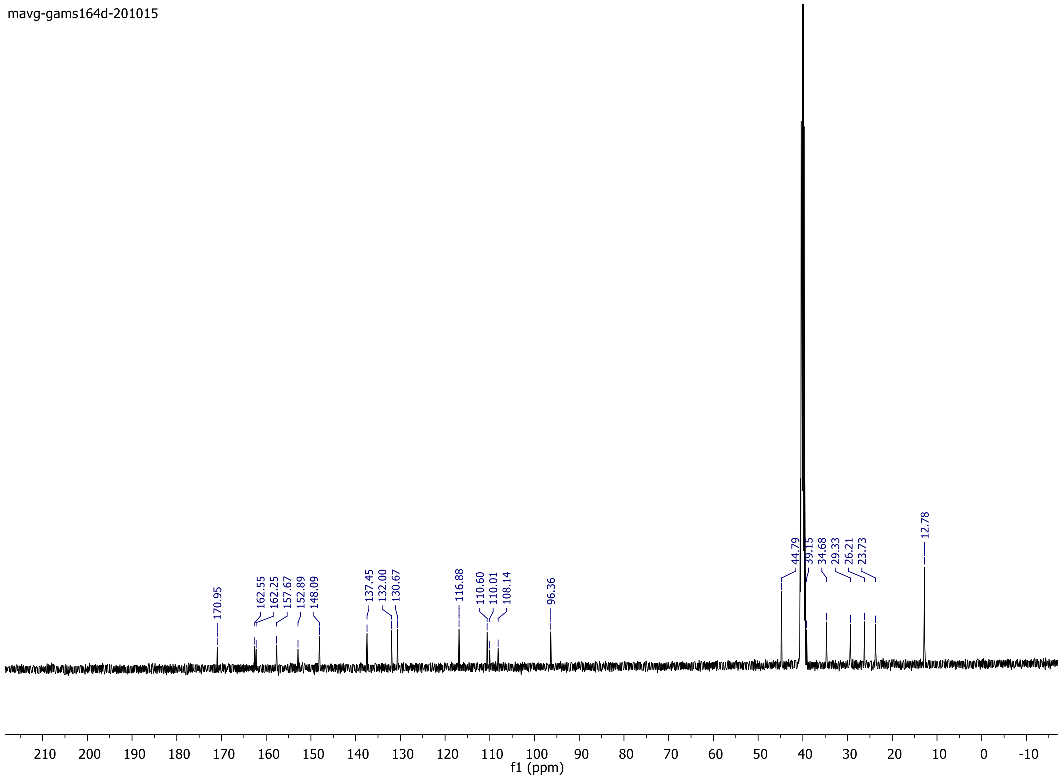

^{13}C spectrum of **7k** in DMSO-d$_6$, 500 MHz

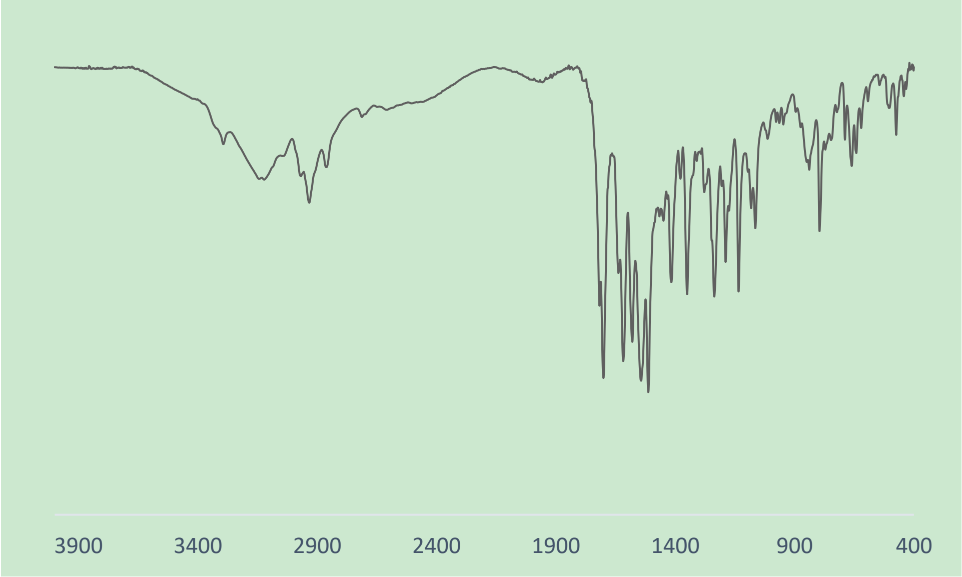

IR spectrum of **7k** in KBr

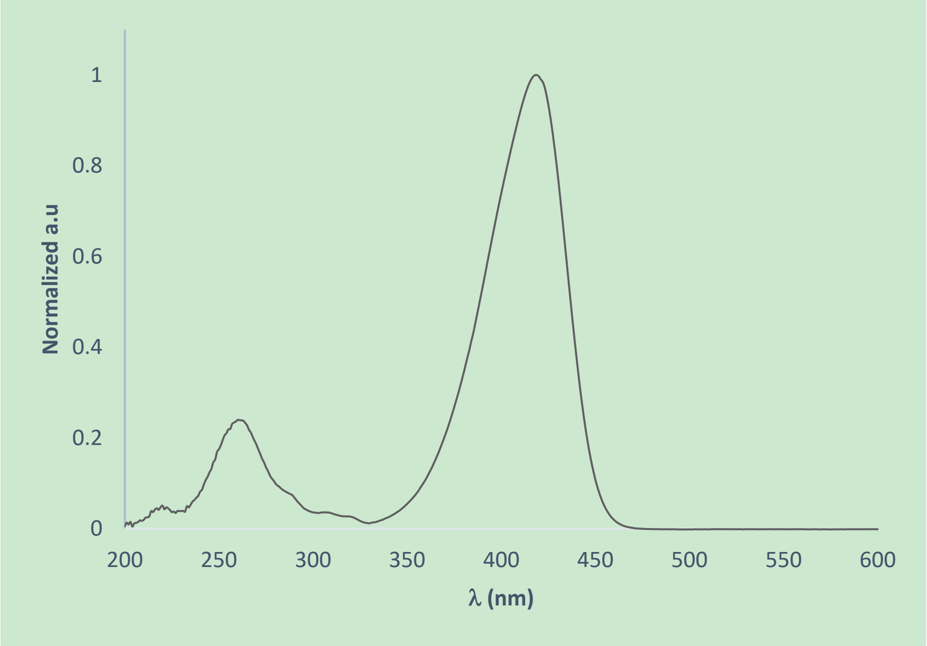

Absorption spectrum of **7k** in MeOH at 10^{-6}M

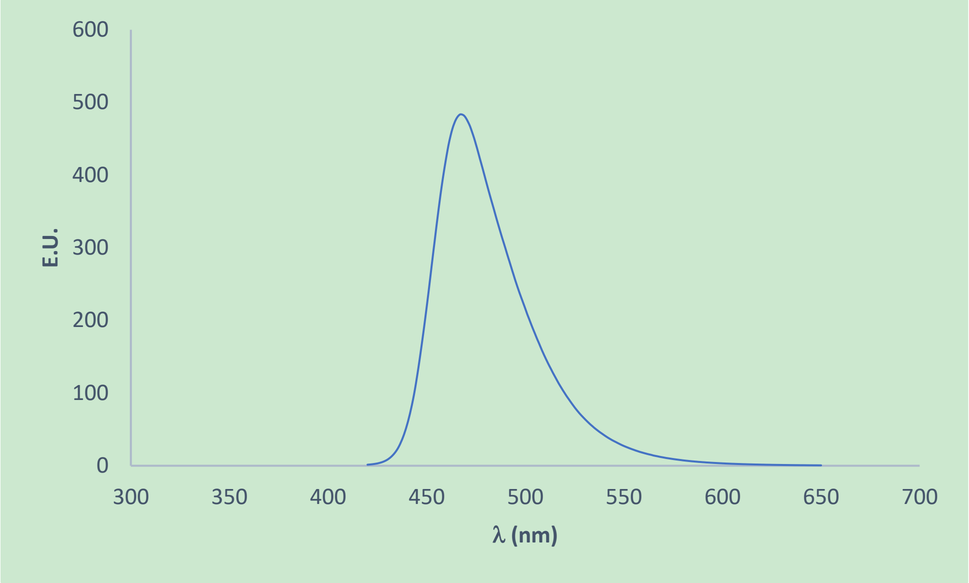

Emission spectrum of **7k** in MeOH at 10^{-6}M

2. Antiproliferative activity

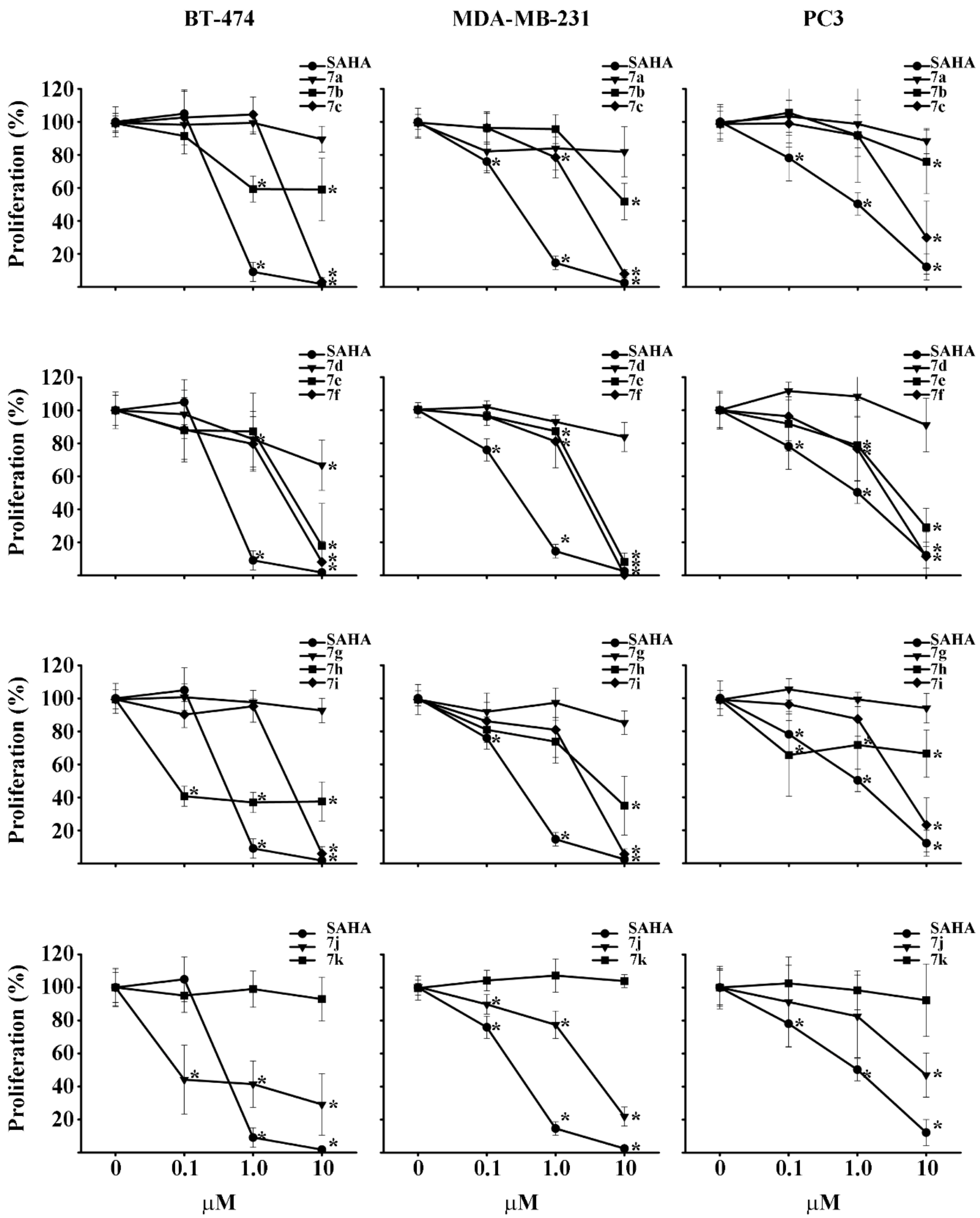

Figure S1. Antiproliferative effect of compounds **7a-k** on cancer cells. BT-474, MDA-MB-231 and PC3 cells were incubated in the presence of different concentrations of the test compounds or SAHA. Cell proliferation was evaluated by using the SRB assay. Data are expressed as the mean ± SD of triplicate determinations and represent at least three different experiments. Data from vehicle-treated cells were normalized to 100%. *P < 0.05 vs vehicle.

3. **Docking of coumarin and 4-hydroxycoumarin with HDAC1, HDAC6 and HDAC8.**

The docking between the molecules (coumarin and hydroxycoumarin) and HDAC1, HDAC6 y HDAC8, show similar energetic interactions of both ligands.

Table SI-1. Docking between coumarin and 3, 4, 5, 6 and 7-hydroxycoumarin, and HDAC isoforms (HDAC1, HDAC6 and HDAC8), the negative interaction free energies.

Ligand	HDAC1*	HDAC6*	HDAC8*
Coumarin	-75.11	-66.83	-76.24
3-hydroxycoumarin	-68.28	-64.57	-79.45
4-hydroxycoumarin	-74.44	-68.06	-81.28
5-hydroxycoumarin	-78.244	-68.07	-81.71
6-hydroxycoumarin	-71.54	-72.58	-79.29
7-hydroxycoumarin	-73.88	-74.50	-82.45

*Kcal/mol

The docking between the 3, 4, 5, 6 and 7-hydroxycoumarin and HDAC1, HDAC6 y HDAC8, revealed unions mainly of π-staking with the aromatic groups of the amino acids of the enzymatic active site and the coumarin ring of both ligands (Figure S2). We detected that, there is a possible monodentate coordination with Zn (II) for 3, 5 and 7-hydroxycoumarin.

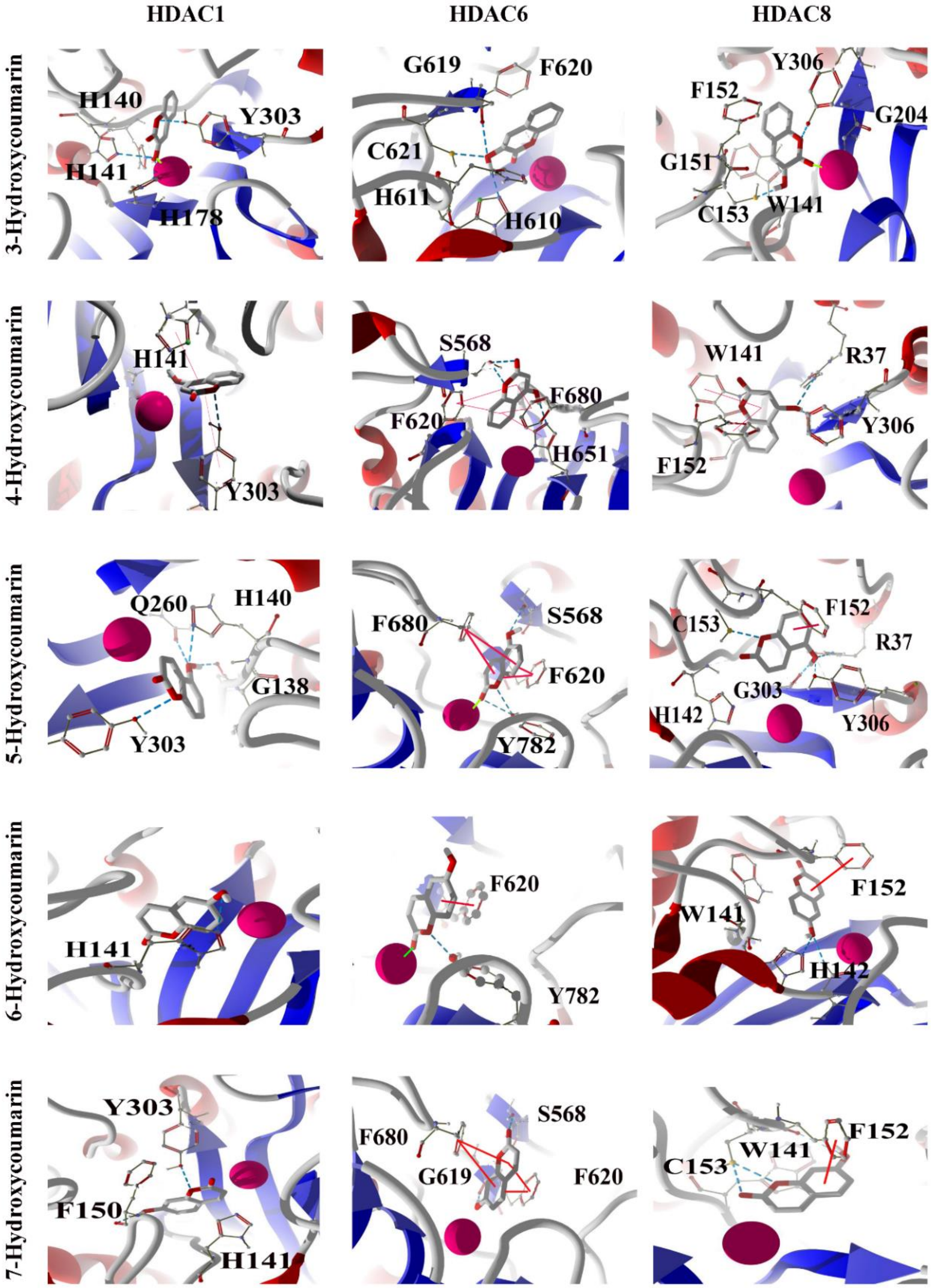

Figure S2. Interactions between the hydroxycoumarins and the residue in the active site of HDAC1, HDAC6 and HDAC8. The stacking interactions are show as red lines, hydrogen bond interactions are represented as blue dotted lines and the coordination bond with Zinc is represented as a green line.
